# Do longer sequences improve the accuracy of identification of forensically important Calliphoridae species?

**DOI:** 10.7717/peerj.5962

**Published:** 2018-12-17

**Authors:** Sara Bortolini, Giorgia Giordani, Fabiola Tuccia, Lara Maistrello, Stefano Vanin

**Affiliations:** 1 Department of Life Sciences, University of Modena and Reggio Emilia, Reggio Emilia, Italy; 2 School of Applied Sciences, University of Huddersfield, Huddersfield, United Kingdom

**Keywords:** ND5, COI, PER, Diptera, EF-1α, Maximum-likelihood, Phylogeny

## Abstract

Species identification is a crucial step in forensic entomology. In several cases the calculation of the larval age allows the estimation of the minimum Post-Mortem Interval (mPMI). A correct identification of the species is the first step for a correct mPMI estimation. To overcome the difficulties due to the morphological identification especially of the immature stages, a molecular approach can be applied. However, difficulties in separation of closely related species are still an unsolved problem. Sequences of 4 different genes (COI, ND5, EF-1α, PER) of 13 different fly species collected during forensic experiments (*Calliphora vicina, Calliphora vomitoria, Lucilia sericata, Lucilia illustris, Lucilia caesar, Chrysomya albiceps, Phormia regina, Cynomya mortuorum, Sarcophaga* sp*., Hydrotaea* sp*., Fannia scalaris, Piophila* sp*., Megaselia scalaris*) were evaluated for their capability to identify correctly the species. Three concatenated sequences were obtained combining the four genes in order to verify if longer sequences increase the probability of a correct identification. The obtained results showed that this rule does not work for the species * L. caesar* and * L. illustris*. Future works on other DNA regions are suggested to solve this taxonomic issue.

## Introduction

Species identification is a crucial step in forensic entomology. In particular, the calculation of the age of the insects collected from a cadaver or a crime scene allows the estimation of the time of oviposition that, except in case of myiasis, can be considered as the minimum Post-Mortem Interval (mPMI) (Erzinclioĝlu, 1983; Marchenko, 1982; Smith, 1986; Amendt et al., 2007; Vanin et al., 2017). This method is particularly precise when insects of the first colonization wave—mainly Diptera in the family Calliphoridae, Sarcophagidae and Muscidae—are considered. Insect development is temperature dependent and each species has a different growth rate. Thus, the correct identification of the species leads to an accurate mPMI estimation. Species identification is classically performed by morphological analysis of the specimens, but the lack of complete identification keys for immature stages represents a limitation to this approach. In the last twenty years, to overcome this limit, several authors have suggested a DNA-based identification method which is frequently used today because of the new sequencing technologies and the relative reduction of the costs (Benecke, 1998; Sperling, Anderson & Hickey, 1994; Stevens & Wall, 1996). Most of the publications about identification of forensically important species focused on the analysis of the genes coding for the cytochrome c oxidase subunit I (COI) as summarized by Tuccia and co-workers (Tuccia, Giordani & Vanin, 2016) and cytochrome c oxidase subunit II (COII) (Sperling, Anderson & Hickey, 1994; Aly, Wen & Wang, 2013; Boehme, Amendt & Zehner, 2012; Malgorn & Coquoz, 1999; Wallman & Donnellan, 2001; Xiong et al., 2012). Tested target markers other than COI and COII were: Cytb (GilArriortua et al., 2013; GilArriortua et al., 2014; GilArriortua et al., 2015; Giraldo, Uribe & Lopez, 2011), ND1 (Giraldo, Uribe & Lopez, 2011), ND5 (Zaidi et al., 2011; Zehner et al., 2004), 28S rDNA (Gibson et al., 2011; McDonagh & Stevens, 2011; Stevens & Wall, 2001; Tourle, Downie & Villet, 2009), mt16S rDNA (Guo et al., 2014; Li et al., 2010), CAD (Gibson et al., 2011; Meiklejohn et al., 2013; Schnell Schühli, Barros de Carvalho & Wiegmann, 2007), EF-1α (Gibson et al., 2011; Schnell Schühli, Barros de Carvalho & Wiegmann, 2007; McDonagh, García & Stevens, 2009), ITS1 (Zaidi et al., 2011), ITS2 (GilArriortua et al., 2014; GilArriortua et al., 2015; Zaidi et al., 2011; Ferreira et al., 2011; Nelson, Wallman & Dowton, 2008; Song, Wang & Liang, 2008; Yusseff-Vanegas & Agnarsson, 2017), PER (Guo et al., 2014) and Bicoid (Park et al., 2013). Analysis of mitochondrial DNA (mtDNA), in particular COI gene, seems to provide good species identification among Diptera, although a correct identification is still problematic for closely related species (Tourle, Downie & Villet, 2009; Harvey et al., 2008; Sonet et al., 2012). Nuclear DNA, especially ITS2 gene, presents a lack of intra-specific genetic divergence but high inter-specific variation in the genus *Lucilia* Robineau-Desvoidy, 1830, leading to a better resolution of closely related species (GilArriortua et al., 2014; GilArriortua et al., 2015). ITS2 was able to fully resolve the relationship within the species in the genus *Cochliomyia* Townsend 1915 (Yusseff-Vanegas & Agnarsson, 2016), otherwise, the same gene appeared to be not useful for *Chrysomya* Robineau-Desvoidy, 1830 genus studies (Nelson, Wallman & Dowton, 2008).

Previous works indicate that the combination of nuclear and mitochondrial markers is a much more accurate approach for species identification. In a recent paper, the study of Caribbean blow-flies through DNA markers highlights the possibility to resolve phylogenetic relations using a combination of COI and ITS2 genes. In fact, COI failed in demonstrating a monophyly in recently diverged species, leading to uncertain identification. The addition of a second nuclear marker, such as ITS2, increases certainty in species identification (Yusseff-Vanegas & Agnarsson, 2017). McDonagh and co-worker tested a multi- loci approach (28S rRNA, COI and EF-1α) finding that multiple-gene phylogenies permit the use of genes that have evolved at different rates, and also allow the identification of experimental errors in species identification and sequencing (McDonagh & Stevens, 2011). Zaidi et al. (2011) based the identification of Diptera species on five genes and demonstrated that such a multi-gene approach allows to overcome and clarify the misdiagnosis given by a single gene identification.

We focused our attention on dipteran specimens morphologically identified in order to evaluate the accuracy of the molecular approach in the identification of forensically important species. Sequences of four different markers, two mitochondrial (COI and ND5) and two nuclear (EF-1α and PER) were used. According to literature, identification based on a single gene had showed discordant outcomes compared with morphological results (Meier et al., 2006; Vilgalys, 2003) especially in the case of closely related species. In order to clarify the accuracy of a molecular multiple-loci approach in the identification of forensically important species, we built concatenated sequences using the four different markers.

## Materials and Methods

Eighty specimens (Table 1) were collected between 2011 and 2014 in Italy (Emilia Romagna, Veneto and Calabria), England (West Yorkshire) and Belgium, and preserved in absolute ethanol. The specimens were observed under the microscope and identified using taxonomic keys (Table 2). DNA extraction from adult insects was performed on abdominal tissues carefully dissected, to prevent external contaminations and to preserve the external structure of the insect for future examination. Full puparia and larvae were instead entirely processed, after a photographic documentation to allow further observations. DNA was extracted using the QIAamp DNA Mini Kit^®^ (QIAGEN, Germantown, MD, USA), following the manufacture protocol “DNA Purification from Tissue” (QIAGEN). Sterile deionized water was used to elute the DNA. The amplification of DNA was carried out on selected regions of four genes. In particular the barcoding region of the COI gene, and portions within ND5, EF-1α and PER genes were amplified. COI gene was selected as mainstream component of the analysis, and conversely ND5, EF-1α and PER genes were selected because only a little information is available on these DNA portions. A list of the used primers and their specifications are reported in Table 3. PCR was performed using 4 µl of the DNA extract as template for a 40 µl reaction final volume, using 0.5 µl of GoTaq^®^ Flexi DNA Polymerase (Promega, Madison, WI, USA) per reaction. Each 40 µl reaction consisted of 8 µl of 5X Colorless GoTaq^®^ Flexi Buffer (Promega), 4 µl of MgCl2 (25 mM), 1 µl of each of the two primers (10 pmol/µl), 1 µl of 10 mM nucleotide mix (Promega), and 20.5 µl sterile distilled water. Thermal cycler program used for the amplification consisted of an initial denaturation step at 95 °C for 1 min, followed by 35 cycles of 1 min at 95 °C, 1 min at the annealing temperature and 1 min at 72 °C; with a final extension at 72 °C for 10 min. Annealing temperatures were 49.8 °C for COI, 53 °C for ND5, 55 °C for EF-1α and 58 °C for PER. Amplifications were confirmed by standard gel electrophoresis, using 2% w/v agarose/TBE gels, stained with ethidium bromide. Thirty-five µl of PCR products were purified using QIAquick PCR Purification kit^®^ (QIAGEN, Germantown, MD, USA) following the manufacturer protocol and were sequenced by Eurofins Genomics (Ebersberg, Germany). Sequences were considered for species identification purposes using nBLAST^®^ (Altschul et al., 1990) to confirm the previous morphological identification. A total of 309 sequences were analysed, from them 257 were sequenced in this work (Table 4), and 52 were downloaded from GenBank (Table 5). Analyses based on the phylogenetic relationships between the studied species were carried out to confirm the identification. It is worth mentioning that in order to obtain consistent blocks of nucleotides for all the species, the sequences were processed with Gblock and manually checked (Talavera & Castresana, 2007; Castresana, 2000). Subsequently, sequences were aligned using Clustal Omega (Sievers et al., 2011) and concatenated with FASconCAT v1.0 (Kück & Meusemann, 2010). Trees were built using the Neighbour Joining and the Maximum Likelihood methods on MEGA 7.0 (Kumar, Stecher & Tamura, 2016) using Kimura 2-parameter (K2P) evolutionary model (Čandek & Kuntner, 2015). A bootstrap of 1,000 replicates was used for the phylogenetic reconstructions. Trees were visualised with ITOL (Letunic & Bork, 2016). In the trees reconstruction Piophilidae and Muscidae species were considered as outgroups.

**Table 1:** List of species analysed, with the number of samples, stage of development (A, adult; P, pupae; III L, third larval instar) and country of origin (B, Belgium; UK, United Kingdom; I, Italy).

Species	Nr. of samples	Stage of development	Country of origin
*Calliphora vicina* Robineau-Desvoidy, 1830	28	A	B, UK, I
*Calliphora vomitoria* (Linnaeus 1758)	10	A	UK, I
*Lucilia sericata* (Linnaeus 1758)	22	A	B, UK, I
*Lucilia illustris* (Meigen 1826)	4	A	I
*Lucilia caesar* (Meigen 1826)	3	A	I
*Chrysomya albiceps* (Wiedemann 1819)	3	A, III L	I
*Phormia regina* (Meigen 1826)	1	P	I
*Cynomya mortuorum* (Linnaeus 1761)	1	A	B
*Sarcophaga africa* (Wiedemann, 1824)	1	A	I
*Sarcophaga* sp*.* Meigen 1826	1	III L	UK
*Hydrotaea* sp*.* Robineau-Desvoidy 1830	2	P	UK
*Fannia scalaris* (Fabricius 1794)	2	III L	I
*Piophila* sp*.* Fallen 1810	1	P	UK
*Megaselia scalaris* (Loew 1866)	1	A	I

**Table 2:** Taxonomical keys used for morphological identification of the specimens.

Family	Identification key
Calliphoridae	Rognes (1991), Szpila (2010)
Sarcophagidae	Pape (1996)
Muscidae	Skidmore (1985)
Fanniidae	Skidmore (1985)
Phoridae	McAlpine (1987)
Piophilidae	McAlpine (1987)

**Table 3:** List of primers used in this study.

Gene	Primer name and sequence	Reference
COI	LCO1490 5′- GGTCAACAAATCATAAAGATATTGG -3′	Folmer et al. (1994)
	HC02198 5′- TAAACTTCAGGGTGACCAAAAAATCA -3′	
ND5	ND5(a) 5′- CCAAAATATTCWGATCAHCCYTG -3′	Zehner et al. (2004)
	ND5(b) 5′- GGATTAACTGTTTGTTATWCTTTTCG -3′	
EF-1α	B1 5′- CCCATYTCCGGHTGGCACGG -3′	McDonagh, García & Stevens (2009)
	C1 5′- GTCTCATGTCACGDACRGCG -3′	
PER	PERFW 5′- CTR GAR YTR CCC AAT GAA -3′	This paper
	PERRV 5′- TSR CCC TCC CAH GAA TG -3′	

**Table 4:** New sequences with geographical origin and GenBank code listed by gene.

Morphological identification	Sequence ID	Geographical origin	Genbank code			
			COI	ND5	EF1a	PER
*Calliphora vicina*	2BGCvi	Belgium - B	MH401768	MH401920	MH401958	MH401876
	ITMA8Cvi	Italy - I	MH401769	MH588583		
	ITMO3Cvi	Italy - I	MH401773	MH401924	MH401961	MH401879
	1ITCvi	Italy - I	MH401777	MH588588		
	2ITCvi	Italy - I		MH588592	MH588602	MH588607
	3ITCvi	Italy - I	MH401780	MH588589	MH588601	
	4ITCvi	Italy - I	MH401782	MH401915	MH401963	MH401866
	5ITCvi	Italy - I	MH401784	MH401916	MH401965	MH401863
	6ITCvi	Italy - I	MH401786	MH401917	MH401966	MH401869
	7ITCvi	Italy - I	MH401788	MH588584	MH588603	
	8ITCvi	Italy - I	MH401790	MH588593		
	9ITCvi	Italy - I	MH401792	MH401918	MH401967	MH401873
	10ITCvi	Italy - I	MH401776	MH588585	MH588604	
	ITMACvi1	Italy - I	MH401803	MH588590		
	ITMACvi2	Italy - I	MH401804	MH588591		
	ITMOCvi3	Italy - I	MH401805	MH401904	MH401946	MH401861
	ITMOCvi4	Italy - I	MH401806	MH401902	MH401944	MH401859
	ITMOII1Cvi	Italy - I	MH401807	MH401884	MH401928	MH401839
	ITMOII2Cvi	Italy - I	MH401809	MH401885	MH401929	MH401840
	ITMOII3Cvi	Italy - I	MH401834	MH401886	MH401930	MH401841
	ITMOII4Cvi	Italy - I	MH401810	MH401887	MH401931	MH401842
	ITMOII5Cvi	Italy - I	MH401811	MH401888	MH401932	MH401843
	ITTVCvi1	Italy - I	MH401818	MH588582		MH588605
	ITTVCvi2	Italy - I	MH401819	MH588586		
	ITTVCvi3	Italy - I	MH401820	MH401901	MH401943	MH401858
	BOX4UKPrt	United Kingdom - UK	MH401798	MH588587		
*Calliphora vomitoria*	CvoUK	United Kingdom - UK	MH401764	MH401923	MH401960	MH401878
	ITMO1Cvo	Italy - I	MH401767	MH401925	MH401964	MH401881
	ITMA1Cvo	Italy - I	MH401775	MH588580		
	BOX3UKCvo	United Kingdom - UK	MH401795	MH401898		MH401855
	ITTVCvo1	Italy - I	MH401821	MH401899	MH401969	MH401856
	ITTVCvo2	Italy - I	MH401822	MH588579		
	ITTVCvo3	Italy - I	MH401823	MH401900	MH401942	MH401857
	UKCvo	United Kingdom - UK	MH401832	MH401893	MH401938	MH401849
	70UKCvo	United Kingdom - UK		MH588581	MH588599	MH588606
	99UKCvo	United Kingdom - UK		MH588578	MH588600	MH401882
*Chrysomya albiceps*	ITVVChalbA	Italy - I	MH401800	MH588568	MH588596	
	ITVVChalbL	Italy - I	MH401801	MH588567		
	ITChalb	Italy - I	MH401833	MH588569	MH605069	
*Cynomya mortuorum*	3BGCym	Belgium - B	MH401763	MH401921	MH401959	MH401877
*Fannia* sp.	FanniaL	Italy - I	MH401835	MH588563		
	FanniaP	Italy - I	MH401836	MH588564		
*Hydrotaea* sp.	BOX4UKH	United Kingdom - UK		MH588560	MH588595	
	BOX6UKH	United Kingdom - UK	MH401799	MH588559		
*Lucilia caesar*	ITTVLc1	Italy - I	MH401824	MH401926	MH401941	MH401853
	ITTVLc2	Italy - I	MH401825	MH588572	MH401968	MH401854
	ITTVLc3	Italy - I	MH401826	MH588573		MH605070
*Lucilia illustris*	ITMO2Li	Italy - I	MH401766	MH401922	MH401962	MH401880
	ITNOai15Li	Italy - I	MH401771	MH588571		
	ITMOLi1	Italy - I	MH401816	MH588570		MH588608
	ITTVLill1	Italy - I	MH401827	MH588574		
*Lucilia sericata*	ITMA3Lse	Italy - I	MH401765	MH588575	MH588598	
	1BGLs	Belgium - B	MH401772	MH401919	MH401957	MH401875
	ITMO1Ls	Italy - I	MH401774	MH588577		
	1ITLs	Italy - I	MH401778	MH401906	MH401948	MH401864
	2ITLs	Italy - I	MH401779	MH401907	MH401949	MH401862
	3ITLs	Italy - I	MH401781	MH401908	MH401950	MH401865
	4ITLs	Italy - I	MH401783	MH401909	MH401951	MH401867
	5ITLs	Italy - I	MH401785	MH401910	MH401952	MH401868
	6ITLs	Italy - I	MH401787	MH401911	MH401953	MH401870
	7ITLs	Italy - I	MH401789	MH401912	MH401954	MH401871
	8ITLs	Italy - I	MH401791	MH401913	MH401955	MH401872
	9ITLs	Italy - I	MH401793	MH401914	MH401956	MH401874
	BOX3UKLs	United Kingdom - UK		MH588576	MH588597	
	ITVVLs	Italy - I	MH401802	MH401905	MH401947	MH401848
	ITMOII10Ls	Italy - I	MH401808	MH401883	MH401927	MH401838
	ITMOII6Ls	Italy - I	MH401812	MH401889	MH401933	MH401844
	ITMOII7Ls	Italy - I	MH401813	MH401890	MH401934	MH401845
	ITMOII8Ls	Italy - I	MH401814	MH401891	MH401935	MH401846
	ITMOII9Ls	Italy - I	MH401815	MH401892	MH401936	MH401847
	ITTVLs1	Italy - I	MH401828	MH401894	MH401937	MH401850
	ITTVLs2	Italy - I	MH401829	MH401895	MH401939	MH401851
	ITTVLs3	Italy - I	MH401830	MH401896	MH401940	MH401852
*Megaselia scalaris*	Ms	Italy - I		MH588562		MH401837
*Phormia regina*	ITQC2Phr	Italy - I	MH401770	MH588566		
*Piophila* sp.	UKPio	United Kingdom - UK	MH401817	MH588561	MH588594	
*Sarcophaga africa*	ITTVSA	Italy - I	MH401831	MH401903	MH401945	MH401860
*Sarcophaga* sp.	BOX1UKSL	United Kingdom	MH401794	MH588565		

**Table 5:** Sequences selected from GenBank listed by gene. Country of origin and its abbreviation used in the cladograms are reported.

**Gene**	**Species**	**GenBank #**	**Country**	**Abbreviation**
**COI**	*C.vicina*	JX438024	Portugal	P
		KC617807	USA	USA
	*C.vomitoria*	JX438025	Portugal	P
		KC775967	Portugal	P
	*C. albiceps*	JX438026	Portugal	P
		HE814059	Germany	D
	*P. regina*	KM569886	Canada	CDN
		KM569803	Canada	CDN
	*P. terraenovae*	KM570349	Canada	CDN
		KF908116	Belgium	B
	*L. sericata*	JX438041	Portugal	P
		KC776060	Portugal	P
	*L. illustris*	KM571189	Canada	CDN
		KM570007	Canada	CDN
	*L. caesar*	KJ635700	Spain	E
		KJ635701	Spain	E
	*S. africa*	JQ413455	Kenya	EAK
	*S. melanura*	JQ413457	Belgium	B
	*H. dentipes*	HM891630	Sweden	S
	*F. canicularis*	JX438029	Portugal	P
		KC617819	USA	USA
	*M. scalaris*	KC407774	Korea	ROK
		JQ941746	China	RC
**ND5**	*C.vicina*	NC_019639	France	F
		JX_913760	France	F
	*C. albiceps*	NC_019631	Zambia	Z
	*P. regina*	NC_026668	USA	USA
	*P. terraenovae*	NC_019636	France	F
	*L. sericata*	FJ614877	China	RC
		FJ614876	China	RC
	*L. illustris*	KM571189	China	RC
		KM570007	China	RC
	*S. africa*	NC_025944	China	RC
	*M. scalaris*	NC_023794	China	RC
**PER**	*L. sericata*	JN792856	USA	USA
		JN792853	South Africa	ZA
	*L. illustris*	KF839550	USA	USA
		KF839549	USA	USA
	*L. caesar*	KF839532	France	F
		JN792858	France	F
	*S. africa*	KC966442	China	RC
		KC966441	China	RC
	*M. scalaris*	KC178059	USA	USA
**EF 1 alfa**	*C.vomitoria*	JQ307782	United Kingdom	UK
		FJ025666	Singapore	SGP
	*P. terraenovae*	JQ307784	United Kingdom	UK
	*L. sericata*	JQ307785	United Kingdom	UK
	*L. illustris*	JQ307786	United Kingdom	UK
	*L. caesar*	JQ307787	United Kingdom	UK
		JQ307787	United Kingdom	UK
	*H. dentipes*	FJ025679	China	RC
	*F. canicularis*	AJ871202	Canada	CDN

## Results

**Figure 1:**
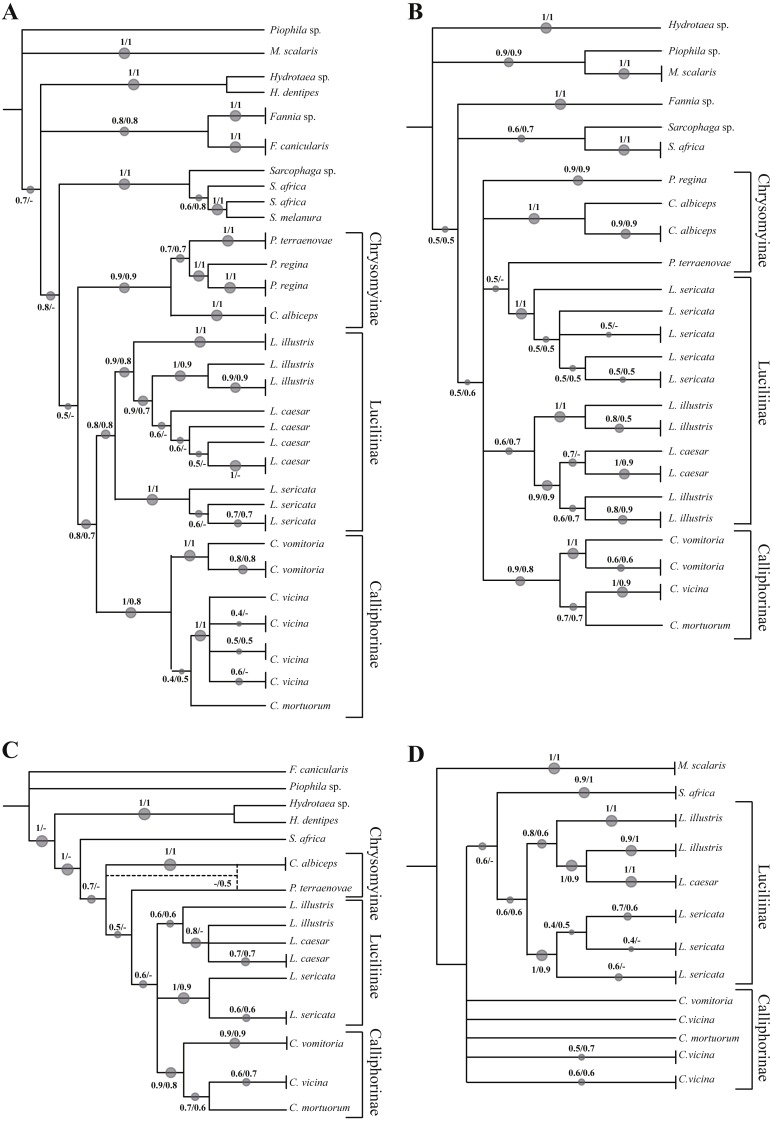
Simplified phylogenetic trees. Simplified representation of the phylogenetic trees obtained using COI (A), ND5 (B), EF-1α (C) and PER (D) genes. Original trees are reported in the Supplemental Information.

The analysed specimens belonged to fourteen species, with *Calliphora vicina* Robineau-Desvoidy, 1830 and *Lucilia sericata* (Meigen, 1826) (Diptera, Calliphoridae) as the most abundant taxa representing 29.8 and 27.4% respectively. The first analysis step was based on a local alignment using GenBank BLAST (Altschul et al., 1990) and the percentage of correct identification was evaluated. In particular, the molecular one-gene identification was compared with the morphological identification obtaining a percentage value match of 87.5% for COI, 72.5% for ND5, 77.1% for EF-1α and 67.9% for PER. Concerning Calliphoridae, the percentages were 77.5, 64.1, 71.2 and 64.2% respectively. A phylogenetic approach was used to verify the molecular identification efficiency, however, the sequencing of EF-1α and PER regions was successful only in 72.6% and 69.1% of the specimens respectively. Independent analysis of COI (Fig. 1A, Fig. S1) recovered the monophyly of all families. All the subfamilies (Calliphorinae, Luciliinae and Chrysomynae) are separated with robust bootstrap values ranging from 0.8 to 1 in a scale between 0 and 1. Among the genus *Lucilia*, *L. sericata* sequences cluster together and are clearly distinct from the other co-generic species (bootstrap 1), while the pattern of *Lucilia illustris* Meigen, 1826 and *Lucilia Caesar* (Linnaeus, 1758) is not clearly resolved with *L. illustris* showing a paraphyletic pattern. The genus *Calliphora* Robineau-Desvoidy, 1830 was also recovered as paraphyletic, in this case *C. vicina* branches with *Cynomya mortuorum* (Linnaeus, 1761), but with a weak support, instead of branching with *C. vomitoria*.

Phylogenetic reconstruction based on the ND5 marker (Fig. 1B, Fig. S2) shows an unresolved topology with problems of determination at all taxonomic levels (family, subfamily, genus and species). *Lucilia caesar* and *L. illustris* are not clearly distinct and in addition *Protophormia terraenovae* Robineau-Desvoidy, 1830 sequence clusters with *L. sericata* sequences. A small number of sequences was available for both EF-1α (Fig. 1C, Fig. S3) and PER gene (Fig. 1D, Fig. S4). Both phylogenetic reconstructions obtained using these markers showed the same problems reported for COI and ND5, with *L. illustris* and *L. caesar* not clearly distinct.

**Figure 2:**
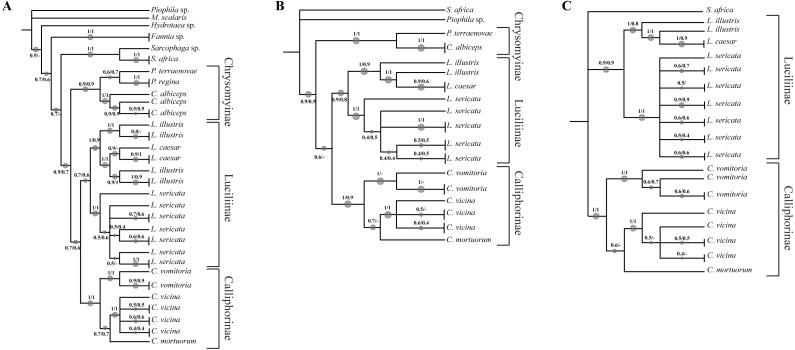
Simplified phylogenetic trees. Simplified representations of the phylogenetic threes obtained using COI and ND5 (A), COI, ND5 and EF-1α (B) and COI, ND5, EF-1α and PER (C) genes. Original trees are reported in the Supplemental Information.

In order to increase the molecular information three concatenated sequences were generated using the previous genes (DeSalle, Egan & Siddall, 2005). The phylogenetic reconstruction based on the chimeric sequence generated on the two mitochondrial genes (COI and ND5) (Fig. 2A, Fig. S5) does not provide a better resolution for *L. illustris/L. caesar* species as well as for the position of *C. mortuorum* among the Calliphorinae. These two points are not better clarified when the nuclear sequences are included and two more chimeric sequences with three (COI, ND5 and EF-1α) (Fig. 2B, Fig. S6) and four (COI, ND5, EF-1α and PER) (Fig. 2C, Fig. S7) genes are generated. Table 6 summarizes the information of the sequences used in the phylogenetic reconstructions.

## Discussion

The results obtained with a local alignment demonstrate that the match of the molecular identification with the morphological identification of the specimens was never higher than 90% also considering COI gene (87.5%), currently used for species identification (DNA Barcoding Project (http://www.barcodeoflife.org/)). The analysis of ND5 gene, a mitochondrial gene, was difficult for *Calliphora vomitoria* (Linnaeus, 1758) due to a complete lack of ND5 sequences from this species in the database (GenBank) at the moment of the analysis. The molecular analysis of the closely related though morphologically well distinct species, *L. illustris* and *L. caesar,* does not allow a unambiguous identification of them, as already reported in previous works where different phylogenetic approaches (e.g., Maximum Parsimony) were also used (GilArriortua et al., 2015; Wells, Wall & Stevens, 2007). In fact, GilArriortua and co-workers (GilArriortua et al., 2015) indicated that *L*. *caesar* and *L. illustris* species appear to share mitochondrial genomes with a divergence value lower than the minimum inter-specific threshold value for mitochondrial loci. ND5 gene showed the same problem in the discrimination of the close *Lucilia* species. To our knowledge, the analysis of closely related species in blowflies using ND5 gene was only reported by Zaidi and co-workers who showed a good identification performance using this gene (Zaidi et al., 2011). In addition, the same mitochondrial region was used to analyse the evolutionary relationship between flesh flies with a good resolution (Zehner et al., 2004). The analysis of EF-1α gene is in agreement with a previous study (McDonagh & Stevens, 2011) that demonstrated a good ability of this gene to separate blowflies according to morphological classification. However, in our reconstruction both the position of the *Lucilia* species and *Cynomya*, within Calliphorinae, are not well resolved. To our knowledge, PER gene was studied for identification purposes only in flesh flies (Guo et al., 2014). This work showed the possibility to use successfully PER gene for identification purposes, although public datasets might be enriched with further DNA sequences belonging to different family of Diptera.

**Table 6:** Size (bp) and number of sequences analysed.

	Gene				Concatenated sequences		
	**COI**	**ND5**	**EF-1α**	**PER**	**COI+ND5**	**COI+ND5+EF-1α**	**COI+ND5+EF-1α+PER**
Length (bp)	605	329	309	327	934	1,243	1,570
Nr. of sequences	72 + 23^*^	78 + 11^*^	55 + 9^*^	52 + 9^*^	72 + 10^*^	50 + 3^*^	43 + 2^*^

**Notes.**

* indicates the sequence from GenBank.

The analysis of the concatenated sequences generated with COI, ND5, EF-1α and PER markers unfortunately does not improve the resolution of the investigation despite previous works indicate that the combination of nuclear and mitochondrial genes for species identification is a much more accurate approach. In fact the combination of markers that have different evolutionary histories, fast and slow evolving genes, allows a better resolution of the phylogenies. In particular, the multi-loci analysis of COI, EF-1α, and 28S rRNA genes and the combined analysis of COI, CYTB, ND5, and ITS1 and ITS2 genes has demonstrated to be more successful compared to the single-locus phylogeny, leading to a better grouping of species belonging to the same family (Zaidi et al., 2011; McDonagh & Stevens, 2011; Grzywacz, Wallman & Piwczyński, 2017). However, as underlined by Sonet et al. (2012), not always the addition of more genes with different evolutionary histories resolves the monophyly of closely related species such as *L. illustris* and *L. caesar*. The monophyly of these two species was clearly demonstrated only by two research groups: one working with the gene Bicoid (Park et al., 2013) and another one using the AFLP (Amplified Fragment Length Polymorphism) approach (Picard et al., 2018). In both cases the two species were well resolved with a strong basal support, confirming the conclusions obtained from the morphological analysis of male and female specimens of both species.

At the moment, because of the small number of sequences available for these two species, we cannot exclude phenomena of hybridization at least in some parts of the distribution area of the species, but this point needs further investigations and a larger dataset to be analysed.

The importance to have complete and correct dataset is a crucial point to reach a correct species identification, with both local alignment systems and/or phylogenetic methods. Molecular approach is strongly related to the quality of information stored in databases, and the possibility to improve the amount of genetic markers from different specimens from different geographical locations is important to recover the best resolution in phylogenetic trees. The availability of genetic data from different populations allows to have information about the intraspecific variability that, in closely related species can affect the phylogenetic reconstruction. The use of a single gene approach to identify animal species is an open argument, especially for closely related species. In particular, mtDNA does not seem to be significantly different from any other marker group revealing an overall success rate of 71% (Dupuis, Roe & Sperling, 2012). In fact, the mitochondrial evolution reduces its applicability for detailed systematic or taxonomic analysis for closely related species (Dupuis, Roe & Sperling, 2012; Will, Mishler & Wheeler, 2005; De Carvalho et al., 2008). Dupuis and co-workers (Dupuis, Roe & Sperling, 2012) highlighted two main results: (i) marker classes (mtDNA, ribosomal DNA, autosomal loci, sex-linked loci, and anonymous loci) were moderately successful to delimit closely related species, if used as unique identifier, and (ii) multi-locus power analysis data support investigation and use of multiple markers for species delimitation. Several papers have discussed multi-locus analysis as species identification methods for animal kingdom. In particular, sex-linked markers showed a high success ratio in delimiting closely related species in Diptera and Lepidoptera (Coyne & Orr, 1989; Roe & Sperling, 2007). The improvement of genetic datasets and the concatenation of different mitochondrial and nuclear loci could improve the capability of molecular approach to identify closely related species but this aspect has to be further explored considering as well the taxon’s specificity.

It is worth mentioning as well that in this kind of studies the species choice and intra-specific sampling scheme can strongly affect the level of resolution of the analysis. In our study, a further investigation including a larger sequence dataset of species in the genus *Lucilia* from different geographical contexts would better clarify the results here reported and the derived conclusions.

## Conclusions

Nowadays, in forensic entomology, the morphological identification approach for some species is not completely replaceable by the molecular one if based on a single gene. The two methodologies can complement each other. In addition, because of the lack of information in databases, a phylogenetic approach can increase the ability of species identification when the molecular approach is used. The analysis of mitochondrial genes is considered the best approach because of the peculiarity of this kind of DNA, in terms of haploidy, high copy numbers, low recombination and lack of introns (Hebert et al., 2003). However, considering the nature of mitochondrial evolution and the results of this and previous studies, the use of mtDNA does not provide a good level of resolution for some of the *Lucilia* species. In addition, the analysis of nuclear genes, such as EF-1α and PER, cannot improve this point. Additional work using mtDNA in association with other genetic markers (i.e., sex-linked loci) could clarify and resolve the relationships among the *Lucilia* genus as well as other close related species. It is worth mentioning that the investigation for the best marker has to be done at the genus level, in fact some markers that have been suggested in addition to COI (e.g., ITS2) work for the resolution of certain taxa but not for others. In addition, given the problems in the resolution of several genera/species in the family Calliphoridae as highlighted as well in this paper, an approach based on NGS technologies (e.g., WGS –whole genome shotgun) will probably provide enough information to distinguish the taxa.

##  Supplemental Information

10.7717/peerj.5962/supp-1 Figure S1 Phylogenetic tree based on Neighbour Joining and Maximum Likelihood analysis of 605 bp sequence of the COI gene Numbers indicate the bootstrap value in the range 0–1. The size of the spots is directly proportional to the bootstrap value. * indicates the sequence from GenBank

10.7717/peerj.5962/supp-2 Figure S2 Phylogenetic tree based on Neighbour Joining and Maximum Likelihood analysis of 329 bp sequence of the ND5 gene Numbers indicate the bootstrap value in the range 0–1. The size of the spots is directly proportional to the bootstrap value. * indicates the sequence from GenBank

10.7717/peerj.5962/supp-3 Figure S3 Phylogenetic tree based on Neighbour Joining and Maximum Likelihood analysis of 309 bp sequence of the EF-1α gene Numbers indicate the bootstrap value in the range 0–1. The size of the spots is directly proportional to the bootstrap value. * indicates the sequence from GenBank

10.7717/peerj.5962/supp-4 Figure S4 Phylogenetic tree based on Neighbour Joining and Maximum Likelihood analysis of 327 bp sequence of the PER gene Numbers indicate the bootstrap value in the range 0-1. The size of the spots is directly proportional to the bootstrap value. * indicates the sequence from GenBank

10.7717/peerj.5962/supp-5 Figure S5 Phylogenetic tree based on Neighbour Joining and Maximum Likelihood analysis of 934 bp of the COI+ND5 concatenated sequences Numbers indicate the bootstrap value in the range 0–1. The size of the spots is directly proportional to the bootstrap value. * indicates the sequence from GenBank

10.7717/peerj.5962/supp-6 Figure S6 Phylogenetic tree based on Neighbour Joining and Maximum Likelihood analysis of 1,243 bp of the COI+ND5+EF-1α concatenated sequences Numbers indicate the bootstrap value in the range 0–1. The size of the spots is directly proportional to the bootstrap value. * indicates the sequence from GenBank

10.7717/peerj.5962/supp-7 Figure S7 Phylogenetic tree based on Neighbour Joining and Maximum Likelihood analysis of 1570 bp of the COI+ND5+EF-1α+PER concatenated sequences Numbers indicate the bootstrap value in the range 0–1. The size of the spots is directly proportional to the bootstrap value. * indicates the sequence from GenBank

10.7717/peerj.5962/supp-8 Supplemental Information 1 Sequences used to create all the phylogenetic trees

## Additional Information and Declarations

### Data Availability

The following information was supplied regarding data availability: The following information was supplied regarding data availability: The downloaded GenBank sequences: JX438024, KC617807, JX438025, KC775967, JX438026, HE814059, KM569886, KM569803, KM570349, KF908116, JX438041, KC776060, KM571189, KM570007, KJ635700, KJ635701, JQ413455, JQ413457, HM891630, JX438029, KC617819, KC407774, JQ941746, NC_019639, JX_913760, NC_019631, NC_026668, NC_019636, FJ614877, FJ614876, KM571189, KM570007, NC_025944, NC_023794, JN792856, JN792853, KF839550, KF839549, KF839532, JN792858, KC966442, KC966441, KC178059, JQ307782, FJ025666, JQ307784, JQ307785, JQ307786, JQ307787, JQ307787, FJ025679, AJ871202
The sequences used to create all the phylogenetic trees are available in the Supplemental File and at Genbank: MH401763–MH401969 and MH588559–MH588609.
