# Supplementary figures and images for: Do longer sequences improve the accuracy of identification of forensically important Calliphoridae species?
Source: PeerJ. 2018 Dec 17;6:e5962. doi: 10.7717/peerj.5962 (PMC6301277; doi:10.7717/peerj.5962)

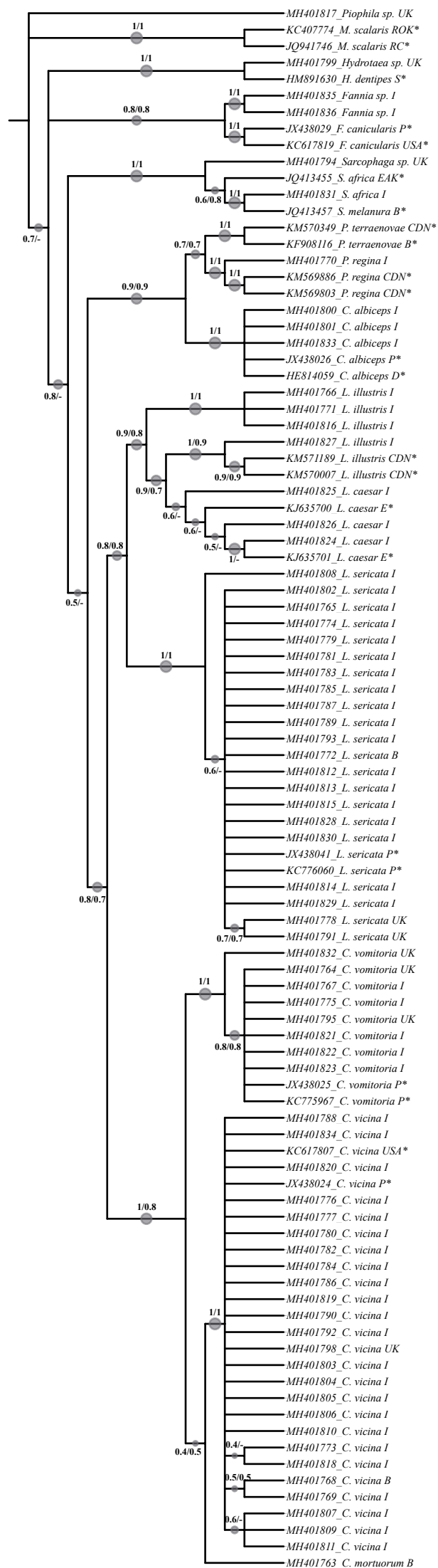

Supplement: Figure S1 — Numbers indicate the bootstrap value in the range 0–1. The size of the spots is directly proportional to the bootstrap value. * indicates the sequence from GenBank [file peerj-06-5962-s001.pdf]

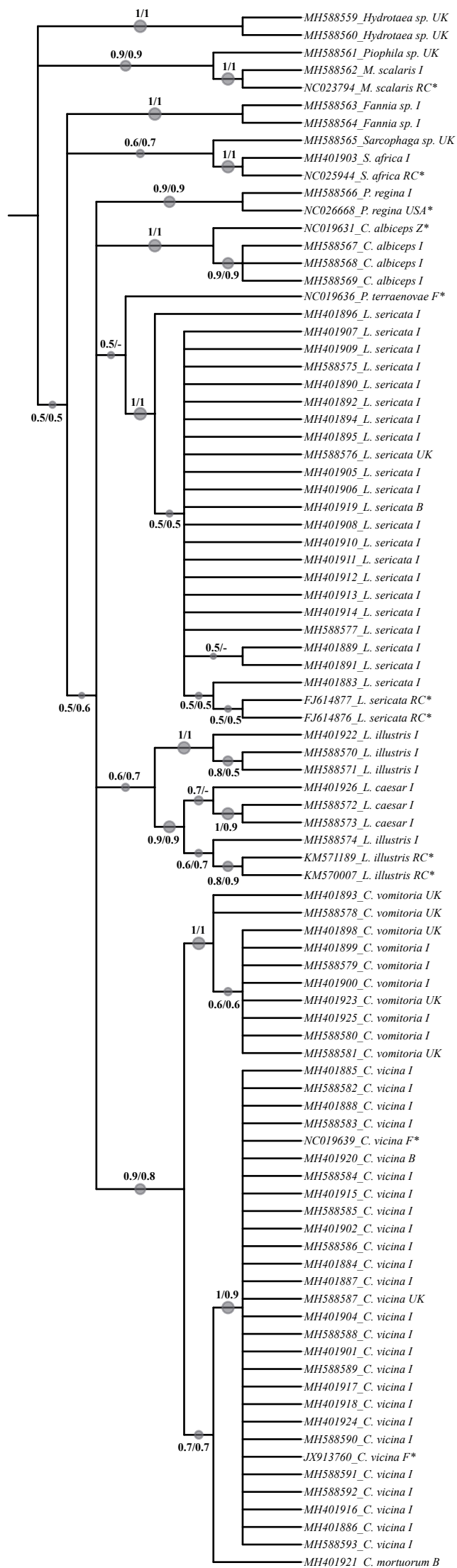

Supplement: Figure S2 — Numbers indicate the bootstrap value in the range 0–1. The size of the spots is directly proportional to the bootstrap value. * indicates the sequence from GenBank [file peerj-06-5962-s002.pdf]

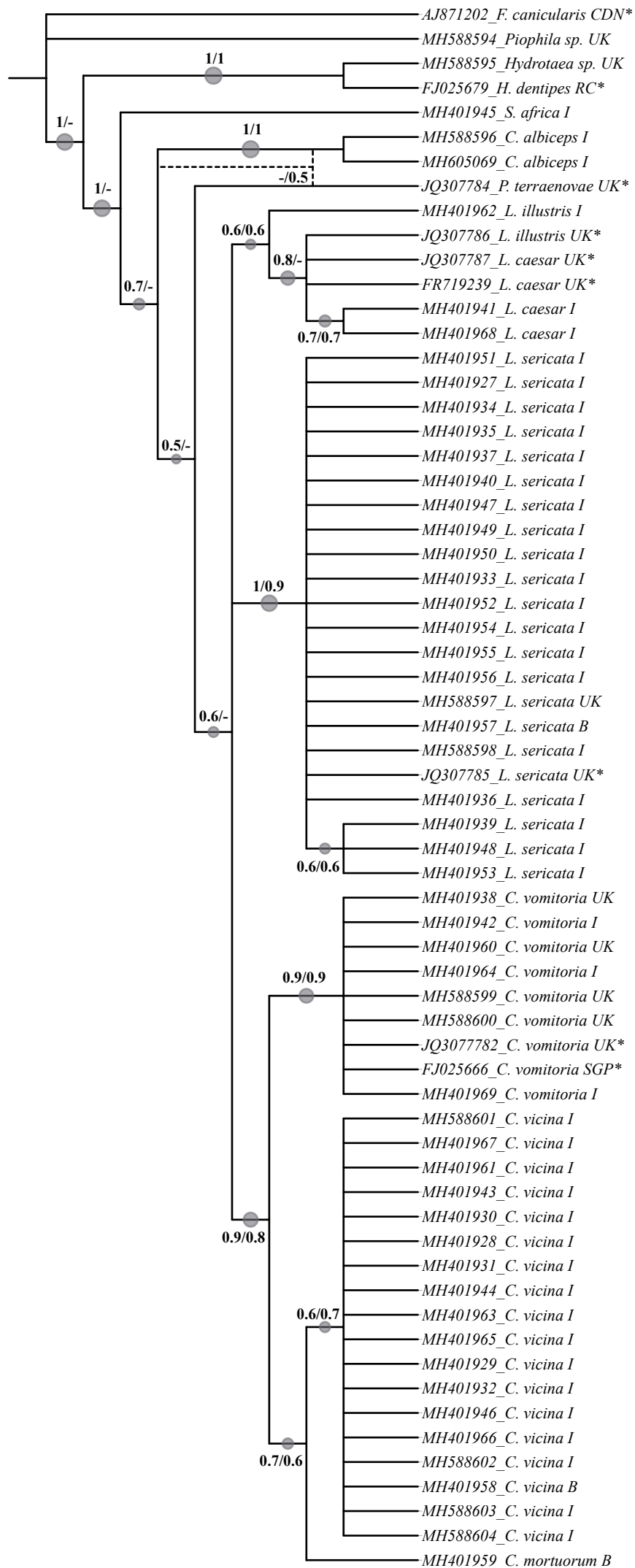

Supplement: Figure S3 — Numbers indicate the bootstrap value in the range 0–1. The size of the spots is directly proportional to the bootstrap value. * indicates the sequence from GenBank [file peerj-06-5962-s003.pdf]

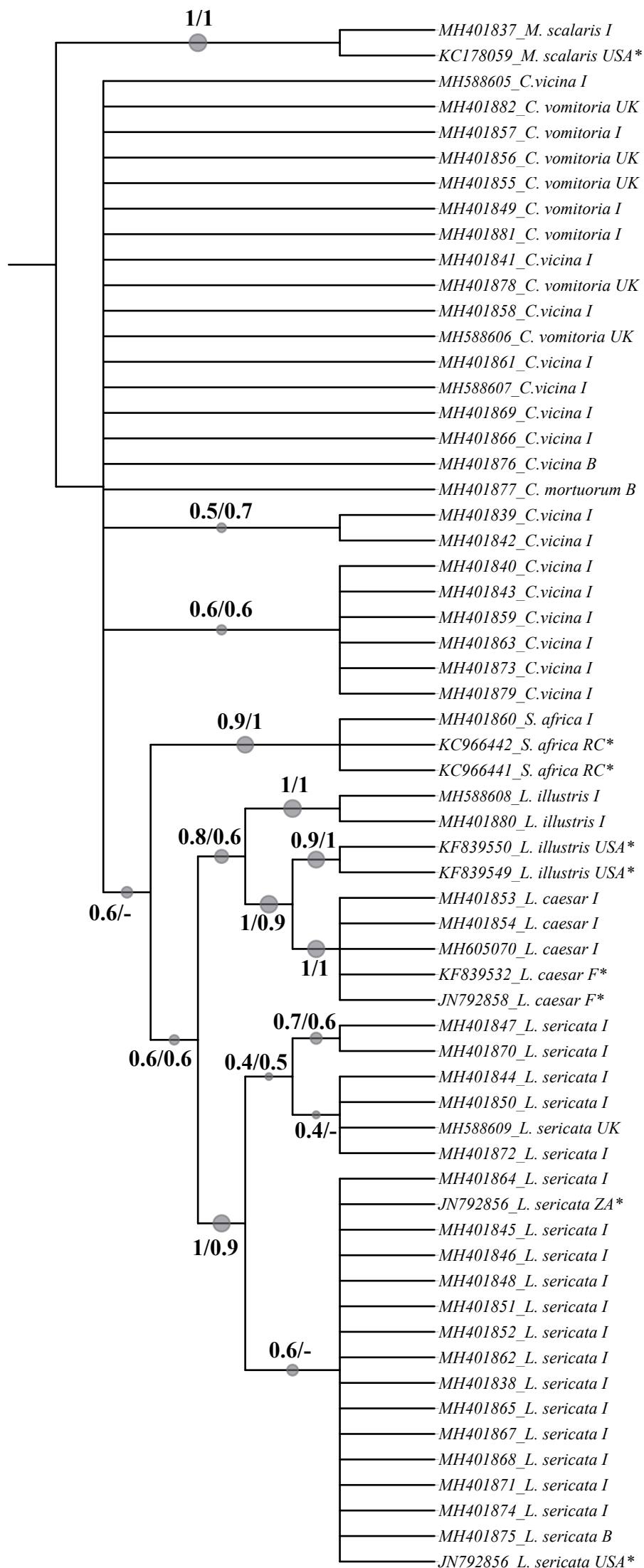

Supplement: Figure S4 — Numbers indicate the bootstrap value in the range 0-1. The size of the spots is directly proportional to the bootstrap value. * indicates the sequence from GenBank [file peerj-06-5962-s004.pdf]

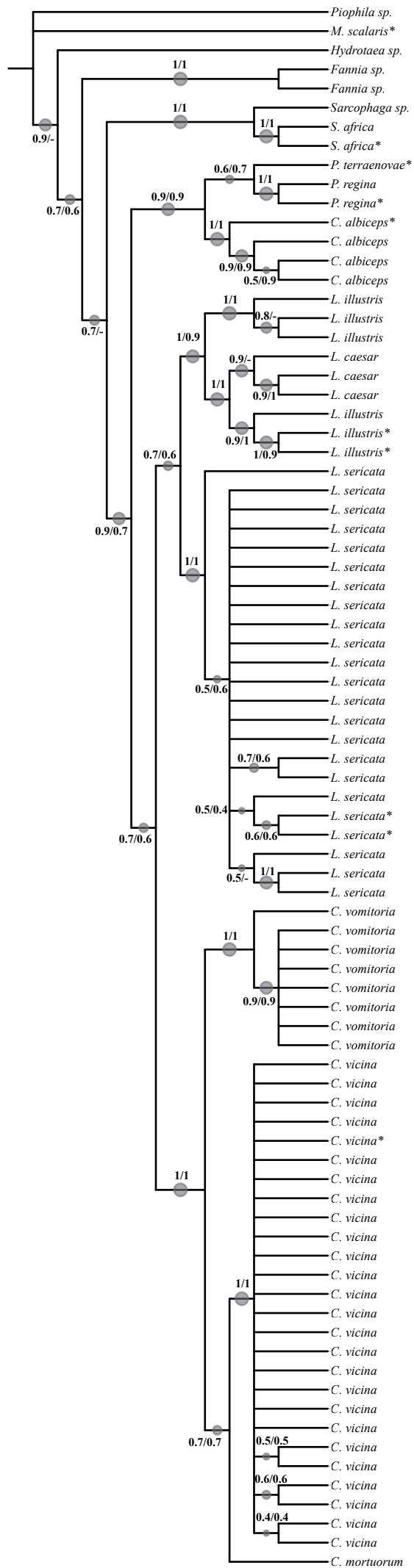

Supplement: Figure S5 — Numbers indicate the bootstrap value in the range 0–1. The size of the spots is directly proportional to the bootstrap value. * indicates the sequence from GenBank [file peerj-06-5962-s005.pdf]

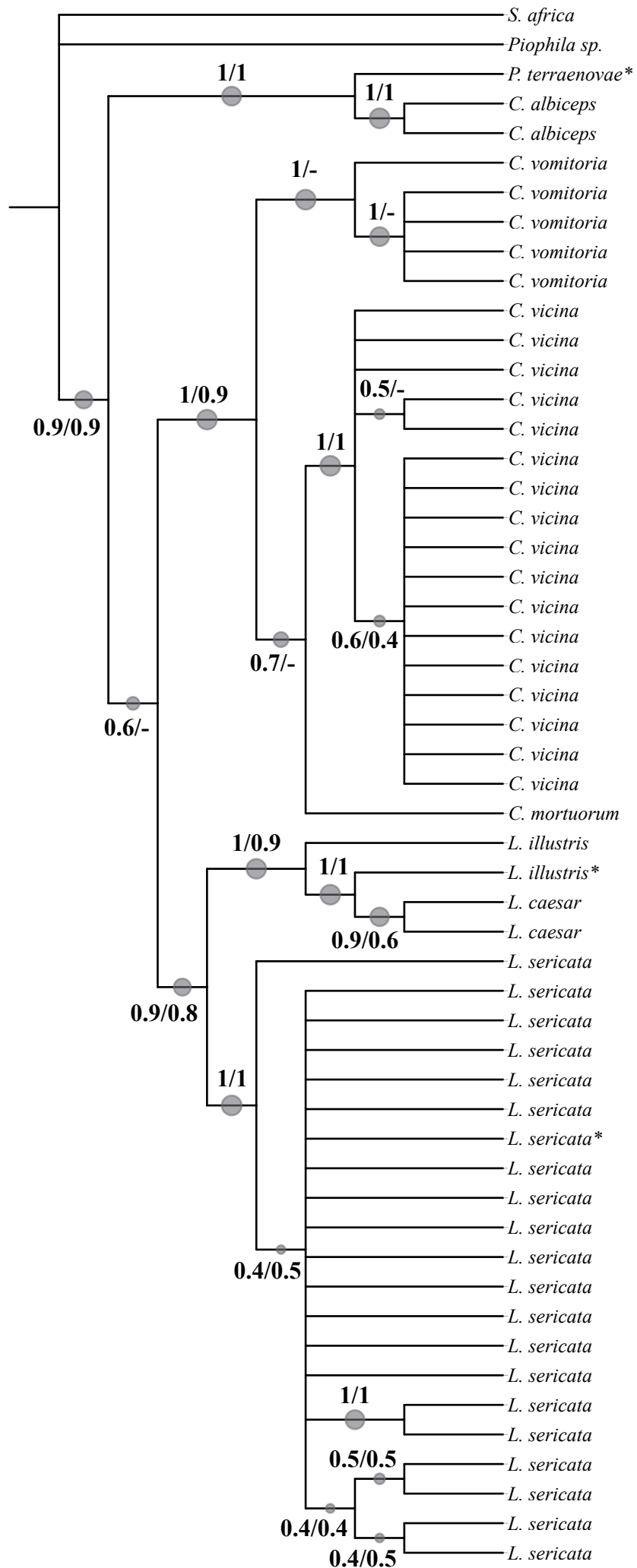

Supplement: Figure S6 — Numbers indicate the bootstrap value in the range 0–1. The size of the spots is directly proportional to the bootstrap value. * indicates the sequence from GenBank [file peerj-06-5962-s006.pdf]

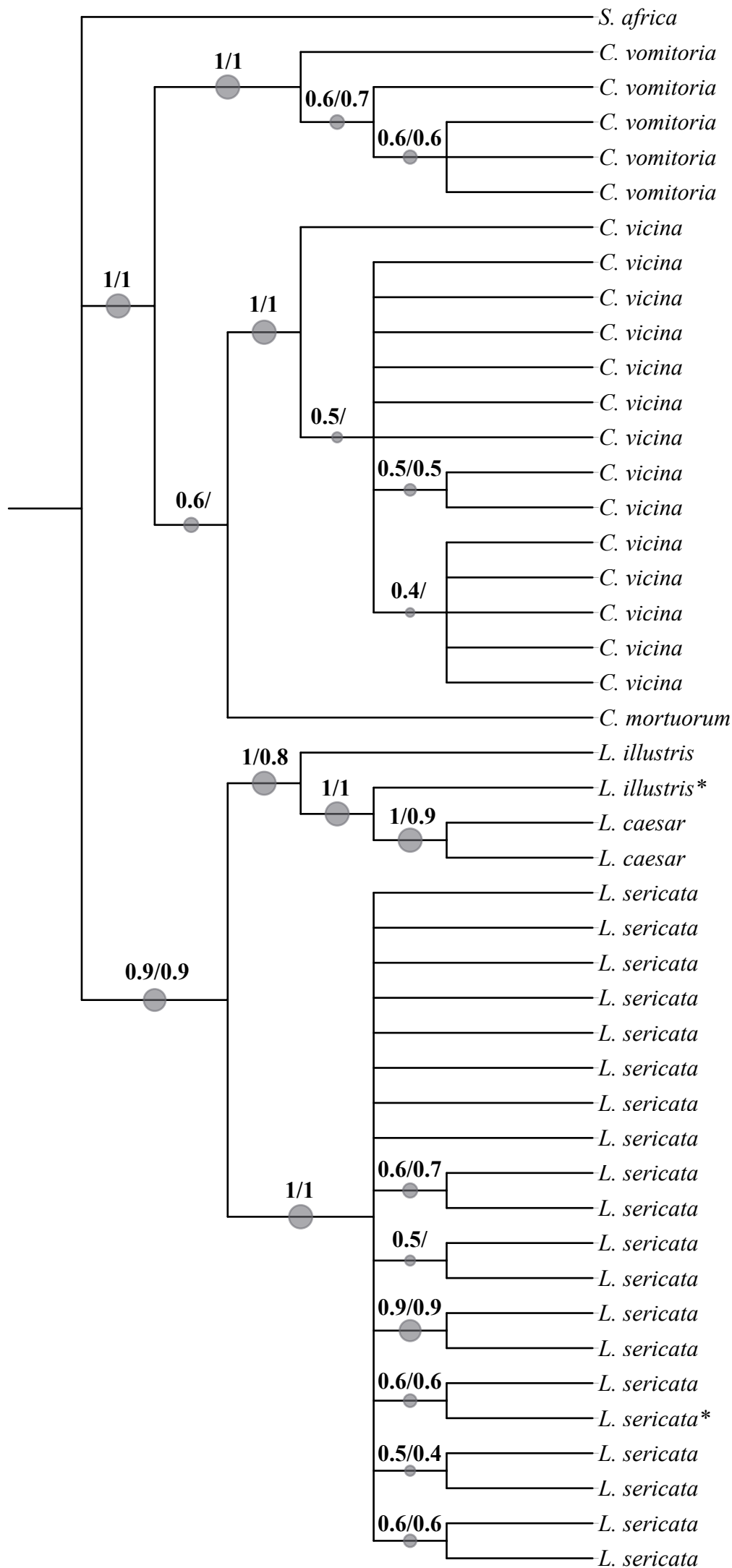

Supplement: Figure S7 — Numbers indicate the bootstrap value in the range 0–1. The size of the spots is directly proportional to the bootstrap value. * indicates the sequence from GenBank [file peerj-06-5962-s007.pdf]
