# Supplementary material for: Do longer sequences improve the accuracy of identification of forensically important Calliphoridae species?
Source: PeerJ. 2018 Dec 17;6:e5962. doi: 10.7717/peerj.5962 (PMC6301277; doi:10.7717/peerj.5962)
Supplement: Supplemental Information 1 [file peerj-06-5962-s008.docx]

**COI sequences**

>MH401763

AGAATTTTAATTCGAGCCGAACTAGGACACCCTGGAGCATTAATTGGTGATGACCAAATCTATAATGTAATTGTTACAGCTCATGCTTTTATTATAATTTTTTTTATAGTAATACCAATTATAATTGGAGGGTTTGGTAATTGATTAGTCCCTTTAATGTTAGGAGCCCCAGATATAGCTTTCCCTCGGATAAACAATATAAGTTTCTGACTTTTACCTCCTGCATTAACTTTACTATTAGTAAGTAGTATAGTGGAAAACGGAGCTGGAACAGGATGAACTGTTTATCCACCTTTATCATCTAATATTGCTCATGGAGGAGCTTCTGTTGATTTAGCTATTTTTTCTTTACATTTAGCGGGAATTTCTTCAATTTTAGGAGCTGTAAATTTTATTACAACAGTTATTAATATACGATCAACAGGAATTACTTTTGACCGAATACCCCTATTCGTTTGATCAGTAGTAATTACAGCTTTATTACTTTTACTATCTTTACCTGTTCTAGCTGGGGCTATTACTATATTATTAACAGACCGAAACCTTAATACTTCATTCTTCGACCCAGCAGGAGGAGGAGACCCAATTCTATACCAACACTTATTTTGATTTTTTGGACAT

>MH401764

AGAATTTTAATTCGAGCTGAACTAGGGCATCCTGGAGCATTAATTGGAGATGATCAAATTTATAATGTAATTGTTACAGCTCATGCTTTTATTATAATTTTTTTTATAGTAATACCAATTATAATTGGAGGGTTTGGAAATTGATTAGTTCCTTTAATATTAGGAGCCCCAGATATAGCATTCCCTCGAATAAATAATATAAGTTTCTGACTTTTACCTCCTGCATTAACTTTACTATTAGTAAGTAGTATAGTAGAAAACGGAGCTGGAACTGGATGAACTGTTTATCCACCTTTATCTTCTAATATTGCACATGGAGGAGCTTCTGTTGATTTAGCTATTTTTTCTTTACATTTAGCAGGAATTTCTTCAATTTTAGGAGCTGTAAATTTTATTACTACAGTTATTAATATACGATCAACAGGTATTACCTTCGACCGAATACCATTATTTGTTTGATCAGTAGTAATTACAGCCTTATTACTTTTATTATCTTTACCAGTATTAGCAGGAGCTATTACTATATTATTAACAGATCGAAATCTTAATACTTCATTCTTTGACCCAGCAGGAGGAGGAGATCCAATTTTATACCAACACTTATTTTGATTCTTTGGA

>MH401765

AGAATTCTAATTCGAGCTGAATTAGGACATCCTGGAGCTTTAATTGGAGATGATCAAATTTATAATGTAATTGTTACAGCTCATGCTTTTATTATAATTTTTTTTATAGTAATGCCAATTATAATTGGAGGATTTGGAAATTGATTAGTTCCATTAATACTAGGAGCTCCAGATATAGCATTCCCTCGAATAAATAATATAAGTTTTTGACTTTTACCTCCTGCATTAACTTTATTATTAGTTAGTAGTATAGTAGAAAACGGAGCTGGAACAGGATGAACAGTTTACCCTCCTCTATCTTCTAATATTGCTCATGGAGGAGCTTCTGTTGATTTAGCTATTTTCTCTCTTCATTTAGCAGGAATTTCTTCAATTTTAGGAGCTGTAAATTTTATTACTACAGTTATTAATATACGATCAACAGGAATTACTTTTGATCGAATACCTTTATTTGTTTGATCAGTAGTAATTACAGCTTTATTACTTTTATTATCATTACCAGTATTAGCAGGAGCTATTACAATACTTTTAACAGACCGAAATCTTAATACATCATTCTTTGACCCTGCAGGAGGAGGAGATCCAATTTTATACCAACATTTATTTTGATTCTTTGGACACCCAGG

>MH401766

AGAATTCTAATTCGAGCTGAATTAGGACACCCTGGTGCACTAATTGGAGATGACCAAATTTATAATGTAATTGTTACAGCTCATGCTTTTATTATAATTTTCTTTATAGTAATACCAATTATAATTGGAGGATTTGGTAATTGATTAGTTCCTTTAATGTTAGGAGCCCCAGATATAGCCTTCCCTCGATTAAATAATATAAGATTTTGACTTTTACCTCCTGCACTAACTTTATTATTAGTAAGTAGTATAGTAGAAAACGGAGCTGGAACAGGATGAACAGTTTACCCCCCTTTATCATCTAATATCGCTCATGGAGGAGCTTCTGTTGATTTAGCTATTTTTTCTCTTCATTTAGCAGGAATTTCTTCAATTTTAGGGGCTGTAAATTTTATTACTACAGTCATTAATATACGATCAACAGGAATTACTTTTGACCGAATACCTTTATTTGTTTGATCAGTTGTAATTACAGCTCTATTACTTTTATTATCATTACCAGTATTAGCAGGAGCTATTACAATACTTTTAACTGACCGAAACCTTAATACTTCATTTTTTGACCCTGCAGGAGGAGGAGACCCAATTCTATACCAACATCTATTTTGATTCTTTGG

>MH401767

AGAATTTTAATTCGAGCTGAACTAGGGCATCCTGGAGCATTAATTGGAGATGATCAAATTTATAATGTAATTGTTACAGCTCATGCTTTTATTATAATTTTTTTTATAGTAATACCAATTATAATTGGAGGGTTTGGAAATTGATTAGTTCCTTTAATATTAGGAGCCCCAGATATAGCATTCCCTCGAATAAATAATATAAGTTTCTGACTTTTACCTCCTGCATTAACTTTACTATTAGTAAGTAGTATAGTAGAAAACGGAGCTGGAACTGGATGAACTGTTTATCCACCTTTATCTTCTAATATTGCACATGGAGGAGCTTCTGTTGATTTAGCTATTTTTTCTTTACATTTAGCAGGAATTTCTTCAATTTTAGGAGCTGTAAATTTTATTACTACAGTTATTAATATACGATCAACAGGTATTACCTTCGACCGAATACCATTATTTGTTTGATCAGTAGTAATTACAGCCTTATTACTTTTATTATCTTTACCAGTATTAGCAGGAGCTATTACTATATTATTAACAGATCGAAATCTTAATACTTCATTCTTTGACCCAGCAGGAGGAGGAGATCCAATTTTATACCAACACTTATTTTGATTCTTTG

>MH401768

AGAATTCTAATTCGAGCCGAACTAGGACATCCTGGAGCATTAATTGGAGATGACCAAATTTATAATGTAATTGTTACAGCTCATGCTTTTATTATAATTTTTTTTATAGTAATACCAATTATAATTGGAGGATTTGGTAATTGATTAGTCCCTTTAATATTAGGAGCTCCAGATATAGCCTTCCCTCGGATAAACAATATAAGTTTCTGACTTTTACCTCCTGCATTAACTTTACTATTAGTAAGTAGTATAGTAGAAAACGGAGCTGGAACAGGATGAACTGTTTACCCACCTTTATCTTCTAATATCGCTCATGGAGGAGCTTCTGTTGATTTAGCTATTTTTTCTTTACACTTAGCAGGAATTTCTTCAATTTTAGGAGCTGTAAATTTTATTACTACAGTTATTAATATACGATCAACAGGAATTACATTCGACCGAATACCATTATTTGTTTGATCTGTAGTAATTACAGCTTTATTACTTTTATTATCTTTACCAGTATTAGCAGGTGCTATTACTATATTATTAACAGATCGAAATCTTAATACATCATTCTTTGATCCAGCAGGAGGAGGAGACCCAATCTTGTACCAACATTTATTTTGATTCTTTGGCACC

>MH401769

AGAATTCTAATTCGAGCCGAACTAGGACATCCTGGAGCATTAATTGGAGATGATCAAATTTATAATGTAATTGTTACAGCTCATGCTTTTATTATAATTTTCTTTATAGTAATACCAATTATAATTGGAGGATTTGGTAATTGATTAGTCCCTTTAATATTAGGAGCTCCAGATATAGCCTTCCCTCGGATAAACAATATAAGTTTCTGACTTTTACCTCCTGCATTAACTTTACTATTAGTAAGTAGTATAGTAGAAAACGGAGCTGGAACAGGATGAACTGTTTACCCACCTTTATCTTCTAATATCGCTCATGGAGGAGCTTCTGTTGATTTAGCTATTTTTTCTTTACACTTAGCAGGAATTTCTTCAATTTTAGGAGCTGTAAATTTTATTACTACAGTTATTAATATACGATCAACAGGAATTACATTCGACCGAATACCATTATTTGTTTGATCTGTAGTAATTACAGCTTTATTACTTTTATTATCTTTACCAGTATTAGCAGGTGCTATTACTATATTATTAACAGATCGAAATCTTAATACATCATTCTTTGATCCAGCAGGAGGAGGAGACCCAATCTTGTACCAACATTTATTTTGATTTTTTGGACAT

>MH401770

AGAATTCTAATTCGAGCTGAACTAGGACATCCCGGAGCTCTAATTGGAGATGACCAAATTTATAATGTAATTGTAACAGCTCATGCTTTTATTATAATTTTCTTTATAGTAATGCCAATTATAATTGGAGGATTTGGAAATTGATTAGTTCCTTTAATACTAGGGGCACCTGATATAGCTTTCCCACGAATAAACAATATAAGTTTCTGACTTTTACCCCCCGCATTAACTCTATTGTTAGTAAGTAGTATAGTAGAAAATGGAGCTGGAACAGGATGAACTGTTTACCCACCCTTATCATCTAATATTGCCCATGGAGGAGCATCTGTTGATCTGGCTATTTTCTCTCTTCACTTAGCAGGAATTTCTTCAATTTTAGGGGCTGTAAATTTCATTACGACTGTAATTAATATACGATCAACTGGAATTACATTTGATCGAATACCTCTATTTGTTTGATCTGTAGTTATTACTGCTCTATTACTTTTATTATCTTTACCTGTATTAGCAGGTGCTATTACTATATTATTAACTGATCGAAATTTAAATACTTCATTCTTTGATCCAGCAGGAGGAGGAGATCCTATTCTATACCAACACTTATTTTGATTTTTTGGACATCCA

>MH401771

AGAATTCTAATTCGAGCTGAATTAGGACACCCTGGTGCACTAATTGGAGATGACCAAATTTATAATGTAATTGTTACAGCTCATGCTTTTATTATAATTTTCTTTATAGTAATACCAATTATAATTGGAGGATTTGGTAATTGATTAGTTCCTTTAATGTTAGGAGCCCCAGATATAGCCTTCCCTCGATTAAATAATATAAGATTTTGACTTTTACCTCCTGCACTAACTTTATTATTAGTAAGTAGTATAGTAGAAAACGGAGCTGGAACAGGATGAACAGTTTACCCCCCTTTATCATCTAATATCGCTCATGGAGGAGCTTCTGTTGATTTAGCTATTTTTTCTCTTCATTTAGCAGGAATTTCTTCAATTTTAGGGGCTGTAAATTTTATTACTACAGTCATTAATATACGATCAACAGGAATTACTTTTGACCGAATACCTTTATTTGTTTGATCAGTTGTAATTACAGCTCTATTACTTTTATTATCATTACCAGTATTAGCAGGAGCTATTACAATACTTTTAACTGACCGAAACCTTAATACTTCATTTTTTGACCCTGCAGGAGGAGGAGACCCAATTCTATACCAACATCTATTTTGATTTTTTGGACAT

>MH401772

AGAATTCTAATTCGAGCTGAATTAGGACATCCTGGAGCTTTAATTGGAGATGATCAAATTTATAATGTAATTGTTACAGCTCATGCTTTTATTATAATTTTTTTTATAGTAATGCCAATTATAATTGGAGGATTTGGAAATTGATTAGTTCCATTAATACTAGGAGCTCCAGATATAGCATTCCCTCGAATAAATAATATAAGTTTTTGACTTTTACCTCCTGCATTAACTTTATTATTAGTTAGTAGTATAGTAGAAAACGGAGCTGGAACAGGATGAACAGTTTACCCTCCTCTATCTTCTAATATTGCTCATGGAGGAGCTTCTGTTGATTTAGCTATTTTCTCTCTTCATTTAGCAGGAATTTCTTCAATTTTAGGAGCTGTAAATTTTATTACTACAGTTATTAATATACGATCAACAGGAATTACTTTTGATCGAATACCTTTATTTGTTTGATCAGTAGTAATTACAGCTTTATTACTTTTATTATCATTACCAGTATTAGCAGGAGCTATTACAATACTTTTAACAGACCGAAATCTTAATACATCATTCTTTGACCCTGCAGGAGGAGGAGATCCAATTTTATACCAACATTTATTTTGATTCTTTGGCAC

>MH401773

AGAATTCTAATTCGAGCCGAACTAGGACATCCTGGAGCATTAATTGGAGATGACCAAATTTATAATGTAATTGTTACAGCTCATGCTTTTATTATAATTTTTTTTATAGTAATACCAATTATAATTGGAGGATTTGGTAATTGATTAGTTCCTTTAATATTAGGAGCTCCAGATATAGCATTCCCTCGGATAAACAATATAAGTTTCTGACTTTTACCTCCTGCATTAACTTTACTATTAGTAAGTAGTATAGTAGAAAACGGAGCTGGAACAGGATGAACTGTTTACCCACCTTTATCTTCTAATATCGCTCATGGAGGAGCTTCTGTTGATTTAGCTATTTTTTCTTTACACTTGGCAGGAATTTCTTCAATTTTAGGAGCTGTAAATTTTATTACTACAGTTATTAATATACGATCAACAGGAATTACATTCGACCGAATACCATTATTTGTTTGATCTGTAGTAATTACAGCTTTATTACTTTTATTATCTTTACCAGTATTAGCAGGTGCTATTACTATATTATTAACAGATCGAAATCTTAATACATCATTCTTTGACCCAGCAGGAGGAGGAGACCCAATCTTGTACCAACATTTATTTTGATTCTTTGGCTC

>MH401774

AGAATTCTAATTCGAGCTGAATTAGGACATCCTGGAGCTTTAATTGGAGATGATCAAATTTATAATGTAATTGTTACAGCTCATGCTTTTATTATAATTTTTTTTATAGTAATGCCAATTATAATTGGAGGATTTGGAAATTGATTAGTTCCATTAATACTAGGAGCTCCAGATATAGCATTCCCTCGAATAAATAATATAAGTTTTTGACTTTTACCTCCTGCATTAACTTTATTATTAGTTAGTAGTATAGTAGAAAACGGAGCTGGAACAGGATGAACAGTTTACCCTCCTCTATCTTCTAATATTGCTCATGGAGGAGCTTCTGTTGATTTAGCTATTTTCTCTCTTCATTTAGCAGGAATTTCTTCAATTTTAGGAGCTGTAAATTTTATTACTACAGTTATTAATATACGATCAACAGGAATTACTTTTGATCGAATACCTTTATTTGTTTGATCAGTAGTAATTACAGCTTTATTACTTTTATTATCATTACCAGTATTAGCAGGAGCTATTACAATACTTTTAACAGACCGAAATCTTAATACATCATTCTTTGACCCTGCAGGAGGAGGAGATCCAATTTTATACCAACATTTATTTTGATTCTTTGGACACCG

>MH401775

AGAATTTTAATTCGAGCTGAACTAGGGCATCCTGGAGCATTAATTGGAGATGATCAAATTTATAATGTAATTGTTACAGCTCATGCTTTTATTATAATTTTTTTTATAGTAATACCAATTATAATTGGAGGGTTTGGAAATTGATTAGTTCCTTTAATATTAGGAGCCCCAGATATAGCATTCCCTCGAATAAATAATATAAGTTTCTGACTTTTACCTCCTGCATTAACTTTACTATTAGTAAGTAGTATAGTAGAAAACGGAGCTGGAACTGGATGAACTGTTTATCCACCTTTATCTTCTAATATTGCACATGGAGGAGCTTCTGTTGATTTAGCTATTTTTTCTTTACATTTAGCAGGAATTTCTTCAATTTTAGGAGCTGTAAATTTTATTACTACAGTTATTAATATACGATCAACAGGTATTACCTTCGACCGAATACCATTATTTGTTTGATCAGTAGTAATTACAGCCTTATTACTTTTATTATCTTTACCAGTATTAGCAGGAGCTATTACTATATTATTAACAGATCGAAATCTTAATACTTCATTCTTTGACCCAGCAGGAGGAGGAGATCCAATTTTATACCAACACTTATTTTGATTCTTTGGACACCAG

>MH401776

AGAATTCTAATTCGAGCCGAACTAGGACATCCTGGAGCATTAATTGGAGATGACCAAATTTATAATGTAATTGTTACAGCTCATGCTTTTATTATAATTTTTTTTATAGTAATACCAATTATAATTGGAGGATTTGGTAATTGATTAGTCCCTTTAATATTAGGAGCTCCAGATATAGCCTTCCCTCGGATAAACAATATAAGTTTCTGACTTTTACCTCCTGCATTAACTTTACTATTAGTAAGTAGTATAGTAGAAAACGGAGCTGGAACAGGATGAACTGTTTACCCACCTTTATCTTCTAATATCGCTCATGGAGGAGCTTCTGTTGATTTAGCTATTTTTTCTTTACACTTAGCAGGAATTTCTTCAATTTTAGGAGCTGTAAATTTTATTACTACAGTTATTAATATACGATCAACAGGAATTACATTCGACCGAATACCATTATTTGTTTGATCTGTAGTAATTACAGCTTTATTACTTTTATTATCTTTACCAGTATTAGCAGGTGCTATTACTATATTATTAACAGATCGAAATCTTAATACATCATTCTTTGACCCAGCAGGAGGAGGAGACCCAATCTTGTACCAACATTTATTTTGATTTTTTGG

>MH401777

AGAATTCTAATTCGAGCCGAACTAGGACATCCTGGAGCATTAATTGGAGATGACCAAATTTATAATGTAATTGTTACAGCTCATGCTTTTATTATAATTTTTTTTATAGTAATACCAATTATAATTGGAGGATTTGGTAATTGATTAGTCCCTTTAATATTAGGAGCTCCAGATATAGCCTTCCCTCGGATAAACAATATAAGTTTCTGACTTTTACCTCCTGCATTAACTTTACTATTAGTAAGTAGTATAGTAGAAAACGGAGCTGGAACAGGATGAACTGTTTACCCACCTTTATCTTCTAATATCGCTCATGGAGGAGCTTCTGTTGATTTAGCTATTTTTTCTTTACACTTAGCAGGAATTTCTTCAATTTTAGGAGCTGTAAATTTTATTACTACAGTTATTAATATACGATCAACAGGAATTACATTCGACCGAATACCATTATTTGTTTGATCTGTAGTAATTACAGCTTTATTACTTTTATTATCTTTACCAGTATTAGCAGGTGCTATTACTATATTATTAACAGATCGAAATCTTAATACATCATTCTTTGACCCAGCAGGAGGAGGAGACCCAATCTTGTACCAACATTTATTTTGATTCTTTGGAAAA

>MH401778

AGAATTCTAATTCGAGCTGAATTAGGACATCCTGGAGCTTTAATTGGAGACGATCAAATTTATAATGTAATTGTTACAGCTCATGCTTTTATTATAATTTTTTTTATAGTAATGCCAATTATAATTGGAGGATTTGGAAATTGATTAGTTCCATTAATACTAGGAGCTCCAGATATAGCATTCCCTCGAATAAATAATATAAGTTTTTGACTTTTACCTCCTGCATTAACTTTATTATTAGTTAGTAGTATAGTAGAAAACGGAGCTGGAACAGGATGAACAGTTTACCCTCCTCTATCTTCTAATATTGCTCATGGAGGAGCTTCTGTTGATTTAGCTATTTTCTCTCTTCATTTAGCAGGAATTTCTTCAATTTTAGGAGCTGTAAATTTTATTACTACAGTTATTAATATACGATCAACAGGAATTACTTTTGATCGAATACCTTTATTTGTTTGATCAGTAGTAATTACAGCTTTATTACTTTTATTATCATTACCAGTATTAGCAGGAGCTATTACAATACTTTTAACAGACCGAAATCTTAATACATCATTCTTTGACCCTGCAGGAGGAGGAGATCCAATTTTATACCAACATTTATTTTGATTTTTGG

>MH401779

AGAATTCTAATTCGAGCTGAATTAGGACATCCTGGAGCTTTAATTGGAGATGATCAAATTTATAATGTAATTGTTACAGCTCATGCTTTTATTATAATTTTTTTTATAGTAATGCCAATTATAATTGGAGGATTTGGAAATTGATTAGTTCCATTAATACTAGGAGCTCCAGATATAGCATTCCCTCGAATAAATAATATAAGTTTTTGACTTTTACCTCCTGCATTAACTTTATTATTAGTTAGTAGTATAGTAGAAAACGGAGCTGGAACAGGATGAACAGTTTACCCTCCTCTATCTTCTAATATTGCTCATGGAGGAGCTTCTGTTGATTTAGCTATTTTCTCTCTTCATTTAGCAGGAATTTCTTCAATTTTAGGAGCTGTAAATTTTATTACTACAGTTATTAATATACGATCAACAGGAATTACTTTTGATCGAATACCTTTATTTGTTTGATCAGTAGTAATTACAGCTTTATTACTTTTATTATCATTACCAGTATTAGCAGGAGCTATTACAATACTTTTAACAGACCGAAATCTTAATACATCATTCTTTGACCCTGCAGGAGGAGGAGATCCAATTTTATACCAACATTTATTTTGATTCTTTGGCTAC

>MH401780

AGAATTCTAATTCGAGCCGAACTAGGACATCCTGGAGCATTAATTGGAGATGACCAAATTTATAATGTAATTGTTACAGCTCATGCTTTTATTATAATTTTTTTTATAGTAATACCAATTATAATTGGAGGATTTGGTAATTGATTAGTCCCTTTAATATTAGGAGCTCCAGATATAGCCTTCCCTCGGATAAACAATATAAGTTTCTGACTTTTACCTCCTGCATTAACTTTACTATTAGTAAGTAGTATAGTAGAAAACGGAGCTGGAACAGGATGAACTGTTTACCCACCTTTATCTTCTAATATCGCTCATGGAGGAGCTTCTGTTGATTTAGCTATTTTTTCTTTACACTTAGCAGGAATTTCTTCAATTTTAGGAGCTGTAAATTTTATTACTACAGTTATTAATATACGATCAACAGGAATTACATTCGACCGAATACCATTATTTGTTTGATCTGTAGTAATTACAGCTTTATTACTTTTATTATCTTTACCAGTATTAGCAGGTGCTATTACTATATTATTAACAGATCGAAATCTTAATACATCATTCTTTGACCCAGCAGGAGGAGGAGACCCAATCTTGTACCAACATTTATTTTGATTCTTTGGAAAA

>MH401781

AGAATTCTAATTCGAGCTGAATTAGGACATCCTGGAGCTTTAATTGGAGATGATCAAATTTATAATGTAATTGTTACAGCTCATGCTTTTATTATAATTTTTTTTATAGTAATGCCAATTATAATTGGAGGATTTGGAAATTGATTAGTTCCATTAATACTAGGAGCTCCAGATATAGCATTCCCTCGAATAAATAATATAAGTTTTTGACTTTTACCTCCTGCATTAACTTTATTATTAGTTAGTAGTATAGTAGAAAACGGAGCTGGAACAGGATGAACAGTTTACCCTCCTCTATCTTCTAATATTGCTCATGGAGGAGCTTCTGTTGATTTAGCTATTTTCTCTCTTCATTTAGCAGGAATTTCTTCAATTTTAGGAGCTGTAAATTTTATTACTACAGTTATTAATATACGATCAACAGGAATTACTTTTGATCGAATACCTTTATTTGTTTGATCAGTAGTAATTACAGCTTTATTACTTTTATTATCATTACCAGTATTAGCAGGAGCTATTACAATACTTTTAACAGACCGAAATCTTAATACATCATTCTTTGACCCTGCAGGAGGAGGAGATCCAATTTTATACCAACATTTATTTTGATTCTTTGGCCC

>MH401782

AGAATTCTAATTCGAGCCGAACTAGGACATCCTGGAGCATTAATTGGAGATGACCAAATTTATAATGTAATTGTTACAGCTCATGCTTTTATTATAATTTTTTTTATAGTAATACCAATTATAATTGGAGGATTTGGTAATTGATTAGTCCCTTTAATATTAGGAGCTCCAGATATAGCCTTCCCTCGGATAAACAATATAAGTTTCTGACTTTTACCTCCTGCATTAACTTTACTATTAGTAAGTAGTATAGTAGAAAACGGAGCTGGAACAGGATGAACTGTTTACCCACCTTTATCTTCTAATATCGCTCATGGAGGAGCTTCTGTTGATTTAGCTATTTTTTCTTTACACTTAGCAGGAATTTCTTCAATTTTAGGAGCTGTAAATTTTATTACTACAGTTATTAATATACGATCAACAGGAATTACATTCGACCGAATACCATTATTTGTTTGATCTGTAGTAATTACAGCTTTATTACTTTTATTATCTTTACCAGTATTAGCAGGTGCTATTACTATATTATTAACAGATCGAAATCTTAATACATCATTCTTTGACCCAGCAGGAGGAGGAGACCCAATCTTGTACCAACATTTATTTTGATTCTTTGGCTAC

>MH401783

AGAATTCTAATTCGAGCTGAATTAGGACATCCTGGAGCTTTAATTGGAGATGATCAAATTTATAATGTAATTGTTACAGCTCATGCTTTTATTATAATTTTTTTTATAGTAATGCCAATTATAATTGGAGGATTTGGAAATTGATTAGTTCCATTAATACTAGGAGCTCCAGATATAGCATTCCCTCGAATAAATAATATAAGTTTTTGACTTTTACCTCCTGCATTAACTTTATTATTAGTTAGTAGTATAGTAGAAAACGGAGCTGGAACAGGATGAACAGTTTACCCTCCTCTATCTTCTAATATTGCTCATGGAGGAGCTTCTGTTGATTTAGCTATTTTCTCTCTTCATTTAGCAGGAATTTCTTCAATTTTAGGAGCTGTAAATTTTATTACTACAGTTATTAATATACGATCAACAGGAATTACTTTTGATCGAATACCTTTATTTGTTTGATCAGTAGTAATTACAGCTTTATTACTTTTATTATCATTACCAGTATTAGCAGGAGCTATTACAATACTTTTAACAGACCGAAATCTTAATACATCATTCTTTGACCCTGCAGGAGGAGGAGATCCAATTTTATACCAACATTTATTTTGATTCTTTG

>MH401784

AGAATTCTAATTCGAGCCGAACTAGGACATCCTGGAGCATTAATTGGAGATGACCAAATTTATAATGTAATTGTTACAGCTCATGCTTTTATTATAATTTTTTTTATAGTAATACCAATTATAATTGGAGGATTTGGTAATTGATTAGTCCCTTTAATATTAGGAGCTCCAGATATAGCCTTCCCTCGGATAAACAATATAAGTTTCTGACTTTTACCTCCTGCATTAACTTTACTATTAGTAAGTAGTATAGTAGAAAACGGAGCTGGAACAGGATGAACTGTTTACCCACCTTTATCTTCTAATATCGCTCATGGAGGAGCTTCTGTTGATTTAGCTATTTTTTCTTTACACTTAGCAGGAATTTCTTCAATTTTAGGAGCTGTAAATTTTATTACTACAGTTATTAATATACGATCAACAGGAATTACATTCGACCGAATACCATTATTTGTTTGATCTGTAGTAATTACAGCTTTATTACTTTTATTATCTTTACCAGTATTAGCAGGTGCTATTACTATATTATTAACAGATCGAAATCTTAATACATCATTCTTTGACCCAGCAGGAGGAGGAGACCCAATCTTGTACCAACATTTATTTTGATTTTTTGG

>MH401785

AGAATTCTAATTCGAGCTGAATTAGGACATCCTGGAGCTTTAATTGGAGATGATCAAATTTATAATGTAATTGTTACAGCTCATGCTTTTATTATAATTTTTTTTATAGTAATGCCAATTATAATTGGAGGATTTGGAAATTGATTAGTTCCATTAATACTAGGAGCTCCAGATATAGCATTCCCTCGAATAAATAATATAAGTTTTTGACTTTTACCTCCTGCATTAACTTTATTATTAGTTAGTAGTATAGTAGAAAACGGAGCTGGAACAGGATGAACAGTTTACCCTCCTCTATCTTCTAATATTGCTCATGGAGGAGCTTCTGTTGATTTAGCTATTTTCTCTCTTCATTTAGCAGGAATTTCTTCAATTTTAGGAGCTGTAAATTTTATTACTACAGTTATTAATATACGATCAACAGGAATTACTTTTGATCGAATACCTTTATTTGTTTGATCAGTAGTAATTACAGCTTTATTACTTTTATTATCATTACCAGTATTAGCAGGAGCTATTACAATACTTTTAACAGACCGAAATCTTAATACATCATTCTTTGACCCTGCAGGAGGAGGAGATCCAATTTTATACCAACATTTATTTTGATTCTTTGGCTACGA

>MH401786

AGAATTCTAATTCGAGCCGAACTAGGACATCCTGGAGCATTAATTGGAGATGACCAAATTTATAATGTAATTGTTACAGCTCATGCTTTTATTATAATTTTTTTTATAGTAATACCAATTATAATTGGAGGATTTGGTAATTGATTAGTCCCTTTAATATTAGGAGCTCCAGATATAGCCTTCCCTCGGATAAACAATATAAGTTTCTGACTTTTACCTCCTGCATTAACTTTACTATTAGTAAGTAGTATAGTAGAAAACGGAGCTGGAACAGGATGAACTGTTTACCCACCTTTATCTTCTAATATCGCTCATGGAGGAGCTTCTGTTGATTTAGCTATTTTTTCTTTACACTTAGCAGGAATTTCTTCAATTTTAGGAGCTGTAAATTTTATTACTACAGTTATTAATATACGATCAACAGGAATTACATTCGACCGAATACCATTATTTGTTTGATCTGTAGTAATTACAGCTTTATTACTTTTATTATCTTTACCAGTATTAGCAGGTGCTATTACTATATTATTAACAGATCGAAATCTTAATACATCATTCTTTGACCCAGCAGGAGGAGGAGACCCAATCTTGTACCAACATTTATTTTGATTCTTTGG

>MH401787

AGAATTCTAATTCGAGCTGAATTAGGACATCCTGGAGCTTTAATTGGAGATGATCAAATTTATAATGTAATTGTTACAGCTCATGCTTTTATTATAATTTTTTTTATAGTAATGCCAATTATAATTGGAGGATTTGGAAATTGATTAGTTCCATTAATACTAGGAGCTCCAGATATAGCATTCCCTCGAATAAATAATATAAGTTTTTGACTTTTACCTCCTGCATTAACTTTATTATTAGTTAGTAGTATAGTAGAAAACGGAGCTGGAACAGGATGAACAGTTTACCCTCCTCTATCTTCTAATATTGCTCATGGAGGAGCTTCTGTTGATTTAGCTATTTTCTCTCTTCATTTAGCAGGAATTTCTTCAATTTTAGGAGCTGTAAATTTTATTACTACAGTTATTAATATACGATCAACAGGAATTACTTTTGATCGAATACCTTTATTTGTTTGATCAGTAGTAATTACAGCTTTATTACTTTTATTATCATTACCAGTATTAGCAGGAGCTATTACAATACTTTTAACAGACCGAAATCTTAATACATCATTCTTTGACCCTGCAGGAGGAGGAGATCCAATTTTATACCAACATTTATTTTGATTCTTTGGCTAC

>MH401788

AGAATTCTAATTCGAGCCGAACTAGGACATCCTGGAGCATTAATTGGAGATGACCAAATTTATAATGTAATTGTTACAGCTCATGCTTTTATTATAATTTTTTTTATAGTAATACCAATTATAATTGGAGGATTTGGTAATTGATTAGTCCCTTTAATATTAGGAGCTCCAGATATAGCCTTCCCTCGGATAAACAATATAAGTTTCTGACTTTTACCTCCTGCATTAACTTTACTATTAGTAAGTAGTATAGTAGAAAACGGAGCTGGAACAGGATGAACTGTTTACCCACCTTTATCTTCTAATATCGCTCATGGAGGAGCTTCTGTTGATTTAGCTATTTTTTCTTTACACTTAGCAGGAATTTCTTCAATTTTAGGAGCTGTAAATTTTATTACTACAGTTATTAATATACGATCAACAGGAATTACATTCGACCGAATACCATTATTTGTTTGATCTGTAGTAATTACAGCTTTATTACTTTTATTATCTTTACCAGTATTAGCAGGTGCTATTACTATATTATTAACAGATCGAAATCTTAATACATCATTCTTTGACCCAGCAGGAGGAGGAGACCCAATCTTGTACCAACATTTATTTTGATTCTTTGGAAA

>MH401789

AGAATTCTAATTCGAGCTGAATTAGGACATCCTGGAGCTTTAATTGGAGATGATCAAATTTATAATGTAATTGTTACAGCTCATGCTTTTATTATAATTTTTTTTATAGTAATGCCAATTATAATTGGAGGATTTGGAAATTGATTAGTTCCATTAATACTAGGAGCTCCAGATATAGCATTCCCTCGAATAAATAATATAAGTTTTTGACTTTTACCTCCTGCATTAACTTTATTATTAGTTAGTAGTATAGTAGAAAACGGAGCTGGAACAGGATGAACAGTTTACCCTCCTCTATCTTCTAATATTGCTCATGGAGGAGCTTCTGTTGATTTAGCTATTTTCTCTCTTCATTTAGCAGGAATTTCTTCAATTTTAGGAGCTGTAAATTTTATTACTACAGTTATTAATATACGATCAACAGGAATTACTTTTGATCGAATACCTTTATTTGTTTGATCAGTAGTAATTACAGCTTTATTACTTTTATTATCATTACCAGTATTAGCAGGAGCTATTACAATACTTTTAACAGACCGAAATCTTAATACATCATTCTTTGACCCTGCAGGAGGAGGAGATCCAATTTTATACCAACATTTATTTTGATTCTTTGGCTACA

>MH401790

AGAATTCTAATTCGAGCCGAACTAGGACATCCTGGAGCATTAATTGGAGATGACCAAATTTATAATGTAATTGTTACAGCTCATGCTTTTATTATAATTTTTTTTATAGTAATACCAATTATAATTGGAGGATTTGGTAATTGATTAGTCCCTTTAATATTAGGAGCTCCAGATATAGCCTTCCCTCGGATAAACAATATAAGTTTCTGACTTTTACCTCCTGCATTAACTTTACTATTAGTAAGTAGTATAGTAGAAAACGGAGCTGGAACAGGATGAACTGTTTACCCACCTTTATCTTCTAATATCGCTCATGGAGGAGCTTCTGTTGATTTAGCTATTTTTTCTTTACACTTAGCAGGAATTTCTTCAATTTTAGGAGCTGTAAATTTTATTACTACAGTTATTAATATACGATCAACAGGAATTACATTCGACCGAATACCATTATTTGTTTGATCTGTAGTAATTACAGCTTTATTACTTTTATTATCTTTACCAGTATTAGCAGGTGCTATTACTATATTATTAACAGATCGAAATCTTAATACATCATTCTTTGACCCAGCAGGAGGAGGAGACCCAATCTTGTACCAACATTTATTTTGATTCTTTGGACACAA

>MH401791

AGAATTCTAATTCGAGCTGAATTAGGACATCCTGGAGCTTTAATTGGAGACGATCAAATTTATAATGTAATTGTTACAGCTCATGCTTTTATTATAATTTTTTTTATAGTAATGCCAATTATAATTGGAGGATTTGGAAATTGATTAGTTCCATTAATACTAGGAGCTCCAGATATAGCATTCCCTCGAATAAATAATATAAGTTTTTGACTTTTACCTCCTGCATTAACTTTATTATTAGTTAGTAGTATAGTAGAAAACGGAGCTGGAACAGGATGAACAGTTTACCCTCCTCTATCTTCTAATATTGCTCATGGAGGAGCTTCTGTTGATTTAGCTATTTTCTCTCTTCATTTAGCAGGAATTTCTTCAATTTTAGGAGCTGTAAATTTTATTACTACAGTTATTAATATACGATCAACAGGAATTACTTTTGATCGAATACCTTTATTTGTTTGATCAGTAGTAATTACAGCTTTATTACTTTTATTATCATTACCAGTATTAGCAGGAGCTATTACAATACTTTTAACAGACCGAAATCTTAATACATCATTCTTTGACCCTGCAGGAGGAGGAGATCCAATTTTATACCAACATTTATTTTGATTCTTTGGA

>MH401792

AGAATTCTAATTCGAGCCGAACTAGGACATCCTGGAGCATTAATTGGAGATGACCAAATTTATAATGTAATTGTTACAGCTCATGCTTTTATTATAATTTTTTTTATAGTAATACCAATTATAATTGGAGGATTTGGTAATTGATTAGTCCCTTTAATATTAGGAGCTCCAGATATAGCCTTCCCTCGGATAAACAATATAAGTTTCTGACTTTTACCTCCTGCATTAACTTTACTATTAGTAAGTAGTATAGTAGAAAACGGAGCTGGAACAGGATGAACTGTTTACCCACCTTTATCTTCTAATATCGCTCATGGAGGAGCTTCTGTTGATTTAGCTATTTTTTCTTTACACTTAGCAGGAATTTCTTCAATTTTAGGAGCTGTAAATTTTATTACTACAGTTATTAATATACGATCAACAGGAATTACATTCGACCGAATACCATTATTTGTTTGATCTGTAGTAATTACAGCTTTATTACTTTTATTATCTTTACCAGTATTAGCAGGTGCTATTACTATATTATTAACAGATCGAAATCTTAATACATCATTCTTTGACCCAGCAGGAGGAGGAGACCCAATCTTGTACCAACATTTATTTTGATTCTTTG

>MH401793

AGAATTCTAATTCGAGCTGAATTAGGACATCCTGGAGCTTTAATTGGAGATGATCAAATTTATAATGTAATTGTTACAGCTCATGCTTTTATTATAATTTTTTTTATAGTAATGCCAATTATAATTGGAGGATTTGGAAATTGATTAGTTCCATTAATACTAGGAGCTCCAGATATAGCATTCCCTCGAATAAATAATATAAGTTTTTGACTTTTACCTCCTGCATTAACTTTATTATTAGTTAGTAGTATAGTAGAAAACGGAGCTGGAACAGGATGAACAGTTTACCCTCCTCTATCTTCTAATATTGCTCATGGAGGAGCTTCTGTTGATTTAGCTATTTTCTCTCTTCATTTAGCAGGAATTTCTTCAATTTTAGGAGCTGTAAATTTTATTACTACAGTTATTAATATACGATCAACAGGAATTACTTTTGATCGAATACCTTTATTTGTTTGATCAGTAGTAATTACAGCTTTATTACTTTTATTATCATTACCAGTATTAGCAGGAGCTATTACAATACTTTTAACAGACCGAAATCTTAATACATCATTCTTTGACCCTGCAGGAGGAGGAGATCCAATTTTATACCAACATTTATTTTGATTCTTTGGCTA

>MH401794

AGAATCCTAATTCGAGCAGAACTAGGTCACCCTGGTGCATTAATTGGAGATGATCAAATTTATAATGTAATTGTTACAGCTCATGCTTTTATTATAATTTTTTTTATAGTAATACCAATCATAATTGGAGGATTTGGAAACTGACTAGTTCCAATTATACTAGGAGCTCCAGATATAGCTTTCCCTCGAATAAATAATATAAGATTTTGACTTTTACCTCCTGCATTAACATTACTACTAGTAAGTAGTATAGTAGAAAATGGAGCTGGAACAGGATGAACTGTTTACCCTCCTTTATCATCTAATATTGCTCATGGAGGAGCTTCTGTTGATCTAGCTATTTTTTCTCTTCACTTAGCTGGAATTTCTTCAATTTTAGGAGCAGTAAATTTTATTACTACAGTAATTAATATACGATCTACAGGTATTACTTTTGATCGAATACCCCTTTTTGTTTGATCAGTAGTAATTACCGCTTTACTTCTCCTTCTATCCCTACCCGTACTTGCAGGAGCAATTACTATATTATTAACTGACCGAAATATTAATACTTCATTTTTTGATCCAGCAGGAGGAGGAGATCCAATTCTATATCAACACTTATTTTGATTCTTTGTC

>MH401795

AGAATTTTAATTCGAGCTGAACTAGGGCATCCTGGAGCATTAATTGGAGATGATCAAATTTATAATGTAATTGTTACAGCTCATGCTTTTATTATAATTTTTTTTATAGTAATACCAATTATAATTGGAGGGTTTGGAAATTGATTAGTTCCTTTAATATTAGGAGCCCCAGATATAGCATTCCCTCGAATAAATAATATAAGTTTCTGACTTTTACCTCCTGCATTAACTTTACTATTAGTAAGTAGTATAGTAGAAAACGGAGCTGGAACTGGATGAACTGTTTATCCACCTTTATCTTCTAATATTGCACATGGAGGAGCTTCTGTTGATTTAGCTATTTTTTCTTTACATTTAGCAGGAATTTCTTCAATTTTAGGAGCTGTAAATTTTATTACTACAGTTATTAATATACGATCAACAGGTATTACCTTCGACCGAATACCATTATTTGTTTGATCAGTAGTAATTACAGCCTTATTACTTTTATTATCTTTACCAGTATTAGCAGGAGCTATTACTATATTATTAACAGATCGAAATCTTAATACTTCATTCTTTGACCCAGCAGGAGGAGGAGATCCAATTTTATACCAACACTTATTTTGATTCTTTGGCA

>MH401798

AGAATTCTAATTCGAGCCGAACTAGGACATCCTGGAGCATTAATTGGAGATGACCAAATTTATAATGTAATTGTTACAGCTCATGCTTTTATTATAATTTTTTTTATAGTAATACCAATTATAATTGGAGGATTTGGTAATTGATTAGTCCCTTTAATATTAGGAGCTCCAGATATAGCCTTCCCTCGGATAAACAATATAAGTTTCTGACTTTTACCTCCTGCATTAACTTTACTATTAGTAAGTAGTATAGTAGAAAACGGAGCTGGAACAGGATGAACTGTTTACCCACCTTTATCTTCTAATATCGCTCATGGAGGAGCTTCTGTTGATTTAGCTATTTTTTCTTTACACTTAGCAGGAATTTCTTCAATTTTAGGAGCTGTAAATTTTATTACTACAGTTATTAATATACGATCAACAGGAATTACATTCGACCGAATACCATTATTTGTTTGATCTGTAGTAATTACAGCTTTATTACTTTTATTATCTTTACCAGTATTAGCAGGTGCTATTACTATATTATTAACAGATCGAAATCTTAATACATCATTCTTTGACCCAGCAGGAGGAGGAGACCCAATCTTGTACCAACATTTATTTTGATTCTTTGGC

>MH401799

AGAATTTTAATTCGAGCTGAATTAGGTCATCCTGGTGCATTAATTGGTAATGATCAAATTTATAATGTAATTGTAACAGCTCATGCTTTTATTATAATTTTTTTTATAGTTATACCTATTATAATTGGAGGATTTGGAAATTGATTAGTTCCTTTAATATTAGGAGCTCCTGATATAGCTTTCCCTCGAATAAATAACATAAGCTTTTGACTTCTACCTCCTGCTTTAACTTTACTTTTAGTAAGCAGAATAGTTGAAAATGGAGCTGGTACAGGTTGAACTGTCTACCCCCCTTTATCATCTAATATTGCTCATGGAGGCGCTTCTGTTGATTTAGCTATTTTTTCATTACATTTAGCAGGAATTTCTTCAATTTTAGGAGCCGTAAATTTTATTACTACTGTAATTAATATACGTTCAACTGGAATTACTTTTGATCGAATACCTTTATTTGTTTGATCCGTAGTAATTACAGCTCTTTTATTATTACTATCTCTACCAGTTTTAGCTGGAGCAATTACTATACTATTAACAGATCGAAATTTAAATACTTCTTTTTTTGATCCTGCAGGAGGAGGAGATCCTATTTTATATCAACATTTATTTTGATTCTTTGGAACA

>MH401800

AGAATTCTAATTCGAGCTGAATTAGGACATCCTGGAGCACTAATTGGAGATGACCAAATTTATAATGTAATTGTAACAGCTCATGCCTTTATTATAATTTTCTTTATAGTAATACCAATTATAATTGGAGGATTTGGAAATTGACTAGTTCCTTTAATATTAGGAGCCCCAGATATAGCTTTCCCACGAATAAATAATATAAGTTTCTGACTTTTACCTCCTGCATTAACTTTACTATTAGTAAGTAGTATAGTAGAAAATGGAGCTGGAACAGGATGAACTGTTTATCCACCTTTATCATCTAATATTGCTCATGGTGGAGCATCAGTTGATTTAGCTATTTTTTCTTTACACTTAGCTGGAATTTCATCAATTTTAGGAGCTGTAAATTTTATTACAACTGTTATTAATATACGATCTACAGGAATCACATTTGATCGAATACCTTTATTCGTATGATCTGTAGTTATTACTGCTCTTCTTTTATTATTATCATTACCAGTATTAGCCGGTGCAATTACTATATTATTAACTGATCGAAATTTAAATACTTCATTCTTTGATCCAGCAGGAGGAGGAGATCCTATTTTATATCAACATTTATTTTGATTCTTTGG

>MH401801

AGAATTCTAATTCGAGCTGAATTAGGACATCCTGGAGCACTAATTGGAGATGACCAAATTTATAATGTAATTGTAACAGCTCATGCCTTTATTATAATTTTCTTTATAGTAATACCAATTATAATTGGAGGATTTGGAAATTGACTAGTTCCTTTAATATTAGGAGCCCCAGATATAGCTTTCCCACGAATAAATAATATAAGTTTCTGACTTTTACCTCCTGCATTAACTTTACTATTAGTAAGTAGTATAGTAGAAAATGGAGCTGGAACAGGATGAACTGTTTATCCACCTTTATCATCTAATATTGCTCATGGTGGAGCATCAGTTGATTTAGCTATTTTTTCTTTACACTTAGCTGGAATTTCATCAATTTTAGGAGCTGTAAATTTTATTACAACTGTTATTAATATACGATCTACAGGAATCACATTTGATCGAATACCTTTATTCGTATGATCTGTAGTTATTACTGCTCTTCTTTTATTATTATCATTACCAGTATTAGCCGGTGCAATTACTATATTATTAACTGATCGAAATTTAAATACTTCATTCTTTGATCCAGCAGGAGGAGGAGATCCTATTTTATATCAACATTTATTTTGATTCTTTGGA

>MH401802

AGAATTCTAATTCGAGCTGAATTAGGACATCCTGGAGCTTTAATTGGAGATGATCAAATTTATAATGTAATTGTTACAGCTCATGCTTTTATTATAATTTTTTTTATAGTAATGCCAATTATAATTGGAGGATTTGGAAATTGATTAGTTCCATTAATACTAGGAGCTCCAGATATAGCATTCCCTCGAATAAATAATATAAGTTTTTGACTTTTACCTCCTGCATTAACTTTATTATTAGTTAGTAGTATAGTAGAAAACGGAGCTGGAACAGGATGAACAGTTTACCCTCCTCTATCTTCTAATATTGCTCATGGAGGAGCTTCTGTTGATTTAGCTATTTTCTCTCTTCATTTAGCAGGAATTTCTTCAATTTTAGGAGCTGTAAATTTTATTACTACAGTTATTAATATACGATCAACAGGAATTACTTTTGATCGAATACCTTTATTTGTTTGATCAGTAGTAATTACAGCTTTATTACTTTTATTATCATTACCAGTATTAGCAGGAGCTATTACAATACTTTTAACAGACCGAAATCTTAATACATCATTCTTTGACCCTGCAGGAGGAGGAGATCCAATTTTATACCAACATTTATTTTGATTCTTTG

>MH401803

AGAATTCTAATTCGAGCCGAACTAGGACATCCTGGAGCATTAATTGGAGATGACCAAATTTATAATGTAATTGTTACAGCTCATGCTTTTATTATAATTTTTTTTATAGTAATACCAATTATAATTGGAGGATTTGGTAATTGATTAGTCCCTTTAATATTAGGAGCTCCAGATATAGCCTTCCCTCGGATAAACAATATAAGTTTCTGACTTTTACCTCCTGCATTAACTTTACTATTAGTAAGTAGTATAGTAGAAAACGGAGCTGGAACAGGATGAACTGTTTACCCACCTTTATCTTCTAATATCGCTCATGGAGGAGCTTCTGTTGATTTAGCTATTTTTTCTTTACACTTAGCAGGAATTTCTTCAATTTTAGGAGCTGTAAATTTTATTACTACAGTTATTAATATACGATCAACAGGAATTACATTCGACCGAATACCATTATTTGTTTGATCTGTAGTAATTACAGCTTTATTACTTTTATTATCTTTACCAGTATTAGCAGGTGCTATTACTATATTATTAACAGATCGAAATCTTAATACATCATTCTTTGACCCAGCAGGAGGAGGAGACCCAATCTTGTACCAACATTTATTTTGATTCTTTGGT

>MH401804

AGAATTCTAATTCGAGCCGAACTAGGACATCCTGGAGCATTAATTGGAGATGACCAAATTTATAATGTAATTGTTACAGCTCATGCTTTTATTATAATTTTTTTTATAGTAATACCAATTATAATTGGAGGATTTGGTAATTGATTAGTCCCTTTAATATTAGGAGCTCCAGATATAGCCTTCCCTCGGATAAACAATATAAGTTTCTGACTTTTACCTCCTGCATTAACTTTACTATTAGTAAGTAGTATAGTAGAAAACGGAGCTGGAACAGGATGAACTGTTTACCCACCTTTATCTTCTAATATCGCTCATGGAGGAGCTTCTGTTGATTTAGCTATTTTTTCTTTACACTTAGCAGGAATTTCTTCAATTTTAGGAGCTGTAAATTTTATTACTACAGTTATTAATATACGATCAACAGGAATTACATTCGACCGAATACCATTATTTGTTTGATCTGTAGTAATTACAGCTTTATTACTTTTATTATCTTTACCAGTATTAGCAGGTGCTATTACTATATTATTAACAGATCGAAATCTTAATACATCATTCTTTGACCCAGCAGGAGGAGGAGACCCAATCTTGTACCAACATTTATTTTGATTTTTTGGACATCCAGAAAGTTTAAA

>MH401805

AGAATTCTAATTCGAGCCGAACTAGGACATCCTGGAGCATTAATTGGAGATGACCAAATTTATAATGTAATTGTTACAGCTCATGCTTTTATTATAATTTTTTTTATAGTAATACCAATTATAATTGGAGGATTTGGTAATTGATTAGTCCCTTTAATATTAGGAGCTCCAGATATAGCCTTCCCTCGGATAAACAATATAAGTTTCTGACTTTTACCTCCTGCATTAACTTTACTATTAGTAAGTAGTATAGTAGAAAACGGAGCTGGAACAGGATGAACTGTTTACCCACCTTTATCTTCTAATATCGCTCATGGAGGAGCTTCTGTTGATTTAGCTATTTTTTCTTTACACTTAGCAGGAATTTCTTCAATTTTAGGAGCTGTAAATTTTATTACTACAGTTATTAATATACGATCAACAGGAATTACATTCGACCGAATACCATTATTTGTTTGATCTGTAGTAATTACAGCTTTATTACTTTTATTATCTTTACCAGTATTAGCAGGTGCTATTACTATATTATTAACAGATCGAAATCTTAATACATCATTCTTTGACCCAGCAGGAGGAGGAGACCCAATCTTGTACCAACATTTATTTTGATTTTTTGGT

>MH401806

AGAATTCTAATTCGAGCCGAACTAGGACATCCTGGAGCATTAATTGGAGATGACCAAATTTATAATGTAATTGTTACAGCTCATGCTTTTATTATAATTTTTTTTATAGTAATACCAATTATAATTGGAGGATTTGGTAATTGATTAGTCCCTTTAATATTAGGAGCTCCAGATATAGCCTTCCCTCGGATAAACAATATAAGTTTCTGACTTTTACCTCCTGCATTAACTTTACTATTAGTAAGTAGTATAGTAGAAAACGGAGCTGGAACAGGATGAACTGTTTACCCACCTTTATCTTCTAATATCGCTCATGGAGGAGCTTCTGTTGATTTAGCTATTTTTTCTTTACACTTAGCAGGAATTTCTTCAATTTTAGGAGCTGTAAATTTTATTACTACAGTTATTAATATACGATCAACAGGAATTACATTCGACCGAATACCATTATTTGTTTGATCTGTAGTAATTACAGCTTTATTACTTTTATTATCTTTACCAGTATTAGCAGGTGCTATTACTATATTATTAACAGATCGAAATCTTAATACATCATTCTTTGACCCAGCAGGAGGAGGAGACCCAATCTTGTACCAACATTTATTTTGATTCTTTGG

>MH401807

AGAATTCTAATTCGAGCCGAACTAGGACATCCTGGAGCATTAATTGGAGATGACCAAATTTATAATGTAATTGTTACAGCTCATGCTTTTATTATAATTTTTTTTATAGTAATACCAATTATAATTGGAGGATTTGGTAATTGATTAGTCCCTTTAATATTAGGAGCTCCAGATATAGCCTTCCCTCGGATAAACAATATAAGTTTCTGACTTTTACCTCCTGCATTAACTTTACTATTAGTAAGTAGTATAGTAGAAAACGGAGCTGGAACAGGATGAACTGTTTACCCACCTTTATCTTCTAATATTGCTCATGGAGGAGCTTCTGTTGATTTAGCTATTTTTTCTTTACACTTAGCAGGAATTTCTTCAATTTTAGGAGCTGTAAATTTTATTACTACAGTTATTAATATACGATCAACAGGAATTACATTCGACCGAATACCATTATTTGTTTGATCTGTAGTAATTACAGCTTTATTACTTTTATTATCTTTACCAGTATTAGCAGGTGCTATTACTATATTATTAACAGATCGAAATCTTAATACATCATTCTTTGACCCAGCAGGAGGAGGAGACCCAATCTTGTACCAACATTTATTTTGATTTTTTGGA

>MH401808

AGAATTCTAATTCGAGCTGAATTAGGACATCCTGGAGCTTTAATTGGAGATGATCAAATTTATAATGTAATTGTTACAGCTCATGCTTTTATTATAATTTTTTTTATAGTAATGCCAATTATAATTGGAGGATTTGGAAATTGATTAGTTCCATTAATACTAGGAGCTCCAGATATAGCATTCCCTCGAATAAATAATATAAGTTTTTGACTTTTACCTCCTGCATTAACTTTATTATTAGTTAGTAGTATAGTAGAAAACGGAGCTGGAACAGGATGAACAGTTTACCCTCCTCTATCTTCTAATATTGCTCATGGAGGAGCTTCTGTTGATTTAGCTATTTTCTCTCTTCATTTAGCAGGAATTTCTTCAATTTTAGGAGCTGTAAATTTTATTACTACAGTTATTAATATACGATCAACAGGAATTACTTTTGATCGAATACCTTTATTTGTTTGATCAGTAGTAATTACAGCTTTATTACTTTTATTATCATTACCAGTATTAGCAGGAGCTATTACAATACTTTTAACAGACCGAAATCTTAATACATCATTCTTTGATCCTGCAGGAGGAGGAGATCCAATTTTATACCAACATTTATTTTGATTTTTTG

>MH401809

AGAATTCTAATTCGAGCCGAACTAGGACATCCTGGAGCATTAATTGGAGATGACCAAATTTATAATGTAATTGTTACAGCTCATGCTTTTATTATAATTTTTTTTATAGTAATACCAATTATAATTGGAGGATTTGGTAATTGATTAGTCCCTTTAATATTAGGAGCTCCAGATATAGCCTTCCCTCGGATAAACAATATAAGTTTCTGACTTTTACCTCCTGCATTAACTTTACTATTAGTAAGTAGTATAGTAGAAAACGGAGCTGGAACAGGATGAACTGTTTACCCACCTTTATCTTCTAATATTGCTCATGGAGGAGCTTCTGTTGATTTAGCTATTTTTTCTTTACACTTAGCAGGAATTTCTTCAATTTTAGGAGCTGTAAATTTTATTACTACAGTTATTAATATACGATCAACAGGAATTACATTCGACCGAATACCATTATTTGTTTGATCTGTAGTAATTACAGCTTTATTACTTTTATTATCTTTACCAGTATTAGCAGGTGCTATTACTATATTATTAACAGATCGAAATCTTAATACATCATTCTTTGACCCAGCAGGAGGAGGAGACCCAATCTTGTACCAACATTTATTTTGATTCTTTGGCA

>MH401810

AGAATTCTAATTCGAGCCGAACTAGGACATCCTGGAGCATTAATTGGAGATGACCAAATTTATAATGTAATTGTTACAGCTCATGCTTTTATTATAATTTTTTTTATAGTAATACCAATTATAATTGGAGGATTTGGTAATTGATTAGTCCCTTTAATATTAGGAGCTCCAGATATAGCCTTCCCTCGGATAAACAATATAAGTTTCTGACTTTTACCTCCTGCATTAACTTTACTATTAGTAAGTAGTATAGTAGAAAACGGAGCTGGAACAGGATGAACTGTTTACCCACCTTTATCTTCTAATATCGCTCATGGAGGAGCTTCTGTTGATTTAGCTATTTTTTCTTTACACTTAGCAGGAATTTCTTCAATTTTAGGAGCTGTAAATTTTATTACTACAGTTATTAATATACGATCAACAGGAATTACATTCGACCGAATACCATTATTTGTTTGATCTGTAGTAATTACAGCTTTATTACTTTTATTATCTTTACCAGTATTAGCAGGTGCTATTACTATATTATTAACAGATCGAAATCTTAATACATCATTCTTTGACCCAGCAGGAGGAGGAGACCCAATCTTGTACCAACATTTATTTTGATTCTTTGGAACA

>MH401811

AGAATTCTAATTCGAGCCGAACTAGGACATCCTGGAGCATTAATTGGAGATGACCAAATTTATAATGTAATTGTTACAGCTCATGCTTTTATTATAATTTTTTTTATAGTAATACCAATTATAATTGGAGGATTTGGTAATTGATTAGTCCCTTTAATATTAGGAGCTCCAGATATAGCCTTCCCTCGGATAAACAATATAAGTTTCTGACTTTTACCTCCTGCATTAACTTTACTATTAGTAAGTAGTATAGTAGAAAACGGAGCTGGAACAGGATGAACTGTTTACCCACCTTTATCTTCTAATATTGCTCATGGAGGAGCTTCTGTTGATTTAGCTATTTTTTCTTTACACTTAGCAGGAATTTCTTCAATTTTAGGAGCTGTAAATTTTATTACTACAGTTATTAATATACGATCAACAGGAATTACATTCGACCGAATACCATTATTTGTTTGATCTGTAGTAATTACAGCTTTATTACTTTTATTATCTTTACCAGTATTAGCAGGTGCTATTACTATATTATTAACAGATCGAAATCTTAATACATCATTCTTTGACCCAGCAGGAGGAGGAGACCCAATCTTGTACCAACATTTATTTTGATTCTTTGGCC

>MH401812

AGAATTCTAATTCGAGCTGAATTAGGACATCCTGGAGCTTTAATTGGAGATGATCAAATTTATAATGTAATTGTTACAGCTCATGCTTTTATTATAATTTTTTTTATAGTAATGCCAATTATAATTGGAGGATTTGGAAATTGATTAGTTCCATTAATACTAGGAGCTCCAGATATAGCATTCCCTCGAATAAATAATATAAGTTTTTGACTTTTACCTCCTGCATTAACTTTATTATTAGTTAGTAGTATAGTAGAAAACGGAGCTGGAACAGGATGAACAGTTTACCCTCCTCTATCTTCTAATATTGCTCATGGAGGAGCTTCTGTTGATTTAGCTATTTTCTCTCTTCATTTAGCAGGAATTTCTTCAATTTTAGGAGCTGTAAATTTTATTACTACAGTTATTAATATACGATCAACAGGAATTACTTTTGATCGAATACCTTTATTTGTTTGATCAGTAGTAATTACAGCTTTATTACTTTTATTATCATTACCAGTATTAGCAGGAGCTATTACAATACTTTTAACAGACCGAAATCTTAATACATCATTCTTTGACCCTGCAGGAGGAGGAGATCCAATTTTATACCAACATTTATTTTGATTCTTTGGTC

>MH401813

AGAATTCTAATTCGAGCTGAATTAGGACATCCTGGAGCTTTAATTGGAGATGATCAAATTTATAATGTAATTGTTACAGCTCATGCTTTTATTATAATTTTTTTTATAGTAATGCCAATTATAATTGGAGGATTTGGAAATTGATTAGTTCCATTAATACTAGGAGCTCCAGATATAGCATTCCCTCGAATAAATAATATAAGTTTTTGACTTTTACCTCCTGCATTAACTTTATTATTAGTTAGTAGTATAGTAGAAAACGGAGCTGGAACAGGATGAACAGTTTACCCTCCTCTATCTTCTAATATTGCTCATGGAGGAGCTTCTGTTGATTTAGCTATTTTCTCTCTTCATTTAGCAGGAATTTCTTCAATTTTAGGAGCTGTAAATTTTATTACTACAGTTATTAATATACGATCAACAGGAATTACTTTTGATCGAATACCTTTATTTGTTTGATCAGTAGTAATTACAGCTTTATTACTTTTATTATCATTACCAGTATTAGCAGGAGCTATTACAATACTTTTAACAGACCGAAATCTTAATACATCATTCTTTGACCCTGCAGGAGGAGGAGATCCAATTTTATACCAACATTTATTTTGATTTTTTGGA

>MH401814

AGAATTCTAATTCGAGCTGAATTAGGACATCCTGGAGCTTTAATTGGAGATGATCAAATTTACAATGTAATTGTTACAGCTCATGCTTTTATTATAATTTTTTTTATAGTAATGCCAATTATAATTGGAGGATTTGGAAATTGATTAGTTCCATTAATACTAGGAGCTCCAGATATAGCATTCCCTCGAATAAATAATATAAGTTTTTGACTTTTACCTCCTGCATTAACTTTATTATTAGTTAGTAGTATAGTAGAAAACGGAGCTGGAACAGGATGAACAGTTTACCCTCCTCTATCTTCTAATATTGCTCATGGAGGAGCTTCTGTTGATTTAGCTATTTTCTCTCTTCATTTAGCAGGAATTTCTTCAATTTTAGGAGCTGTAAATTTTATTACTACAGTTATTAATATACGATCAACAGGAATTACTTTTGATCGAATACCTTTATTTGTTTGATCAGTAGTAATTACAGCTTTATTACTTTTATTATCATTACCAGTATTAGCGGGAGCTATTACAATACTTTTAACAGACCGAAATCTTAATACATCATTCTTTGACCCTGCAGGAGGAGGAGATCCAATTTTATACCAACATTTATTTTGATTCTTTGGAA

>MH401815

AGAATTCTAATTCGAGCTGAATTAGGACATCCTGGAGCTTTAATTGGAGATGATCAAATTTATAATGTAATTGTTACAGCTCATGCTTTTATTATAATTTTTTTTATAGTAATGCCAATTATAATTGGAGGATTTGGAAATTGATTAGTTCCATTAATACTAGGAGCTCCAGATATAGCATTCCCTCGAATAAATAATATAAGTTTTTGACTTTTACCTCCTGCATTAACTTTATTATTAGTTAGTAGTATAGTAGAAAACGGAGCTGGAACAGGATGAACAGTTTACCCTCCTCTATCTTCTAATATTGCTCATGGAGGAGCTTCTGTTGATTTAGCTATTTTCTCTCTTCATTTAGCAGGAATTTCTTCAATTTTAGGAGCTGTAAATTTTATTACTACAGTTATTAATATACGATCAACAGGAATTACTTTTGATCGAATACCTTTATTTGTTTGATCAGTAGTAATTACAGCTTTATTACTTTTATTATCATTACCAGTATTAGCAGGAGCTATTACAATACTTTTAACAGACCGAAATCTTAATACATCATTCTTTGACCCTGCAGGAGGAGGAGATCCAATTTTATACCAACATTTATTTTGATTCTTTGGCACA

>MH401816

AGAATTCTAATTCGAGCTGAATTAGGACACCCTGGTGCACTAATTGGAGATGACCAAATTTATAATGTAATTGTTACAGCTCATGCTTTTATTATAATTTTCTTTATAGTAATACCAATTATAATTGGAGGATTTGGTAATTGATTAGTTCCTTTAATGTTAGGAGCCCCAGATATAGCCTTCCCTCGATTAAATAATATAAGATTTTGACTTTTACCTCCTGCACTAACTTTATTATTAGTAAGTAGTATAGTAGAAAACGGAGCTGGAACAGGATGAACAGTTTACCCCCCTTTATCATCTAATATCGCTCATGGAGGAGCTTCTGTTGATTTAGCTATTTTTTCTCTTCATTTAGCAGGAATTTCTTCAATTTTAGGGGCTGTAAATTTTATTACTACAGTCATTAATATACGATCAACAGGAATTACTTTTGACCGAATACCTTTATTTGTTTGATCAGTTGTAATTACAGCTCTATTACTTTTATTATCATTACCAGTATTAGCAGGAGCTATTACAATACTTTTAACTGACCGAAACCTTAATACTTCATTTTTTGACCCTGCAGGAGGAGGAGACCCAATTCTATACCAACATCTATTTTGATTCTTTG

>MH401817

AGTATCTTAATCCGAGCAGAGCTAGGACATCCAGGAGCTTTAATTGGTGATGATCAAATTTATAATGTAATTGTCACCGCCCATGCTTTTGTCATAATTTTTTTTATAGTAATACCAATTATAATTGGGGGATTTGGAAATTGATTAGTTCCTTTAATACTAGGAGCTCCTGATATAGCTTTCCCACGAATAAATAATATAAGTTTTTGAATATTACCTCCTTCTCTTACACTACTATTAGTAAGAAGTATAGTAGAAAACGGAGCTGGTACAGGTTGAACAGTTTACCCTCCTCTTTCTTCTGTTATTGCTCATGGAGGGGCTTCTGTTGATTTAGCTATTTTTTCCCTTCACCTAGCAGGAATTTCTTCAATTTTAGGAGCTGTAAATTTTATCACAACTGTAATTAATATACGATCTACAGGTATCACTTTTGATCGAATACCTTTATTTGTTTGATCTGTTGTAATTACAGCTTTATTATTACTTTTATCTCTACCTGTTCTAGCAGGAGCAATTACAATATTATTAACAGATCGAAATTTAAATACTTCATTCTTTGACCCAGCAGGAGGAGGAGACCCAATTCTTTACCAACATTTATTTTGATTCTTTGGC

>MH401818

AGAATTCTAATTCGAGCCGAACTAGGGCATCCTGGAGCATTAATTGGAGATGACCAAATTTATAATGTAATTGTTACAGCTCATGCTTTTATTATAATTTTTTTTATAGTAATACCAATTATAATTGGAGGATTTGGTAATTGATTAGTCCCTTTAATATTAGGAGCTCCAGACATAGCCTTCCCTCGGATAAACAATATAAGTTTCTGACTTTTACCTCCTGCATTAACTTTACTATTAGTAAGTAGTATAGTAGAAAACGGAGCTGGAACAGGATGAACTGTTTACCCACCTTTATCTTCTAATATCGCTCATGGAGGAGCTTCTGTTGATTTAGCTATTTTTTCTTTACACTTGGCAGGAATTTCTTCAATTTTAGGAGCTGTAAATTTTATTACTACAGTTATTAATATACGATCAACAGGAATTACATTCGACCGAATACCATTATTTGTTTGATCTGTAGTAATTACAGCTTTATTACTTTTATTATCTTTACCAGTATTAGCAGGTGCTATTACTATATTATTAACAGATCGAAATCTTAATACATCATTCTTTGACCCAGCAGGAGGAGGAGACCCAATCTTGTACCAACATTTATTTTGATTTTTTGGA

>MH401819

AGAATTCTAATTCGAGCCGAACTAGGACATCCTGGAGCATTAATTGGAGATGACCAAATTTATAATGTAATTGTTACAGCTCATGCTTTTATTATAATTTTTTTTATAGTAATACCAATTATAATTGGAGGATTTGGTAATTGATTAGTCCCTTTAATATTAGGAGCTCCAGATATAGCCTTCCCTCGGATAAACAATATAAGTTTCTGACTTTTACCTCCTGCATTAACTTTACTATTAGTAAGTAGTATAGTAGAAAACGGAGCTGGAACAGGATGAACTGTTTACCCACCTTTATCTTCTAATATCGCTCATGGAGGAGCTTCTGTTGATTTAGCTATTTTTTCTTTACACTTAGCAGGAATTTCTTCAATTTTAGGAGCTGTAAATTTTATTACTACAGTTATTAATATACGATCAACAGGAATTACATTCGACCGAATACCATTATTTGTTTGATCTGTAGTAATTACAGCTTTATTACTTTTATTATCTTTACCAGTATTAGCAGGTGCTATTACTATATTATTAACAGATCGAAATCTTAATACATCATTCTTTGACCCAGCAGGAGGAGGAGACCCAATCTTGTACCAACATTTATTTTGATTCTTTGGCCA

>MH401820

AGAATTCTAATTCGAGCCGAACTAGGACATCCTGGAGCATTAATTGGAGATGACCAAATTTATAATGTAATTGTTACAGCTCATGCTTTTATTATAATTTTTTTTATAGTAATACCAATTATAATTGGAGGATTTGGTAATTGATTAGTCCCTTTAATATTAGGAGCTCCAGATATAGCCTTCCCTCGGATAAACAATATAAGTTTCTGACTTTTACCTCCTGCATTAACTTTACTATTAGTAAGTAGTATAGTAGAAAACGGAGCTGGAACAGGATGAACTGTTTACCCACCTTTATCTTCTAATATCGCTCATGGAGGAGCTTCTGTTGATTTAGCTATTTTTTCTTTACACTTAGCAGGAATTTCTTCAATTTTAGGGGCTGTAAATTTTATTACTACAGTTATTAATATACGATCAACAGGAATTACATTCGACCGAATACCATTATTTGTTTGATCTGTAGTAATTACAGCTTTATTACTTTTATTATCTTTACCAGTATTAGCAGGTGCTATTACTATATTATTAACAGATCGAAATCTTAATACATCATTCTTTGACCCAGCAGGAGGAGGAGACCCAATCTTGTACCAACATTTATTTTGATTCTTTGGC

>MH401821

AGAATTTTAATTCGAGCTGAACTAGGGCATCCTGGAGCATTAATTGGAGATGATCAAATTTATAATGTAATTGTTACAGCTCATGCTTTTATTATAATTTTTTTTATAGTAATACCAATTATAATTGGAGGGTTTGGAAATTGATTAGTTCCTTTAATATTAGGAGCCCCAGATATAGCATTCCCTCGAATAAATAATATAAGTTTCTGACTTTTACCTCCTGCATTAACTTTACTATTAGTAAGTAGTATAGTAGAAAACGGAGCTGGAACTGGATGAACTGTTTATCCACCTTTATCTTCTAATATTGCACATGGAGGAGCTTCTGTTGATTTAGCTATTTTTTCTTTACATTTAGCAGGAATTTCTTCAATTTTAGGAGCTGTAAATTTTATTACTACAGTTATTAATATACGATCAACAGGTATTACCTTCGACCGAATACCATTATTTGTTTGATCAGTAGTAATTACAGCCTTATTACTTTTATTATCTTTACCAGTATTAGCAGGAGCTATTACTATATTATTAACAGATCGAAATCTTAATACTTCATTCTTTGACCCAGCAGGAGGAGGAGATCCAATTTTATACCAACACTTATTTTGATTCTTTG

>MH401822

AGAATTTTAATTCGAGCTGAACTAGGGCATCCTGGAGCATTAATTGGAGATGATCAAATTTATAATGTAATTGTTACAGCTCATGCTTTTATTATAATTTTTTTTATAGTAATACCAATTATAATTGGAGGGTTTGGAAATTGATTAGTTCCTTTAATATTAGGAGCCCCAGATATAGCATTCCCTCGAATAAATAATATAAGTTTCTGACTTTTACCTCCTGCATTAACTTTACTATTAGTAAGTAGTATAGTAGAAAACGGAGCTGGAACTGGATGAACTGTTTATCCACCTTTATCTTCTAATATTGCACATGGAGGAGCTTCTGTTGATTTAGCTATTTTTTCTTTACATTTAGCAGGAATTTCTTCAATTTTAGGAGCTGTAAATTTTATTACTACAGTTATTAATATACGATCAACAGGTATTACCTTCGACCGAATACCATTATTTGTTTGATCAGTAGTAATTACAGCCTTATTACTTTTATTATCTTTACCAGTATTAGCAGGAGCTATTACTATATTATTAACAGATCGAAATCTTAATACTTCATTCTTTGACCCAGCAGGAGGAGGAGATCCAATTTTATACCAACACTTATTTTGATTTTTTGGC

>MH401823

AGAATTTTAATTCGAGCTGAACTAGGGCATCCTGGAGCATTAATTGGAGATGATCAAATTTATAATGTAATTGTTACAGCTCATGCTTTTATTATAATTTTTTTTATAGTAATACCAATTATAATTGGAGGGTTTGGAAATTGATTAGTTCCTTTAATATTAGGAGCCCCAGATATAGCATTCCCTCGAATAAATAATATAAGTTTCTGACTTTTACCTCCTGCATTAACTTTACTATTAGTAAGTAGTATAGTAGAAAACGGAGCTGGAACTGGATGAACTGTTTATCCACCTTTATCTTCTAATATTGCACATGGAGGAGCTTCTGTTGATTTAGCTATTTTTTCTTTACATTTAGCAGGAATTTCTTCAATTTTAGGAGCTGTAAATTTTATTACTACAGTTATTAATATACGATCAACAGGTATTACCTTCGACCGAATACCATTATTTGTTTGATCAGTAGTAATTACAGCCTTATTACTTTTATTATCTTTACCAGTATTAGCAGGAGCTATTACTATATTATTAACAGATCGAAATCTTAATACTTCATTCTTTGACCCAGCAGGAGGAGGAGATCCAATTTTATACCAACACTTATTTTGATTCTTTGGCC

>MH401824

AGAATTTTAATTCGAGCTGAATTAGGACACCCTGGTGCATTAATTGGAGATGACCAAATTTATAATGTAATTGTTACAGCTCATGCTTTTATTATAATTTTCTTTATAGTAATACCAATTATAATTGGAGGATTTGGAAATTGATTAGTTCCTTTAATATTAGGAGCCCCAGATATGGCATTCCCTCGAATAAATAATATAAGATTTTGACTTTTACCTCCTGCATTAACTTTATTATTAGTAAGTAGTATAGTAGAAAACGGAGCTGGAACAGGATGAACAGTTTACCCTCCTTTATCATCTAATATCGCTCATGGAGGAGCTTCTGTTGATTTAGCTATTTTTTCTCTTCATTTAGCAGGAATCTCATCAATTTTAGGGGCTGTAAATTTTATTACTACAGTTATTAATATACGATCAACAGGAATTACTTTTGACCGAATACCTTTATTCGTTTGATCAGTAGTAATTACAGCTTTATTACTTTTATTATCATTACCAGTATTAGCTGGAGCTATTACTATACTTTTAACTGACCGAAATCTTAATACTTCATTCTTTGACCCAGCAGGAGGAGGAGACCCAATTTTATACCAACATTTATTTTGATTCTTTGGC

>MH401825

AGAATTTTAATTCGAGCTGAATTAGGACACCCTGGTGCATTAATTGGAGATGACCAAATTTATAATGTAATTGTTACAGCTCATGCTTTTATTATAATTTTCTTTATAGTAATACCAATTATAATTGGAGGATTTGGAAATTGATTAGTTCCTTTAATATTAGGAGCCCCAGATATAGCATTCCCTCGAATAAATAATATAAGTTTTTGACTTTTACCTCCTGCATTAACTTTATTATTAGTAAGTAGTATAGTAGAAAACGGAGCTGGAACAGGATGAACAGTTTACCCTCCTTTATCATCTAATATCGCTCATGGAGGAGCTTCTGTTGATTTAGCTATTTTTTCTCTTCATTTAGCAGGAATTTCATCAATTTTAGGAGCTGTAAATTTTATTACTACAGTTATTAATATACGATCAACAGGAATTACTTTTGACCGAATACCTTTATTCGTTTGATCAGTAGTAATTACAGCTTTATTACTTTTATTATCATTACCAGTATTAGCTGGAGCTATTACTATACTTTTAACTGATCGAAATCTTAATACTTCATTCTTTGATCCAGCAGGAGGAGGAGACCCAATTTTATACCAACATTTATTTTGATTCTTTGGACACA

>MH401826

AGAATTTTAATTCGAGCTGAATTAGGACACCCTGGTGCATTAATTGGAGATGACCAAATTTATAATGTAATTGTTACAGCTCATGCTTTTATTATAATTTTCTTTATAGTAATACCAATTATAATTGGAGGATTTGGAAATTGATTAGTTCCTTTAATATTAGGAGCCCCAGATATAGCATTCCCTCGAATAAATAATATAAGATTTTGACTTTTACCTCCTGCATTAACTTTATTGTTAGTAAGTAGTATAGTAGAAAACGGAGCTGGAACAGGATGAACAGTTTACCCTCCTTTATCATCTAATATCGCTCATGGAGGAGCTTCTGTTGATTTAGCTATTTTTTCTCTTCATTTAGCAGGAATTTCATCAATTTTAGGAGCTGTAAATTTTATTACTACAGTTATTAATATACGATCAACAGGAATTACTTTTGACCGAATACCTTTATTCGTTTGATCAGTAGTAATTACAGCTTTATTACTTTTATTATCATTACCAGTATTAGCTGGAGCTATTACTATACTTTTAACTGATCGAAATCTTAATACTTCATTCTTTGACCCAGCAGGAGGAGGAGACCCAATTTTATACCAACATTTATTTTGATTCTTTGGAA

>MH401827

AGAATTTTAATTCGAGCTGAATTAGGACACCCTGGTGCATTAATTGGAGATGACCAAATTTATAATGTAATTGTTACAGCTCATGCTTTTATTATAATTTTCTTTATAGTAATGCCAATTATAATTGGAGGATTTGGAAATTGATTAGTTCCTTTAATATTAGGAGCTCCAGATATAGCATTCCCTCGAATAAATAATATAAGATTTTGACTTTTACCTCCTGCATTAACTTTACTATTAGTAAGTAGTATAGTAGAAAACGGAGCTGGAACAGGATGAACAGTTTACCCTCCTTTATCATCTAATATCGCTCATGGAGGAGCTTCTGTTGATTTAGCTATTTTTTCTCTTCATTTAGCAGGAATTTCATCAATTTTAGGAGCTGTAAATTTTATTACTACAGTTATTAATATACGATCAACAGGGATTACTTTTGATCGAATACCTTTATTCGTTTGATCAGTAGTAATTACAGCTTTATTACTTTTATTATCATTACCAGTATTAGCTGGAGCTATTACTATACTTTTAACTGATCGAAATCTTAATACTTCATTCTTTGATCCAGCAGGAGGAGGAGACCCAATTTTATACCAACATTTATTTTGATTCTTTGGTC

>MH401828

AGAATTCTAATTCGAGCTGAATTAGGACATCCTGGAGCTTTAATTGGAGATGATCAAATTTATAATGTAATTGTTACAGCTCATGCTTTTATTATAATTTTTTTTATAGTAATGCCAATTATAATTGGAGGATTTGGAAATTGATTAGTTCCATTAATACTAGGAGCTCCAGATATAGCATTCCCTCGAATAAATAATATAAGTTTTTGACTTTTACCTCCTGCATTAACTTTATTATTAGTTAGTAGTATAGTAGAAAACGGAGCTGGAACAGGATGAACAGTTTACCCTCCTCTATCTTCTAATATTGCTCATGGAGGAGCTTCTGTTGATTTAGCTATTTTCTCTCTTCATTTAGCAGGAATTTCTTCAATTTTAGGAGCTGTAAATTTTATTACTACAGTTATTAATATACGATCAACAGGAATTACTTTTGATCGAATACCTTTATTTGTTTGATCAGTAGTAATTACAGCTTTATTACTTTTATTATCATTACCAGTATTAGCAGGAGCTATTACAATACTTTTAACAGACCGAAATCTTAATACATCATTCTTTGACCCTGCAGGAGGAGGAGATCCAATTTTATACCAACATTTATTTTGATTCTTTGGC

>MH401829

AGAATTCTAATTCGAGCTGAATTAGGACATCCTGGAGCTTTAATTGGAGATGATCAAATTTATAATGTAATTGTTACAGCTCATGCTTTTATTATAATTTTTTTTATGGTAATGCCAATTATAATTGGAGGATTTGGAAATTGATTAGTTCCATTAATACTAGGAGCTCCAGATATAGCATTCCCTCGAATAAATAATATAAGTTTTTGACTTTTACCTCCTGCATTAACTTTATTATTAGTTAGTAGTATAGTAGAAAACGGAGCTGGAACAGGATGAACAGTTTACCCTCCTCTATCTTCTAATATTGCTCATGGAGGAGCTTCTGTTGATTTAGCTATTTTCTCTCTTCATTTAGCAGGAATTTCTTCAATTTTAGGAGCTGTAAATTTTATTACTACAGTTATTAATATACGATCAACAGGAATTACTTTTGATCGAATACCTTTATTTGTTTGATCAGTAGTAATTACAGCTTTATTACTTTTATTATCATTACCAGTATTAGCAGGAGCTATTACAATACTTTTAACAGACCGAAATCTTAATACATCATTCTTTGACCCTGCAGGAGGAGGAGATCCAATTTTATACCAACATTTATTTTGATTCTTTGA

>MH401830

AGAATTCTAATTCGAGCTGAATTAGGACATCCTGGAGCTTTAATTGGAGATGATCAAATTTATAATGTAATTGTTACAGCTCATGCTTTTATTATAATTTTTTTTATAGTAATGCCAATTATAATTGGAGGATTTGGAAATTGATTAGTTCCATTAATACTAGGAGCTCCAGATATAGCATTCCCTCGAATAAATAATATAAGTTTTTGACTTTTACCTCCTGCATTAACTTTATTATTAGTTAGTAGTATAGTAGAAAACGGAGCTGGAACAGGATGAACAGTTTACCCTCCTCTATCTTCTAATATTGCTCATGGAGGAGCTTCTGTTGATTTAGCTATTTTCTCTCTTCATTTAGCAGGAATTTCTTCAATTTTAGGAGCTGTAAATTTTATTACTACAGTTATTAATATACGATCAACAGGAATTACTTTTGATCGAATACCTTTATTTGTTTGATCAGTAGTAATTACAGCTTTATTACTTTTATTATCATTACCAGTATTAGCAGGAGCTATTACAATACTTTTAACAGACCGAAATCTTAATACATCATTCTTTGACCCTGCAGGAGGAGGAGATCCAATTTTATACCAACATTTATTTTGATTCTTTGGA

>MH401831

AGAATTCTTATTCGAGCAGAATTAGGTCACCCTGGTGCATTAATTGGTGATGATCAAATTTATAATGTAATTGTTACAGCCCATGCTTTCATTATAATTTTTTTTATAGTAATACCAATTATAATTGGAGGATTTGGAAATTGATTAGTGCCAATTATACTAGGAGCTCCAGATATAGCCTTCCCTCGGATAAACAATATAAGTTTTTGACTTTTACCTCCTGCATTAACATTGCTTCTAGTAAGTAGTATAGTAGAAAATGGAGCTGGAACAGGTTGAACTGTATACCCTCCTTTATCTTCTAATATTGCTCATGGAGGAGCTTCTGTTGATTTAGCTATTTTTTCTCTCCATTTAGCTGGAATTTCTTCAATTCTAGGAGCAGTAAATTTTATTACTACAGTTATTAATATACGATCAACAGGAATCACTTTTGATCGAATACCTTTATTTGTATGATCTGTAGTAATCACAGCCCTACTTTTATTACTTTCTTTACCTGTACTTGCCGGAGCTATTACTATATTATTAACTGATCGAAATATTAATACTTCATTTTTTGACCCGGCAGGAGGAGGAGATCCTATTCTATATCAACATTTATTTTGATTCTTTGGC

>MH401832

AGAATTTTAATTCGAGCTGAACTAGGGCATCCTGGAGCATTAATTGGAGATGACCAAATTTATAATGTAATTGTTACAGCTCATGCTTTTATTATAATTTTTTTTATAGTAATACCAATTATAATTGGAGGGTTTGGAAATTGATTAGTTCCTTTAATATTAGGAGCCCCAGATATAGCATTCCCTCGAATAAATAATATAAGTTTCTGACTTTTACCTCCTGCATTAACTTTATTATTAGTAAGTAGTATAGTAGAAAACGGAGCTGGAACTGGATGAACTGTTTATCCACCTTTATCTTCTAATATTGCACATGGAGGAGCTTCTGTTGATTTAGCTATTTTTTCTTTACATTTAGCAGGAATTTCTTCAATTTTAGGAGCTGTAAATTTTATTACTACAGTTATTAATATACGATCAACAGGTATTACCTTCGACCGAATACCATTATTTGTTTGATCAGTAGTAATTACAGCCTTATTACTTTTATTATCTTTACCAGTATTAGCAGGAGCTATTACTATATTATTAACAGATCGAAATCTTAATACTTCATTCTTTGACCCAGCAGGAGGAGGAGATCCAATTTTATACCAACACTTATTTTGATTCTTTGTC

>MH401833

AGAATTCTAATTCGAGCTGAATTAGGACATCCTGGAGCACTAATTGGAGATGACCAAATTTATAATGTAATTGTAACAGCTCATGCCTTTATTATAATTTTCTTTATAGTAATACCAATTATAATTGGAGGATTTGGAAATTGACTAGTTCCTTTAATATTAGGAGCCCCAGATATAGCTTTCCCACGAATAAATAATATAAGTTTCTGACTTTTACCTCCTGCATTAACTTTACTATTAGTAAGTAGTATAGTAGAAAATGGAGCTGGAACAGGATGAACTGTTTATCCACCTTTATCATCTAATATTGCTCATGGTGGAGCATCAGTTGATTTAGCTATTTTTTCTTTACACTTAGCTGGAATTTCATCAATTTTAGGAGCTGTAAATTTTATTACAACTGTTATTAATATACGATCTACAGGAATCACATTTGATCGAATACCTTTATTCGTATGATCTGTAGTTATTACTGCTCTTCTTTTATTATTATCATTACCAGTATTAGCCGGTGCAATTACTATATTATTAACTGATCGAAATTTAAATACTTCATTCTTTGATCCAGCAGGAGGAGGAGATCCTATTTTATATCAACATTTATTTTGATTTTTTGG

>MH401834

AGAATTCTAATTCGAGCCGAACTAGGACATCCTGGAGCATTAATTGGAGATGACCAAATTTATAATGTAATTGTTACAGCTCATGCTTTTATTATAATTTTTTTTATAGTAATACCAATTATAATTGGAGGATTTGGTAATTGATTAGTCCCTTTAATATTAGGAGCTCCAGATATAGCCTTCCCTCGGATAAACAATATAAGTTTCTGACTTTTACCTCCTGCATTAACTTTACTATTAGTAAGTAGTATAGTAGAAAACGGAGCTGGAACAGGATGAACTGTTTACCCACCTTTATCTTCTAATATCGCTCATGGAGGAGCTTCTGTTGATTTAGCTATTTTTTCTTTACACTTAGCAGGAATTTCTTCAATTTTAGGAGCTGTAAATTTTATTACTACAGTTATTAATATACGATCAACAGGAATTACATTCGACCGAATACCATTATTTGTTTGATCTGTAGTAATTACAGCTTTATTACTTTTATTATCTTTACCAGTATTAGCAGGTGCTATTACTATATTATTAACAGATCGAAATCTTAATACATCATTCTTTGACCCAGCAGGAGGAGGAGACCCAATCTTGTACCAACATTTATTTTGATTTTTGGCCC

>MH401835

AGTATTCTAATTCGAGCTGAATTAGGACATCCAGGAGCACTAATTGGAGATGATCAAATTTATAATGTAATTGTAACAGCTCATGCTTTTATTATAATTTTTTTCATAGTAATACCTATTATAATTGGAGGATTTGGTAATTGATTAGTTCCACTTATACTAGGAGCTCCTGATATAGCATTTCCTCGAATAAACAATATAAGCTTTTGATTATTACCTCCAGCATTAACTTTACTTCTGGTAAGTAGTATAGTAGAAAATGGAGCTGGTACAGGATGAACTGTCTACCCTCCACTTTCATCTAATATCGCACATGGAGGAGCTTCAGTTGATTTAGCAATTTTCTCTCTTCATTTAGCTGGAATTTCATCTATTCTAGGAGCTGTAAATTTTATTACTACTGTAATTAATATACGATCAACTGGAATTACCTTAGATCGAATACCTTTATTTGTTTGATCTGTAGTAATTACAGCACTATTACTTCTTTTATCTTTACCAGTCTTAGCTGGAGCAATTACTATATTATTAACAGATCGTAATTTAAATACCTCATTTTTTGATCCAGCAGGAGGTGGTGATCCAATTTTATATCAACATTTATTTTGATTCTTTGGACATCAGG

>MH401836

AGTATTCTAATTCGAGCTGAATTAGGACATCCAGGAGCACTAATTGGAGATGATCAAATTTATAATGTAATTGTAACAGCTCATGCTTTTATTATAATTTTTTTCATAGTAATACCTATTATAATTGGAGGATTTGGTAATTGATTAGTTCCACTTATACTAGGAGCTCCTGATATAGCATTTCCTCGAATAAACAATATAAGCTTTTGATTATTACCTCCAGCATTAACTTTACTTCTGGTAAGTAGTATAGTAGAAAATGGAGCTGGTACAGGATGAACTGTCTACCCTCCACTTTCATCTAATATCGCACATGGAGGAGCTTCAGTTGATTTAGCAATTTTCTCTCTTCATTTAGCTGGAATTTCATCTATTCTAGGAGCTGTAAATTTTATTACTACTGTAATTAATATACGATCAACTGGAATTACCTTAGATCGAATACCTTTATTTGTTTGATCTGTAGTAATTACAGCACTATTACTTCTTTTATCTTTACCAGTCTTAGCTGGAGCAATTACTATATTATTAACAGATCGTAATTTAAATACCTCATTTTTTGATCCAGCAGGAGGTGGTGATCCAATTTTATATCAACATTTATTTTGATTCTTTGG

**ND5 sequences**

>MH401883

GATAATTAATAATTCCATAAGTAGAAATATAAGGTATAAACCACATAGAACCTA

AAAAATAAGAAAAATTATAATTATTTAAAGCCTTATTATAAAAAAATAAAGAAACATGAG

AAATTATATAACCTATAATACCCCCAACAATACAAACAAATAATGTTAATAATTTTAAAT

AAGAAGGAAGAACAACTACTATAGGAGTAGGAAAAATTAATCATCTTAACATTCTCCCAC

CAAAAATTCTTAAAATTAATAAACCTATTATACTTTTCAATATTACTCAACCTTCATCAT

TTAATATATTTAAAGAAGAAAAATTAGAATCACCA

>MH401884

AATAATTAATAATTCCATATGTAGAAATATAAGGTATAAATCAT

ATTGAACCTAAAAAGTAAGAAGAATTATAATTATTTAAAGCCTTATTATAAAAAAATAAA

GAAATATTAGAAATTAAATAACCTCTAACTCCTCCTACAATACAAACAAATAAAGTTAAT

AATTTTAAATAAGAAGGAAGAACTACTACTACTGGTGTAGGAAAAATTAATCAACTTAAT

ATACTACCCCCAAAAATTCTCAAAATTAATAGTCCTATTATACTTTTTAATATAACTCAA

CCTTCATCATTTAGTATATTTAAAGAAGAAAAATTTGAATCCCCA

>MH401885

AATAATTAATAATTCCATATGTAGAAATATAAGGTATAAATCATAT

TGAACCTAAAAAGTAAGAAGAATTATAATTATTTAAAGCCTTATTATAAAAAAATAAAGA

AATATTAGAAATTAAATAACCTCTAACTCCTCCTACAATACAAACAAATAAAGTTAATAA

TTTTAAATAAGAAGGAAGAACTACTACTACTGGTGTAGGAAAAATTAATCAACTTAATAT

ACTACCCCCAAAAATTCTCAAAATTAATAGTCCTATTATACTTTTTAATATAACTCAACC

TTCATCATTTAGTATATTTAAAGAAGAAAAATTTGAATCCCCA

>MH401886

AATAATTGATAATTCCATATGTAGAAATATAAGGTATAAATCATATT

GAACCTAAAAAGTAAGAAGAATTATAATTATTTAAAGCCTTATTATAAAAAAATAAAGAA

ATATTAGAAATTAAATAACCTCTAACTCCTCCTACAATACAAACAAATAAAGTTAATAAT

TTTAAATAAGAAGGAAGAACTACTACTACTGGTGTAGGAAAAATTAATCAACTTAATATA

CTACCCCCAAAAATTCTCAAAATTAATAGTCCTATTATACTTTTTAATATAACTCAACCT

TCATCATTTAGTATATTTAAAGAAGAAAAATTTGAATCCCCA

>MH401887

AATAATTAATAATTCCATATGTAGAAATATAAGGTATAAATCATATTGAAC

CTAAAAAGTAAGAAGAATTATAATTATTTAAAGCCTTATTATAAAAAAATAAAGAAATAT

TAGAAATTAAATAACCTCTAACTCCTCCTACAATACAAACAAATAAAGTTAATAATTTTA

AATAAGAAGGAAGAACTACTACTACTGGTGTAGGAAAAATTAATCAACTTAATATACTAC

CCCCAAAAATTCTCAAAATTAATAGTCCTATTATACTTTTTAATATAACTCAACCTTCAT

CATTTAGTATATTTAAAGAAGAAAAATTTGAATCCCCA

>MH401888

AATAATTAATAATTCCATATGTAGAAATATAAGGTATAAATCATATTGAAC

CTAAAAAATAAGAAGAATTATAATTATTTAAAGCCTTATTATAAAAAAATAAAGAAATAT

TAGAAATTAAATAACCTCTAACTCCTCCTACAATACAAACAAATAAAGTTAATAATTTTA

AATAAGAAGGAAGAACTACTACTACTGGTGTAGGAAAAATTAATCAACTTAATATACTAC

CCCCAAAAATTCTCAAAATTAATAGTCCTATTATACTTTTTAATATAACTCAACCTTCAT

CATTTAGTATATTTAAAGAAGAAAAATTTGAATCCCCA

>MH401889

AATAATTAATAATTCCATAAGTAGAAATATAAGGTATAAACCAC

ATAGAACCTAAAAAATAAGAAAAATTATAATTATTTAAAGCCTTGTTATAAAAAAATAAA

GAAACATTAGAAATTATATAACCTATAATACCCCCAACAATACAAACAAATAATGTTAAT

AATTTTAAATAAGAAGGAAGAACAATTACTATAGGAGTAGGAAAAATCAATCATCTTAAC

ATTCTCCCACCAAAAATTCTTAAAATTAATAAACCTATTATACTTTTCAATATTACTCAA

CCTTCATCATTTAATATATTTAAAGAAGAAAAATTAGAATCACCA

>MH401890

AATAATTAATAATTCCATAAGTAGAAATATAAGGTATAAACCACATAGAACCT

AAAAAATAAGAAAAATTATAATTATTTAAAGCCTTATTATAAAAAAATAAAGAAACATTA

GAAATTATATAACCTATAATACCCCCAACAATACAAACAAATAATGTTAATAATTTTAAA

TAAGAAGGAAGAACAACTACTATAGGAGTAGGAAAAATCAATCATCTTAACATTCTCCCA

CCAAAAATTCTTAAAATTAATAAACCTATTATACTTTTCAATATTACTCAACCTTCATCA

TTTAATATATTTAAAGAAGAAAAATTAGAATCACCA

>MH401891

AATAATTAATAATTCCATAAGTAGAAATATATGGTATAAACCACAT

AGAACCTAAAAAATAAGAAAAATTATAATTATTTAAAGCCTTATTATAAAAAAATAAAGA

AACATTAGAAATTATATAACCTATAATACCCCCAACAATACAAACAAATAATGTTAATAA

TTTTAAATAAGAAGGAAGAACAACTACTATAGGAGTAGGAAAAATCAATCATCTTAACAT

TCTCCCACCAAAAATTCTTAAAATTAATAAACCTATTATACTTTTCAATATTACTCAACC

TTCATCATTTAATATATTTAAAGAAGAAAAATTAGAATCACCA

>MH401892

AATAATTAATAATTCCATAAGTAGAAATATAAGGTATAAACCA

CATAGAACCTAAAAAATAAGAAAAATTATAATTATTTAAAGCCTTATTATAAAAAAATAA

AGAAACATTAGAAATTATATAACCTATAATACCCCCAACAATACAAACAAATAATGTTAA

TAATTTTAAATAAGAAGGAAGAACAACTACTATAGGAGTAGGAAAAATCAATCATCTTAA

CATTCTCCCACCAAAAATTCTTAAAATTAATAAACCTATTATACTTTTCAATATTACTCA

ACCTTCATCATTTAATATATTTAAAGAAGAAAAATTAGAATCACCA

>MH401893

AATAATTAATAATTCCGTATGTAGAAATATAAGGCATAAACCACAT

TGACCCTAAAAAATAAGAAGAATTATAGTTATTTAAAGCCTTATTATAAAAAAATAAAGA

AATATTAGAAATTAAATACCCTCTAATTCCTCCTACAATACAAACAAATAAAGTTAATAA

CTTTAAATAAGAAGGTAGAACTACTACTACTGGTGTAGGAAAAATTAATCAACTTAACAT

TCTACCCCCAAAAATTCTTAAAATTAATAACCCTATTATACTCTTTAACATAACTCAACC

TTCATCATTTAATATATTTAAAGAAGAAAAATTTGAATCCCCA

>MH401894

AATAATTAATAATTCCATAAGTAGAAATATAAGGTATAAACCACATAGA

ACCTAAAAAATAAGAAAAATTATAATTATTTAAAGCCTTATTATAAAAAAATAAAGAAAC

ATTAGAAATTATATAACCTATAATACCCCCAACAATACAAACAAATAATGTTAATAATTT

TAAATAAGAAGGAAGAACAACTACTATAGGAGTAGGAAAAATCAATCATCTTAACATTCT

CCCACCAAAAATTCTTAAAATTAATAAACCTATTATACTTTTCAATATTACTCAACCTTC

ATCATTTAATATATTTAAAGAAGAAAAATTAGAATCACCA

>MH401895

AATAATTAATAATTCCATAAGTAGAAATATAAGGTATAAACCACATAGAACC

TAAAAAATAAGAAAAATTATAATTATTTAAAGCCTTATTATAAAAAAATAAAGAAACATT

AGAAATTATATAACCTATAATACCCCCAACAATACAAACAAATAATGTTAATAATTTTAA

ATAAGAAGGAAGAACAACTACTATAGGAGTAGGAAAAATCAATCATCTTAACATTCTCCC

ACCAAAAATTCTTAAAATTAATAAACCTATTATACTTTTCAATATTACTCAACCTTCATC

ATTTAATATATTTAAAGAAGAAAAATTAGAATCACCA

>MH401896

AATAATTAATAATTCCATAAGTAGAAATATAAGGTATAAACCACATAGAACC

TAAAAAATAAGAAAAATTATAATTATTTAAAGCCTTATTATAAAAAAATAAAGAAACATT

AGAAATTATATAACCTATAATACCCCCAACAATACAAACAAATAATGTTAATAATTTTAA

ATAAGAAGGAAGAACAACTACTATAGGAGTAGGAAAAATCAATCATCTTAACATTCTCCC

ACCAAAAATTCTTAAAATTAATAAACCTATTATACTTTTTAATATTACTCAACCTTCATC

ATTTAATATATTTAAAGAAGAAAAATTAGAATCACCA

>MH588572

AATAATTAATAATCCCGTAAGTAGAAATATAAGGTATAAACCATAT

TGACCCTAAAAAATAAGAAAAATTATAATTATTTAAAGCCTTATTATAAAAAAATAAAGA

AATATTTGAAATTATATACCCTCTTACTCCTCCTACAATACAAACAAATAATGTTAATAA

CTTCAAATATGAAGGAAGAACTACAACTACTGGAGTTGGAAAAATTAATCATCTTAATAT

TCTTCCTCCAAAAATTCTTAAAATTAATAACCCTATTATACTCTTTAATATTACTCAACC

TTCATCATTTAATATATTTAAAGAAGAAAAATTTGAATCACCA

>MH401898

AGTAATTAATAATTCCGTATGTAGAAATATAAGGCATAAACCACA

TTGACCCTAAAAAATAAGAAGAATTATAGTTATTTAAAGCCTTATTATAAAAAAATAAAG

AAATATTAGAAATTAAATACCCTCTAATTCCTCCTACAATACAAACAAATAAAGTTAATA

ACTTTAAATAAGAAGGTAGAACTACTACTACTGGTGTAGGAAAAATTAATCAACTTAACA

TTCTACCCCCAAAAATTCTTAAAATTAATAACCCTATTATACTCTTTAACATAACTCAAC

CTTCATCATTTAATATATTTAAAGAAGAAAAATTTGAATCCCCA

>MH401899

AGTAATTAATAATTCCGTATGTAGAAATATAAGGCATAAACCACATTGA

CCCTAAAAAATAAGAAGAATTATAGTTATTTAAAGCCTTATTATAAAAAAATAAAGAAAT

ATTAGAAATTAAATACCCTCTAATTCCTCCTACAATACAAACAAATAAAGTTAATAACTT

TAAATAAGAAGGTAGAACTACTACTACTGGTGTAGGAAAAATTAATCAACTTAACATTCT

ACCCCCAAAAATTCTTAAAATTAATAACCCTATTATACTCTTTAACATAACTCAACCTTC

ATCATTTAATATATTTAAAGAAGAAAAATTTGAATCCCCA

>MH401900

AGTAATTAATAATTCCGTATGTAGAAATATAAGGCATAAACCACATT

GACCCTAAAAAATAAGAAGAATTATAGTTATTTAAAGCCTTATTATAAAAAAATAAAGAA

ATATTAGAAATTAAATACCCTCTAATTCCTCCTACAATACAAACAAATAAAGTTAATAAC

TTTAAATAAGAAGGTAGAACTACTACTACTGGTGTAGGAAAAATTAATCAACTTAACATT

CTACCCCCAAAAATTCTTAAAATTAATAACCCTATTATACTCTTTAACATAACTCAACCT

TCATCATTTAATATATTTAAAGAAGAAAAATTTGAATCCCCA

>MH401901

AATAATTAATAATTCCATATGTAGAAATATAAGGTATAAATCATATTGAA

CCTAAAAAGTAAGAAGAATTATAATTATTTAAAGCCTTATTATAAAAAAATAAAGAAATA

TTAGAAATTAAATAACCTCTAACTCCTCCTACAATACAAACAAATAAAGTTAATAATTTT

AAATAAGAAGGAAGAACTACTACTACTGGTGTAGGAAAAATTAATCAACTTAATATACTA

CCCCCAAAAATTCTCAAAATTAATAGTCCTATTATACTTTTTAATATAACTCAACCTTCA

TCATTTAGTATATTTAAAGAAGAAAAATTTGAATCCCCA

>MH401902

AATAATTAATAATTCCATATGTAGAAATATAAGGTATAAATCATATT

GAACCTAAAAAGTAAGAAGAATTATAATTATTTAAAGCCTTATTATAAAAAAATAAAGAA

ATATTAGAAATTAAATAACCTCTAACTCCTCCTACAATACAAACAAATAAAGTTAATAAT

TTTAAATAAGAAGGAAGAACTACTACTACTGGTGTAGGAAAAATTAATCAACTTAATATA

CTACCCCCAAAAATTCTCAAAATTAATAGTCCTATTATACTTTTTAATATAACTCAACCT

TCATCATTTAGTATATTTAAAGAAGAAAAATTTGAATCCCCA

>MH401903

AATAATTTATAATCCCATAAGTAGAAATATAAGGCATAAATCAT

ATTGAACCTAAAAAATAAGAACTATTATATATATTTAAAGCCTTATTAGAAAAAAATAAA

GAAATATTAGAAATTAAATAACCTATTAATCCTCCTACAATACACACAAATAAAGTTAAT

AACTTTAAATAATAAGGTAAAACAATTACCATAGGACTAGGAAAAATTAATCAACTTAAT

ATTCTACCCCCAAAAATTCTTAAAATTAATAATCCTATTATACTTTTTAATATAACTCAA

CCCTCATCATTTAATATATTTAAAGAAGAAAAATTCGAATCTCCA

>MH401904

AATAATTAATAATTCCATATGTAGAAATATAAGGTATAAATCATATTGA

ACCTAAAAAGTAAGAAGAATTATAATTATTTAAAGCCTTATTATAAAAAAATAAAGAAAT

ATTAGAAATTAAATAACCTCTAATTCCTCCTACAATACAAACAAATAAAGTTAATAATTT

TAAATAAAAAGGAAGAACTACTACTACTGGTGTAGGAAAAATTAATCAACTTAATATACT

ACCCCCAAAAATTCTCAAAATTAATAGTCCTATTATACTTTTTAATATAACTCAACCTTC

ATCATTTAGTATATTTAAAGAAGAAAAATTTGAATCTCCA

>MH401905

AATAATTAATAATTCCATAAGTAGAAATATAAGGTATAAACCACATAGAA

CCTAAAAAATAAGAAAAATTATAATTATTTAAAGCCTTATTATAAAAAAATAAAGAAACA

TTAGAAATTATATAACCTATAATACCCCCAACAATACAAACAAATAATGTTAATAATTTT

AAATAAGAAGGAAGAACAACTACTATAGGAGTAGGAAAAATCAATCATCTTAACATTCTC

CCACCAAAAATTCTTAAAATTAATAAACCTATTATACTTTTCAATATTACTCAACCTTCA

TCATTTAATATATTTAAAGAAGAAAAATTAGAATCACCA

>MH401906

AATAATTAATAATTCCATAAGTAGAAATATAAGGTATAAACCACATAGAAC

CTAAAAAATAAGAAAAATTATAATTATTTAAAGCCTTATTATAAAAAAATAAAGAAACAT

TAGAAATTATATAACCTATAATACCCCCAACAATACAAACAAATAATGTTAATAATTTTA

AATAAGAAGGAAGAACAACTACTATAGGAGTAGGAAAAATCAATCATCTTAACATTCTCC

CACCAAAAATTCTTAAAATTAATAAACCTATTATACTTTTCAATATTACTCAACCTTCAT

CATTTAATATATTTAAAGAAGAAAAATTAGAATCACCA

>MH401907

AATAATTAATAATTCCATAAGTAGAAATATAAGGTATAAACCAC

ATAGAACCTAAAAAATAAGAAAAATTATAATTATTTAAAGCCTTATTATAAAAAAATAAA

GAAACATTAGAAATTATATAACCTATAATACCCCCAACAATACAAACAAATAATGTTAAT

AATTTTAAATAAGAAGGAAGAACAACTACTATAGGAGTAGGAAAAATCAATCATCTTAAC

ATTCTCCCACCAAAAATTCTTAAAATTAATAAACCTATTATACTTTTCAATATTACTCAA

CCTTCATCATTTAATATATTTAAAGAAGAAAAATTAGAATCACCA

>MH401908

AATAATTAATAATTCCATAAGTAGAAATATAAGGTATAAACCACATAGAAC

CTAAAAAATAAGAAAAATTATAATTATTTAAAGCCTTATTATAAAAAAATAAAGAAACAT

TAGAAATTATATAACCTATAATACCCCCAACAATACAAACAAATAATGTTAATAATTTTA

AATAAGAAGGAAGAACAACTACTATAGGAGTAGGAAAAATCAATCATCTTAACATTCTCC

CACCAAAAATTCTTAAAATTAATAAACCTATTATACTTTTCAATATTACTCAACCTTCAT

CATTTAATATATTTAAAGAAGAAAAATTAGAATCACCA

>MH401909

AATAATTAATAATTCCATAAGTAGAAATATAAGGTATAAACCA

CATAGAACCTAAAAAATAAGAAAAATTATAATTATTTAAAGCCTTATTATAAAAAAATAA

AGAAACATTAGAAATTATATAACCTATAATACCCCCAACAATACAAACAAATAATGTTAA

TAATTTTAAATAAGAAGGAAGAACAACTACTATAGGGGTAGGAAAAATCAATCATCTTAA

CATTCTCCCACCAAAAATTCTTAAAATTAATAAACCTATTATACTTTTCAATATTACTCA

ACCTTCATCATTTAATATATTTAAAGAAGAAAAATTAGAATCACCA

>MH401910

AATAATTAATAATTCCATAAGTAGAAATATAAGGTATAAACCACATAGAACC

TAAAAAATAAGAAAAATTATAATTATTTAAAGCCTTATTATAAAAAAATAAAGAAACATT

AGAAATTATATAACCTATAATACCCCCAACAATACAAACAAATAATGTTAATAATTTTAA

ATAAGAAGGAAGAACAACTACTATAGGAGTAGGAAAAATCAATCATCTTAACATTCTCCC

ACCAAAAATTCTTAAAATTAATAAACCTATTATACTTTTCAATATTACTCAACCTTCATC

ATTTAATATATTTAAAGAAGAAAAATTAGAATCACCA

>MH401911

AATAATTAATAATTCCATAAGTAGAAATATAAGGTATAAACCACATAGAA

CCTAAAAAATAAGAAAAATTATAATTATTTAAAGCCTTATTATAAAAAAATAAAGAAACA

TTAGAAATTATATAACCTATAATACCCCCAACAATACAAACAAATAATGTTAATAATTTT

AAATAAGAAGGAAGAACAACTACTATAGGAGTAGGAAAAATCAATCATCTTAACATTCTC

CCACCAAAAATTCTTAAAATTAATAAACCTATTATACTTTTCAATATTACTCAACCTTCA

TCATTTAATATATTTAAAGAAGAAAAATTAGAATCACCA

>MH401912

AATAATTAATAATTCCATAAGTAGAAATATAAGGTATAAACCACATAG

AACCTAAAAAATAAGAAAAATTATAATTATTTAAAGCCTTATTATAAAAAAATAAAGAAA

CATTAGAAATTATATAACCTATAATACCCCCAACAATACAAACAAATAATGTTAATAATT

TTAAATAAGAAGGAAGAACAACTACTATAGGAGTAGGAAAAATCAATCATCTTAACATTC

TCCCACCAAAAATTCTTAAAATTAATAAACCTATTATACTTTTCAATATTACTCAACCTT

CATCATTTAATATATTTAAAGAAGAAAAATTAGAATCACCA

>MH401913

AATAATTAATAATTCCATAAGTAGAAATATAAGGT

ATAAACCACATAGAACCTAAAAAATAAGAAAAATTATAATTATTTAAAGCCTTATTATAA

AAAAATAAAGAAACATTAGAAATTATATAACCTATAATACCCCCAACAATACAAACAAAT

AATGTTAATAATTTTAAATAAGAAGGAAGAACAACTACTATAGGAGTAGGAAAAATCAAT

CATCTTAACATTCTCCCACCAAAAATTCTTAAAATTAATAAACCTATTATACTTTTCAAT

ATTACTCAACCTTCATCATTTAATATATTTAAAGAAGAAAAATTAGAATCACCA

>MH401914

AATAATTAATAATTCCATAAGTAGAAATATAAGGTATAAACCAC

ATAGAACCTAAAAAATAAGAAAAATTATAATTATTTAAAGCCTTATTATAAAAAAATAAA

GAAACATTAGAAATTATATAACCTATAATACCCCCAACAATACAAACAAATAATGTTAAT

AATTTTAAATAAGAAGGAAGAACAACTACTATAGGAGTAGGAAAAATCAATCATCTTAAC

ATTCTCCCACCAAAAATTCTTAAAATTAATAAACCTATTATACTTTTCAATATTACTCAA

CCTTCATCATTTAATATATTTAAAGAAGAAAAATTAGAATCACCA

>MH401915

AATAATTAATAATTCCATATGTAGAAATATAAGGTATAAATCATATTGA

ACCTAAAAAGTAAGAAGAATTATAATTATTTAAAGCCTTATTATAAAAAAATAAAGAAAT

ATTAGAAATTAAATAACCTCTAACTCCTCCTACAATACAAACAAATAAAGTTAATAATTT

TAAATAAGAAGGAAGAACTACTACTACTGGTGTAGGAAAAATTAATCAACTTAATATACT

ACCCCCAAAAATTCTCAAAATTAATAGTCCTATTATACTTTTTAATATAACTCAACCTTC

ATCATTTAGTATATTTAAAGAAGAAAAATTTGAATCCCCA

>MH401916

AATAATTAATAATTCCATATGTAGAAATATAAGGTATAAATCATATT

GAACCTAAAAAGTAAGAAGAATTATAATTATTTAAAGCCTTATTATAAAAAAATAAAGAA

ATATTAGAAATTAAATAACCTCTAACTCCTCCTACAATACAAACAAATAAAGTTAATAAT

TTTAAATAAGAAGGAAGAACTACTACTACTGGTGTAGGAAAAATTAATCAACTTAATATA

CTACCCCCAAAAATTCTCAAAATTAATAGTCCTATTATACTTTTTAATATAACTCAACCT

TCATCATTTAGTATATTTAAAGAAGAAAAATTTGAATCCCCA

>MH401917

AATAATTAATAATTCCATATGTAGAAATATAAGGTATAAATCATATTGA

ACCTAAAAAGTAAGAAGAATTATAATTATTTAAAGCCTTATTATAAAAAAATAAAGAAAT

ATTAGAAATTAAATAACCTCTAACTCCTCCTACAATACAAACAAATAAAGTTAATAATTT

TAAATAAGAAGGAAGAACTACTACTACTGGTGTAGGAAAAATTAATCAACTTAATATACT

ACCCCCAAAAATTCTCAAAATTAATAGTCCTATTATACTTTTTAATATAACTCAACCTTC

ATCATTTAGTATATTTAAAGAAGAAAAATTTGAATCCCCA

>MH401918

AATAATTAATAATTCCATATGTAGAAATATAAGGTATAAATCATA

TTGAACCTAAAAAGTAAGAAGAATTATAATTATTTAAAGCCTTATTATAAAAAAATAAAG

AAATATTAGAAATTAAATAACCTCTAACTCCTCCTACAATACAAACAAATAAAGTTAATA

ATTTTAAATAAGAAGGAAGAACTACTACTACTGGTGTAGGAAAAATTAATCAACTTAATA

TACTACCCCCAAAAATTCTCAAAATTAATAGTCCTATTATACTTTTTAATATAACTCAAC

CTTCATCATTTAGTATATTTAAAGAAGAAAAATTTGAATCCCCA

>MH401919

AATAATTAATAATTCCATAAGTAGAAATATAAGGTATAAACCACATAGA

ACCTAAAAAATAAGAAAAATTATAATTATTTAAAGCCTTATTATAAAAAAATAAAGAAAC

ATTAGAAATTATATAACCTATAATACCCCCAACAATACAAACAAATAATGTTAATAATTT

TAAATAAGAAGGAAGAACAACTACTATAGGAGTAGGAAAAATCAATCATCTTAACATTCT

CCCACCAAAAATTCTTAAAATTAATAAACCTATTATACTTTTCAATATTACTCAACCTTC

ATCATTTAATATATTTAAAGAAGAAAAATTAGAATCACCA

>MH401920

AATAATTAATAATTCCATATGTAGAAATATAAGGTATAAATCATATT

GAACCTAAAAAGTAAGAAGAATTATAATTATTTAAAGCCTTATTATAAAAAAATAAAGAA

ATATTAGAAATTAAATAACCTCTAACTCCTCCTACAATACAAACAAATAAAGTTAATAAT

TTTAAATAAGAAGGAAGAACTACTACTACTGGTGTAGGAAAAATTAATCAACTTAATATA

CTACCCCCAAAAATTCTCAAAATTAATAGTCCTATTATACTTTTTAATATAACTCAACCT

TCATCATTTAGTATATTTAAAGAAGAAAAATTTGAATCCCCA

>MH401921

AATAATTAATAATTCCGTATGTAGAAATATAAGGTATAAATCACATT

GACCCTAAAAAGTAAGAAGAGTTATAATTATTTAAAGCCTTATTGTAAAAAAATAAAGAA

ATGTTAGAAATTAAATAACCTCTAACTCCCCCTACAATACAAACAAATAAAGTTAATAAT

TTTAAATAATAAGGAAGAACCACTACTACAGGGGTAGGAAAAATCAATCAACTTAATATT

CTACCTCCAAAGATTCTCAAAATTAATAACCCTATTATACTTTTTAATATAACTCAACCT

TCATCATTTAATATATTTAAAGAAGAAAAATTAGAATCCCCA

>MH401922

AATAATTAATAATTCCGTAAGTAGAAATATAAGGTATAAATCATA

TTGATCCTAAAAAATAAGAAAAATTATAATTATTTAAAGCCTTATTATAAAAAAATAAAG

AAATATTTGAAATTATATAACCTCTTACTCCTCCTACAATACAAACAAATAATGTTAACA

ACTTTAAATATGAAGGTAATACTACTACTACTGGGGTAGGAAAAATTAATCATCTTAACA

TTCTCCCCCCAAAAATTCTTAAAATTAATAAACCTATTATACTTTTTAATATAACTCAAC

CTTCATCATTTAATATATTTAAAGAAGAAAAATTTGAATCACCA

>MH401923

AGTAATTAATAATTCCGTATGTAGAAATATAAGGCATAAACCACATTGAC

CCTAAAAAATAAGAAGAATTATAGTTATTTAAAGCCTTATTATAAAAAAATAAAGAAATA

TTAGAAATTAAATACCCTCTAATTCCTCCTACAATACAAACAAATAAAGTTAATAACTTT

AAATAAGAAGGTAGAACTACTACTACTGGTGTAGGAAAAATTAATCAACTTAACATTCTA

CCCCCAAAAATTCTTAAAATTAATAACCCTATTATACTCTTTAACATAACTCAACCTTCA

TCATTTAATATATTTAAAGAAGAAAAATTTGAATCCCCA

>MH401924

AATAATTAATAATTCCATATGTAGAAATATAAGGTATAAATCATATTG

AACCTAAAAAGTAAGAAGAATTATAATTATTTAAAGCCTTATTATAAAAAAATAAAGAAA

TATTAGAAATTAAATAACCTCTAACTCCTCCTACAATACAAACAAATAAAGTTAATAATT

TTAAATAAGAAGGAAGAACTACTACTACTGGTGTAGGAAAAATTAATCAACTTAATATAC

TACCCCCAAAAATTCTCAAAATTAATAGTCCTATTATACTTTTTAATATAACTCAACCTT

CATCATTTAGTATATTTAAAGAAGAAAAATTTGAATCCCCA

>MH401925

AGTAATTAATAATTCCGTATGTAGAAATATAAGGCATAAACCACA

TTGACCCTAAAAAATAAGAAGAATTATAGTTATTTAAAGCCTTATTATAAAAAAATAAAG

AAATATTAGAAATTAAATACCCTCTAATTCCTCCTACAATACAAACAAATAAAGTTAATA

ACTTTAAATAAGAAGGTAGAACTACTACTACTGGTGTAGGAAAAATTAATCAACTTAACA

TTCTACCCCCAAAAATTCTTAAAATTAATAACCCTATTATACTCTTTAACATAACTCAAC

CTTCATCATTTAATATATTTAAAGAAGAAAAATTTGAATCCCCA

>MH401926

AATAATTAATAATTCCGTAAGTAGAAATATAAGGTATAAACCACATT

GACCCTAAAAAATAAGAAAAATTATAATTATTTAAAGCCTTATTATAAAAAAATAAAGAA

ATATTTGAAATTATATACCCTCTTACTCCTCCTACAATACAAACAAATAATGTTAATAAC

TTCAAATATGAAGGAAGAACTACAACTACTGGAGTTGGAAAAATTAATCATCTTAATATT

CTTCCCCCAAAAATTCTTAAAATTAATAACCCTATTATACTTTTTAATATTACTCAACCT

TCATCATTTAATATATTTAAAGAAGAAAAATTTGAATCACCA

>MH588559

GATAATTATTAATTCCATATGTTGAAATATAAGGTATAAATCATATTGAACCTAAAAAATAAGACATATTATAATTACTAAAAGAATTATTATTAAAAAATAAAGAAACATTAGAAATATAATAACCAAATAATCCTCCAATAATACATACAATTAAAGTTAATAATTTTATTACTTTAGGTAAAATAATTACTACAGGAGTTGGAAAAATTAATCAACTTAATATTCTTCCTCCAATAATTCTTATAATTAATAAACCTATTATACTTTTTAATATAATTCAACCTTCATCATTTAATATATTTAAAGAAGAAAAATTAGAATTACCC

>MH588560

GATAATTATTAATTCCATATGTTGAAATATAAGGTATAAATCATATTGAACCTAAAAAATAAGACATATTATAATTACTAAAAGAATTATTATTAAAAAATAAAGAAACATTAGAAATATAATAACCAAATAATCCTCCAATAATACATACAATTAAAGTTAATAATTTTATTACTTTAGGTAAAATAATTACTACAGGAGTTGGAAAAATTAATCAACTTAATATTCTTCCTCCAATAATTCTTATAATTAATAAACCTATTATACTTTTTAATATAATTCAACCTTCATCATTTAATATATTTAAAGAAGAAAAATTAGAATTACCC

>MH588561

GATAACTCACAATTCCGTAAGTCGAAATAAAAGGTATAAATCATATTGAGCCAAAATAATATCTTAAAACATATGTTATTAAAGACTTATTAACATAAAATAAAGAAACGTTAGAAATTAAATAACCTAAAATACCCCCTACGATACAAACAAATAATGTTAATAACTTTAAATAATAAGGTAAACACACTATTAAAGGAGTAGGAAAAATTAATCAATTTAATATTCTACCTCCAATAATTCTTATAAATAATAATCCTAGTATACCCTTTAATATAATTCATCCCTCATCATTAAGTATATTTAAAGACCCGCAATTTAAATCCCCA

>MH588562

GATAATTAATAATTCCGTAAGTTGAAATATAAGGTATAAATCATATTGATCCTAAAAAATAAGAAATATTATATAAGTCTAAAGATTTATTATAAAAGTATAAATTAACCAATGAAATGAAATAACCAAATAAACCCCCAGTAATACAAACAAATAAAGTCAACTGTTTTAAATAAATAGGTAAACAAATCATATAAGGAGTAGGAAAAATTAATCAATTTAAAATTCTACCTCCAATAATTGATATAATAAGTAAACCTATTATACCCTTTAATATTACTCATCCTTCATCTCTTAAAACATTTAATCTTCTACAATTTAAATCTCCA

>MH588563

GATGATTATTAAAnCCATAAGTAGAAATATAAGGTATAAACCATATTGAACCTAAAAAATATGAAAAATTATATATATTTAAAGCCCTATTATAAAAAAATAATGAAATATTTGAAATAAAATACCCTATAATCCCACCAATTAAACAAACAAATAAAGTTAATATTTTTAAATGTATTGGTAAAACTACAACAATAGGAGTTGAAAATATTAATCATCTTAGTATTCTTCCTCCAAAAATTCTTAAAATTAAAAGTCCCATTATTCTTTTTAGTATAATTCATCTTTCATCATTCAACAAATTTAAAGAACTAAAATTTGAATCCCCA

>MH588564

AATGATTATTAAAnCCATAAGTAGAAATATAAGGTATAAACCATATTGAACCTAAAAAATATGAAAAATTATATATATTTAAAGCCCTATTATAAAAAAATAATGAAATATTTGAAATAAAATACCCTATAATCCCACCAATTAAACAAACAAATAAAGTTAATATTTTTAAATGTATTGGTAAAACTACAACAATAGGAGTTGAAAATATTAATCATCTTAGTATTCTTCCTCCAAAAATTCTTAAAATTAAAAGTCCCATTATTCTTTTTAGTATAATTCATCTTTCATCATTCAACAAATTTAAAGAACTAAAATTTGAATCCCCA

>MH588565

AATAATTTATAATCCCATAAGTAGAGATATAAGGTATAAATCATATTGAACCTAAAAAATAAGAACTATTATAATTACTTAAAGCCTTATTAAAAAAAAATAAAGAAACATTAGAAATTAAATAACCTATCAATCCTCCTATAATACAAACAAATAAAATTAACAACTTTAAATAAGAAGGTAATACAATTACTATAGGTCTAGGAAAAATTAATCATCTAAGTATTCTTCCTCCAAAAATTCTTAAAATTAATAAACCTATTATACTTTTTAATATAACTCAACCTTCATCATTTAACATATTTAAAGAAGAAAAATTTGAATCTCCA

>MH588566

AATAATTAATAATTCCATAAGTAGAAATATAAGGTATAAACCATATAGAGCCTAAAAAATAAGAAGAATGATAATTATTTAAAGCCTTATTATAAAAAAATAAAGAGACATGAGAAATTATGTAACCTATTAGTCCTCCTACAATACAAACAAATAAAGTTAATAATTTTAAATAAAAAGGAAGAACTACTACTACTGGTGTAGGAAAAATTAATCATCTTAACATTCTCCCTCCAAAAATTCTTAAAATCAATAAACCTAATATACTTTTTAATATTACTCAACCTTCATCATTAAGTATATTTAAAGATGAAAAATTTGAATTCCCA

>MH588567

AATAATTAATAATACCATATGTAGAAATATATGGTATAAATCATATTGAACCTAAAAAATAAGAAGAATTATAATTATTTAATGCCTTATTATAAAAAAATAATGAAACATTAGAAATTATATAACCACTTACTCCTCCTACAATACAAACAAATATAGTTAACAACTTTAAATATATTGGAAGAACTACTAACACTGGAGTAGGAAAAATTAATCATCTTAATATTCTCCCTCCAAAAATTCTTAGAATCAATAAACCTATTATACTTTTTAATATTACTCAACCTTCATCATTTAATATATTTAAAGATGAAAAATTTGAATCTCCA

>MH588568

AATAATTAATAATACCATATGTAGAAATATATGGTATAAATCATATTGAACCTAAAAAATAAGAAGAATTATAATTATTTAATGCCTTATTATAAAAAAATAATGAAACATTAGAAATTATATAACCACTTACTCCTCCTACAATACAAACAAATATAGTTAACAACTTTAAATATATTGGAAGAACTACTAACACTGGAGTAGGAAAAATTAATCATCTTAATATTCTCCCTCCAAAAATTCTTAGAATCAATAAACCTATTATACTTTTTAATATTACTCAACCTTCATCATTTAATATATTTAAAGATGAAAAATTTGAATCTCCA

>MH588569

AATAATTAATAATACCATATGTAGAAATATATGGTATAAATCATATTGAACCTAAAAAATAAGAAGAATTATAATTATTTAATGCCTTATTATAAAAAAATAATGAAACATTAGAAATTATATAACCACTTACTCCTCCTACAATACAAACAAATATAGTTAACAACTTTAAATATATTGGAAGAACTACTAACACTGGAGTAGGAAAAATTAATCATCTTAATATTCTCCCTCCAAAAATTCTTAGAATCAATAAACCTATTATACTTTTTAATATTACTCAACCTTCATCATTTAATATATTTAAAGATGAAAAATTTGAATCTCCA

>MH588570

AATAATTAATAATTCCGTAAGTAGAAATATAAGGTATAAATCATATTGACCCTAAAAAATAAGAAAAATTATAATTATTTAAAGCCTTATTATAAAAAAATAAAGAAATATTTGAAATTATATAACCTCTTACTCCTCCTACAATACAAACAAATAATGTTAACAACTTTAAATATGAAGGTAATACTACTACTACTGGGGTAGGAAAAATTAATCATCTTAACATTCTCCCCCCAAAAATTCTTAAAATTAATAAACCTATTATACTTTTTAATATAACTCAACCTTCATCATTTAATATATTTAAAGAAGAAAAATTTGAATCACCA

>MH588571

AAGATTTAATAATTCCGTAAGTAGAAATATAAGGTATAAATCATATTGACCCTAAAAAATAAGAAAAATTATAATTATTTAAAGCCTTATTATAAAAAAATAAAGAAATATTTGAAATTATATAACCTCTTACTCCTCCTACAATACAAACAAATAATGTTAACAACTTTAAATATGAAGGTAATACTACTACTACTGGGGTAGGAAAAATTAATCATCTTAACATTCTCCCCCCAAAAATTCTTAAAATTAATAAACCTATTATACTTTTTAATATAACTCAACCTTCATCATTTAATATATTTAAAGAAGAAAAATTTGAATCACCA

>MH588572

AATAATTAATAATCCCGTAAGTAGAAATATAAGGTATAAACCATATTGACCCTAAAAAATAAGAAAAATTATAATTATTTAAAGCCTTATTATAAAAAAATAAAGAAATATTTGAAATTATATACCCTCTTACTCCTCCTACAATACAAACAAATAATGTTAATAACTTCAAATATGAAGGAAGAACTACAACTACTGGAGTTGGAAAAATTAATCATCTTAATATTCTTCCTCCAAAAATTCTTAAAATTAATAACCCTATTATACTCTTTAATATTACTCAACCTTCATCATTTAATATATTTAAAGAAGAAAAATTTGAATCACCA

>MH588573

AATAATTAATAATCCCGTAAGTAGAAATATAAGGTATAAACCATATTGACCCTAAAAAATAAGAAAAATTATAATTATTTAAAGCCTTATTATAAAAAAATAAAGAAATATTTGAAATTATATACCCTCTTACTCCTCCTACAATACAAACAAATAATGTTAATAACTTCAAATATGAAGGAAGAACTACAACTACTGGAGTTGGAAAAATTAATCATCTTAATATTCTTCCTCCAAAAATTCTTAAAATTAATAACCCTATTATACTCTTTAATATTACTCAACCTTCATCATTTAATATATTTAAAGAAGAAAAATTTGAATCACCA

>MH588574

GATAATTAATGATTCCATAAGTAGAAATATAAGGTATAAACCACATTGAACCTAAAAAATAAGAAAAATTATAATTATTTAAAGCCTTATTATAAAAAAATAAGGAAATATTTGAAATTATATACCCTCTTACTCCTCCTACAATACAAACAAATAATGTTAATAACTTTAAATATGAAGGAAGAACTACAACTACTGGAGTTGGAAAAATTAATCATCTTAATATTCTTCCTCCAAAAATTCTTAAAATTAAGAATCCTATTATACTTTTTAATATTACTCAACCTTCATCATTTAATATATTTAAAGAAGAAAAATTTGAATCACCA

>MH588575

GATAATTAATAATTCCATAAGTAGAAATATAAGGTATAAACCACATAGAACCTAAAAAATAAGAAAAATTATAATTATTTAAAGCCTTATTATAAAAAAATAAAGAAACATTAGAAATTATATAACCTATAATACCCCCAACAATACAAACAAATAATGTTAATAATTTTAAATAAAAAGGAAGAACAACTACTATAGGAGTAGGAAAAATCAATCATCTTAACATTCTCCCACCAAAAATTCTTAAAATTAATAAACCTATTATACTTTTCAATATTACTCAACCTTCATCATTTAATATATTTAAAGAAGAAAAATTAGAATCACCA

>MH588576

AATAATTAATAATTCCATAAGTAGAAATATAAGGTATAAACCACATAGAACCTAAAAAATAAGAAAAATTATAATTATTTAAAGCCTTATTATAAAAAAATAAAGAAACATTAGAAATTATATAACCTATAATACCCCCAACAATACAAACAAATAATGTTAATAATTTTAAATAAGAAGGAAGAACAACTACTATAGGAGTAGGAAAAATCAATCATCTTAACATTCTCCCACCAAAAATTCTTAAAATTAATAAACCTATTATACTTTTCAATATTACTCAACCTTCATCATTTAATATATTTAAAGAAGAAAAATTAGAATCACCA

>MH588577

AATAATTAATAATTCCATAAGTAGAAATATAAGGTATAAACCACATAGAACCTAAAAAATAAGAAAAATTATAATTATTTAAAGCCTTATTATAAAAAAATAAAGAAACATTAGAAATTATATAACCTATAATACCCCCAACAATACAAACAAATAATGTTAATAATTTTAAATAAGAAGGAAGAACAACTACTATAGGAGTAGGAAAAATCAATCATCTTAACATTCTCCCACCAAAAATTCTTAAAATTAATAAACCTATTATACTTTTCAATATTACTCAACCTTCATCATTTAATATATTTAAAGAAGAAAAATTAGAATCACCA

>MH588578

AATAATTAATAATTCCGTATGTAGAAATATAAGGCATAAACCACATTGACCCTAAAAAATAAGAAGAATTATAGTTATTTAAAGCCTTATTATAAAAAAATAAAGAAATATTAGAAATTAAATACCCTCTAATTCCTCCTACAATACAAACAAATAAAGTTAATAACTTTAAATAAGAAGGTAGAACTACTACTACTGGTGTAGGAAAAATTAATCAACTTAACATTCTACCCCCAAAAATTCTTAAAATTAATAACCCTATTATACTCTTTAACATAACTCAACCTTCATCATTTAATATATTTAAAGAAGAAAAATTTGAATCCCCA

>MH588579

AGTAATTAATAATTCCGTATGTAGAAATATAAGGCATAAACCACATTGACCCTAAAAAATAAGAAGAATTATAGTTATTTAAAGCCTTATTATAAAAAAATAAAGAAATATTAGAAATTAAATACCCTCTAATTCCTCCTACAATACAAACAAATAAAGTTAATAACTTTAAATAAGAAGGTAGAACTACTACTACTGGTGTAGGAAAAATTAATCAACTTAACATTCTACCCCCAAAAATTCTTAAAATTAATAACCCTATTATACTCTTTAACATAACTCAACCTTCATCATTTAATATATTTAAAGAAGAAAAATTTGAATCCCCA

>MH588580

AGTAATTAATAATTCCGTATGTAGAAATATAAGGCATAAACCACATTGACCCTAAAAAATAAGAAGAATTATAGTTATTTAAAGCCTTATTATAAAAAAATAAAGAAATATTAGAAATTAAATACCCTCTAATTCCTCCTACAATACAAACAAATAAAGTTAATAACTTTAAATAAGAAGGTAGAACTACTACTACTGGTGTAGGAAAAATTAATCAACTTAACATTCTACCCCCAAAAATTCTTAAAATTAATAACCCTATTATACTCTTTAACATAACTCAACCTTCATCATTTAATATATTTAAAGAAGAAAAATTTGAATCCCCA

>MH588581

AGTAATTAATAATTCCGTATGTAGAAATATAAGGCATAAACCACATTGACCCTAAAAAATAAGAAGAATTATAGTTATTTAAAGCCTTATTATAAAAAAATAAAGAAATATTAGAAATTAAATACCCTCTAATTCCTCCTACAATACAAACAAATAAAGTTAATAACTTTAAATAAGAAGGTAGAACTACTACTACTGGTGTAGGAAAAATTAATCAACTTAACATTCTACCCCCAAAAATTCTTAAAATTAATAACCCTATTATACTCTTTAACATAACTCAACCTTCATCATTTAATATATTTAAAGAAGAAAAATTTGAATCCCCA

>MH588582

AATAATTAATAATTCCATATGTAGAAATATAAGGTATAAATCATATTGAACCTAAAAAGTAAGAAGAATTATAATTATTTAAAGCCTTATTATAAAAAAATAAAGAAATATTAGAAATTAAATAACCTCTAACTCCTCCTACAATACAAACAAATAAAGTTAATAATTTTAAATAAGAAGGAAGAACTACTACTACTGGTGTAGGAAAAATTAATCAACTTAATATACTACCCCCAAAAATTCTCAAAATTAATAGCCCTATTATACTTTTTAATATAACTCAACCTTCATCATTTAGTATATTTAAAGAAGAAAAATTTGAATCCCCA

>MH588583

AATAATTAATAATTCCATATGTAGAAATATAAGGTATAAATCATATTGAACCTAAAAAGTAAGAAGAATTATAATTATTTAAAGCCTTATTATAAAAAAATAAAGAAATATTAGAAATTAAATAACCTCTAACTCCTCCTACAATACAAACAAATAAAGTTAATAATTTTAAATAAGAAGGAAGAACTACTACTACTGGCGTAGGAAAAATTAATCAACTTAATATACTACCCCCAAAAATTCTCAAAATTAATAGTCCTATTATACTTTTTAATATAACTCAACCTTCATCATTTAGTATATTTAAAGAAGAAAAATTTGAATCCCCA

>MH588584

AATAATTAATAATTCCATATGTAGAAATATAAGGTATAAATCATATTGAACCTAAAAAGTAAGAAGAATTATAATTATTTAAAGCCTTATTATAAAAAAATAAAGAAATATTAGAAATTAAATAACCTCTAACTCCTCCTACAATACAAACAAATAAAGTTAATAATTTTAAATAAGAAGGAAGAACTACTACTACTGGTGTAGGAAAAATTAATCAACTTAATATACTACCCCCAAAAATTCTCAAAATTAATAGTCCTATTATACTTTTTAATATAACTCAACCTTCATCATTTAGTATATTTAAAGAAGAAAAATTTGAATCCCCA

>MH588585

AATAATTAATAATTCCATATGTAGAAATATAAGGTATAAATCATATTGAACCTAAAAAGTAAGAAGAATTATAATTATTTAAAGCCTTATTATAAAAAAATAAAGAAATATTAGAAATTAAATAACCTCTAACTCCTCCTACAATACAAACAAATAAAGTTAATAATTTTAAATAAGAAGGAAGAACTACTACTACTGGTGTAGGAAAAATTAATCAACTTAATATACTACCCCCAAAAATTCTCAAAATTAATAGTCCTATTATACTTTTTAATATAACTCAACCTTCATCATTTAGTATATTTAAAGAAGAAAAATTTGAATCCCCA

>MH588586

AATAATTAATAATTCCATATGTAGAAATATAAGGTATAAATCATATTGAACCTAAAAAGTAAGAAGAATTATAATTATTTAAAGCCTTATTATAAAAAAATAAAGAAATATTAGAAATTAAATAACCTCTAACTCCTCCTACAATACAAACAAATAAAGTTAATAATTTTAAATAAGAAGGAAGAACTACTACTACTGGTGTAGGAAAAATTAATCAACTTAATATACTACCCCCAAAAATTCTCAAAATTAATAGTCCTATTATACTTTTTAATATAACTCAACCTTCATCATTTAGTATATTTAAAGAAGAAAAATTTGAATCCCCA

>MH588587

AATAATTAATAATTCCATATGTAGAAATATAAGGTATAAATCATATTGAACCTAAAAAGTAAGAAGAATTATAATTATTTAAAGCCTTATTATAAAAAAATAAAGAAATATTAGAAATTAAATAACCTCTAACTCCTCCTACAATACAAACAAATAAAGTTAATAATTTTAAATAAGAAGGAAGAACTACTACTACTGGTGTAGGAAAAATTAATCAACTTAATATACTACCCCCAAAAATTCTCAAAATTAATAGTCCTATTATACTTTTTAATATAACTCAACCTTCATCATTTAGTATATTTAAAGAAGAAAAATTTGAATCCCCA

>MH588588

AATAATTAATAATTCCATATGTAGAAATATAAGGTATAAATCATATTGAACCTAAAAAGTAAGAAGAATTATAATTATTTAAAGCCTTATTATAAAAAAATAAAGAAATATTAGAAATTAAATAACCTCTAACTCCTCCTACAATACAAACAAATAAAGTTAATAATTTTAAATAAGAAGGAAGAACTACTACTACTGGTGTAGGAAAAATTAATCAACTTAATATACTACCCCCAAAAATTCTCAAAATTAATAGTCCTATTATACTTTTTAATATAACTCAACCTTCATCATTTAGTATATTTAAAGAAGAAAAATTTGAATCCCCA

>MH588589

AATAATTAATAATTCCATATGTAGAAATATAAGGTATAAATCATATTGAACCTAAAAAGTAAGAAGAATTATAATTATTTAAAGCCTTATTATAAAAAAATAAAGAAATATTAGAAATTAAATAACCTCTAACTCCTCCTACAATACAAACAAATAAAGTTAATAATTTTAAATAAGAAGGAAGAACTACTACTACTGGTGTAGGAAAAATTAATCAACTTAATATACTACCCCCAAAAATTCTCAAAATTAATAGTCCTATTATACTTTTTAATATAACTCAACCTTCATCATTTAGTATATTTAAAGAAGAAAAATTTGAATCCCCA

>MH588590

AATAATTAATAATTCCATATGTAGAAATATAAGGTATAAATCATATTGAACCTAAAAAGTAAGAAGAATTATAATTATTTAAAGCCTTATTATAAAAAAATAAAGAAATATTAGAAATTAAATAACCTCTAACTCCTCCTACAATACAAACAAATAAAGTTAATAATTTTAAATAAGAAGGAAGAACTACTACTACTGGTGTAGGAAAAATTAATCAACTTAATATACTACCCCCAAAAATTCTCAAAATTAATAGTCCTATTATACTTTTTAATATAACTCAACCTTCATCATTTAGTATATTTAAAGAAGAAAAATTTGAATCCCCA

>MH588591

GAATATTAATAATTCCATATGTAGAAATATAAGGTATAAATCATATTGAACCTAAAAAGTAAGAAGAATTATAATTATTTAAAGCCTTATTATAAAAAAATAAAGAAATATTAGAAATTAAATAACCTCTAACTCCTCCTACAATACAAACAAATAAAGTTAATAATTTTAAATAAGAAGGAAGAACTACTACTACTGGTGTAGGAAAAATTAATCAACTTAATATACTACCCCCAAAAATTCTCAAAATTAATAGTCCTATTATACTTTTTAATATAACTCAACCTTCATCATTTAGTATATTTAAAGAAGAAAAATTTGAATCCCCA

>MH588592

AATAATTAATAATTCCATATGTAGAAATATAAGGTATAAATCATATTGAACCTAAAAAGTAAGAAGAATTATAATTATTTAAAGCCTTATTATAAAAAAATAAAGAAATATTAGAAATTAAATAACCTCTAACTCCTCCTACAATACAAACAAATAAAGTTAATAATTTTAAATAAGAAGGAAGAACTACTACTACTGGTGTAGGAAAAATTAATCAACTTAATATACTACCCCCAAAAATTCTCAAAATTAATAGTCCTATTATACTTTTTAATATAACTCAACCTTCATCATTTAGTATATTTAAAGAAGAAAAATTTGAATCCCCA

>MH588593

AATAATTAATAATTCCATATGTAGAAATATAAGGTATAAATCATATTGAACCTAAAAAGTAAGAAGAATTATAATTATTTAAAGCCTTATTATAAAAAAATAAAGAAATATTAGAAATTAAATAACCTCTAACTCCTCCTACAATACAAACAAATAAAGTTAATAATTTTAAATAAGAAGGAAGAACTACTACTACTGGTGTAGGAAAAATTAATCAACTTAATATACTACCCCCAAAAATTCTCAAAATTAATAGTCCTATTATACTTTTTAATATAACTCAACCTTCATCATTTAGTATATTTAAAGAAGAAAAATTTGAATCCCCA

**EF1a sequences**

>MH401927

GTTGTCTTC

GCTCCCGCTAACATCACCACTGAAGTCAAGTCCGTTGAAATGCACCACGAAGCTCTCGTT

GAAGCCGTCCCCGGTGACAACGTTGGTTTCAACGTAAAGAACGTCTCCGTCAAGGAATTG

CGTCGTGGTTACGTCGCTGGTGACTCCAAGGCTAACCCCCCCAAGGGCGCCGCTGACTTC

ACCGCTCAAGTCATCGTCTTGAACCACCCTGGTCAAATCTCCAACGGTTACACCCCCGTT

TTGGATTGTCACACCGCTCACATTGCTTGCAAATTCGCCGAAATCAAGGAGAAGGTCGAT

>MH401928

GTTGTCTTCGCTCCCGCTAACATCACCACTGAAGTCAAG

TCCGTAGAAATGCACCACGAAGCTCTCGTTGAAGCTGTTCCCGGTGACAACGTTGGTTTC

AACGTTAAGAACGTCTCCGTCAAGGAATTGCGTCGTGGTTACGTCGCTGGTGACTCCAAG

GCTAGCCCCCCCAAGGGTGCTGCTGACTTCACCGCTCAAGTCATCGTCTTGAACCACCCT

GGTCAAATCTCCAACGGTTACACTCCCGTTTTGGATTGTCACACCGCTCACATTGCTTGC

AAATTCGCCGAAATCAAGGAGAAGGTCGAT

>MH401929

GTTGTCTTCGCTCCCGCTAACATCACCACTGAAGTCAAG

TCCGTAGAAATGCACCACGAAGCTCTCGTTGAAGCTGTTCCCGGTGACAACGTTGGTTTC

AACGTTAAGAACGTCTCCGTCAAGGAATTGCGTCGTGGTTACGTCGCTGGTGACTCCAAG

GCTAGCCCCCCCAAGGGTGCTGCTGACTTCACCGCTCAAGTCATCGTCTTGAACCACCCT

GGTCAAATCTCCAACGGTTACACTCCCGTTTTGGATTGTCACACCGCTCACATTGCTTGC

AAATTCGCCGAAATCAAGGAGAAGGTCGAT

>MH401930

GTTGTCTTCGCTCCCGCTAACATCACCACTGAAGT

CAAGTCCGTAGAAATGCACCACGAAGCTCTCGTTGAAGCTGTTCCCGGTGACAACGTTGG

TTTCAACGTTAAGAACGTCTCCGTCAAGGAATTGCGTCGTGGTTACGTCGCTGGTGACTC

CAAGGCTAGCCCCCCCAAGGGTGCTGCTGACTTCACCGCTCAAGTCATCGTCTTGAACCA

CCCTGGTCAAATCTCCAACGGTTACACTCCCGTTTTGGATTGTCACACCGCTCACATTGC

TTGCAAATTCGCCGAAATCAAGGAGAAGGTCGAT

>MH401931

GTTGTCTTCGCTCCCGCTAACATCACCACTGAAG

TCAAGTCCGTAGAAATGCACCACGAAGCTCTCGTTGAAGCTGTTCCCGGTGACAACGTTG

GTTTCAACGTTAAGAACGTCTCCGTCAAGGAATTGCGTCGTGGTTACGTCGCTGGTGACT

CCAAGGCTAGCCCCCCCAAGGGTGCTGCTGACTTCACCGCTCAAGTCATCGTCTTGAACC

ACCCTGGTCAAATCTCCAACGGTTACACTCCCGTTTTGGATTGTCACACCGCTCACATTG

CTTGCAAATTCGCCGAAATCAAGGAGAAGGTCGAT

>MH401932

GTTGTCTTCGCTCCCGCTAACATCACCACTGAAGT

CAAGTCCGTAGAAATGCACCACGAAGCTCTCGTTGAAGCTGTTCCCGGTGACAACGTTGG

TTTCAACGTTAAGAACGTCTCCGTCAAGGAATTGCGTCGTGGTTACGTCGCTGGTGACTC

CAAGGCTAGCCCCCCCAAGGGTGCTGCTGACTTCACCGCTCAAGTCATCGTCTTGAACCA

CCCTGGTCAAATCTCCAACGGTTACACTCCCGTTTTGGATTGTCACACCGCTCACATTGC

TTGCAAATTCGCCGAAATCAAGGAGAAGGTCGAT

>MH401933

GTTGTCTTCGCTCCCGCTAACATCACCACTGAAGTCAAG

TCCGTTGAAATGCACCACGAAGCTCTCGTTGAAGCCGTCCCCGGTGACAACGTTGGTTTC

AACGTAAAGAACGTCTCCGTCAAGGAATTGCGTCGTGGTTACGTCGCTGGTGACTCCAAG

GCTAACCCCCCCAAGGGCGCCGCTGACTTCACCGCTCAAGTCATCGTCTTGAACCACCCT

GGTCAAATCTCCAACGGTTACACCCCCGTTTTGGATTGTCACACCGCTCACATTGCTTGC

AAATTCGCCGAAATCAAGGAGAAGGTCGAT

>MH401934

GTTGTCTTCGCTCCCGCTAACATCACCACTGAAGTCAAGT

CCGTTGAAATGCACCACGAAGCTCTCGTTGAAGCCGTCCCCGGTGACAACGTTGGTTTCA

ACGTAAAGAACGTCTCCGTCAAGGAATTGCGTCGTGGTTACGTCGCTGGTGACTCCAAGG

CCAACCCCCCCAAGGGCGCCGCTGACTTCACCGCTCAAGTCATCGTCTTGAACCACCCTG

GTCAAATCTCCAACGGTTACACCCCCGTTTTGGATTGTCACACCGCTCACATTGCTTGCA

AATTCGCCGAAATCAAGGAGAAGGTCGAT

>MH401935

GTTGTCTTCGCTCCCGCTAACATCACCACTGAAGTCAAG

TCCGTTGAAATGCACCACGAAGCTCTCGTTGAAGCCGTCCCCGGTGACAACGTTGGTTTC

AACGTAAAGAACGTCTCCGTCAAGGAATTGCGTCGTGGTTACGTCGCTGGTGACTCCAAG

GCTAACCCCCCCAAGGGCGCCGCTGACTTCACCGCTCAAGTCATCGTCTTGAACCACCCT

GGTCAAATCTCCAACGGTTACACCCCCGTTTTGGATTGTCACACCGCTCACATTGCTTGC

AAATTCGCCGAAATCAAGGAGAAGGTCGAT

>MH401936

GTTGTCTTCGCTCCCGCTAACATCACCACTGAAGTCAAGTCCGTTGAAATGC

ACCACGAAGCTCTCGTTGAAGCCGTCCCCGGTGACAACGTTGGTTTCAACGTAAAGAACG

TCTCCGTCAAGGAATTGCGTCGTGGTTACGTCGCTGGTGACTCCAAGGCCAACCCCCCCA

AGGGCGCCGCTGACTTCACCGCTCAAGTCATCGTCTTGAACCACCCTGGTCAAATCTCCA

ACGGTTATACCCCCGTTTTGGATTGTCACACCGCTCACATTGCTTGCAAATTCGCCGAAA

TCAAGGAGAAGGTCGAT

>MH401937

GTTGTCTTCGCTCCCGCTAACATCACCACTGAAGTCAAG

TCCGTTGAAATGCACCACGAAGCTCTCGTTGAAGCCGTCCCCGGTGACAACGTTGGTTTC

AACGTAAAGAACGTCTCCGTCAAGGAATTGCGTCGTGGTTACGTCGCTGGTGACTCCAAG

GCTAACCCCCCCAAGGGCGCCGCTGACTTCACCGCTCAAGTCATCGTCTTGAACCACCCT

GGTCAAATCTCCAACGGTTACACCCCCGTTTTGGATTGTCACACCGCTCACATTGCTTGC

AAATTCGCCGAAATCAAGGAGAAGGTCGAT

>MH401938

GTTGTCTTCGCTCCCGCTAACATCACCACTGAAGTCAAATCCGTAGAAATGCACCACGAAGCTCTCGTT

GAAGCCGTCCCCGGTGACAACGTTGGTTTCAACGTTAAGAACGTCTCCGTCAAGGAATTG

CGTCGTGGTTACGTCGCTGGTGACTCCAAGGCTAGCCCCCCCAAGGGTGCTGCTGACTTC

ACCGCTCAAGTCATCGTCTTGAACCACCCTGGTCAAATCTCCAACGGTTACACTCCCGTT

TTGGATTGTCACACCGCTCACATTGCTTGCAAATTCGCCGAAATCAAGGAGAAGGTCGAT

>MH401939

GTTGTCTTCGCTCCCGCTAACATCACCACTGAAG

TCAAGTCCGTTGAAATGCACCACGAAGCTCTCGTTGAAGCCGTCCCCGGTGACAACGTTG

GTTTCAACGTAAAGAACGTCTCCGTTAAGGAATTGCGTCGTGGTTACGTCGCTGGTGACT

CCAAGGCCAACCCCCCCAAGGGCGCCGCTGACTTCACCGCTCAAGTCATCGTCTTGAACC

ACCCTGGTCAAATCTCCAACGGTTACACCCCCGTTTTGGATTGTCACACCGCTCACATTG

CTTGCAAATTCGCCGAAATCAAGGAGAAGGTCGAT

>MH401940

GTTGTCTTCGCTCCCGCTAACATCACCACTGAAGTCAAG

TCCGTTGAAATGCACCACGAAGCTCTCGTTGAAGCCGTCCCCGGTGACAACGTTGGTTTC

AACGTAAAGAACGTCTCCGTCAAGGAATTGCGTCGTGGTTACGTCGCTGGTGACTCCAAG

GCCAACCCCCCCAAGGGCGCCGCTGACTTCACCGCTCAAGTCATCGTCTTGAACCACCCT

GGTCAAATCTCCAACGGTTACACCCCCGTTTTGGATTGTCACACCGCTCACATTGCTTGC

AAATTCGCCGAAATCAAGGAGAAGGTCGAT

>MH401941

GTTGTCATCGCTCCCGCTAACATCACCACTGAAGTCAAGTCCGTTGAAAT

GCACCACGAAGCTCTCGTTGAAGCCGTCCCCGGTGACAACGTTGGTTTCAACGTAAAGAA

CGTCTCCGTCAAGGAATTGCGTCGTGGTTACGTCGCTGGTGACTCCAAGGCCAACCCCCC

CAAGGGTGCCGCTGACTTCACCGCTCAAGTCATCGTCTTGAACCATCCCGGTCAAATCTC

CAACGGTTACACTCCCGTTTTGGATTGTCACACCGCTCACATTGCTTGCAAATTCGCCGA

AATCAAGGAGAAGGTCGAT

>MH401942

GTTGTCTTCGCTCCCGCTAACATCACCACTGAAGTCAAATCCGTAG

AAATGCACCACGAAGCTCTCGTTGAAGCCGTCCCCGGTGACAACGTTGGTTTCAACGTTA

AGAACGTCTCCGTCAAGGAATTGCGTCGTGGTTACGTCGCTGGTGACTCCAAGGCTAGCC

CCCCCAAGGGTGCTGCTGACTTCACCGCTCAAGTCATCGTCTTGAACCACCCTGGTCAAA

TCTCCAACGGTTACACTCCCGTTTTGGATTGTCACACCGCTCACATTGCTTGCAAATTCG

CCGAAATCAAGGAGAAGGTCGAT

>MH401943

GTTGTCTTCGCTCCCGCTAACATCACCACTGAAGTCAAGT

CCGTAGAAATGCACCACGAAGCTCTCGTTGAAGCTGTTCCCGGTGACAACGTTGGTTTCA

ACGTTAAGAACGTCTCCGTCAAGGAATTGCGTCGTGGTTACGTCGCTGGTGACTCCAAGG

CTAGCCCCCCCAAGGGTGCTGCTGACTTCACCGCTCAAGTCATCGTCTTGAACCACCCTG

GTCAAATCTCCAACGGTTACACTCCCGTTTTGGATTGTCACACCGCTCACATTGCTTGCA

AATTCGCCGAAATCAAGGAGAAGGTCGAT

>MH401944

GTTGTCTTCGCTCCCGCTAACATCACCACTGAAGTCAAGTC

CGTAGAAATGCACCACGAAGCTCTCGTTGAAGCTGTTCCCGGTGACAACGTTGGTTTCAA

CGTTAAGAACGTCTCCGTCAAGGAATTGCGTCGTGGTTACGTCGCTGGTGACTCCAAGGC

TAGCCCCCCCAAGGGTGCTGCTGACTTCACCGCTCAAGTCATCGTCTTGAACCACCCTGG

TCAAATCTCCAACGGTTACACTCCCGTTTTGGATTGTCACACCGCTCACATTGCTTGCAA

ATTCGCCGAAATCAAGGAGAAGGTCGAT

>MH401945

GTTGTCTTCGCCCCCGCTAACATCACCACTGAAGT

CAAGTCCGTTGAAATGCACCACGAAGCTCTTGTTGAAGCCGTTCCCGGTGACAACGTTGG

TTTCAACGTTAAGAACGTCTCCGTCAAGGAATTGCGTCGTGGTTACGTCGCTGGTGACTC

CAAGGCTTCTCCCCCCAAGGGTGCTGCTGACTTCACCGCTCAAGTTATTGTGTTGAACCA

TCCTGGTCAAATCTCCAACGGTTATACCCCCGTCTTGGATTGTCACACCGCTCACATTGC

TTGCAAATTCGCTGAAATCAAGGAGAAGGTCGAT

>MH401946

GTTGTCTTCGCTCCCGCTAACATCACCACTGAAGTCAAGTCCGTAGAAATGCACCACGAA

GCTCTCGTTGAAGCTGTTCCCGGTGACAACGTTGGTTTCAACGTTAAGAACGTCTCCGTC

AAGGAATTGCGTCGTGGTTACGTCGCTGGTGACTCCAAGGCTAGCCCCCCCAAGGGTGCT

GCTGACTTCACCGCTCAAGTCATCGTCTTGAACCACCCTGGTCAAATCTCCAACGGTTAC

ACTCCCGTTTTGGATTGTCACACCGCTCACATTGCTTGCAAATTCGCCGAAATCAAGGAG

AAGGTCGAT

>MH401947

GTTGTCTTCGCTCCCGCTAACATCACCACTGAAGTCA

AGTCCGTTGAAATGCACCACGAAGCTCTCGTTGAAGCCGTCCCCGGTGACAACGTTGGTT

TCAACGTAAAGAACGTCTCCGTCAAGGAATTGCGTCGTGGTTACGTCGCTGGTGACTCCA

AGGCCAACCCCCCCAAGGGCGCCGCTGACTTCACCGCTCAAGTCATCGTCTTGAACCACC

CTGGTCAAATCTCCAACGGTTACACCCCCGTTTTGGATTGTCACACCGCTCACATTGCTT

GCAAATTCGCCGAAATCAAGGAGAAGGTCGAT

>MH401948

GTTGTCTTCGCTCCCGCTAACATCACCACTGAAGTCAAGT

CCGTTGAAATGCACCACGAAGCTCTCGTTGAAGCCGTCCCCGGTGACAACGTTGGTTTCA

ACGTAAAGAACGTCTCCGTTAAGGAATTGCGTCGTGGTTACGTCGCTGGTGACTCCAAGG

CCAACCCCCCCAAGGGCGCCGCTGACTTCACCGCTCAAGTCATCGTCTTGAACCACCCTG

GTCAAATCTCCAACGGTTACACCCCCGTTTTGGATTGTCACACCGCTCACATTGCTTGCA

AATTCGCCGAAATCAAGGAGAAGGTCGAT

>MH401949

GTTGTCTTCGCTCCCGCTAACATCACCAC

TGAAGTCAAGTCCGTTGAAATGCACCACGAAGCTCTCGTTGAAGCCGTCCCCGGTGACAA

CGTTGGTTTCAACGTAAAGAACGTCTCCGTCAAGGAATTGCGTCGTGGTTACGTCGCTGG

TGACTCCAAGGCCAACCCCCCCAAGGGCGCCGCTGACTTCACCGCTCAAGTCATCGTCTT

GAACCACCCTGGTCAAATCTCCAACGGTTACACCCCCGTTTTGGATTGTCACACCGCTCA

CATTGCTTGCAAATTCGCCGAAATCAAGGAGAAGGTCGAT

>MH401950

GTTGTCTTCGCTCCCGCTAACATCACCACTGAAG

TCAAGTCCGTTGAAATGCACCACGAAGCTCTCGTTGAAGCCGTCCCCGGTGACAACGTTG

GTTTCAACGTAAAGAACGTCTCCGTCAAGGAATTGCGTCGTGGTTACGTCGCTGGTGACT

CCAAGGCCAACCCCCCCAAGGGCGCCGCTGACTTCACCGCTCAAGTCATCGTCTTGAACC

ACCCTGGTCAAATCTCCAACGGTTACACCCCCGTTTTGGATTGTCACACCGCTCACATTG

CTTGCAAATTCGCCGAAATCAAGGAGAAGGTCGAT

>MH401951

GTTGTTTTCGCTCCCGCTAACATCACCACT

GAAGTCAAGTCCGTTGAAATGCACCACGAAGCTCTCGTTGAAGCCGTCCCCGGTGACAAC

GTTGGTTTCAACGTAAAGAACGTCTCCGTCAAGGAATTGCGTCGTGGTTACGTCGCTGGT

GACTCCAAGGCTAACCCCCCCAAGGGCGCCGCTGACTTCACCGCTCAAGTCATCGTCTTG

AACCACCCTGGTCAAATCTCCAACGGTTACACCCCCGTTTTGGATTGTCACACCGCTCAC

ATTGCTTGCAAATTCGCCGAAATCAAGGAGAAGGTCGAT

>MH401952

GTTGTCTTCGCTCCCGCTAACATCACCACTGAAGTCAAGTCCGTTGA

AATGCACCACGAAGCTCTCGTTGAAGCCGTCCCCGGTGACAACGTTGGTTTCAACGTAAA

GAACGTCTCCGTCAAGGAATTGCGTCGTGGTTACGTCGCTGGTGACTCCAAGGCTAACCC

CCCCAAGGGCGCCGCTGACTTCACCGCTCAAGTCATCGTCTTGAACCACCCTGGTCAAAT

CTCCAACGGTTACACCCCCGTTTTGGATTGTCACACCGCTCACATTGCTTGCAAATTCGC

CGAAATCAAGGAGAAGGTCGAT

>MH401953

GTTGTCTTCGCTCCCGCTAACATCACCACTGAAGTCAAGT

CCGTTGAAATGCACCACGAAGCTCTCGTTGAAGCCGTCCCCGGTGACAACGTTGGTTTCA

ACGTAAAGAACGTCTCCGTTAAGGAATTGCGTCGTGGTTACGTCGCTGGTGACTCCAAGG

CCAACCCCCCCAAGGGCGCCGCTGACTTCACCGCTCAAGTCATCGTCTTGAACCACCCTG

GTCAAATCTCCAACGGTTACACCCCCGTTTTGGATTGTCACACCGCTCACATTGCTTGCA

AATTCGCCGAAATCAAGGAGAAGGTCGAT

>MH401954

GTTGTCTTCGCTCCCGCTAACATCACCACTGAAGTCAAGTCCGTTG

AAATGCACCACGAAGCTCTCGTTGAAGCCGTCCCCGGTGACAACGTTGGTTTCAACGTAA

AGAACGTCTCCGTCAAGGAATTGCGTCGTGGTTACGTCGCTGGTGACTCCAAGGCCAACC

CCCCCAAGGGCGCCGCTGACTTCACCGCTCAAGTCATCGTCTTGAACCACCCTGGTCAAA

TCTCCAACGGTTACACCCCCGTTTTGGATTGTCACACCGCTCACATTGCTTGCAAATTCG

CCGAAATCAAGGAGAAGGTCGAT

>MH401955

GTTGTCTTCGCCCCCGCTAACATCACCACTGAAGTCAAGTC

CGTTGAAATGCACCACGAAGCTCTCGTTGAAGCCGTCCCCGGTGACAACGTTGGTTTCAA

CGTAAAGAACGTCTCCGTCAAGGAATTGCGTCGTGGTTACGTCGCTGGTGACTCCAAGGC

CAACCCCCCCAAGGGCGCCGCTGACTTCACCGCTCAAGTCATCGTCTTGAACCACCCTGG

TCAAATCTCCAACGGTTACACCCCCGTTTTGGATTGTCACACCGCTCACATTGCTTGCAA

ATTCGCCGAAATCAAGGAGAAGGTCGAT

>MH401956

GTTGTCTTCGCTCCCGCTAACATCACCACTGAAGTCAAGT

CCGTTGAAATGCACCACGAAGCTCTCGTTGAAGCCGTCCCCGGTGACAACGTTGGTTTCA

ACGTAAAGAACGTCTCCGTCAAGGAATTGCGTCGTGGTTACGTCGCTGGTGACTCCAAGG

CCAACCCCCCCAAGGGCGCCGCTGACTTCACCGCTCAAGTCATCGTCTTGAACCACCCTG

GTCAAATCTCCAACGGTTACACCCCCGTTTTGGATTGTCACACCGCTCACATTGCTTGCA

AATTCGCCGAAATCAAGGAGAAGGTCGAT

>MH401957

GTTGTCTTCGCTCCCG

CTAACATCACCACTGAAGTCAAGTCCGTTGAAATGCACCACGAAGCTCTCGTTGAAGCCG

TCCCCGGTGACAACGTTGGTTTCAACGTAAAGAACGTCTCCGTCAAGGAATTGCGTCGTG

GTTACGTCGCTGGTGACTCCAAGGCCAACCCCCCCAAGGGCGCCGCTGACTTCACCGCTC

AAGTCATCGTCTTGAACCACCCTGGTCAAATCTCCAACGGTTACACCCCCGTTTTGGATT

GTCACACCGCTCACATTGCTTGCAAATTCGCCGAAATCAAGGAGAAGGTCGAT

>MH401958

GTTGTCTTCGCTCCCGCTAACATCACCACTGAAGTCAAGTCCG

TAGAAATGCACCACGAAGCTCTCGTTGAAGCTGTTCCCGGTGACAACGTTGGTTTCAACG

TTAAGAACGTCTCCGTCAAGGAATTGCGTCGTGGTTACGTCGCTGGTGACTCCAAGGCTA

GCCCCCCCAAGGGTGCTGCTGACTTCACCGCTCAAGTCATCGTCTTGAACCACCCTGGTC

AAATCTCCAACGGTTACACTCCCGTTTTGGATTGTCACACCGCTCACATTGCTTGCAAAT

TCGCCGAAATCAAGGAGAAGGTCGAT

>MH401959

GTTGTCTTCGCTCCCGCTAACATCACCAC

TGAAGTCAAGTCCGTAGAAATGCACCACGAAGCTCTCGTTGAAGCCGTTCCCGGTGACAA

CGTTGGTTTCAACGTTAAGAACGTCTCCGTCAAGGAATTGCGTCGTGGTTACGTCGCTGG

TGACTCCAAGGCTAGCCCCCCCAAGGGTGCTGCTGACTTCACCGCTCAAGTCATCGTCTT

GAACCACCCTGGTCAAATCTCCAACGGTTACACTCCCGTTTTGGATTGTCACACCGCTCA

CATTGCTTGCAAATTCGCCGAAATCAAGGAGAAGGTCGAT

>MH401960

GTTGTCTTCGCTCCCGCTAACATCACCACTGAAGTCAAAT

CCGTAGAAATGCACCACGAAGCTCTCGTTGAAGCCGTCCCCGGTGACAACGTTGGTTTCA

ACGTTAAGAACGTCTCCGTCAAGGAATTGCGTCGTGGTTACGTCGCTGGTGACTCCAAGG

CTAGCCCCCCCAAGGGTGCTGCTGACTTCACCGCTCAAGTCATCGTCTTGAACCACCCTG

GTCAAATCTCCAACGGTTACACTCCCGTTTTGGATTGTCACACCGCTCACATTGCTTGCA

AATTCGCCGAAATCAAGGAGAAGGTCGAT

>MH401961

GTTGTCTTCGCTCCCGCTAACATCACCACTGAAGTCAAGT

CCGTAGAAATGCACCACGAAGCTCTCGTTGAAGCTGTTCCCGGTGACAACGTTGGTTTCA

ACGTTAAGAACGTCTCCGTCAAGGAATTGCGTCGTGGTTACGTCGCTGGTGACTCCAAGG

CTAGCCCCCCCAAGGGTGCTGCTGACTTCACCGCTCAAGTCATCGTCTTGAACCACCCTG

GTCAAATCTCCAACGGTTACACTCCCGTTTTGGATTGTCACACCGCTCACATTGCTTGCA

AATTCGCCGAAATCAAGGAGAAGGTCGAT

>MH401962

GTTGTCTTCGCTCCCGCTAA

CATCACCACTGAAGTCAAGTCCGTTGAAATGCACCACGAAGCTCTCGTTGAAGCCGTTCC

CGGTGACAACGTTGGTTTCAACGTAAAGAACGTCTCCGTCAAGGAATTGCGTCGTGGTTA

CGTCGCTGGTGACTCCAAGGCCAGCCCCCCCAAGGGTGCCGCTGACTTCACCGCTCAAGT

CATCGTCTTGAACCATCCCGGTCAAATCTCCAACGGTTACACTCCCGTTTTGGATTGTCA

CACCGCTCACATTGCTTGCAAATTCGCCGAAATCAAGGAGAAGGTCGAT

>MH401963

GTTGTCTTCGCTCCCGCTAACATCACCACTG

AAGTCAAGTCCGTAGAAATGCACCACGAAGCTCTCGTTGAAGCTGTTCCCGGTGACAACG

TTGGTTTCAACGTTAAGAACGTCTCCGTCAAGGAATTGCGTCGTGGTTACGTCGCTGGTG

ACTCCAAGGCTAGCCCCCCCAAGGGTGCTGCTGACTTCACCGCTCAAGTCATCGTCTTGA

ACCACCCTGGTCAAATCTCCAACGGTTACACTCCCGTTTTGGATTGTCACACCGCTCACA

TTGCTTGCAAATTCGCCGAAATCAAGGAAAAGGTCGAT

>MH401964

GTTGTCTTCGCTCCCGCTAACATCACCACTGAAGT

CAAATCCGTAGAAATGCACCACGAAGCTCTCGTTGAAGCCGTCCCCGGTGACAACGTTGG

TTTCAACGTTAAGAACGTCTCCGTCAAGGAATTGCGTCGTGGTTACGTCGCTGGTGACTC

CAAGGCTAGCCCCCCCAAGGGTGCTGCTGACTTCACCGCTCAAGTCATCGTCTTGAACCA

CCCTGGTCAAATCTCCAACGGTTACACTCCCGTTTTGGATTGTCACACCGCTCACATTGC

TTGCAAATTCGCCGAAATCAAGGAGAAGGTCGAT

>MH401965

GTTGTCTTCGCTCCCGCTAACATCACCA

CTGAAGTCAAGTCCGTAGAAATGCACCACGAAGCTCTCGTTGAAGCTGTTCCCGGTGACA

ACGTTGGTTTCAACGTTAAGAACGTCTCCGTCAAGGAATTGCGTCGTGGTTACGTCGCTG

GTGACTCCAAGGCTAGCCCCCCCAAGGGTGCTGCTGACTTCACCGCTCAAGTCATCGTCT

TGAACCACCCTGGTCAAATCTCCAACGGTTACACTCCCGTTTTGGATTGTCACACCGCTC

ACATTGCTTGCAAATTCGCCGAAATCAAGGAGAAGGTCGAT

>MH401966

GTTGTCTTCGCTCCCGCTAACATCACCACTGAAG

TCAAGTCCGTAGAAATGCACCACGAAGCTCTCGTTGAAGCTGTTCCCGGTGACAACGTTG

GTTTCAACGTTAAGAACGTCTCCGTCAAGGAATTGCGTCGTGGTTACGTCGCTGGTGACT

CCAAGGCTAGCCCCCCCAAGGGTGCTGCTGACTTCACCGCTCAAGTCATCGTCTTGAACC

ACCCTGGTCAAATCTCCAACGGTTACACTCCCGTTTTGGATTGTCACACCGCTCACATTG

CTTGCAAATTCGCCGAAATCAAGGAGAAGGTCGAT

>MH401967

GTTGTCTTCGCTCCCGCTAACATCACCACTGAAGTC

AAGTCCGTAGAAATGCACCACGAAGCTCTCGTTGAAGCTGTTCCCGGTGACAACGTTGGT

TTCAACGTTAAGAACGTCTCCGTCAAGGAATTGCGTCGTGGTTACGTCGCTGGTGACTCC

AAGGCTAGCCCCCCCAAGGGTGCTGCTGACTTCACCGCTCAAGTCATCGTCTTGAACCAC

CCTGGTCAAATCTCCAACGGTTACACTCCCGTTTTGGATTGTCACACCGCTCACATTGCT

TGCAAATTCGCCGAAATCAAGGAGAAGGTCGAT

>MH401968

GTTGTCATCGCTCCCGCTAACATCACCACTGAAGTCAAGTCCGTTGAAATGCACCACGAA

GCTCTCGTTGAAGCCGTCCCCGGTGACAACGTTGGTTTCAACGTAAAGAACGTCTCCGTC

AAGGAATTGCGTCGTGGTTACGTCGCTGGTGACTCCAAGGCCAACCCCCCCAAGGGTGCC

GCTGACTTCACCGCTCAAGTCATCGTCTTGAACCATCCCGGTCAAATCTCCAACGGTTAC

ACTCCCGTTTTGGATTGTCACACCGCTCACATTGCTTGCAAATTCGCCGAAATCAAGGAG

AAGGTCGAT

>MH401969

GTTGTCATCGCTCCCGCTAACATCACCACTGAAGTCAAATC

CGTAGAAATGCACCACGAAGCTCTCGTTGAAGCCGTCCCCGGTGACAACGTTGGTTTCAA

CGTTAAGAACGTCTCCGTCAAGGAATTGCGTCGTGGTTACGTCGCTGGTGACTCCAAGGC

TAGCCCCCCCAAGGGTGCTGCTGACTTCACCGCTCAAGTCATCGTCTTGAACCACCCTGG

TCAAATCTCCAACGGTTACACTCCCGTTTTGGATTGTCACACCGCTCACATTGCTTGCAA

ATTCGCCGAAATCAAGGAGAAGGTCGAT

>MH588594

GTTGTCTTTGCCCCAGCGAACATCACCACTGAAGTTAAGTCC

GTTGAAATGCATCACGAAGCTCTCATCGAGGCTGTTCCCGGTGACAATGTTGGCTTCAAC

GTCAAGAACGTGTCCGTCAAGGAATTGCGTCGTGGCTATGTTGCCGGTGACACCAAGAAC

AACCCACCCCGCGGTACTGCCGATTTCAACGCTCAGGTTATTGTATTGAACCATCCCGGT

CAAATTGCCAACGGTTACACTCCAGTCTTGGATTGCCACACCGCTCACATTGCCTGCAAA

TTCGCTGAGATCAAGGAGAAGGTCGAT

>MH588595

GTTGTATTTGCCCCCGCTAACATCACCACTGAAGTTAAG

TCCGTGGAAATGCATCACGAAGCCCTCACCGAAGCCGTACCCGGTGACAACGTTGGTTTC

AACGTTAAGAACGTCTCCGTCAAGGAATTGCGTCGTGGTTATGTTGCTGGTGACTCCAAG

GCCAGCCCCCCCAAGGGTGCTGCTGATTTCACCGCCCAAGTTATTGTGTTGAACCATCCC

GGTCAAATCTCCCAGGGTTATACCCCCGTGTTGGATTGCCACACCGCTCACATTGCTTGC

AAGTTCGCTGAAATCAAGGAGAAGGTCGAT

>MH588596

GTTGTCTTCGCTCCCGCTAACATCACCACTGAAGTTAAG

TCCGTTGAAATGCACCACGAAGCTCTCGCTGAAGCCGTACCCGGTGACAACGTTGGTTTC

AACGTAAAGAACGTCTCCGTCAAGGAATTGCGTCGTGGTTACGTTGCTGGTGACTCCAAG

GCTAACCCACCCAAGGGTGCTGCTGACTTCACCGCTCAAGTCATTGTCTTGAACCACCCT

GGTCAAATCTCCAACGGTTACACCCCCGTTTTGGATTGTCACACCGCTCACATTGCTTGC

AAATTCGCTGAAATCAAGGAGAAGGTCGAT

>MH588597

GTTGTCTTCGCTCCCGCTAACATCACCACTGAAGT

CAAGTCCGTTGAAATGCACCACGAAGCTCTCGTTGAAGCCGTCCCCGGTGACAACGTTGG

TTTCAACGTAAAGAACGTCTCCGTCAAGGAATTGCGTCGTGGTTACGTCGCTGGTGACTC

CAAGGCTAACCCCCCCAAGGGCGCCGCTGACTTCACCGCTCAAGTCATCGTCTTGAACCA

CCCTGGTCAAATCTCCAACGGTTACACCCCCGTTTTGGATTGTCACACCGCTCACATTGC

TTGCAAATTCGCCGAAATCAAGGAGAAGGTCGAT

>MH588598

GTTGTCTTCGCTCCCGCTAACATCACCACTGAAGTCAAGTCCGTTGAAATGCACCACGA

AGCTCTCGTTGAAGCCGTCCCCGGTGACAACGTTGGTTTCAACGTAAAGAACGTCTCCGT

CAAGGAATTGCGTCGTGGTTACGTCGCTGGTGACTCCAAGGCCAACCCCCCCAAGGGCGC

CGCTGACTTCACCGCTCAAGTCATCGTCTTGAACCACCCTGGTCAAATCTCCAACGGTTA

CACCCCCGTTTTGGATTGTCACACCGCTCACATTGCTTGCAAATTCGCCGAAATCAAGGA

GAAGGTCGAT

>MH588599

GTTGTCTTCGCTCCCGCTAACATCACCAC

TGAAGTCAAATCCGTAGAAATGCACCACGAAGCTCTCGTTGAAGCCGTCCCCGGTGACAA

CGTTGGTTTCAACGTTAAGAACGTCTCCGTCAAGGAATTGCGTCGTGGTTACGTCGCTGG

TGACTCCAAGGCTAGCCCCCCCAAGGGTGCTGCTGACTTCACCGCTCAAGTCATCGTCTT

GAACCACCCTGGTCAAATCTCCAACGGTTACACTCCCGTTTTGGATTGTCACACCGCTCA

CATTGCTTGCAAATTCGCCGAAATCAAGGAGAAGGTCGAT

>MH588600

GTTGTCTTCGCTCCCGCTAACATCACCAC

TGAAGTCAAATCCGTAGAAATGCACCACGAAGCTCTCGTTGAAGCCGTCCCCGGTGACAA

CGTTGGTTTCAACGTTAAGAACGTCTCCGTCAAGGAATTGCGTCGTGGTTACGTCGCTGG

TGACTCCAAGGCTAGCCCCCCCAAGGGTGCTGCTGACTTCACCGCTCAAGTCATCGTCTT

GAACCACCCTGGTCAAATCTCCAACGGTTACACTCCCGTTTTGGATTGTCACACCGCTCA

CATTGCTTGCAAATTCGCCGAAATCAAGGAGAAGGTCGAT

>MH588601

GTTGTCTTCGCTCCCGCTAACATCACCAC

TGAAGTCAAGTCCGTAGAAATGCACCACGAAGCTCTCGTTGAAGCTGTTCCCGGTGACAA

CGTTGGTTTCAACGTTAAGAACGTCTCCGTCAAGGAATTGCGTCGTGGTTACGTCGCTGG

TGACTCCAAGGCTAGCCCCCCCAAGGGTGCTGCTGACTTCACCGCTCAAGTCATCGTCTT

GAACCACCCTGGTCAAATCTCCAACGGTTACACTCCCGTTTTGGATTGTCACACCGCTCA

CATTGCTTGCAAATTCGCCGAAATCAAGGAGAAGGTCGAT

>MH588602

GTTGTCTTCGCTCCCGCTAACATCACCAC

TGAAGTCAAGTCCGTAGAAATGCACCACGAAGCTCTCGTTGAAGCTGTTCCCGGTGACAA

CGTTGGTTTCAACGTTAAGAACGTCTCCGTCAAGGAATTGCGTCGTGGTTACGTCGCTGG

TGACTCCAAGGCTAGCCCCCCCAAGGGTGCTGCTGACTTCACCGCTCAAGTCATCGTCTT

GAACCACCCTGGTCAAATCTCCAACGGTTACACTCCCGTTTTGGATTGTCACACCGCTCA

CATTGCTTGCAAATTCGCCGAAATCAAGGAGAAGGTCGAT

>MH588603

GTTGTCTTCGCTCCCGCTAACATCACCACTGAAGTCAAG

TCCGTAGAAATGCACCACGAAGCTCTCGTTGAAGCTGTTCCCGGTGACAACGTTGGTTTC

AACGTTAAGAACGTCTCCGTCAAGGAATTGCGTCGTGGTTACGTCGCTGGTGACTCCAAG

GCTAGCCCCCCCAAGGGTGCTGCTGACTTCACCGCTCAAGTCATCGTCTTGAACCACCCT

GGTCAAATCTCCAACGGTTACACTCCCGTTTTGGATTGTCACACCGCTCACATTGCTTGC

AAATTCGCCGAAATCAAGGAGAAGGTCGAT

>MH588604

GTTGTCTTCGCTCCCGCTAACATCACCACTGAAATCAAG

TCCGTAGAAATGCACCACGAAGCTCTCGTTGAAGCTGTTCCCGGTGACAACGTTGGTTTC

AACGTTAAGAACGTCTCCGTCAAGGAATTGCGTCGTGGTTACGTCGCTGGTGACTCCAAG

GCTAGCCCCCCCAAGGGTGCTGCTGACTTCACCGCTCAAGTCATCGTCTTGAACCACCCT

GGTCAAATCTCCAACGGTTACACTCCCGTTTTGGATTGTCACACCGCTCACATTGCTTGC

AAATTCGCCGAAATCAAGGAGAAGGTCGAT

>MH605069

GTTGTCTTCGCTCCCGCTAACATCACCAC

TGAAGTTAAGTCCGTTGAAATGCACCACGAAGCTCTCGCTGAAGCCGTACCCGGTGACAA

CGTTGGTTTCAACGTAAAGAACGTCTCCGTCAAGGAATTGCGTCGTGGTTACGTTGCTGG

TGACTCCAAGGCTAACCCACCCAAGGGTGCTGCTGACTTCACCGCTCAAGTCATTGTCTT

GAACCACCCTGGTCAAATCTCCAACGGTTACACCCCCGTTTTGGATTGTCACACCGCTCA

CATTGCTTGCAAATTCGCTGAAATCAAGGAGAAGGT

**PER sequences**

>MH401837

TAAAGGCACTAGTATGTTGATCGTCATATTCGCCAAACTTATAAAAAGTGCTT

ACAAAGTTCCAAATGAACTTTCAACACTCAAAAGTCCAAAGTTTTCGATTCGACACTCCG

CTGCAGGAATTGTGTCTCACGTGGACAGCACTGCTGTCTCTGCTCTTGGTTATTTACCTC

AACATATGATGGGACGTCCAATTCTGGACTTTTACCATCCTGAAGACCTGGATGCCTTGA

AAGATG

>MH401838

GGATGCACGCA

GTACCCTAAGTCCGGTTCAGTGCTTTGAGGGCAGCGGTGGAAGTGGTTCATCGGGAAATT

TTACTTCGGGCAGTAACTTAAATATGGGCAGTGTGACTAATACCAGCAACACCGGTACTG

GTACTTCCTCAGGAAGTGTTCCCATGGTCACGTTGACGGAATCTCTACTAAATAAACATA

ACGATGAAATGGAAAAGTTTATGCTTAAGAAGCATAGAGAATCTCGAGGACGAAGTGGCG

AGAAGAACAAAAAGAATTCTGAAAAGATAATGGAGTACACTGGCCCGGGACACGGAATCA

AAAGAGTTGGTTCACA

>MH401839

TGAAGCACGCAGTACCCTGAGTCCGGTTCAGTGCTTTGAGGGTAGCGGTGGTAG

TGGATCCTCAGGAAATTTTACTTCGGGTAGCAACTTGAATATGGGCAGTGTTACTAATAC

AAGCAATACCGGTACAGGCACTTCATCGGGAAGTGTACCCATGGTTACGTTGACGGAATC

TCTGCTAAATAAACACAACGATGAAATGGAAAAGTTTATGCTAAAGAAACACAGAGAATC

TCGCGGACGAAGTGGTGAGAAGAATAAAAAGAACTCGGAAAAGATAATGGAGTATACTGG

TCCTGGGCATGGAATAAAGAGAGTTGGCTCACA

>MH401840

TGAAGCACGCAGTACCCTGAGTCCGGTTCAGTGCTTTGAGGGTAGCGGTGGTAGTGGAT

CCTCGGGAAATTTTACTTCGGGTAGCAACTTGAATATGGGCAGTGTTACTAATACAAGCA

ATACCGGTACAGGCACTTCATCGGGAAGTGTACCCATGGTTACGTTGACGGAATCTCTAC

TAAATAAACACAACGATGAAATGGAAAAGTTTATGCTAAAGAAACACAGAGAATCTCGCG

GACGAAGTGGTGAGAAGAATAAAAAGAACTCGGAAAAGATAATGGAGTATACTGGTCCTG

GGCATGGAATAAAGAGAGTTGGCTCACA

>MH401841

TGAAGCACGCAGTACCCTGAGTCCGGTTCAGTGCTTTGAGGGTAGCGGTGGTAGT

GGATCCTCAGGAAATTTTACTTCGGGTAGCAACTTGAATATGGGCAGTGTTACTAATACA

AGCAATACCGGTACAGGCACTTCATCGGGAAGTGTACCCATGGTTACGTTGACGGAATCT

CTACTAAATAAACACAACGATGAAATGGAAAAGTTTATGCTAAAGAAACACAGAGAATCT

CGCGGACGAAGTGGTGAGAAGAATAAAAAGAACTCGGAAAAGATAATGGAGTATACTGGT

CCTGGGCATGGAATAAAGAGAGTTGGCTCACA

>MH401842

TGAAGCACGCAGTACCCTGAGTCCGGTTCAGTGCTTTGAGGGTAGCGGTGGTAGTG

GATCCTCAGGAAATTTTACTTCGGGTAGCAACTTGAATATGGGCAGTGTTACTAATACAA

GCAATACCGGTACAGGCACTTCATCGGGAAGTGTACCCATGGTTACGTTGACGGAATCTC

TGCTAAATAAACACAACGATGAAATGGAAAAGTTTATGCTAAAGAAACACAGAGAATCTC

GCGGACGAAGTGGTGAGAAGAATAAAAAGAACTCGGAAAAGATAATGGAGTATACTGGTC

CTGGGCATGGAATAAAGAGAGTTGGCTCACA

>MH401843

TGAAGCACGCAGTACCCTGAGTCCGGTTCAGTGCTTTGAGGGTAGCGGTGGTAGTGG

ATCCTCGGGAAATTTTACTTCGGGTAGCAACTTGAATATGGGCAGTGTTACTAATACAAG

CAATACCGGTACAGGCACTTCATCGGGAAGTGTACCCATGGTTACGTTGACGGAATCTCT

ACTAAATAAACACAACGATGAAATGGAAAAGTTTATGCTAAAGAAACACAGAGAATCTCG

CGGACGAAGTGGTGAGAAGAATAAAAAGAACTCGGAAAAGATAATGGAGTATACTGGTCC

TGGGCATGGAATAAAGAGAGTTGGCTCACA

>MH401844

CGATGCACGCAGTACCCTAAGTCCGGTTCAGTGCTTTGAGGGCAGCGGTGGAAGT

GGTTCATCGGGAAATTTTACTTCGGGCAGTAACTTAAATATGGGCAGTGTGACTAATACC

AGCAACACCGGTACTGGTACTTCCTCTGGAAGTGTTCCCATGGTCACGTTGACCGAATCT

CTACTAAATAAACATAACGATGAAATGGAAAAGTTTATGCTTAAGAAGCATAGAGAATCT

CGAGGACGAAGTGGCGAGAAGAACAAAAAGAATTCTGAAAAGATAATGGAGTACACTGGC

CCGGGACACGGAATCAAAAGAGTTGGTTCACA

>MH401845

CGATGCACGCAGTACCCTAAGTCCGGTTCAGTGCTTTGAGGGCAGCGGTGGAAGT

GGTTCATCGGGAAATTTTACTTCGGGCAGTAACTTAAATATGGGCAGTGTGACTAATACC

AGCAACACCGGTACTGGTACTTCCTCAGGAAGTGTTCCCATGGTCACGTTGACCGAATCT

CTACTAAATAAACATAACGATGAAATGGAAAAGTTTATGCTTAAGAAGCATAGAGAATCT

CGAGGACGAAGTGGCGAGAAGAACAAAAAGAATTCTGAAAAGATAATGGAGTACACTGGC

CCGGGACACGGAATCAAAAGAGTTGGTTCACA

>MH401846

CGATGCACGCAGTACCCTAAGTCCGGTTCAGTGCTTTGAGGGCAGCGGTGGAAGT

GGTTCATCGGGAAATTTTACTTCGGGCAGTAACTTAAATATGGGCAGTGTGACTAATACC

AGCAACACCGGTACTGGTACTTCCTCAGGAAGTGTTCCCATGGTCACGTTGACGGAATCT

CTACTAAATAAACATAACGATGAAATGGAAAAGTTTATGCTTAAGAAGCATAGAGAATCT

CGAGGACGAAGTGGCGAGAAGAACAAAAAGAATTCTGAAAAGATAATGGAGTACACTGGC

CCGGGACACGGAATCAAAAGAGTTGGTTCACA

>MH401847

CGATGCACGCAGTACCCTAAGTCCGGTTCAGTGCTTTGAGGGCAGCGGTGGAAG

TGGTTCGTCGGGAAATTTTACTTCGGGCAGTAACTTAAATATGGGCAGTGTGACTAATAC

CAGCAACACCGGTACTGGTACTTCCTCTGGAAGTGTTCCCATGGTCACGTTGACCGAATC

TCTACTAAATAAACATAACGATGAAATGGAAAAGTTTATGCTTAAGAAGCATAGAGAATC

TCGAGGACGAAGTGGCGAGAAGAACAAAAAGAATTCTGAAAAGATAATGGAGTACACTGG

CCCGGGACACGGAATCAAAAGAGTTGGTTCACA

>MH401848

GGATGCACGCAGTACCCTAAGTCCGGTTCAGTGCTTTGAGGGCAGCGGTGGAAG

TGGTTCATCGGGAAATTTTACTTCGGGCAGTAACTTAAATATGGGCAGTGTGACTAATAC

CAGCAACACCGGTACTGGTACTTCCTCAGGAAGTGTTCCCATGGTCACGTTGACCGAATC

TCTACTAAATAAACATAACGATGAAATGGAAAAGTTTATGCTTAAGAAGCATAGAGAATC

TCGAGGACGAAGTGGCGAGAAGAACAAAAAGAATTCTGAAAAGATAATGGAGTACACTGG

CCCGGGACACGGAATCAAAAGAGTTGGTTCACA

>MH401849

TGAAGCACGCAGTACCCTGAGTCCGGTTCAGTGCTTTGAGGGTAGCGGTGGTAGTGGATC

CTCAGGAAATTTTACTTCGGGCAGCAACTTGAATATGGGCAGTGTTACTAATACAAGCAA

TACCGGTACAGGCACTTCATCGGGAAGTGTACCCATGGTTACGTTGACGGAATCTCTACT

AAATAAACACAACGATGAAATGGAAAAGTTTATGCTAAAGAAACACAGAGAATCTCGCGG

ACGCAGTGGTGAGAAGAATAAAAAGAACTCGGAAAAGATAATGGAGTATTCTGGTCCTGG

GCATGGAATAAAGAGAGTTGGCTCACA

>MH401850

CGATGCACGCAGTACCCTAAGTCCGGTTCAGTGCTTTGAGGGCAGCGGTGGAAGT

GGTTCATCGGGAAATTTTACTTCGGGCAGTAACTTAAATATGGGCAGTGTGACTAATACC

AGCAACACCGGTACTGGTACTTCCTCTGGAAGTGTTCCCATGGTCACGTTGACCGAATCT

CTACTAAATAAACATAACGATGAAATGGAAAAGTTTATGCTTAAGAAGCATAGAGAATCT

CGAGGACGAAGTGGCGAGAAGAACAAAAAGAATTCTGAAAAGATAATGGAGTACACTGGC

CCGGGACACGGAATCAAAAGAGTTGGTTCACA

>MH401851

GGATGCACGCAGTACCCTAAGTCCGGTTCAGTGCTTTGAGGGCAGCGGTGGAAGT

GGTTCATCGGGAAATTTTACTTCGGGCAGTAACTTAAATATGGGCAGTGTGACTAATACC

AGCAACACCGGTACTGGTACTTCCTCAGGAAGTGTTCCCATGGTCACGTTGACGGAATCT

CTACTAAATAAACATAACGATGAAATGGAAAAGTTTATGCTTAAGAAGCATAGAGAATCT

CGAGGACGAAGTGGCGAGAAGAACAAAAAGAATTCTGAAAAGATAATGGAGTACACTGGC

CCGGGACACGGAATCAAAAGAGTTGGTTCACA

>MH401852

GGATGCACGCAGTACCCTAAGTCCGGTTCAGTGCTTTGAGGGCAGCGGTGGAAGT

GGTTCATCGGGAAATTTTACTTCGGGCAGTAACTTAAATATGGGCAGTGTGACTAATACC

AGCAACACCGGTACTGGTACTTCCTCAGGAAGTGTTCCCATGGTCACGTTGACGGAATCT

CTACTAAATAAACATAACGATGAAATGGAAAAGTTTATGCTTAAGAAGCATAGAGAATCT

CGAGGACGAAGTGGCGAGAAGAACAAAAAGAATTCTGAAAAGATAATGGAGTACACTGGC

CCGGGACACGGAATCAAAAGAGTTGGTTCACA

>MH401853

TGATGCACGCAGTGCTTTGAGTCCGGTTCAGTGCTTTGAAGGCAGCGGTGGTAG

TGGTTCATCGGGAAATTTTACTTCGGGCAGTAACTTGAATATGGGCAGTGTGACTAATAC

CAGCAACACTGGTACCGGCACTTCCTCGGGAAGTGTTCCCATGGTTACATTAACGGAATC

TCTGCTAAATAAGCATAACGATGAAATGGAAAAATTTATGCTAAAAAAGCACAGAGAATC

TCGTGGACGAAGTGGCGAGAAGAATAAAAAGACTTCTGAAAAGATAATGGAATATACTGG

ACCGGGGCATGGAATCAAAAGAGTTGGATCACA

>MH401854

TGATGCACGCAGTGCTTTGAGTCCGGTTCAGTGCTTTGAAGGCAGCGGTGGTAG

TGGTTCATCGGGAAATTTTACTTCGGGCAGTAACTTGAATATGGGCAGTGTGACTAATAC

CAGCAACACTGGTACCGGCACTTCCTCGGGAAGTGTTCCCATGGTTACATTAACGGAATC

TCTGCTAAATAAGCATAACGATGAAATGGAAAAATTTATGCTAAAAAAGCACAGAGAATC

TCGTGGACGAAGTGGCGAGAAGAATAAAAAGACTTCTGAAAAGATAATGGAATATACTGG

ACCGGGGCATGGAATCAAAAGAGTTGGATCACA

>MH401855

TGAAGCACGCAGTACCCTGAGTCCGGTTCAGTGCTTTGAGGGTAGCGGTGGTAGTGGATC

CTCAGGAAATTTTACTTCGGGCAGCAACTTGAATATGGGCAGTGTTACTAATACAAGCAA

TACCGGTACAGGCACTTCATCGGGAAGTGTACCCATGGTTACGTTGACGGAATCTCTACT

AAATAAACACAACGATGAAATGGAAAAGTTTATGCTAAAGAAACACAGAGAATCTCGCGG

ACGCAGTGGTGAGAAGAATAAAAAGAACTCGGAAAAGATAATGGAGTATACTGGTCCTGG

GCATGGAATAAAGAGAGTTGGCTCACA

>MH401856

TGAAGCACGCAGTACCCTGAGTCCGGTTCAGTGCTTTGAGGGTAGCGGTGGTAGT

GGATCCTCAGGAAATTTTACTTCGGGCAGCAACTTGAATATGGGCAGTGTTACTAATACA

AGCAATACCGGTACAGGCACTTCATCGGGAAGTGTACCCATGGTTACGTTGACGGAATCT

CTACTAAATAAACACAACGATGAAATGGAAAAGTTTATGCTAAAGAAACACAGAGAATCT

CGCGGACGCAGTGGTGAGAAGAATAAAAAGAACTCGGAAAAGATAATGGAGTATACTGGT

CCTGGGCATGGAATAAAGAGAGTTGGCTCACA

>MH401857

TGAAGCACGCAGTACCCTGAGTCCGGTTCAGTGCTTTGAGGGTAGCGGTGGTAGTGGATC

CTCAGGAAATTTTACTTCGGGCAGCAACTTGAATATGGGCAGTGTTACTAATACAAGCAA

TACCGGTACAGGCACTTCATCGGGAAGTGTACCCATGGTTACGTTGACGGAATCTCTACT

AAATAAACACAACGATGAAATGGAAAAGTTTATGCTAAAGAAACACAGAGAATCTCGCGG

ACGAAGTGGTGAGAAGAATAAAAAGAACTCGGAAAAGATAATGGAGTATACTGGTCCTGG

GCATGGAATAAAGAGAGTTGGCTCACA

>MH401858

TGAAGCACGCAGTACCCTGAGTCCGGTTCAGTGCTTTGAGGGTAGCGGTGGTAGT

GGATCCTCAGGAAATTTTACTTCGGGTAGCAACTTGAATATGGGCAGTGTTACTAATACA

AGCAATACCGGTACAGGCACTTCATCGGGAAGTGTACCCATGGTTACGTTGACGGAATCT

CTACTAAATAAACACAACGATGAAATGGAAAAGTTTATGCTAAAGAAACACAGAGAATCT

CGCGGACGAAGTGGTGAGAAGAATAAAAAGAACTCGGAAAAGATAATGGAGTATACTGGT

CCTGGGCATGGAATAAAGAGAGTTGGCTCACA

>MH401859

TGAAGCACGCAGTACCCTGAGTCCGGTTCAGTGCTTTGAGGGTAGCGGTGGT

AGTGGATCCTCGGGAAATTTTACTTCGGGTAGCAACTTGAATATGGGCAGTGTTACTAAT

ACAAGCAATACCGGTACAGGCACTTCATCGGGAAGTGTACCCATGGTTACGTTGACGGAA

TCTCTACTAAATAAACACAACGATGAAATGGAAAAGTTTATGCTAAAGAAACACAGAGAA

TCTCGCGGACGAAGTGGTGAGAAGAATAAAAAGAACTCGGAAAAGATAATGGAGTATACT

GGTCCTGGGCATGGAATAAAGAGAGTTGGCTCACA

>MH401860

TGATGCACGAAGTACATTAAGCCCGGTTCAGTGCTTTGAGGGTAGTGGTGGCAG

TGGTTCGTCAGGAAATTTTACATCTGGCAGTAATCTTAATATGGGCAGTGTCACTAACAC

AAGCAATACCGGCACGGGTACTTCCTCGGGTAGTGTTCCCATGGTAACTTTAACAGAATC

TCTGTTAAATAAACACAACGATGAAATGGAAAAATTTATGCTGAAAAAACATCGCGAATC

TCGTGGACGAAGCGGTGAAAAAAATAAAAAGAATACCGAAAAAATAATGGAATACAGTGG

TCCAGGGCATGGAATTAAACGGGTAGGATCCCA

>MH401861

TGAAGCACGCAGTACCCTGAGTCCGGTTCAGTGCTTTGAGGGTAGCGGTGGTAGT

GGATCCTCAGGAAATTTTACTTCGGGTAGCAACTTGAATATGGGCAGTGTTACTAATACA

AGCAATACCGGTACAGGCACTTCATCGGGAAGTGTACCCATGGTTACGTTGACGGAATCT

CTACTAAATAAACACAACGATGAAATGGAAAAGTTTATGCTAAAGAAACACAGAGAATCT

CGCGGACGAAGTGGTGAGAAGAATAAAAAGAACTCGGAAAAGATAATGGAGTATACTGGT

CCTGGGCATGGAATAAAGAGAGTTGGCTCACA

>MH401862

GGATGCACGCAGTACCCTAAGTCCGGTTCAGTGCTTTGAGGGCAGCGGTGG

AAGTGGTTCATCGGGAAATTTTACTTCGGGCAGTAACTTAAATATGGGCAGTGTGACTAA

TACCAGCAACACCGGTACTGGTACTTCCTCAGGAAGTGTTCCCATGGTCACGTTGACGGA

ATCTCTACTAAATAAACATAACGATGAAATGGAAAAGTTTATGCTTAAGAAGCATAGAGA

ATCTCGAGGACGAAGTGGCGAGAAGAACAAAAAGAATTCTGAAAAGATAATGGAGTACAC

TGGCCCGGGACACGGAATCAAAAGAGTTGGTTCACA

>MH401863

TGAAGCACGCAGTACCCTGAGTCCGGTTCAGTGCTTTGAGGGTAGCGGT

GGTAGTGGATCCTCGGGAAATTTTACTTCGGGTAGCAACTTGAATATGGGCAGTGTTACT

AATACAAGCAATACCGGTACAGGCACTTCATCGGGAAGTGTACCCATGGTTACGTTGACG

GAATCTCTACTAAATAAACACAACGATGAAATGGAAAAGTTTATGCTAAAGAAACACAGA

GAATCTCGCGGACGAAGTGGTGAGAAGAATAAAAAGAACTCGGAAAAGATAATGGAGTAT

ACTGGTCCTGGGCATGGAATAAAGAGAGTTGGCTCACA

>MH401864

GGATGCACGCAGTACCCTAAGTCCGGTTCAGTGCTTTGAGGGCAGCGGTGGAAG

TGGTTCATCGGGAAATTTTACTTCGGGCAGTAACTTAAATATGGGCAGTGTGACTAATAC

CAGCAACACCGGTACTGGTACTTCCTCAGGAAGTGTTCCCATGGTCACGTTGACGGAATC

TCTACTAAATAAACATAACGATGAAATGGAAAAGTTTATGCTTAAGAAGCATAGAGAATC

TCGAGGACGAAGTGGCGAGAAGAACAAAAAGAATTCTGAAAAGATAATGGAGTACACTGG

CCCGGGACACGGAATCAAAAGAGTTGGTTCACA

>MH401865

GGATGCACGCAGTACCCTAAGTCCGGTTCAGTGCTTTGAGGGCAGCGGTGGAAG

TGGTTCATCGGGAAATTTTACTTCGGGCAGTAACTTAAATATGGGCAGTGTGACTAATAC

CAGCAACACCGGTACTGGTACTTCCTCAGGAAGTGTTCCCATGGTCACGTTGACCGAATC

TCTACTAAATAAACATAACGATGAAATGGAAAAGTTTATGCTTAAGAAGCATAGAGAATC

TCGAGGACGAAGTGGCGAGAAGAACAAAAAGAATTCTGAAAAGATAATGGAATACACTGG

CCCGGGACACGGAATCAAAAGAGTTGGTTCACA

>MH401866

TGAAGC

ACGCAGTACCCTGAGTCCGGTTCAGTGCTTTGAGGGTAGCGGTGGTAGTGGATCCTCAGG

AAATTTTACTTCGGGTAGCAACTTGAATATGGGCAGTGTTACTAATACAAGCAATACCGG

TACAGGCACTTCATCGGGAAGTGTACCCATGGTTACGTTGACGGAATCTCTACTAAATAA

ACACAACGATGAAATGGAAAAGTTTATGCTAAAGAAACACAGAGAATCTCGCGGACGAAG

TGGTGAGAAGAATAAAAAGAACTCGGAAAAGATAATGGAGTATACTGGTCCTGGGCATGG

AATAAAGAGAGTTGGCTCACA

>MH401867

CGATGCACGCAGTACCCTAAGTCCGGTTCAGTGCTTTGAGGGCAGCGGTGGAAG

TGGTTCATCGGGAAATTTTACTTCGGGCAGTAACTTAAATATGGGCAGTGTGACTAATAC

CAGCAACACCGGTACTGGTACTTCCTCAGGAAGTGTTCCCATGGTCACGTTGACCGAATC

TCTACTAAATAAACATAACGATGAAATGGAAAAGTTTATGCTTAAGAAGCATAGAGAATC

TCGAGGACGAAGTGGCGAGAAGAACAAAAAGAATTCTGAAAAGATAATGGAGTACACTGG

CCCGGGACACGGAATCAAAAGAGTTGGTTCACA

>MH401868

GGATGCACGCAGTACCCTAAGTCCGGTTCAGTGCTTTGAGGGCAGCGGTGGAAG

TGGTTCATCGGGAAATTTTACTTCGGGCAGTAACTTAAATATGGGCAGTGTGACTAATAC

CAGCAACACCGGTACTGGTACTTCCTCAGGAAGTGTTCCCATGGTCACGTTGACGGAATC

TCTACTAAATAAACATAACGATGAAATGGAAAAGTTTATGCTTAAGAAGCATAGAGAATC

TCGAGGACGAAGTGGCGAGAAGAACAAAAAGAATTCTGAAAAGATAATGGAGTACACTGG

CCCGGGACACGGAATCAAAAGAGTTGGTTCACA

>MH401869

TGAAGCACGCAGTACCCTGAGTCCGGTTCAGTGCTTTGAGGGTAGCGGTGGTAGTGGATCCTC

AGGAAATTTTACTTCGGGTAGCAACTTGAATATGGGCAGTGTTACTAATACAAGCAATAC

CGGTACAGGCACTTCATCGGGAAGTGTACCCATGGTTACGTTGACGGAATCTCTACTAAA

TAAACACAACGATGAAATGGAAAAGTTTATGCTAAAGAAACACAGAGAATCTCGCGGACG

AAGTGGTGAGAAGAATAAAAAGAACTCGGAAAAGATAATGGAGTATACTGGTCCTGGGCA

TGGAATAAAGAGAGTTGGCTCACA

>MH401870

CGATGCACGCAGTACCCTAAGTCCGGTTCAGTGCTTTGAGGGCAGCGGTGGAAG

TGGTTCGTCGGGAAATTTTACTTCGGGCAGTAACTTAAATATGGGCAGTGTGACTAATAC

CAGCAACACCGGTACTGGTACTTCCTCTGGAAGTGTTCCCATGGTCACGTTGACCGAATC

TCTACTAAATAAACATAACGATGAAATGGAAAAGTTTATGCTTAAGAAGCATAGAGAATC

TCGAGGACGAAGTGGCGAGAAGAACAAAAAGAATTCTGAAAAGATAATGGAGTACACTGG

CCCGGGACACGGAATCAAAAGAGTTGGTTCACA

>MH401871

GGATGCACGCAGTACCCTAAGTCCGGTTCAGTGCTTTGAGGGCAGCGGTGGAAGTGGT

TCATCGGGAAATTTTACTTCGGGCAGTAACTTAAATATGGGCAGTGTGACTAATACCAGC

AACACCGGTACTGGTACTTCCTCAGGAAGTGTTCCCATGGTCACGTTGACCGAATCTCTA

CTAAATAAACATAACGATGAAATGGAAAAGTTTATGCTTAAGAAGCATAGAGAATCTCGA

GGACGAAGTGGCGAGAAGAACAAAAAGAATTCTGAAAAGATAATGGAATACACTGGCCCG

GGACACGGAATCAAAAGAGTTGGTTCACA

>MH401872

GGATGCACGCAGTACCCTAAGTCCGGTTCAGTGCTTTGAGGGCAGCGGTGGAAG

TGGTTCATCGGGAAATTTTACTTCGGGCAGTAACTTAAATATGGGCAGTGTGACTAATAC

CAGCAACACCGGTACTGGTACTTCCTCTGGAAGTGTTCCCATGGTCACGTTGACCGAATC

TCTACTAAATAAACATAACGATGAAATGGAAAAGTTTATGCTTAAGAAGCATAGAGAATC

TCGAGGACGAAGTGGCGAGAAGAACAAAAAGAATTCTGAAAAGATAATGGAGTACACTGG

CCCGGGACACGGAATCAAAAGAGTTGGTTCACA

>MH401873

TGAAGCACGCAGTACCCTGAGTCCGGTTCAGTGCTTTGAGGGTAGCGGTGGTAGTG

GATCCTCGGGAAATTTTACTTCGGGTAGCAACTTGAATATGGGCAGTGTTACTAATACAA

GCAATACCGGTACAGGCACTTCATCGGGAAGTGTACCCATGGTTACGTTGACGGAATCTC

TACTAAATAAACACAACGATGAAATGGAAAAGTTTATGCTAAAGAAACACAGAGAATCTC

GCGGACGAAGTGGTGAGAAGAATAAAAAGAACTCGGAAAAGATAATGGAGTATACTGGTC

CTGGGCATGGAATAAAGAGAGTTGGCTCACA

>MH401874

GGATGCACGCAGTACCCTAAGTCCGGTTCAGTGCTTTGAGGGCAGCGGTGGAAG

TGGTTCATCGGGAAATTTTACTTCGGGCAGTAACTTAAATATGGGCAGTGTGACTAATAC

CAGCAACACCGGTACTGGTACTTCCTCAGGAAGTGTTCCCATGGTCACGTTGACGGAATC

TCTACTAAATAAACATAACGATGAAATGGAAAAGTTTATGCTTAAGAAGCATAGAGAATC

TCGAGGACGAAGTGGCGAGAAGAACAAAAAGAATTCTGAAAAGATAATGGAGTACACTGG

CCCGGGACACGGAATCAAAAGAGTTGGTTCACA

>MH401875

CGATGCACGC

AGTACCCTAAGTCCGGTTCAGTGCTTTGAGGGCAGCGGTGGAAGTGGTTCATCGGGAAAT

TTTACTTCGGGCAGTAACTTAAATATGGGCAGTGTGACTAATACCAGCAACACCGGTACT

GGTACTTCCTCAGGAAGTGTTCCCATGGTCACGTTGACCGAATCTCTACTAAATAAACAT

AACGATGAAATGGAAAAGTTTATGCTTAAGAAGCATAGAGAATCTCGAGGACGAAGTGGC

GAGAAGAACAAAAAGAATTCTGAAAAGATAATGGAGTACACTGGCCCGGGACACGGAATC

AAAAGAGTTGGTTCACA

>MH401876

TGAAGCACGCAGTACCCTGAGTCCGGTTCAGTGCTTTGAGGGTAGCGGTGGTAG

TGGATCCTCAGGAAATTTTACTTCGGGTAGCAACTTGAATATGGGCAGTGTTACTAATAC

AAGCAATACCGGTACAGGCACTTCATCGGGAAGTGTACCCATGGTTACGTTGACGGAATC

TCTACTAAATAAACACAACGATGAAATGGAAAAGTTTATGCTAAAGAAACACAGAGAATC

TCGCGGACGAAGTGGTGAGAAGAATAAAAAGAACTCGGAAAAGATAATGGAGTATACTGG

TCCTGGGCATGGAATAAAGAGAGTTGGCTCACA

>MH401877

TGAAGCACGCAGTACCCTGAGTCCGGTTCAGTGCTTTGAGGGTAGCGGTGGTAGTGGAT

CCTCAGGAAATTTTACTTCGGGCAGCAACTTGAATATGGGCAGTGTTACTAATACAAGCA

ATACCGGCACAGGCACTTCATCGGGAAGTGTACCCATGGTTACGTTGACGGAATCTCTAC

TAAATAAACATAACGATGAAATGGAAAAGTTTATGCTAAAGAAACACAGAGAATCTCGCG

GACGAAGTGGTGAGAAGAATAAAAAGAACTCGGAAAAGATAATGGAGTATACTGGTCCTG

GACATGGAGTAAAGAGAGTTGGCTCACA

>MH401878

TGAAGCACGCAGTACCCTGAGTCCGGTTCAGTGCTTTGAGGGTAGCGGTGGTAGTG

GATCCTCAGGAAATTTTACTTCGGGTAGCAACTTGAATATGGGCAGTGTTACTAATACAA

GCAATACCGGTACAGGCACTTCATCGGGAAGTGTACCCATGGTTACGTTGACGGAATCTC

TACTAAATAAACACAACGATGAAATGGAAAAGTTTATGCTAAAGAAACACAGAGAATCTC

GTGGACGAAGTGGTGAGAAGAATAAAAAGAACTCGGAAAAGATAATGGAGTATACTGGTC

CTGGGCATGGAATAAAGAGAGTTGGCTCACA

>MH401879

TGAAGCACG

CAGTACCCTGAGTCCGGTTCAGTGCTTTGAGGGTAGCGGTGGTAGTGGATCCTCGGGAAA

TTTTACTTCGGGTAGCAACTTGAATATGGGCAGTGTTACTAATACAAGCAATACCGGTAC

AGGCACTTCATCGGGAAGTGTACCCATGGTTACGTTGACGGAATCTCTACTAAATAAACA

CAACGATGAAATGGAAAAGTTTATGCTAAAGAAACACAGAGAATCTCGCGGACGAAGTGG

TGAGAAGAATAAAAAGAACTCGGAAAAGATAATGGAGTATACTGGTCCTGGGCATGGAAT

AAAGAGAGTTGGCTCACA

>MH401880

CGATGCACGCAGTACTTTGAGTCCGGTTCAGTGCTTTGAAGGCAGCGGTGGTAG

TGGTTCATCGGGAAATTTTACTTCGGGCAGTAATTTGAATATGGGTAGTGTGACAAATAC

CAGCAACACCGGTACCGGCACTTCATCGGGAAGTGTTCCCATGGTTACGTTAACGGAATC

TCTACTAAATAAACATAACGATGAAATGGAAAAGTTTATGCTAAAGAAACATAGAGAATC

TCGTGGACGAAGTGGTGAGAAGAATAAAAAGAATTCTGAAAAGATAATGGAGTATACTGG

ACCGGGGCATGGAATCAAAAGAGTTGGATCTCA

>MH401881

AGCACGCAGTACCCTGAGTCCGGTTCAGTGCTTTGAGGGTAGCGGTGGTAGTGGATCCTC

AGGAAATTTTACTTCGGGCAGCAACTTGAATATGGGCAGTGTTACTAATACAAGCAATAC

CGGTACAGGCACTTCATCGGGAAGTGTACCCATGGTTACGTTGACGGAATCTCTACTAAA

TAAACACAACGATGAAATGGAAAAGTTTATGCTAAAGAAACACAGAGAATCTCGCGGACG

AAGTGGTGAGAAGAATAAAAAGAACTCGGAAAAGATAATGGAGTATACTGGTCCTGGGCA

TGGAATAAAGAGAGTTGGCTCACA

>MH401882

TGAAGCACGCAGTACCCTGAGTCCGGTTCAGTGCTTTGAGGGTAGCGGTGGTA

GTGGATCCTCAGGAAATTTTACTTCGGGCAGCAACTTGAATATGGGCAGTGTTACTAATA

CAAGCAATACCGGTACAGGCACTTCATCGGGAAGTGTACCCATGGTTACGTTGACGGAAT

CTCTACTAAATAAACACAACGATGAAATGGAAAAGTTTATGCTAAAGAAACACAGAGAAT

CTCGCGGACGCAGTGGTGAGAAGAATAAAAAGAACTCGGAAAAGATAATGGAGTATACTG

GTCCTGGGCATGGAATAAAGAGAGTTGGCTCACA

>MH588605

TGAAGCACGCAGTACCCTGAGTCCGGTTCAGTGCTTTGAGGGTAGCGGTGGTAGTGGATCCTCAGGAAATTTTACTTCGGGTAGCAACTTGAATATGGGCAGTGTTACTAATACAAGCAATACCGGTACAGGCACTTCATCGGGAAGTGTACCCATGGTTACGTTGACGGAATCTCTACTAAATAAACACAACGATGAAATGGAAAAGTTTATGCTAAAGAAACACAGAGAATCTCGCGGACGAAGTGGTGAGAAGAATAAAAAGAACTCGGAAAAGATAATGGAGTATACTGGTCCTGGGCATGGAATAAAGAGAGTTGGCTCACA

>MH588606

TGAAGCACGCAGTACCCTGAGTCCGGTTCAGTGCTTTGAGGGTAGCGGTGGTAGTGGATCCTCAGGAAATTTTACTTCGGGCAGCAACTTGAATATGGGCAGTGTTACTAATACAAGCAATACCGGTACAGGCACTTCATCGGGAAGTGTACCCATGGTTACGTTGACGGAATCTCTACTAAATAAACACAACGATGAAATGGAAAAGTTTATGCTAAAGAAACACAGAGAATCTCGCGGACGCAGTGGTGAGAAGAATAAAAAGAACTCGGAAAAGATAATGGAGTATACTGGTCCTGGGCATGGAATAAAGAGAGTTGGCTCACA

>MH588607

TGAAGCACGCAGTACCCTGAGTCCGGTTCAGTGCTTTGAGGGTAGCGGTGGTAGTGGATCCTCAGGAAATTTTACTTCGGGTAGCAACTTGAATATGGGCAGTGTTACTAATACAAGCAATACCGGTACAGGCACTTCATCGGGAAGTGTACCCATGGTTACGTTGACGGAATCTCTACTAAATAAACACAACGATGAAATGGAAAAGTTTATGCTAAAGAAACACAGAGAATCTCGCGGACGAAGTGGTGAGAAGAATAAAAAGAACTCGGAAAAGATAATGGAGTATACTGGTCCTGGGCATGGAATAAAGAGAGTTGGCTCACA

>MH588608

CGATGCACGCAGTACTTTGAGTCCGGTTCAGTGCTTTGAAGGCAGCGGTGGTAGTGGTTCATCGGGAAATTTTACTTCGGGCAGTAATTTGAATATGGGTAGTGTGACAAATACCAGCAACACCGGTACCGGCACTTCATCGGGAAGTGTTCCCATGGTTACGTTAACGGAATCTCTACTAAATAAACATAACGATGAAATGGAAAAGTTTATGCTAAAGAAACATAGAGAATCTCGTGGACGAAGTGGTGAGAAGAATAAAAAGAATTCTGAAAAGATAATGGAGTATACTGGACCGGGGCATGGAATCAAAAGAGTTGGATCTCA

>MH605070

CGATGCACGCAGTGCTTTGAGTCCGGTTCAGTGCTTTGAAGGCAGCGGTGGTAG

TGGTTCATCGGGAAATTTTACTTCGGGCAGTAACTTGAATATGGGCAGTGTGACTAATAC

CAGCAACACTGGTACCGGCACTTCCTCGGGAAGTGTTCCCATGGTTACATTAACGGAATC

TCTGCTAAATAAGCATAACGATGAAATGGAAAAATTTATGCTAAAAAAGCACAGAGAATC

TCGTGGACGAAGTGGCGAGAAGAATAAAAAGACTTCTGAAAAGATAATGGAATATACTGG

ACCGGGGCATGGAATCAAAAGAGTTGGATCACA

**COI+NAD5 sequences**

>3BGCym

AGAATTTTAATTCGAGCCGAACTAGGACACCCTGGAGCATTAAT
TGGTGATGACCAAATCTATAATGTAATTGTTACAGCTCATGCTTTTATTATAATTTTTTT
TATAGTAATACCAATTATAATTGGAGGGTTTGGTAATTGATTAGTCCCTTTAATGTTAGG
AGCCCCAGATATAGCTTTCCCTCGGATAAACAATATAAGTTTCTGACTTTTACCTCCTGC
ATTAACTTTACTATTAGTAAGTAGTATAGTGGAAAACGGAGCTGGAACAGGATGAACTGT
TTATCCACCTTTATCATCTAATATTGCTCATGGAGGAGCTTCTGTTGATTTAGCTATTTT
TTCTTTACATTTAGCGGGAATTTCTTCAATTTTAGGAGCTGTAAATTTTATTACAACAGT
TATTAATATACGATCAACAGGAATTACTTTTGACCGAATACCCCTATTCGTTTGATCAGT
AGTAATTACAGCTTTATTACTTTTACTATCTTTACCTGTTCTAGCTGGGGCTATTACTAT
ATTATTAACAGACCGAAACCTTAATACTTCATTCTTCGACCCAGCAGGAGGAGGAGACCC
AATTCTATACCAACACTTATTTAATAATTAATAATTCCGTATGTAGAAATATAAGGTATAAATCACATT

GACCCTAAAAAGTAAGAAGAGTTATAATTATTTAAAGCCTTATTGTAAAAAAATAAAGAA

ATGTTAGAAATTAAATAACCTCTAACTCCCCCTACAATACAAACAAATAAAGTTAATAAT

TTTAAATAATAAGGAAGAACCACTACTACAGGGGTAGGAAAAATCAATCAACTTAATATT

CTACCTCCAAAGATTCTCAAAATTAATAACCCTATTATACTTTTTAATATAACTCAACCT

TCATCATTTAATATATTTAAAGAAGAAAAATTAGAATCCCCA

>CvoUK
AGAATTTTAATTCGAGCTGAACTAGGGCAT
CCTGGAGCATTAATTGGAGATGATCAAATTTATAATGTAATTGTTACAGCTCATGCTTTT
ATTATAATTTTTTTTATAGTAATACCAATTATAATTGGAGGGTTTGGAAATTGATTAGTT
CCTTTAATATTAGGAGCCCCAGATATAGCATTCCCTCGAATAAATAATATAAGTTTCTGA
CTTTTACCTCCTGCATTAACTTTACTATTAGTAAGTAGTATAGTAGAAAACGGAGCTGGA
ACTGGATGAACTGTTTATCCACCTTTATCTTCTAATATTGCACATGGAGGAGCTTCTGTT
GATTTAGCTATTTTTTCTTTACATTTAGCAGGAATTTCTTCAATTTTAGGAGCTGTAAAT
TTTATTACTACAGTTATTAATATACGATCAACAGGTATTACCTTCGACCGAATACCATTA
TTTGTTTGATCAGTAGTAATTACAGCCTTATTACTTTTATTATCTTTACCAGTATTAGCA
GGAGCTATTACTATATTATTAACAGATCGAAATCTTAATACTTCATTCTTTGACCCAGCA
GGAGGAGGAGATCCAATTTTATACCAACACTTATTTAGTAATTAATAATTCCGTATGTAGAAATATAAGGCATAAACCACATTGAC

CCTAAAAAATAAGAAGAATTATAGTTATTTAAAGCCTTATTATAAAAAAATAAAGAAATA

TTAGAAATTAAATACCCTCTAATTCCTCCTACAATACAAACAAATAAAGTTAATAACTTT

AAATAAGAAGGTAGAACTACTACTACTGGTGTAGGAAAAATTAATCAACTTAACATTCTA

CCCCCAAAAATTCTTAAAATTAATAACCCTATTATACTCTTTAACATAACTCAACCTTCA

TCATTTAATATATTTAAAGAAGAAAAATTTGAATCCCCA

>ITMA3Lsi
AGAATTCTAATTCGAGCTGAATTAGGACATCCTGGAGCTTT
AATTGGAGATGATCAAATTTATAATGTAATTGTTACAGCTCATGCTTTTATTATAATTTT
TTTTATAGTAATGCCAATTATAATTGGAGGATTTGGAAATTGATTAGTTCCATTAATACT
AGGAGCTCCAGATATAGCATTCCCTCGAATAAATAATATAAGTTTTTGACTTTTACCTCC
TGCATTAACTTTATTATTAGTTAGTAGTATAGTAGAAAACGGAGCTGGAACAGGATGAAC
AGTTTACCCTCCTCTATCTTCTAATATTGCTCATGGAGGAGCTTCTGTTGATTTAGCTAT
TTTCTCTCTTCATTTAGCAGGAATTTCTTCAATTTTAGGAGCTGTAAATTTTATTACTAC
AGTTATTAATATACGATCAACAGGAATTACTTTTGATCGAATACCTTTATTTGTTTGATC
AGTAGTAATTACAGCTTTATTACTTTTATTATCATTACCAGTATTAGCAGGAGCTATTAC
AATACTTTTAACAGACCGAAATCTTAATACATCATTCTTTGACCCTGCAGGAGGAGGAGA
TCCAATTTTATACCAACATTTATTTGATAATTAATAATTCCATAAGTAGAAATATAAGGTATAAACCACATAGAACCTAAAAAATAAGAAAAATTATAATTATTTAAAGCCTTATTATAAAAAAATAAAGAAACATTAGAAATTATATAACCTATAATACCCCCAACAATACAAACAAATAATGTTAATAATTTTAAATAAAAAGGAAGAACAACTACTATAGGAGTAGGAAAAATCAATCATCTTAACATTCTCCCACCAAAAATTCTTAAAATTAATAAACCTATTATACTTTTCAATATTACTCAACCTTCATCATTTAATATATTTAAAGAAGAAAAATTAGAATCACCA
>ITMO2Li

AGAATTCTAATTCGAGCTGAATTAGGACACC
CTGGTGCACTAATTGGAGATGACCAAATTTATAATGTAATTGTTACAGCTCATGCTTTTA
TTATAATTTTCTTTATAGTAATACCAATTATAATTGGAGGATTTGGTAATTGATTAGTTC
CTTTAATGTTAGGAGCCCCAGATATAGCCTTCCCTCGATTAAATAATATAAGATTTTGAC
TTTTACCTCCTGCACTAACTTTATTATTAGTAAGTAGTATAGTAGAAAACGGAGCTGGAA
CAGGATGAACAGTTTACCCCCCTTTATCATCTAATATCGCTCATGGAGGAGCTTCTGTTG
ATTTAGCTATTTTTTCTCTTCATTTAGCAGGAATTTCTTCAATTTTAGGGGCTGTAAATT
TTATTACTACAGTCATTAATATACGATCAACAGGAATTACTTTTGACCGAATACCTTTAT
TTGTTTGATCAGTTGTAATTACAGCTCTATTACTTTTATTATCATTACCAGTATTAGCAG
GAGCTATTACAATACTTTTAACTGACCGAAACCTTAATACTTCATTTTTTGACCCTGCAG
GAGGAGGAGACCCAATTCTATACCAACATCTATTT AATAATTAATAATTCCGTAAGTAGAAATATAAGGTATAAATCATA

TTGATCCTAAAAAATAAGAAAAATTATAATTATTTAAAGCCTTATTATAAAAAAATAAAG

AAATATTTGAAATTATATAACCTCTTACTCCTCCTACAATACAAACAAATAATGTTAACA

ACTTTAAATATGAAGGTAATACTACTACTACTGGGGTAGGAAAAATTAATCATCTTAACA

TTCTCCCCCCAAAAATTCTTAAAATTAATAAACCTATTATACTTTTTAATATAACTCAAC

CTTCATCATTTAATATATTTAAAGAAGAAAAATTTGAATCACCA
>ITMO1Cvo

AGAATTTTAATTCGAGCTGAACTAGGGCATC
CTGGAGCATTAATTGGAGATGATCAAATTTATAATGTAATTGTTACAGCTCATGCTTTTA
TTATAATTTTTTTTATAGTAATACCAATTATAATTGGAGGGTTTGGAAATTGATTAGTTC
CTTTAATATTAGGAGCCCCAGATATAGCATTCCCTCGAATAAATAATATAAGTTTCTGAC
TTTTACCTCCTGCATTAACTTTACTATTAGTAAGTAGTATAGTAGAAAACGGAGCTGGAA
CTGGATGAACTGTTTATCCACCTTTATCTTCTAATATTGCACATGGAGGAGCTTCTGTTG
ATTTAGCTATTTTTTCTTTACATTTAGCAGGAATTTCTTCAATTTTAGGAGCTGTAAATT
TTATTACTACAGTTATTAATATACGATCAACAGGTATTACCTTCGACCGAATACCATTAT
TTGTTTGATCAGTAGTAATTACAGCCTTATTACTTTTATTATCTTTACCAGTATTAGCAG
GAGCTATTACTATATTATTAACAGATCGAAATCTTAATACTTCATTCTTTGACCCAGCAG
GAGGAGGAGATCCAATTTTATACCAACACTTATTT AGTAATTAATAATTCCGTATGTAGAAATATAAGGCATAAACCACA

TTGACCCTAAAAAATAAGAAGAATTATAGTTATTTAAAGCCTTATTATAAAAAAATAAAG

AAATATTAGAAATTAAATACCCTCTAATTCCTCCTACAATACAAACAAATAAAGTTAATA

ACTTTAAATAAGAAGGTAGAACTACTACTACTGGTGTAGGAAAAATTAATCAACTTAACA

TTCTACCCCCAAAAATTCTTAAAATTAATAACCCTATTATACTCTTTAACATAACTCAAC

CTTCATCATTTAATATATTTAAAGAAGAAAAATTTGAATCCCCA

>2BGCvi

AGAATTCTAATTCGAGCCGAACTAGGACA
TCCTGGAGCATTAATTGGAGATGACCAAATTTATAATGTAATTGTTACAGCTCATGCTTT
TATTATAATTTTTTTTATAGTAATACCAATTATAATTGGAGGATTTGGTAATTGATTAGT
CCCTTTAATATTAGGAGCTCCAGATATAGCCTTCCCTCGGATAAACAATATAAGTTTCTG
ACTTTTACCTCCTGCATTAACTTTACTATTAGTAAGTAGTATAGTAGAAAACGGAGCTGG
AACAGGATGAACTGTTTACCCACCTTTATCTTCTAATATCGCTCATGGAGGAGCTTCTGT
TGATTTAGCTATTTTTTCTTTACACTTAGCAGGAATTTCTTCAATTTTAGGAGCTGTAAA
TTTTATTACTACAGTTATTAATATACGATCAACAGGAATTACATTCGACCGAATACCATT
ATTTGTTTGATCTGTAGTAATTACAGCTTTATTACTTTTATTATCTTTACCAGTATTAGC
AGGTGCTATTACTATATTATTAACAGATCGAAATCTTAATACATCATTCTTTGATCCAGC
AGGAGGAGGAGACCCAATCTTGTACCAACATTTATTT AATAATTAATAATTCCATATGTAGAAATATAAGGTATAAATCATATT

GAACCTAAAAAGTAAGAAGAATTATAATTATTTAAAGCCTTATTATAAAAAAATAAAGAA

ATATTAGAAATTAAATAACCTCTAACTCCTCCTACAATACAAACAAATAAAGTTAATAAT

TTTAAATAAGAAGGAAGAACTACTACTACTGGTGTAGGAAAAATTAATCAACTTAATATA

CTACCCCCAAAAATTCTCAAAATTAATAGTCCTATTATACTTTTTAATATAACTCAACCT

TCATCATTTAGTATATTTAAAGAAGAAAAATTTGAATCCCCA

>ITMA8Cvi
AGAATTCTAATTCGAGCCGAACTAGGACATCCTGGAGC
ATTAATTGGAGATGATCAAATTTATAATGTAATTGTTACAGCTCATGCTTTTATTATAAT
TTTCTTTATAGTAATACCAATTATAATTGGAGGATTTGGTAATTGATTAGTCCCTTTAAT
ATTAGGAGCTCCAGATATAGCCTTCCCTCGGATAAACAATATAAGTTTCTGACTTTTACC
TCCTGCATTAACTTTACTATTAGTAAGTAGTATAGTAGAAAACGGAGCTGGAACAGGATG
AACTGTTTACCCACCTTTATCTTCTAATATCGCTCATGGAGGAGCTTCTGTTGATTTAGC
TATTTTTTCTTTACACTTAGCAGGAATTTCTTCAATTTTAGGAGCTGTAAATTTTATTAC
TACAGTTATTAATATACGATCAACAGGAATTACATTCGACCGAATACCATTATTTGTTTG
ATCTGTAGTAATTACAGCTTTATTACTTTTATTATCTTTACCAGTATTAGCAGGTGCTAT
TACTATATTATTAACAGATCGAAATCTTAATACATCATTCTTTGATCCAGCAGGAGGAGG
AGACCCAATCTTGTACCAACATTTATTT AATAATTAATAATTCCATATGTAGAAATATAAGGTATAAATCATATTGAACCT

AAAAAGTAAGAAGAATTATAATTATTTAAAGCCTTATTATAAAAAAATAAAGAAATATTA

GAAATTAAATAACCTCTAACTCCTCCTACAATACAAACAAATAAAGTTAATAATTTTAAA

TAAGAAGGAAGAACTACTACTACTGGCGTAGGAAAAATTAATCAACTTAATATACTACCC

CCAAAAATTCTCAAAATTAATAGTCCTATTATACTTTTTAATATAACTCAACCTTCATCA

TTTAGTATATTTAAAGAAGAAAAATTTGAATCCCCA

>ITQC2Phr
AGAATTCTAATTCGAGCTGAACTAGGACATCCCGGAGC
TCTAATTGGAGATGACCAAATTTATAATGTAATTGTAACAGCTCATGCTTTTATTATAAT
TTTCTTTATAGTAATGCCAATTATAATTGGAGGATTTGGAAATTGATTAGTTCCTTTAAT
ACTAGGGGCACCTGATATAGCTTTCCCACGAATAAACAATATAAGTTTCTGACTTTTACC
CCCCGCATTAACTCTATTGTTAGTAAGTAGTATAGTAGAAAATGGAGCTGGAACAGGATG
AACTGTTTACCCACCCTTATCATCTAATATTGCCCATGGAGGAGCATCTGTTGATCTGGC
TATTTTCTCTCTTCACTTAGCAGGAATTTCTTCAATTTTAGGGGCTGTAAATTTCATTAC
GACTGTAATTAATATACGATCAACTGGAATTACATTTGATCGAATACCTCTATTTGTTTG
ATCTGTAGTTATTACTGCTCTATTACTTTTATTATCTTTACCTGTATTAGCAGGTGCTAT
TACTATATTATTAACTGATCGAAATTTAAATACTTCATTCTTTGATCCAGCAGGAGGAGG
AGATCCTATTCTATACCAACACTTATTTTAATATTAATAATTCCATAAGTAGAAATATAAGGTATAAACCATATAGAGCCTAAAAAAT

AAGAAGAATGATAATTATTTAAAGCCTTATTATAAAAAAATAAAGAGACATGAGAAATTA

TGTAACCTATTAGTCCTCCTACAATACAAACAAATAAAGTTAATAATTTTAAATAAAAAG

GAAGAACTACTACTACTGGTGTAGGAAAAATTAATCATCTTAACATTCTCCCTCCAAAAA

TTCTTAAAATCAATAAACCTAATATACTTTTTAATATTACTCAACCTTCATCATTAAGTA

TATTTAAAGATGAAAAATTTGAATTCCCA

>ITNOai15Li
AGAATTCTAATTCGAGCTGAATTAGGACACCCTGGTGCACTA
ATTGGAGATGACCAAATTTATAATGTAATTGTTACAGCTCATGCTTTTATTATAATTTTC
TTTATAGTAATACCAATTATAATTGGAGGATTTGGTAATTGATTAGTTCCTTTAATGTTA
GGAGCCCCAGATATAGCCTTCCCTCGATTAAATAATATAAGATTTTGACTTTTACCTCCT
GCACTAACTTTATTATTAGTAAGTAGTATAGTAGAAAACGGAGCTGGAACAGGATGAACA
GTTTACCCCCCTTTATCATCTAATATCGCTCATGGAGGAGCTTCTGTTGATTTAGCTATT
TTTTCTCTTCATTTAGCAGGAATTTCTTCAATTTTAGGGGCTGTAAATTTTATTACTACA
GTCATTAATATACGATCAACAGGAATTACTTTTGACCGAATACCTTTATTTGTTTGATCA
GTTGTAATTACAGCTCTATTACTTTTATTATCATTACCAGTATTAGCAGGAGCTATTACA
ATACTTTTAACTGACCGAAACCTTAATACTTCATTTTTTGACCCTGCAGGAGGAGGAGAC
CCAATTCTATACCAACATCTATTTAAGATTTAATAATTCCGTAAGTAGAAATATAAGGTATAAATCATATTGACCCTAAAAAAT

AAGAAAAATTATAATTATTTAAAGCCTTATTATAAAAAAATAAAGAAATATTTGAAATTA

TATAACCTCTTACTCCTCCTACAATACAAACAAATAATGTTAACAACTTTAAATATGAAG

GTAATACTACTACTACTGGGGTAGGAAAAATTAATCATCTTAACATTCTCCCCCCAAAAA

TTCTTAAAATTAATAAACCTATTATACTTTTTAATATAACTCAACCTTCATCATTTAATA

TATTTAAAGAAGAAAAATTTGAATCACCA

>1BGLs
AGAATTCTAATTCGAGCTGAATTAGGACATCCTGG
AGCTTTAATTGGAGATGATCAAATTTATAATGTAATTGTTACAGCTCATGCTTTTATTAT
AATTTTTTTTATAGTAATGCCAATTATAATTGGAGGATTTGGAAATTGATTAGTTCCATT
AATACTAGGAGCTCCAGATATAGCATTCCCTCGAATAAATAATATAAGTTTTTGACTTTT
ACCTCCTGCATTAACTTTATTATTAGTTAGTAGTATAGTAGAAAACGGAGCTGGAACAGG
ATGAACAGTTTACCCTCCTCTATCTTCTAATATTGCTCATGGAGGAGCTTCTGTTGATTT
AGCTATTTTCTCTCTTCATTTAGCAGGAATTTCTTCAATTTTAGGAGCTGTAAATTTTAT
TACTACAGTTATTAATATACGATCAACAGGAATTACTTTTGATCGAATACCTTTATTTGT
TTGATCAGTAGTAATTACAGCTTTATTACTTTTATTATCATTACCAGTATTAGCAGGAGC
TATTACAATACTTTTAACAGACCGAAATCTTAATACATCATTCTTTGACCCTGCAGGAGG
AGGAGATCCAATTTTATACCAACATTTATTTAATAATTAATAATTCCATAAGTAGAAATATAAGGTATAAACCACATAGA

ACCTAAAAAATAAGAAAAATTATAATTATTTAAAGCCTTATTATAAAAAAATAAAGAAAC

ATTAGAAATTATATAACCTATAATACCCCCAACAATACAAACAAATAATGTTAATAATTT

TAAATAAGAAGGAAGAACAACTACTATAGGAGTAGGAAAAATCAATCATCTTAACATTCT

CCCACCAAAAATTCTTAAAATTAATAAACCTATTATACTTTTCAATATTACTCAACCTTC

ATCATTTAATATATTTAAAGAAGAAAAATTAGAATCACCA
>ITMO3Cvi
AGAATTCTAATTCGAGCCGAACTAGGACATCCTG
GAGCATTAATTGGAGATGACCAAATTTATAATGTAATTGTTACAGCTCATGCTTTTATTA
TAATTTTTTTTATAGTAATACCAATTATAATTGGAGGATTTGGTAATTGATTAGTTCCTT
TAATATTAGGAGCTCCAGATATAGCATTCCCTCGGATAAACAATATAAGTTTCTGACTTT
TACCTCCTGCATTAACTTTACTATTAGTAAGTAGTATAGTAGAAAACGGAGCTGGAACAG
GATGAACTGTTTACCCACCTTTATCTTCTAATATCGCTCATGGAGGAGCTTCTGTTGATT
TAGCTATTTTTTCTTTACACTTGGCAGGAATTTCTTCAATTTTAGGAGCTGTAAATTTTA
TTACTACAGTTATTAATATACGATCAACAGGAATTACATTCGACCGAATACCATTATTTG
TTTGATCTGTAGTAATTACAGCTTTATTACTTTTATTATCTTTACCAGTATTAGCAGGTG
CTATTACTATATTATTAACAGATCGAAATCTTAATACATCATTCTTTGACCCAGCAGGAG
GAGGAGACCCAATCTTGTACCAACATTTATTTAATAATTAATAATTCCATATGTAGAAATATAAGGTATAAATCATATTG

AACCTAAAAAGTAAGAAGAATTATAATTATTTAAAGCCTTATTATAAAAAAATAAAGAAA

TATTAGAAATTAAATAACCTCTAACTCCTCCTACAATACAAACAAATAAAGTTAATAATT

TTAAATAAGAAGGAAGAACTACTACTACTGGTGTAGGAAAAATTAATCAACTTAATATAC

TACCCCCAAAAATTCTCAAAATTAATAGTCCTATTATACTTTTTAATATAACTCAACCTT

CATCATTTAGTATATTTAAAGAAGAAAAATTTGAATCCCCA
>ITMO1Ls
AGAATTCTAATTCGAGCTGAATTAGGACATCCTGGAG
CTTTAATTGGAGATGATCAAATTTATAATGTAATTGTTACAGCTCATGCTTTTATTATAA
TTTTTTTTATAGTAATGCCAATTATAATTGGAGGATTTGGAAATTGATTAGTTCCATTAA
TACTAGGAGCTCCAGATATAGCATTCCCTCGAATAAATAATATAAGTTTTTGACTTTTAC
CTCCTGCATTAACTTTATTATTAGTTAGTAGTATAGTAGAAAACGGAGCTGGAACAGGAT
GAACAGTTTACCCTCCTCTATCTTCTAATATTGCTCATGGAGGAGCTTCTGTTGATTTAG
CTATTTTCTCTCTTCATTTAGCAGGAATTTCTTCAATTTTAGGAGCTGTAAATTTTATTA
CTACAGTTATTAATATACGATCAACAGGAATTACTTTTGATCGAATACCTTTATTTGTTT
GATCAGTAGTAATTACAGCTTTATTACTTTTATTATCATTACCAGTATTAGCAGGAGCTA
TTACAATACTTTTAACAGACCGAAATCTTAATACATCATTCTTTGACCCTGCAGGAGGAG
GAGATCCAATTTTATACCAACATTTATTTAATAATTAATAATTCCATAAGTAGAAATATAAGGTATAAACCACATAGAACC

TAAAAAATAAGAAAAATTATAATTATTTAAAGCCTTATTATAAAAAAATAAAGAAACATT

AGAAATTATATAACCTATAATACCCCCAACAATACAAACAAATAATGTTAATAATTTTAA

ATAAGAAGGAAGAACAACTACTATAGGAGTAGGAAAAATCAATCATCTTAACATTCTCCC

ACCAAAAATTCTTAAAATTAATAAACCTATTATACTTTTCAATATTACTCAACCTTCATC

ATTTAATATATTTAAAGAAGAAAAATTAGAATCACCA

>ITMA1Cvo

AGAATTTTAATTCGAGCTGAACTAGGGCATCCTGGAGC
ATTAATTGGAGATGATCAAATTTATAATGTAATTGTTACAGCTCATGCTTTTATTATAAT
TTTTTTTATAGTAATACCAATTATAATTGGAGGGTTTGGAAATTGATTAGTTCCTTTAAT
ATTAGGAGCCCCAGATATAGCATTCCCTCGAATAAATAATATAAGTTTCTGACTTTTACC
TCCTGCATTAACTTTACTATTAGTAAGTAGTATAGTAGAAAACGGAGCTGGAACTGGATG
AACTGTTTATCCACCTTTATCTTCTAATATTGCACATGGAGGAGCTTCTGTTGATTTAGC
TATTTTTTCTTTACATTTAGCAGGAATTTCTTCAATTTTAGGAGCTGTAAATTTTATTAC
TACAGTTATTAATATACGATCAACAGGTATTACCTTCGACCGAATACCATTATTTGTTTG
ATCAGTAGTAATTACAGCCTTATTACTTTTATTATCTTTACCAGTATTAGCAGGAGCTAT
TACTATATTATTAACAGATCGAAATCTTAATACTTCATTCTTTGACCCAGCAGGAGGAGG
AGATCCAATTTTATACCAACACTTATTTAGTAATTAATAATTCCGTATGTAGAAATATAAGGCATAAACCACATTGACC

CTAAAAAATAAGAAGAATTATAGTTATTTAAAGCCTTATTATAAAAAAATAAAGAAATAT

TAGAAATTAAATACCCTCTAATTCCTCCTACAATACAAACAAATAAAGTTAATAACTTTA

AATAAGAAGGTAGAACTACTACTACTGGTGTAGGAAAAATTAATCAACTTAACATTCTAC

CCCCAAAAATTCTTAAAATTAATAACCCTATTATACTCTTTAACATAACTCAACCTTCAT

CATTTAATATATTTAAAGAAGAAAAATTTGAATCCCCA

>10ITCvi
AGAATTCTAATTCGAGCCGAACTAGGACATCCTGGAGCA
TTAATTGGAGATGACCAAATTTATAATGTAATTGTTACAGCTCATGCTTTTATTATAATT
TTTTTTATAGTAATACCAATTATAATTGGAGGATTTGGTAATTGATTAGTCCCTTTAATA
TTAGGAGCTCCAGATATAGCCTTCCCTCGGATAAACAATATAAGTTTCTGACTTTTACCT
CCTGCATTAACTTTACTATTAGTAAGTAGTATAGTAGAAAACGGAGCTGGAACAGGATGA
ACTGTTTACCCACCTTTATCTTCTAATATCGCTCATGGAGGAGCTTCTGTTGATTTAGCT
ATTTTTTCTTTACACTTAGCAGGAATTTCTTCAATTTTAGGAGCTGTAAATTTTATTACT
ACAGTTATTAATATACGATCAACAGGAATTACATTCGACCGAATACCATTATTTGTTTGA
TCTGTAGTAATTACAGCTTTATTACTTTTATTATCTTTACCAGTATTAGCAGGTGCTATT
ACTATATTATTAACAGATCGAAATCTTAATACATCATTCTTTGACCCAGCAGGAGGAGGA
GACCCAATCTTGTACCAACATTTATTTAATAATTAATAATTCCATATGTAGAAATATAAGAGTATAAATCATATTG

AACCTAAAAAGTAAGAAGAATTATAATTATTTAAAGCCTTATTATAAAAAAATAAAGAAA

TATTAGAAATTAAATAACCTCTAACTCCTCCTACAATACAAACAAATAAAGTTAATAATT

TTAAATAAGAAGGAAGAACTACTACTACTGGTGTAGGAAAAATTAATCAACTTAATATAC

TACCCCCAAAAATTCTCAAAATTAATAGTCCTATTATACTTTTTAATATAACTCAACCTT

CATCATTTAGTATATTTAAAGAAGAAAAATTTGAATCCCCA

>1ITCvi
AGAATTCTAATTCGAGCCGAACTAGGACATCCTGGAGCATTA
ATTGGAGATGACCAAATTTATAATGTAATTGTTACAGCTCATGCTTTTATTATAATTTTT
TTTATAGTAATACCAATTATAATTGGAGGATTTGGTAATTGATTAGTCCCTTTAATATTA
GGAGCTCCAGATATAGCCTTCCCTCGGATAAACAATATAAGTTTCTGACTTTTACCTCCT
GCATTAACTTTACTATTAGTAAGTAGTATAGTAGAAAACGGAGCTGGAACAGGATGAACT
GTTTACCCACCTTTATCTTCTAATATCGCTCATGGAGGAGCTTCTGTTGATTTAGCTATT
TTTTCTTTACACTTAGCAGGAATTTCTTCAATTTTAGGAGCTGTAAATTTTATTACTACA
GTTATTAATATACGATCAACAGGAATTACATTCGACCGAATACCATTATTTGTTTGATCT
GTAGTAATTACAGCTTTATTACTTTTATTATCTTTACCAGTATTAGCAGGTGCTATTACT
ATATTATTAACAGATCGAAATCTTAATACATCATTCTTTGACCCAGCAGGAGGAGGAGAC
CCAATCTTGTACCAACATTTATTTAATAATTAATAATTCCATATGTAGAAATATAAGGTATAAATCATAT

TGAACCTAAAAAGTAAGAAGAATTATAATTATTTAAAGCCTTATTATAAAAAAATAAAGA

AATATTAGAAATTAAATAACCTCTAACTCCTCCTACAATACAAACAAATAAAGTTAATAA

TTTTAAATAAGAAGGAAGAACTACTACTACTGGTGTAGGAAAAATTAATCAACTTAATAT

ACTACCCCCAAAAATTCTCAAAATTAATAGTCCTATTATACTTTTTAATATAACTCAACC

TTCATCATTTAGTATATTTAAAGAAGAAAAATTTGAATCCCCA

>1ITLs
AGAATTCTAATTCGAGCTGAATTAGGACATC
CTGGAGCTTTAATTGGAGACGATCAAATTTATAATGTAATTGTTACAGCTCATGCTTTTA
TTATAATTTTTTTTATAGTAATGCCAATTATAATTGGAGGATTTGGAAATTGATTAGTTC
CATTAATACTAGGAGCTCCAGATATAGCATTCCCTCGAATAAATAATATAAGTTTTTGAC
TTTTACCTCCTGCATTAACTTTATTATTAGTTAGTAGTATAGTAGAAAACGGAGCTGGAA
CAGGATGAACAGTTTACCCTCCTCTATCTTCTAATATTGCTCATGGAGGAGCTTCTGTTG
ATTTAGCTATTTTCTCTCTTCATTTAGCAGGAATTTCTTCAATTTTAGGAGCTGTAAATT
TTATTACTACAGTTATTAATATACGATCAACAGGAATTACTTTTGATCGAATACCTTTAT
TTGTTTGATCAGTAGTAATTACAGCTTTATTACTTTTATTATCATTACCAGTATTAGCAG
GAGCTATTACAATACTTTTAACAGACCGAAATCTTAATACATCATTCTTTGACCCTGCAG
GAGGAGGAGATCCAATTTTATACCAACATTTATTTAATAATTAATAATTCCATAAGTAGAAATATAAGAGTATAAACCACATAGAAC

CTAAAAAATAAGAAAAATTATAATTATTTAAAGCCTTATTATAAAAAAATAAAGAAACAT

TAGAAATTATATAACCTATAATACCCCCAACAATACAAACAAATAATGTTAATAATTTTA

AATAAGAAGGAAGAACAACTACTATAGGAGTAGGAAAAATCAATCATCTTAACATTCTCC

CACCAAAAATTCTTAAAATTAATAAACCTATTATACTTTTCAATATTACTCAACCTTCAT

CATTTAATATATTTAAAGAAGAAAAATTAGAATCACCA

>2ITLs
AGAATTCTAATTCGAGCTGAATTAGGACATCCT
GGAGCTTTAATTGGAGATGATCAAATTTATAATGTAATTGTTACAGCTCATGCTTTTATT
ATAATTTTTTTTATAGTAATGCCAATTATAATTGGAGGATTTGGAAATTGATTAGTTCCA
TTAATACTAGGAGCTCCAGATATAGCATTCCCTCGAATAAATAATATAAGTTTTTGACTT
TTACCTCCTGCATTAACTTTATTATTAGTTAGTAGTATAGTAGAAAACGGAGCTGGAACA
GGATGAACAGTTTACCCTCCTCTATCTTCTAATATTGCTCATGGAGGAGCTTCTGTTGAT
TTAGCTATTTTCTCTCTTCATTTAGCAGGAATTTCTTCAATTTTAGGAGCTGTAAATTTT
ATTACTACAGTTATTAATATACGATCAACAGGAATTACTTTTGATCGAATACCTTTATTT
GTTTGATCAGTAGTAATTACAGCTTTATTACTTTTATTATCATTACCAGTATTAGCAGGA
GCTATTACAATACTTTTAACAGACCGAAATCTTAATACATCATTCTTTGACCCTGCAGGA
GGAGGAGATCCAATTTTATACCAACATTTATTTAATAATTAATAATTCCATAAGTAGAAATATAAGGTATAAACCAC

ATAGAACCTAAAAAATAAGAAAAATTATAATTATTTAAAGCCTTATTATAAAAAAATAAA

GAAACATTAGAAATTATATAACCTATAATACCCCCAACAATACAAACAAATAATGTTAAT

AATTTTAAATAAGAAGGAAGAACAACTACTATAGGAGTAGGAAAAATCAATCATCTTAAC

ATTCTCCCACCAAAAATTCTTAAAATTAATAAACCTATTATACTTTTCAATATTACTCAA

CCTTCATCATTTAATATATTTAAAGAAGAAAAATTAGAATCACCA
>3ITCvi
AGAATTCTAATTCGAGCCGAACTAGGACATCCTGGAGC
ATTAATTGGAGATGACCAAATTTATAATGTAATTGTTACAGCTCATGCTTTTATTATAAT
TTTTTTTATAGTAATACCAATTATAATTGGAGGATTTGGTAATTGATTAGTCCCTTTAAT
ATTAGGAGCTCCAGATATAGCCTTCCCTCGGATAAACAATATAAGTTTCTGACTTTTACC
TCCTGCATTAACTTTACTATTAGTAAGTAGTATAGTAGAAAACGGAGCTGGAACAGGATG
AACTGTTTACCCACCTTTATCTTCTAATATCGCTCATGGAGGAGCTTCTGTTGATTTAGC
TATTTTTTCTTTACACTTAGCAGGAATTTCTTCAATTTTAGGAGCTGTAAATTTTATTAC
TACAGTTATTAATATACGATCAACAGGAATTACATTCGACCGAATACCATTATTTGTTTG
ATCTGTAGTAATTACAGCTTTATTACTTTTATTATCTTTACCAGTATTAGCAGGTGCTAT
TACTATATTATTAACAGATCGAAATCTTAATACATCATTCTTTGACCCAGCAGGAGGAGG
AGACCCAATCTTGTACCAACATTTATTT AATAATTAATAATTCCATATGTAGAAATATAAGGTATAAATCATATTGAACCTAA

AAAGTAAGAAGAATTATAATTATTTAAAGCCTTATTATAAAAAAATAAAGAAATATTAGA

AATTAAATAACCTCTAACTCCTCCTACAATACAAACAAATAAAGTTAATAATTTTAAATA

AGAAGGAAGAACTACTACTACTGGTGTAGGAAAAATTAATCAACTTAATATACTACCCCC

AAAAATTCTCAAAATTAATAGTCCTATTATACTTTTTAATATAACTCAACCTTCATCATT

TAGTATATTTAAAGAAGAAAAATTTGAATCCCCA

>3ITLs
AGAATTCTAATTCGAGCTGAATTAGGACATCCTGG
AGCTTTAATTGGAGATGATCAAATTTATAATGTAATTGTTACAGCTCATGCTTTTATTAT
AATTTTTTTTATAGTAATGCCAATTATAATTGGAGGATTTGGAAATTGATTAGTTCCATT
AATACTAGGAGCTCCAGATATAGCATTCCCTCGAATAAATAATATAAGTTTTTGACTTTT
ACCTCCTGCATTAACTTTATTATTAGTTAGTAGTATAGTAGAAAACGGAGCTGGAACAGG
ATGAACAGTTTACCCTCCTCTATCTTCTAATATTGCTCATGGAGGAGCTTCTGTTGATTT
AGCTATTTTCTCTCTTCATTTAGCAGGAATTTCTTCAATTTTAGGAGCTGTAAATTTTAT
TACTACAGTTATTAATATACGATCAACAGGAATTACTTTTGATCGAATACCTTTATTTGT
TTGATCAGTAGTAATTACAGCTTTATTACTTTTATTATCATTACCAGTATTAGCAGGAGC
TATTACAATACTTTTAACAGACCGAAATCTTAATACATCATTCTTTGACCCTGCAGGAGG
AGGAGATCCAATTTTATACCAACATTTATTT AATAATTAATAATTCCATAAGTAGAAATATAAGGTATAAACCACATAGAAC

CTAAAAAATAAGAAAAATTATAATTATTTAAAGCCTTATTATAAAAAAATAAAGAAACAT

TAGAAATTATATAACCTATAATACCCCCAACAATACAAACAAATAATGTTAATAATTTTA

AATAAGAAGGAAGAACAACTACTATAGGAGTAGGAAAAATCAATCATCTTAACATTCTCC

CACCAAAAATTCTTAAAATTAATAAACCTATTATACTTTTCAATATTACTCAACCTTCAT

CATTTAATATATTTAAAGAAGAAAAATTAGAATCACCA

>4ITCvi
AGAATTCTAATTCGAGCCGAACTAGGAC
ATCCTGGAGCATTAATTGGAGATGACCAAATTTATAATGTAATTGTTACAGCTCATGCTT
TTATTATAATTTTTTTTATAGTAATACCAATTATAATTGGAGGATTTGGTAATTGATTAG
TCCCTTTAATATTAGGAGCTCCAGATATAGCCTTCCCTCGGATAAACAATATAAGTTTCT
GACTTTTACCTCCTGCATTAACTTTACTATTAGTAAGTAGTATAGTAGAAAACGGAGCTG
GAACAGGATGAACTGTTTACCCACCTTTATCTTCTAATATCGCTCATGGAGGAGCTTCTG
TTGATTTAGCTATTTTTTCTTTACACTTAGCAGGAATTTCTTCAATTTTAGGAGCTGTAA
ATTTTATTACTACAGTTATTAATATACGATCAACAGGAATTACATTCGACCGAATACCAT
TATTTGTTTGATCTGTAGTAATTACAGCTTTATTACTTTTATTATCTTTACCAGTATTAG
CAGGTGCTATTACTATATTATTAACAGATCGAAATCTTAATACATCATTCTTTGACCCAG
CAGGAGGAGGAGACCCAATCTTGTACCAACATTTATTTAATAATTAATAATTCCATATGTAGAAATATAAGGTATAAATCATATTGA

ACCTAAAAAGTAAGAAGAATTATAATTATTTAAAGCCTTATTATAAAAAAATAAAGAAAT

ATTAGAAATTAAATAACCTCTAACTCCTCCTACAATACAAACAAATAAAGTTAATAATTT

TAAATAAGAAGGAAGAACTACTACTACTGGTGTAGGAAAAATTAATCAACTTAATATACT

ACCCCCAAAAATTCTCAAAATTAATAGTCCTATTATACTTTTTAATATAACTCAACCTTC

ATCATTTAGTATATTTAAAGAAGAAAAATTTGAATCCCCA

>4ITLs
AGAATTCTAATTCGAGCTGAATTAGGACATCC
TGGAGCTTTAATTGGAGATGATCAAATTTATAATGTAATTGTTACAGCTCATGCTTTTAT
TATAATTTTTTTTATAGTAATGCCAATTATAATTGGAGGATTTGGAAATTGATTAGTTCC
ATTAATACTAGGAGCTCCAGATATAGCATTCCCTCGAATAAATAATATAAGTTTTTGACT
TTTACCTCCTGCATTAACTTTATTATTAGTTAGTAGTATAGTAGAAAACGGAGCTGGAAC
AGGATGAACAGTTTACCCTCCTCTATCTTCTAATATTGCTCATGGAGGAGCTTCTGTTGA
TTTAGCTATTTTCTCTCTTCATTTAGCAGGAATTTCTTCAATTTTAGGAGCTGTAAATTT
TATTACTACAGTTATTAATATACGATCAACAGGAATTACTTTTGATCGAATACCTTTATT
TGTTTGATCAGTAGTAATTACAGCTTTATTACTTTTATTATCATTACCAGTATTAGCAGG
AGCTATTACAATACTTTTAACAGACCGAAATCTTAATACATCATTCTTTGACCCTGCAGG
AGGAGGAGATCCAATTTTATACCAACATTTATTTAATAATTAATAATTCCATAAGTAGAAATATAAGAGTATAAACCA

CATAGAACCTAAAAAATAAGAAAAATTATAATTATTTAAAGCCTTATTATAAAAAAATAA

AGAAACATTAGAAATTATATAACCTATAATACCCCCAACAATACAAACAAATAATGTTAA

TAATTTTAAATAAGAAGGAAGAACAACTACTATAGGGGTAGGAAAAATCAATCATCTTAA

CATTCTCCCACCAAAAATTCTTAAAATTAATAAACCTATTATACTTTTCAATATTACTCA

ACCTTCATCATTTAATATATTTAAAGAAGAAAAATTAGAATCACCA

>5ITCvi
AGAATTCTAATTCGAGCCGAACTAGGACA
TCCTGGAGCATTAATTGGAGATGACCAAATTTATAATGTAATTGTTACAGCTCATGCTTT
TATTATAATTTTTTTTATAGTAATACCAATTATAATTGGAGGATTTGGTAATTGATTAGT
CCCTTTAATATTAGGAGCTCCAGATATAGCCTTCCCTCGGATAAACAATATAAGTTTCTG
ACTTTTACCTCCTGCATTAACTTTACTATTAGTAAGTAGTATAGTAGAAAACGGAGCTGG
AACAGGATGAACTGTTTACCCACCTTTATCTTCTAATATCGCTCATGGAGGAGCTTCTGT
TGATTTAGCTATTTTTTCTTTACACTTAGCAGGAATTTCTTCAATTTTAGGAGCTGTAAA
TTTTATTACTACAGTTATTAATATACGATCAACAGGAATTACATTCGACCGAATACCATT
ATTTGTTTGATCTGTAGTAATTACAGCTTTATTACTTTTATTATCTTTACCAGTATTAGC
AGGTGCTATTACTATATTATTAACAGATCGAAATCTTAATACATCATTCTTTGACCCAGC
AGGAGGAGGAGACCCAATCTTGTACCAACATTTATTTAATAATTAATAATTCCATATGTAGAAATATAAGGTATAAATCATATT

GAACCTAAAAAGTAAGAAGAATTATAATTATTTAAAGCCTTATTATAAAAAAATAAAGAA

ATATTAGAAATTAAATAACCTCTAACTCCTCCTACAATACAAACAAATAAAGTTAATAAT

TTTAAATAAGAAGGAAGAACTACTACTACTGGTGTAGGAAAAATTAATCAACTTAATATA

CTACCCCCAAAAATTCTCAAAATTAATAGTCCTATTATACTTTTTAATATAACTCAACCT

TCATCATTTAGTATATTTAAAGAAGAAAAATTTGAATCCCCA

>5ITLs
AGAATTCTAATTCGAGCTGAATTAGGACATCCTGGAGC
TTTAATTGGAGATGATCAAATTTATAATGTAATTGTTACAGCTCATGCTTTTATTATAAT
TTTTTTTATAGTAATGCCAATTATAATTGGAGGATTTGGAAATTGATTAGTTCCATTAAT
ACTAGGAGCTCCAGATATAGCATTCCCTCGAATAAATAATATAAGTTTTTGACTTTTACC
TCCTGCATTAACTTTATTATTAGTTAGTAGTATAGTAGAAAACGGAGCTGGAACAGGATG
AACAGTTTACCCTCCTCTATCTTCTAATATTGCTCATGGAGGAGCTTCTGTTGATTTAGC
TATTTTCTCTCTTCATTTAGCAGGAATTTCTTCAATTTTAGGAGCTGTAAATTTTATTAC
TACAGTTATTAATATACGATCAACAGGAATTACTTTTGATCGAATACCTTTATTTGTTTG
ATCAGTAGTAATTACAGCTTTATTACTTTTATTATCATTACCAGTATTAGCAGGAGCTAT
TACAATACTTTTAACAGACCGAAATCTTAATACATCATTCTTTGACCCTGCAGGAGGAGG
AGATCCAATTTTATACCAACATTTATTTAATAATTAATAATTCCATAAGTAGAAATATAAGGTATAAACCACATAGAACC

TAAAAAATAAGAAAAATTATAATTATTTAAAGCCTTATTATAAAAAAATAAAGAAACATT

AGAAATTATATAACCTATAATACCCCCAACAATACAAACAAATAATGTTAATAATTTTAA

ATAAGAAGGAAGAACAACTACTATAGGAGTAGGAAAAATCAATCATCTTAACATTCTCCC

ACCAAAAATTCTTAAAATTAATAAACCTATTATACTTTTCAATATTACTCAACCTTCATC

ATTTAATATATTTAAAGAAGAAAAATTAGAATCACCA

>6ITCvi
AGAATTCTAATTCGAGCCGAACTAGGACAT
CCTGGAGCATTAATTGGAGATGACCAAATTTATAATGTAATTGTTACAGCTCATGCTTTT
ATTATAATTTTTTTTATAGTAATACCAATTATAATTGGAGGATTTGGTAATTGATTAGTC
CCTTTAATATTAGGAGCTCCAGATATAGCCTTCCCTCGGATAAACAATATAAGTTTCTGA
CTTTTACCTCCTGCATTAACTTTACTATTAGTAAGTAGTATAGTAGAAAACGGAGCTGGA
ACAGGATGAACTGTTTACCCACCTTTATCTTCTAATATCGCTCATGGAGGAGCTTCTGTT
GATTTAGCTATTTTTTCTTTACACTTAGCAGGAATTTCTTCAATTTTAGGAGCTGTAAAT
TTTATTACTACAGTTATTAATATACGATCAACAGGAATTACATTCGACCGAATACCATTA
TTTGTTTGATCTGTAGTAATTACAGCTTTATTACTTTTATTATCTTTACCAGTATTAGCA
GGTGCTATTACTATATTATTAACAGATCGAAATCTTAATACATCATTCTTTGACCCAGCA
GGAGGAGGAGACCCAATCTTGTACCAACATTTATTTAATAATTAATAATTCCATATGTAGAAATATAAGGTATAAATCATATTGA

ACCTAAAAAGTAAGAAGAATTATAATTATTTAAAGCCTTATTATAAAAAAATAAAGAAAT

ATTAGAAATTAAATAACCTCTAACTCCTCCTACAATACAAACAAATAAAGTTAATAATTT

TAAATAAGAAGGAAGAACTACTACTACTGGTGTAGGAAAAATTAATCAACTTAATATACT

ACCCCCAAAAATTCTCAAAATTAATAGTCCTATTATACTTTTTAATATAACTCAACCTTC

ATCATTTAGTATATTTAAAGAAGAAAAATTTGAATCCCCA

>6ITLs
AGAATTCTAATTCGAGCTGAATTAGGACATCCTGGAGC
TTTAATTGGAGATGATCAAATTTATAATGTAATTGTTACAGCTCATGCTTTTATTATAAT
TTTTTTTATAGTAATGCCAATTATAATTGGAGGATTTGGAAATTGATTAGTTCCATTAAT
ACTAGGAGCTCCAGATATAGCATTCCCTCGAATAAATAATATAAGTTTTTGACTTTTACC
TCCTGCATTAACTTTATTATTAGTTAGTAGTATAGTAGAAAACGGAGCTGGAACAGGATG
AACAGTTTACCCTCCTCTATCTTCTAATATTGCTCATGGAGGAGCTTCTGTTGATTTAGC
TATTTTCTCTCTTCATTTAGCAGGAATTTCTTCAATTTTAGGAGCTGTAAATTTTATTAC
TACAGTTATTAATATACGATCAACAGGAATTACTTTTGATCGAATACCTTTATTTGTTTG
ATCAGTAGTAATTACAGCTTTATTACTTTTATTATCATTACCAGTATTAGCAGGAGCTAT
TACAATACTTTTAACAGACCGAAATCTTAATACATCATTCTTTGACCCTGCAGGAGGAGG
AGATCCAATTTTATACCAACATTTATTTAATAATTAATAATTCCATAAGTAGAAATATAAGGTATAAACCACATAGAA

CCTAAAAAATAAGAAAAATTATAATTATTTAAAGCCTTATTATAAAAAAATAAAGAAACA

TTAGAAATTATATAACCTATAATACCCCCAACAATACAAACAAATAATGTTAATAATTTT

AAATAAGAAGGAAGAACAACTACTATAGGAGTAGGAAAAATCAATCATCTTAACATTCTC

CCACCAAAAATTCTTAAAATTAATAAACCTATTATACTTTTCAATATTACTCAACCTTCA

TCATTTAATATATTTAAAGAAGAAAAATTAGAATCACCA

>7ITCvi
AGAATTCTAATTCGAGCCGAACTAGGACATCCTGGAGC
ATTAATTGGAGATGACCAAATTTATAATGTAATTGTTACAGCTCATGCTTTTATTATAAT
TTTTTTTATAGTAATACCAATTATAATTGGAGGATTTGGTAATTGATTAGTCCCTTTAAT
ATTAGGAGCTCCAGATATAGCCTTCCCTCGGATAAACAATATAAGTTTCTGACTTTTACC
TCCTGCATTAACTTTACTATTAGTAAGTAGTATAGTAGAAAACGGAGCTGGAACAGGATG
AACTGTTTACCCACCTTTATCTTCTAATATCGCTCATGGAGGAGCTTCTGTTGATTTAGC
TATTTTTTCTTTACACTTAGCAGGAATTTCTTCAATTTTAGGAGCTGTAAATTTTATTAC
TACAGTTATTAATATACGATCAACAGGAATTACATTCGACCGAATACCATTATTTGTTTG
ATCTGTAGTAATTACAGCTTTATTACTTTTATTATCTTTACCAGTATTAGCAGGTGCTAT
TACTATATTATTAACAGATCGAAATCTTAATACATCATTCTTTGACCCAGCAGGAGGAGG
AGACCCAATCTTGTACCAACATTTATTTAATAATTAATAATTCCATATGTAGAAATATAAGGTATAAATCATATT

GAACCTAAAAAGTAAGAAGAATTATAATTATTTAAAGCCTTATTATAAAAAAATAAAGAA

ATATTAGAAATTAAATAACCTCTAACTCCTCCTACAATACAAACAAATAAAGTTAATAAT

TTTAAATAAGAAGGAAGAACTACTACTACTGGTGTAGGAAAAATTAATCAACTTAATATA

CTACCCCCAAAAATTCTCAAAATTAATAGTCCTATTATACTTTTTAATATAACTCAACCT

TCATCATTTAGTATATTTAAAGAAGAAAAATTTGAATCCCCA

>7ITLs
AGAATTCTAATTCGAGCTGAATTAGGACATC
CTGGAGCTTTAATTGGAGATGATCAAATTTATAATGTAATTGTTACAGCTCATGCTTTTA
TTATAATTTTTTTTATAGTAATGCCAATTATAATTGGAGGATTTGGAAATTGATTAGTTC
CATTAATACTAGGAGCTCCAGATATAGCATTCCCTCGAATAAATAATATAAGTTTTTGAC
TTTTACCTCCTGCATTAACTTTATTATTAGTTAGTAGTATAGTAGAAAACGGAGCTGGAA
CAGGATGAACAGTTTACCCTCCTCTATCTTCTAATATTGCTCATGGAGGAGCTTCTGTTG
ATTTAGCTATTTTCTCTCTTCATTTAGCAGGAATTTCTTCAATTTTAGGAGCTGTAAATT
TTATTACTACAGTTATTAATATACGATCAACAGGAATTACTTTTGATCGAATACCTTTAT
TTGTTTGATCAGTAGTAATTACAGCTTTATTACTTTTATTATCATTACCAGTATTAGCAG
GAGCTATTACAATACTTTTAACAGACCGAAATCTTAATACATCATTCTTTGACCCTGCAG
GAGGAGGAGATCCAATTTTATACCAACATTTATTTAATAATTAATAATTCCATAAGTAGAAATATAAGGTATAAACCACATAG

AACCTAAAAAATAAGAAAAATTATAATTATTTAAAGCCTTATTATAAAAAAATAAAGAAA

CATTAGAAATTATATAACCTATAATACCCCCAACAATACAAACAAATAATGTTAATAATT

TTAAATAAGAAGGAAGAACAACTACTATAGGAGTAGGAAAAATCAATCATCTTAACATTC

TCCCACCAAAAATTCTTAAAATTAATAAACCTATTATACTTTTCAATATTACTCAACCTT

CATCATTTAATATATTTAAAGAAGAAAAATTAGAATCACCA

>8ITCvi
AGAATTCTAATTCGAGCCGAACTAGGACATCCTGGA
GCATTAATTGGAGATGACCAAATTTATAATGTAATTGTTACAGCTCATGCTTTTATTATA
ATTTTTTTTATAGTAATACCAATTATAATTGGAGGATTTGGTAATTGATTAGTCCCTTTA
ATATTAGGAGCTCCAGATATAGCCTTCCCTCGGATAAACAATATAAGTTTCTGACTTTTA
CCTCCTGCATTAACTTTACTATTAGTAAGTAGTATAGTAGAAAACGGAGCTGGAACAGGA
TGAACTGTTTACCCACCTTTATCTTCTAATATCGCTCATGGAGGAGCTTCTGTTGATTTA
GCTATTTTTTCTTTACACTTAGCAGGAATTTCTTCAATTTTAGGAGCTGTAAATTTTATT
ACTACAGTTATTAATATACGATCAACAGGAATTACATTCGACCGAATACCATTATTTGTT
TGATCTGTAGTAATTACAGCTTTATTACTTTTATTATCTTTACCAGTATTAGCAGGTGCT
ATTACTATATTATTAACAGATCGAAATCTTAATACATCATTCTTTGACCCAGCAGGAGGA
GGAGACCCAATCTTGTACCAACATTTATTTAATAATTAATAATTCCATATGTAGAAATATAAGGTATAAATCATATTGAA

CCTAAAAAGTAAGAAGAATTATAATTATTTAAAGCCTTATTATAAAAAAATAAAGAAATA

TTAGAAATTAAATAACCTCTAACTCCTCCTACAATACAAACAAATAAAGTTAATAATTTT

AAATAAGAAGGAAGAACTACTACTACTGGTGTAGGAAAAATTAATCAACTTAATATACTA

CCCCCAAAAATTCTCAAAATTAATAGTCCTATTATACTTTTTAATATAACTCAACCTTCA

TCATTTAGTATATTTAAAGAAGAAAAATTTGAATCCCCA

>8ITLs
AGAATTCTAATTCGAGCTGAATTAGGACATCCTGGAG
CTTTAATTGGAGACGATCAAATTTATAATGTAATTGTTACAGCTCATGCTTTTATTATAA
TTTTTTTTATAGTAATGCCAATTATAATTGGAGGATTTGGAAATTGATTAGTTCCATTAA
TACTAGGAGCTCCAGATATAGCATTCCCTCGAATAAATAATATAAGTTTTTGACTTTTAC
CTCCTGCATTAACTTTATTATTAGTTAGTAGTATAGTAGAAAACGGAGCTGGAACAGGAT
GAACAGTTTACCCTCCTCTATCTTCTAATATTGCTCATGGAGGAGCTTCTGTTGATTTAG
CTATTTTCTCTCTTCATTTAGCAGGAATTTCTTCAATTTTAGGAGCTGTAAATTTTATTA
CTACAGTTATTAATATACGATCAACAGGAATTACTTTTGATCGAATACCTTTATTTGTTT
GATCAGTAGTAATTACAGCTTTATTACTTTTATTATCATTACCAGTATTAGCAGGAGCTA
TTACAATACTTTTAACAGACCGAAATCTTAATACATCATTCTTTGACCCTGCAGGAGGAG
GAGATCCAATTTTATACCAACATTTATTTAATAATTAATAATTCCATAAGTAGAAATATAAGAGT

ATAAACCACATAGAACCTAAAAAATAAGAAAAATTATAATTATTTAAAGCCTTATTATAA

AAAAATAAAGAAACATTAGAAATTATATAACCTATAATACCCCCAACAATACAAACAAAT

AATGTTAATAATTTTAAATAAGAAGGAAGAACAACTACTATAGGAGTAGGAAAAATCAAT

CATCTTAACATTCTCCCACCAAAAATTCTTAAAATTAATAAACCTATTATACTTTTCAAT

ATTACTCAACCTTCATCATTTAATATATTTAAAGAAGAAAAATTAGAATCACCA

>9ITCvi
AGAATTCTAATTCGAGCCGAACTAGGACATCCTGGA
GCATTAATTGGAGATGACCAAATTTATAATGTAATTGTTACAGCTCATGCTTTTATTATA
ATTTTTTTTATAGTAATACCAATTATAATTGGAGGATTTGGTAATTGATTAGTCCCTTTA
ATATTAGGAGCTCCAGATATAGCCTTCCCTCGGATAAACAATATAAGTTTCTGACTTTTA
CCTCCTGCATTAACTTTACTATTAGTAAGTAGTATAGTAGAAAACGGAGCTGGAACAGGA
TGAACTGTTTACCCACCTTTATCTTCTAATATCGCTCATGGAGGAGCTTCTGTTGATTTA
GCTATTTTTTCTTTACACTTAGCAGGAATTTCTTCAATTTTAGGAGCTGTAAATTTTATT
ACTACAGTTATTAATATACGATCAACAGGAATTACATTCGACCGAATACCATTATTTGTT
TGATCTGTAGTAATTACAGCTTTATTACTTTTATTATCTTTACCAGTATTAGCAGGTGCT
ATTACTATATTATTAACAGATCGAAATCTTAATACATCATTCTTTGACCCAGCAGGAGGA
GGAGACCCAATCTTGTACCAACATTTATTTAATAATTAATAATTCCATATGTAGAAATATAAGGTATAAATCATA

TTGAACCTAAAAAGTAAGAAGAATTATAATTATTTAAAGCCTTATTATAAAAAAATAAAG

AAATATTAGAAATTAAATAACCTCTAACTCCTCCTACAATACAAACAAATAAAGTTAATA

ATTTTAAATAAGAAGGAAGAACTACTACTACTGGTGTAGGAAAAATTAATCAACTTAATA

TACTACCCCCAAAAATTCTCAAAATTAATAGTCCTATTATACTTTTTAATATAACTCAAC

CTTCATCATTTAGTATATTTAAAGAAGAAAAATTTGAATCCCCA

>9ITLs
AGAATTCTAATTCGAGCTGAATTAGGACATCCT
GGAGCTTTAATTGGAGATGATCAAATTTATAATGTAATTGTTACAGCTCATGCTTTTATT
ATAATTTTTTTTATAGTAATGCCAATTATAATTGGAGGATTTGGAAATTGATTAGTTCCA
TTAATACTAGGAGCTCCAGATATAGCATTCCCTCGAATAAATAATATAAGTTTTTGACTT
TTACCTCCTGCATTAACTTTATTATTAGTTAGTAGTATAGTAGAAAACGGAGCTGGAACA
GGATGAACAGTTTACCCTCCTCTATCTTCTAATATTGCTCATGGAGGAGCTTCTGTTGAT
TTAGCTATTTTCTCTCTTCATTTAGCAGGAATTTCTTCAATTTTAGGAGCTGTAAATTTT
ATTACTACAGTTATTAATATACGATCAACAGGAATTACTTTTGATCGAATACCTTTATTT
GTTTGATCAGTAGTAATTACAGCTTTATTACTTTTATTATCATTACCAGTATTAGCAGGA
GCTATTACAATACTTTTAACAGACCGAAATCTTAATACATCATTCTTTGACCCTGCAGGA
GGAGGAGATCCAATTTTATACCAACATTTATTTAATAATTAATAATTCCATAAGTAGAAATATAAGGTATAAACCAC

ATAGAACCTAAAAAATAAGAAAAATTATAATTATTTAAAGCCTTATTATAAAAAAATAAA

GAAACATTAGAAATTATATAACCTATAATACCCCCAACAATACAAACAAATAATGTTAAT

AATTTTAAATAAGAAGGAAGAACAACTACTATAGGAGTAGGAAAAATCAATCATCTTAAC

ATTCTCCCACCAAAAATTCTTAAAATTAATAAACCTATTATACTTTTCAATATTACTCAA

CCTTCATCATTTAATATATTTAAAGAAGAAAAATTAGAATCACCA

>BOX1UKSL
AGAATCCTAATTCGAGCAGAACTAGGTCACCC
TGGTGCATTAATTGGAGATGATCAAATTTATAATGTAATTGTTACAGCTCATGCTTTTAT
TATAATTTTTTTTATAGTAATACCAATCATAATTGGAGGATTTGGAAACTGACTAGTTCC
AATTATACTAGGAGCTCCAGATATAGCTTTCCCTCGAATAAATAATATAAGATTTTGACT
TTTACCTCCTGCATTAACATTACTACTAGTAAGTAGTATAGTAGAAAATGGAGCTGGAAC
AGGATGAACTGTTTACCCTCCTTTATCATCTAATATTGCTCATGGAGGAGCTTCTGTTGA
TCTAGCTATTTTTTCTCTTCACTTAGCTGGAATTTCTTCAATTTTAGGAGCAGTAAATTT
TATTACTACAGTAATTAATATACGATCTACAGGTATTACTTTTGATCGAATACCCCTTTT
TGTTTGATCAGTAGTAATTACCGCTTTACTTCTCCTTCTATCCCTACCCGTACTTGCAGG
AGCAATTACTATATTATTAACTGACCGAAATATTAATACTTCATTTTTTGATCCAGCAGG
AGGAGGAGATCCAATTCTATATCAACACTTATTTAATAATTTATAATCCCATAAGTAGAGATATAAGGTATAAATCAT

ATTGAACCTAAAAAATAAGAACTATTATAATTACTTAAAGCCTTATTAAAAAAAAATAAA

GAAACATTAGAAATTAAATAACCTATCAATCCTCCTATAATACAAACAAATAAAATTAAC

AACTTTAAATAAGAAGGTAATACAATTACTATAGGTCTAGGAAAAATTAATCATCTAAGT

ATTCTTCCTCCAAAAATTCTTAAAATTAATAAACCTATTATACTTTTTAATATAACTCAA

CCTTCATCATTTAACATATTTAAAGAAGAAAAATTTGAATCTCCA

>BOX3UKCvo
AGAATTTTAATTCGAGCTGAACTAGGGCATCC
TGGAGCATTAATTGGAGATGATCAAATTTATAATGTAATTGTTACAGCTCATGCTTTTAT
TATAATTTTTTTTATAGTAATACCAATTATAATTGGAGGGTTTGGAAATTGATTAGTTCC
TTTAATATTAGGAGCCCCAGATATAGCATTCCCTCGAATAAATAATATAAGTTTCTGACT
TTTACCTCCTGCATTAACTTTACTATTAGTAAGTAGTATAGTAGAAAACGGAGCTGGAAC
TGGATGAACTGTTTATCCACCTTTATCTTCTAATATTGCACATGGAGGAGCTTCTGTTGA
TTTAGCTATTTTTTCTTTACATTTAGCAGGAATTTCTTCAATTTTAGGAGCTGTAAATTT
TATTACTACAGTTATTAATATACGATCAACAGGTATTACCTTCGACCGAATACCATTATT
TGTTTGATCAGTAGTAATTACAGCCTTATTACTTTTATTATCTTTACCAGTATTAGCAGG
AGCTATTACTATATTATTAACAGATCGAAATCTTAATACTTCATTCTTTGACCCAGCAGG
AGGAGGAGATCCAATTTTATACCAACACTTATTTAGTAATTAATAATTCCGTATGTAGAAATATAAGGCATAAACCACA

TTGACCCTAAAAAATAAGAAGAATTATAGTTATTTAAAGCCTTATTATAAAAAAATAAAG

AAATATTAGAAATTAAATACCCTCTAATTCCTCCTACAATACAAACAAATAAAGTTAATA

ACTTTAAATAAGAAGGTAGAACTACTACTACTGGTGTAGGAAAAATTAATCAACTTAACA

TTCTACCCCCAAAAATTCTTAAAATTAATAACCCTATTATACTCTTTAACATAACTCAAC

CTTCATCATTTAATATATTTAAAGAAGAAAAATTTGAATCCCCA
>BOX4UKPrt
AGAATTCTAATTCGAGCCGAACTAGGACA
TCCTGGAGCATTAATTGGAGATGACCAAATTTATAATGTAATTGTTACAGCTCATGCTTT
TATTATAATTTTTTTTATAGTAATACCAATTATAATTGGAGGATTTGGTAATTGATTAGT
CCCTTTAATATTAGGAGCTCCAGATATAGCCTTCCCTCGGATAAACAATATAAGTTTCTG
ACTTTTACCTCCTGCATTAACTTTACTATTAGTAAGTAGTATAGTAGAAAACGGAGCTGG
AACAGGATGAACTGTTTACCCACCTTTATCTTCTAATATCGCTCATGGAGGAGCTTCTGT
TGATTTAGCTATTTTTTCTTTACACTTAGCAGGAATTTCTTCAATTTTAGGAGCTGTAAA
TTTTATTACTACAGTTATTAATATACGATCAACAGGAATTACATTCGACCGAATACCATT
ATTTGTTTGATCTGTAGTAATTACAGCTTTATTACTTTTATTATCTTTACCAGTATTAGC
AGGTGCTATTACTATATTATTAACAGATCGAAATCTTAATACATCATTCTTTGACCCAGC
AGGAGGAGGAGACCCAATCTTGTACCAACATTTATTTAATAATTAATAATTCCATATGTAGAAATATAAGGTATAAATCATATTGAA

CCTAAAAAGTAAGAAGAATTATAATTATTTAAAGCCTTATTATAAAAAAATAAAGAAATA

TTAGAAATTAAATAACCTCTAACTCCTCCTACAATACAAACAAATAAAGTTAATAATTTT

AAATAAGAAGGAAGAACTACTACTACTGGTGTAGGAAAAATTAATCAACTTAATATACTA

CCCCCAAAAATTCTCAAAATTAATAGTCCTATTATACTTTTTAATATAACTCAACCTTCA

TCATTTAGTATATTTAAAGAAGAAAAATTTGAATCCCCA
>BOX6UKH
AGAATTTTAATTCGAGCTGAATTAGGTCAT
CCTGGTGCATTAATTGGTAATGATCAAATTTATAATGTAATTGTAACAGCTCATGCTTTT
ATTATAATTTTTTTTATAGTTATACCTATTATAATTGGAGGATTTGGAAATTGATTAGTT
CCTTTAATATTAGGAGCTCCTGATATAGCTTTCCCTCGAATAAATAACATAAGCTTTTGA
CTTCTACCTCCTGCTTTAACTTTACTTTTAGTAAGCAGAATAGTTGAAAATGGAGCTGGT
ACAGGTTGAACTGTCTACCCCCCTTTATCATCTAATATTGCTCATGGAGGCGCTTCTGTT
GATTTAGCTATTTTTTCATTACATTTAGCAGGAATTTCTTCAATTTTAGGAGCCGTAAAT
TTTATTACTACTGTAATTAATATACGTTCAACTGGAATTACTTTTGATCGAATACCTTTA
TTTGTTTGATCCGTAGTAATTACAGCTCTTTTATTATTACTATCTCTACCAGTTTTAGCT
GGAGCAATTACTATACTATTAACAGATCGAAATTTAAATACTTCTTTTTTTGATCCTGCA
GGAGGAGGAGATCCTATTTTATATCAACATTTATTTGATAATTTATTATTCCATATGTTGAAATATAAGGTATAAATCATATT

GAACCTAAAAAATAAGACATATTATAATTACTAAAAGAATTATTATTAAAAAATAAAGAA

ACATTAGAAATATAATAACCAAATAATCCTCCAATAATACATACAATTAAAGTTAATAAT

TTTATTACTTTAGGTAAAATAATTACTACAGGAGTTGGAAAAATTAATCAACTTAATATT

CTTCCTCCAATAATTCTTATAATTAATAAACCTATTATACTTTTTAATATAATTCAACCT

TCATCATTTAATATATTTAAAGAAGAAAAATTAGAATTACCC

>ITVVChalbA

AGAATTCTAATTCGAGCTGAATTAGGACA
TCCTGGAGCACTAATTGGAGATGACCAAATTTATAATGTAATTGTAACAGCTCATGCCTT
TATTATAATTTTCTTTATAGTAATACCAATTATAATTGGAGGATTTGGAAATTGACTAGT
TCCTTTAATATTAGGAGCCCCAGATATAGCTTTCCCACGAATAAATAATATAAGTTTCTG
ACTTTTACCTCCTGCATTAACTTTACTATTAGTAAGTAGTATAGTAGAAAATGGAGCTGG
AACAGGATGAACTGTTTATCCACCTTTATCATCTAATATTGCTCATGGTGGAGCATCAGT
TGATTTAGCTATTTTTTCTTTACACTTAGCTGGAATTTCATCAATTTTAGGAGCTGTAAA
TTTTATTACAACTGTTATTAATATACGATCTACAGGAATCACATTTGATCGAATACCTTT
ATTCGTATGATCTGTAGTTATTACTGCTCTTCTTTTATTATTATCATTACCAGTATTAGC
CGGTGCAATTACTATATTATTAACTGATCGAAATTTAAATACTTCATTCTTTGATCCAGC
AGGAGGAGGAGATCCTATTTTATATCAACATTTATTT AATAATTAATAATACCATATGTAGAAATATATGGTATAAATCA

TATTGAACCTAAAAAATAAGAAGAATTATAATTATTTAATGCCTTATTATAAAAAAATAA

TGAAACATTAGAAATTATATAACCACTTACTCCTCCTACAATACAAACAAATATAGTTAA

CAACTTTAAATATATTGGAAGAACTACTAACACTGGAGTAGGAAAAATTAATCATCTTAA

TATTCTCCCTCCAAAAATTCTTAGAATCAATAAACCTATTATACTTTTTAATATTACTCA

ACCTTCATCATTTAATATATTTAAAGATGAAAAATTTGAATCTCCA

>ITVVChalbL

AGAATTCTAATTCGAGCTGAATTAGGACAT
CCTGGAGCACTAATTGGAGATGACCAAATTTATAATGTAATTGTAACAGCTCATGCCTTT
ATTATAATTTTCTTTATAGTAATACCAATTATAATTGGAGGATTTGGAAATTGACTAGTT
CCTTTAATATTAGGAGCCCCAGATATAGCTTTCCCACGAATAAATAATATAAGTTTCTGA
CTTTTACCTCCTGCATTAACTTTACTATTAGTAAGTAGTATAGTAGAAAATGGAGCTGGA
ACAGGATGAACTGTTTATCCACCTTTATCATCTAATATTGCTCATGGTGGAGCATCAGTT
GATTTAGCTATTTTTTCTTTACACTTAGCTGGAATTTCATCAATTTTAGGAGCTGTAAAT
TTTATTACAACTGTTATTAATATACGATCTACAGGAATCACATTTGATCGAATACCTTTA
TTCGTATGATCTGTAGTTATTACTGCTCTTCTTTTATTATTATCATTACCAGTATTAGCC
GGTGCAATTACTATATTATTAACTGATCGAAATTTAAATACTTCATTCTTTGATCCAGCA
GGAGGAGGAGATCCTATTTTATATCAACATTTATTTATAATTTAATAATACCATATGTAGAAATATATGGTATAAATCATATTGAACC

TAAAAAATAAGAAGAATTATAATTATTTAATGCCTTATTATAAAAAAATAATGAAACATT

AGAAATTATATAACCACTTACTCCTCCTACAATACAAACAAATATAGTTAACAACTTTAA

ATATATTGGAAGAACTACTAACACTGGAGTAGGAAAAATTAATCATCTTAATATTCTCCC

TCCAAAAATTCTTAGAATCAATAAACCTATTATACTTTTTAATATTACTCAACCTTCATC

ATTTAATATATTTAAAGATGAAAAATTTGAATCTCCA

>ITVVLs
AGAATTCTAATTCGAGCTGAATTAGGACATCCT
GGAGCTTTAATTGGAGATGATCAAATTTATAATGTAATTGTTACAGCTCATGCTTTTATT
ATAATTTTTTTTATAGTAATGCCAATTATAATTGGAGGATTTGGAAATTGATTAGTTCCA
TTAATACTAGGAGCTCCAGATATAGCATTCCCTCGAATAAATAATATAAGTTTTTGACTT
TTACCTCCTGCATTAACTTTATTATTAGTTAGTAGTATAGTAGAAAACGGAGCTGGAACA
GGATGAACAGTTTACCCTCCTCTATCTTCTAATATTGCTCATGGAGGAGCTTCTGTTGAT
TTAGCTATTTTCTCTCTTCATTTAGCAGGAATTTCTTCAATTTTAGGAGCTGTAAATTTT
ATTACTACAGTTATTAATATACGATCAACAGGAATTACTTTTGATCGAATACCTTTATTT
GTTTGATCAGTAGTAATTACAGCTTTATTACTTTTATTATCATTACCAGTATTAGCAGGA
GCTATTACAATACTTTTAACAGACCGAAATCTTAATACATCATTCTTTGACCCTGCAGGA
GGAGGAGATCCAATTTTATACCAACATTTATTTATAATTTAATAATTCCATAAGTAGAAATATAAGAGGTATAAACCACATAGAA

CCTAAAAAATAAGAAAAATTATAATTATTTAAAGCCTTATTATAAAAAAATAAAGAAACA

TTAGAAATTATATAACCTATAATACCCCCAACAATACAAACAAATAATGTTAATAATTTT

AAATAAGAAGGAAGAACAACTACTATAGGAGTAGGAAAAATCAATCATCTTAACATTCTC

CCACCAAAAATTCTTAAAATTAATAAACCTATTATACTTTTCAATATTACTCAACCTTCA

TCATTTAATATATTTAAAGAAGAAAAATTAGAATCACCA

>ITMACvi1
AGAATTCTAATTCGAGCCGAACTAGGACATCCTGGAGCA
TTAATTGGAGATGACCAAATTTATAATGTAATTGTTACAGCTCATGCTTTTATTATAATT
TTTTTTATAGTAATACCAATTATAATTGGAGGATTTGGTAATTGATTAGTCCCTTTAATA
TTAGGAGCTCCAGATATAGCCTTCCCTCGGATAAACAATATAAGTTTCTGACTTTTACCT
CCTGCATTAACTTTACTATTAGTAAGTAGTATAGTAGAAAACGGAGCTGGAACAGGATGA
ACTGTTTACCCACCTTTATCTTCTAATATCGCTCATGGAGGAGCTTCTGTTGATTTAGCT
ATTTTTTCTTTACACTTAGCAGGAATTTCTTCAATTTTAGGAGCTGTAAATTTTATTACT
ACAGTTATTAATATACGATCAACAGGAATTACATTCGACCGAATACCATTATTTGTTTGA
TCTGTAGTAATTACAGCTTTATTACTTTTATTATCTTTACCAGTATTAGCAGGTGCTATT
ACTATATTATTAACAGATCGAAATCTTAATACATCATTCTTTGACCCAGCAGGAGGAGGA
GACCCAATCTTGTACCAACATTTATTTAATAATTAATAATTCCATATGTAGAAATATAAGGTATAAATCATATTGAACCTA

AAAAGTAAGAAGAATTATAATTATTTAAAGCCTTATTATAAAAAAATAAAGAAATATTAG

AAATTAAATAACCTCTAACTCCTCCTACAATACAAACAAATAAAGTTAATAATTTTAAAT

AAGAAGGAAGAACTACTACTACTGGTGTAGGAAAAATTAATCAACTTAATATACTACCCC

CAAAAATTCTCAAAATTAATAGTCCTATTATACTTTTTAATATAACTCAACCTTCATCAT

TTAGTATATTTAAAGAAGAAAAATTTGAATCCCCA

>ITMACvi2
AGAATTCTAATTCGAGCCGAACTAGGACATCCTGGAGCA
TTAATTGGAGATGACCAAATTTATAATGTAATTGTTACAGCTCATGCTTTTATTATAATT
TTTTTTATAGTAATACCAATTATAATTGGAGGATTTGGTAATTGATTAGTCCCTTTAATA
TTAGGAGCTCCAGATATAGCCTTCCCTCGGATAAACAATATAAGTTTCTGACTTTTACCT
CCTGCATTAACTTTACTATTAGTAAGTAGTATAGTAGAAAACGGAGCTGGAACAGGATGA
ACTGTTTACCCACCTTTATCTTCTAATATCGCTCATGGAGGAGCTTCTGTTGATTTAGCT
ATTTTTTCTTTACACTTAGCAGGAATTTCTTCAATTTTAGGAGCTGTAAATTTTATTACT
ACAGTTATTAATATACGATCAACAGGAATTACATTCGACCGAATACCATTATTTGTTTGA
TCTGTAGTAATTACAGCTTTATTACTTTTATTATCTTTACCAGTATTAGCAGGTGCTATT
ACTATATTATTAACAGATCGAAATCTTAATACATCATTCTTTGACCCAGCAGGAGGAGGA
GACCCAATCTTGTACCAACATTTATTTGAATATTAATAATTCCATATGTAGAAATATAAGGTATAAATCATATTGAACCTAAAAAG

TAAGAAGAATTATAATTATTTAAAGCCTTATTATAAAAAAATAAAGAAATATTAGAAATT

AAATAACCTCTAACTCCTCCTACAATACAAACAAATAAAGTTAATAATTTTAAATAAGAA

GGAAGAACTACTACTACTGGTGTAGGAAAAATTAATCAACTTAATATACTACCCCCAAAA

ATTCTCAAAATTAATAGTCCTATTATACTTTTTAATATAACTCAACCTTCATCATTTAGT

ATATTTAAAGAAGAAAAATTTGAATCCCCA

>ITMOCvi3
AGAATTCTAATTCGAGCCGAACTAGGACATCCTGGA
GCATTAATTGGAGATGACCAAATTTATAATGTAATTGTTACAGCTCATGCTTTTATTATA
ATTTTTTTTATAGTAATACCAATTATAATTGGAGGATTTGGTAATTGATTAGTCCCTTTA
ATATTAGGAGCTCCAGATATAGCCTTCCCTCGGATAAACAATATAAGTTTCTGACTTTTA
CCTCCTGCATTAACTTTACTATTAGTAAGTAGTATAGTAGAAAACGGAGCTGGAACAGGA
TGAACTGTTTACCCACCTTTATCTTCTAATATCGCTCATGGAGGAGCTTCTGTTGATTTA
GCTATTTTTTCTTTACACTTAGCAGGAATTTCTTCAATTTTAGGAGCTGTAAATTTTATT
ACTACAGTTATTAATATACGATCAACAGGAATTACATTCGACCGAATACCATTATTTGTT
TGATCTGTAGTAATTACAGCTTTATTACTTTTATTATCTTTACCAGTATTAGCAGGTGCT
ATTACTATATTATTAACAGATCGAAATCTTAATACATCATTCTTTGACCCAGCAGGAGGA
GGAGACCCAATCTTGTACCAACATTTATTTAATAATTAATAATTCCATATGTAGAAATATAAGGTATAAATCATATTGA

ACCTAAAAAGTAAGAAGAATTATAATTATTTAAAGCCTTATTATAAAAAAATAAAGAAAT

ATTAGAAATTAAATAACCTCTAATTCCTCCTACAATACAAACAAATAAAGTTAATAATTT

TAAATAAAAAGGAAGAACTACTACTACTGGTGTAGGAAAAATTAATCAACTTAATATACT

ACCCCCAAAAATTCTCAAAATTAATAGTCCTATTATACTTTTTAATATAACTCAACCTTC

ATCATTTAGTATATTTAAAGAAGAAAAATTTGAATCTCCA

>ITMOCvi4
AGAATTCTAATTCGAGCCGAACTAGGACA
TCCTGGAGCATTAATTGGAGATGACCAAATTTATAATGTAATTGTTACAGCTCATGCTTT
TATTATAATTTTTTTTATAGTAATACCAATTATAATTGGAGGATTTGGTAATTGATTAGT
CCCTTTAATATTAGGAGCTCCAGATATAGCCTTCCCTCGGATAAACAATATAAGTTTCTG
ACTTTTACCTCCTGCATTAACTTTACTATTAGTAAGTAGTATAGTAGAAAACGGAGCTGG
AACAGGATGAACTGTTTACCCACCTTTATCTTCTAATATCGCTCATGGAGGAGCTTCTGT
TGATTTAGCTATTTTTTCTTTACACTTAGCAGGAATTTCTTCAATTTTAGGAGCTGTAAA
TTTTATTACTACAGTTATTAATATACGATCAACAGGAATTACATTCGACCGAATACCATT
ATTTGTTTGATCTGTAGTAATTACAGCTTTATTACTTTTATTATCTTTACCAGTATTAGC
AGGTGCTATTACTATATTATTAACAGATCGAAATCTTAATACATCATTCTTTGACCCAGC
AGGAGGAGGAGACCCAATCTTGTACCAACATTTATTTAATAATTAATAATTCCATATGTAGAAATATAAGGTATAAATCATATT

GAACCTAAAAAGTAAGAAGAATTATAATTATTTAAAGCCTTATTATAAAAAAATAAAGAA

ATATTAGAAATTAAATAACCTCTAACTCCTCCTACAATACAAACAAATAAAGTTAATAAT

TTTAAATAAGAAGGAAGAACTACTACTACTGGTGTAGGAAAAATTAATCAACTTAATATA

CTACCCCCAAAAATTCTCAAAATTAATAGTCCTATTATACTTTTTAATATAACTCAACCT

TCATCATTTAGTATATTTAAAGAAGAAAAATTTGAATCCCCA

>ITMOII1Cvi
AGAATTCTAATTCGAGCCGAACTAGGACATCCTGG
AGCATTAATTGGAGATGACCAAATTTATAATGTAATTGTTACAGCTCATGCTTTTATTAT
AATTTTTTTTATAGTAATACCAATTATAATTGGAGGATTTGGTAATTGATTAGTCCCTTT
AATATTAGGAGCTCCAGATATAGCCTTCCCTCGGATAAACAATATAAGTTTCTGACTTTT
ACCTCCTGCATTAACTTTACTATTAGTAAGTAGTATAGTAGAAAACGGAGCTGGAACAGG
ATGAACTGTTTACCCACCTTTATCTTCTAATATTGCTCATGGAGGAGCTTCTGTTGATTT
AGCTATTTTTTCTTTACACTTAGCAGGAATTTCTTCAATTTTAGGAGCTGTAAATTTTAT
TACTACAGTTATTAATATACGATCAACAGGAATTACATTCGACCGAATACCATTATTTGT
TTGATCTGTAGTAATTACAGCTTTATTACTTTTATTATCTTTACCAGTATTAGCAGGTGC
TATTACTATATTATTAACAGATCGAAATCTTAATACATCATTCTTTGACCCAGCAGGAGG
AGGAGACCCAATCTTGTACCAACATTTATTTAATAATTAATAATTCCATATGTAGAAATATAAGGTATAAATCAT
ATTGAACCTAAAAAGTAAGAAGAATTATAATTATTTAAAGCCTTATTATAAAAAAATAAA
GAAATATTAGAAATTAAATAACCTCTAACTCCTCCTACAATACAAACAAATAAAGTTAAT
AATTTTAAATAAGAAGGAAGAACTACTACTACTGGTGTAGGAAAAATTAATCAACTTAAT
ATACTACCCCCAAAAATTCTCAAAATTAATAGTCCTATTATACTTTTTAATATAACTCAA
CCTTCATCATTTAGTATATTTAAAGAAGAAAAATTTGAATCCCCA

>ITMOII10Ls
AGAATTCTAATTCGAGCTGAATTAGGACATCCTGGAG
CTTTAATTGGAGATGATCAAATTTATAATGTAATTGTTACAGCTCATGCTTTTATTATAA
TTTTTTTTATAGTAATGCCAATTATAATTGGAGGATTTGGAAATTGATTAGTTCCATTAA
TACTAGGAGCTCCAGATATAGCATTCCCTCGAATAAATAATATAAGTTTTTGACTTTTAC
CTCCTGCATTAACTTTATTATTAGTTAGTAGTATAGTAGAAAACGGAGCTGGAACAGGAT
GAACAGTTTACCCTCCTCTATCTTCTAATATTGCTCATGGAGGAGCTTCTGTTGATTTAG
CTATTTTCTCTCTTCATTTAGCAGGAATTTCTTCAATTTTAGGAGCTGTAAATTTTATTA
CTACAGTTATTAATATACGATCAACAGGAATTACTTTTGATCGAATACCTTTATTTGTTT
GATCAGTAGTAATTACAGCTTTATTACTTTTATTATCATTACCAGTATTAGCAGGAGCTA
TTACAATACTTTTAACAGACCGAAATCTTAATACATCATTCTTTGATCCTGCAGGAGGAG
GAGATCCAATTTTATACCAACATTTATTT GATAATTAATAATTCCATAAGTAGAAATATAAGGTATAAACCACATAGAACCTA
AAAAATAAGAAAAATTATAATTATTTAAAGCCTTATTATAAAAAAATAAAGAAACATGAG
AAATTATATAACCTATAATACCCCCAACAATACAAACAAATAATGTTAATAATTTTAAAT
AAGAAGGAAGAACAACTACTATAGGAGTAGGAAAAATTAATCATCTTAACATTCTCCCAC
CAAAAATTCTTAAAATTAATAAACCTATTATACTTTTCAATATTACTCAACCTTCATCAT
TTAATATATTTAAAGAAGAAAAATTAGAATCACCA
>ITMOII2Cvi
AGAATTCTAATTCGAGCCGAACTAGGACATCCTGG
AGCATTAATTGGAGATGACCAAATTTATAATGTAATTGTTACAGCTCATGCTTTTATTAT
AATTTTTTTTATAGTAATACCAATTATAATTGGAGGATTTGGTAATTGATTAGTCCCTTT
AATATTAGGAGCTCCAGATATAGCCTTCCCTCGGATAAACAATATAAGTTTCTGACTTTT
ACCTCCTGCATTAACTTTACTATTAGTAAGTAGTATAGTAGAAAACGGAGCTGGAACAGG
ATGAACTGTTTACCCACCTTTATCTTCTAATATTGCTCATGGAGGAGCTTCTGTTGATTT
AGCTATTTTTTCTTTACACTTAGCAGGAATTTCTTCAATTTTAGGAGCTGTAAATTTTAT
TACTACAGTTATTAATATACGATCAACAGGAATTACATTCGACCGAATACCATTATTTGT
TTGATCTGTAGTAATTACAGCTTTATTACTTTTATTATCTTTACCAGTATTAGCAGGTGC
TATTACTATATTATTAACAGATCGAAATCTTAATACATCATTCTTTGACCCAGCAGGAGG
AGGAGACCCAATCTTGTACCAACATTTATTT AATAATTAATAATTCCATATGTAGAAATATAAGGTATAAATCATAT
TGAACCTAAAAAGTAAGAAGAATTATAATTATTTAAAGCCTTATTATAAAAAAATAAAGA
AATATTAGAAATTAAATAACCTCTAACTCCTCCTACAATACAAACAAATAAAGTTAATAA
TTTTAAATAAGAAGGAAGAACTACTACTACTGGTGTAGGAAAAATTAATCAACTTAATAT
ACTACCCCCAAAAATTCTCAAAATTAATAGTCCTATTATACTTTTTAATATAACTCAACC
TTCATCATTTAGTATATTTAAAGAAGAAAAATTTGAATCCCCA

>ITMOII4Cvi
AGAATTCTAATTCGAGCCGAACTAGGACATCCTGGAG
CATTAATTGGAGATGACCAAATTTATAATGTAATTGTTACAGCTCATGCTTTTATTATAA
TTTTTTTTATAGTAATACCAATTATAATTGGAGGATTTGGTAATTGATTAGTCCCTTTAA
TATTAGGAGCTCCAGATATAGCCTTCCCTCGGATAAACAATATAAGTTTCTGACTTTTAC
CTCCTGCATTAACTTTACTATTAGTAAGTAGTATAGTAGAAAACGGAGCTGGAACAGGAT
GAACTGTTTACCCACCTTTATCTTCTAATATCGCTCATGGAGGAGCTTCTGTTGATTTAG
CTATTTTTTCTTTACACTTAGCAGGAATTTCTTCAATTTTAGGAGCTGTAAATTTTATTA
CTACAGTTATTAATATACGATCAACAGGAATTACATTCGACCGAATACCATTATTTGTTT
GATCTGTAGTAATTACAGCTTTATTACTTTTATTATCTTTACCAGTATTAGCAGGTGCTA
TTACTATATTATTAACAGATCGAAATCTTAATACATCATTCTTTGACCCAGCAGGAGGAG
GAGACCCAATCTTGTACCAACATTTATTT AATAATTAATAATTCCATATGTAGAAATATAAGGTATAAATCATATTGAAC
CTAAAAAGTAAGAAGAATTATAATTATTTAAAGCCTTATTATAAAAAAATAAAGAAATAT
TAGAAATTAAATAACCTCTAACTCCTCCTACAATACAAACAAATAAAGTTAATAATTTTA
AATAAGAAGGAAGAACTACTACTACTGGTGTAGGAAAAATTAATCAACTTAATATACTAC
CCCCAAAAATTCTCAAAATTAATAGTCCTATTATACTTTTTAATATAACTCAACCTTCAT
CATTTAGTATATTTAAAGAAGAAAAATTTGAATCCCCA
>ITMOII5Cvi
AGAATTCTAATTCGAGCCGAACTAGGACATC
CTGGAGCATTAATTGGAGATGACCAAATTTATAATGTAATTGTTACAGCTCATGCTTTTA
TTATAATTTTTTTTATAGTAATACCAATTATAATTGGAGGATTTGGTAATTGATTAGTCC
CTTTAATATTAGGAGCTCCAGATATAGCCTTCCCTCGGATAAACAATATAAGTTTCTGAC
TTTTACCTCCTGCATTAACTTTACTATTAGTAAGTAGTATAGTAGAAAACGGAGCTGGAA
CAGGATGAACTGTTTACCCACCTTTATCTTCTAATATTGCTCATGGAGGAGCTTCTGTTG
ATTTAGCTATTTTTTCTTTACACTTAGCAGGAATTTCTTCAATTTTAGGAGCTGTAAATT
TTATTACTACAGTTATTAATATACGATCAACAGGAATTACATTCGACCGAATACCATTAT
TTGTTTGATCTGTAGTAATTACAGCTTTATTACTTTTATTATCTTTACCAGTATTAGCAG
GTGCTATTACTATATTATTAACAGATCGAAATCTTAATACATCATTCTTTGACCCAGCAG
GAGGAGGAGACCCAATCTTGTACCAACATTTATTTAATAATTAATAATTCCATATGTAGAAATATAAGGTATAAATCATATTGAAC
CTAAAAAATAAGAAGAATTATAATTATTTAAAGCCTTATTATAAAAAAATAAAGAAATAT
TAGAAATTAAATAACCTCTAACTCCTCCTACAATACAAACAAATAAAGTTAATAATTTTA
AATAAGAAGGAAGAACTACTACTACTGGTGTAGGAAAAATTAATCAACTTAATATACTAC
CCCCAAAAATTCTCAAAATTAATAGTCCTATTATACTTTTTAATATAACTCAACCTTCAT
CATTTAGTATATTTAAAGAAGAAAAATTTGAATCCCCA

>ITMOII6Ls
AGAATTCTAATTCGAGCTGAATTAGGACATCCT
GGAGCTTTAATTGGAGATGATCAAATTTATAATGTAATTGTTACAGCTCATGCTTTTATT
ATAATTTTTTTTATAGTAATGCCAATTATAATTGGAGGATTTGGAAATTGATTAGTTCCA
TTAATACTAGGAGCTCCAGATATAGCATTCCCTCGAATAAATAATATAAGTTTTTGACTT
TTACCTCCTGCATTAACTTTATTATTAGTTAGTAGTATAGTAGAAAACGGAGCTGGAACA
GGATGAACAGTTTACCCTCCTCTATCTTCTAATATTGCTCATGGAGGAGCTTCTGTTGAT
TTAGCTATTTTCTCTCTTCATTTAGCAGGAATTTCTTCAATTTTAGGAGCTGTAAATTTT
ATTACTACAGTTATTAATATACGATCAACAGGAATTACTTTTGATCGAATACCTTTATTT
GTTTGATCAGTAGTAATTACAGCTTTATTACTTTTATTATCATTACCAGTATTAGCAGGA
GCTATTACAATACTTTTAACAGACCGAAATCTTAATACATCATTCTTTGACCCTGCAGGA
GGAGGAGATCCAATTTTATACCAACATTTATTTAATAATTAATAATTCCATAAGTAGAAATATAAGGTATAAACCAC
ATAGAACCTAAAAAATAAGAAAAATTATAATTATTTAAAGCCTTGTTATAAAAAAATAAA
GAAACATTAGAAATTATATAACCTATAATACCCCCAACAATACAAACAAATAATGTTAAT
AATTTTAAATAAGAAGGAAGAACAATTACTATAGGAGTAGGAAAAATCAATCATCTTAAC
ATTCTCCCACCAAAAATTCTTAAAATTAATAAACCTATTATACTTTTCAATATTACTCAA
CCTTCATCATTTAATATATTTAAAGAAGAAAAATTAGAATCACCA

>ITMOII7Ls
AGAATTCTAATTCGAGCTGAATTAGGA
CATCCTGGAGCTTTAATTGGAGATGATCAAATTTATAATGTAATTGTTACAGCTCATGCT
TTTATTATAATTTTTTTTATAGTAATGCCAATTATAATTGGAGGATTTGGAAATTGATTA
GTTCCATTAATACTAGGAGCTCCAGATATAGCATTCCCTCGAATAAATAATATAAGTTTT
TGACTTTTACCTCCTGCATTAACTTTATTATTAGTTAGTAGTATAGTAGAAAACGGAGCT
GGAACAGGATGAACAGTTTACCCTCCTCTATCTTCTAATATTGCTCATGGAGGAGCTTCT
GTTGATTTAGCTATTTTCTCTCTTCATTTAGCAGGAATTTCTTCAATTTTAGGAGCTGTA
AATTTTATTACTACAGTTATTAATATACGATCAACAGGAATTACTTTTGATCGAATACCT
TTATTTGTTTGATCAGTAGTAATTACAGCTTTATTACTTTTATTATCATTACCAGTATTA
GCAGGAGCTATTACAATACTTTTAACAGACCGAAATCTTAATACATCATTCTTTGACCCT
GCAGGAGGAGGAGATCCAATTTTTATACCAACATTTATTAATAATTAATAATTCCATAAGTAGAAATATAAGGTATAAACCACATAGAACCT
AAAAAATAAGAAAAATTATAATTATTTAAAGCCTTATTATAAAAAAATAAAGAAACATTA
GAAATTATATAACCTATAATACCCCCAACAATACAAACAAATAATGTTAATAATTTTAAA
TAAGAAGGAAGAACAACTACTATAGGAGTAGGAAAAATCAATCATCTTAACATTCTCCCA
CCAAAAATTCTTAAAATTAATAAACCTATTATACTTTTCAATATTACTCAACCTTCATCA
TTTAATATATTTAAAGAAGAAAAATTAGAATCACCA

>ITMOII8Ls
AGAATTCTAATTCGAGCTGAATTAGGACATCCT
GGAGCTTTAATTGGAGATGATCAAATTTACAATGTAATTGTTACAGCTCATGCTTTTATT
ATAATTTTTTTTATAGTAATGCCAATTATAATTGGAGGATTTGGAAATTGATTAGTTCCA
TTAATACTAGGAGCTCCAGATATAGCATTCCCTCGAATAAATAATATAAGTTTTTGACTT
TTACCTCCTGCATTAACTTTATTATTAGTTAGTAGTATAGTAGAAAACGGAGCTGGAACA
GGATGAACAGTTTACCCTCCTCTATCTTCTAATATTGCTCATGGAGGAGCTTCTGTTGAT
TTAGCTATTTTCTCTCTTCATTTAGCAGGAATTTCTTCAATTTTAGGAGCTGTAAATTTT
ATTACTACAGTTATTAATATACGATCAACAGGAATTACTTTTGATCGAATACCTTTATTT
GTTTGATCAGTAGTAATTACAGCTTTATTACTTTTATTATCATTACCAGTATTAGCGGGA
GCTATTACAATACTTTTAACAGACCGAAATCTTAATACATCATTCTTTGACCCTGCAGGA
GGAGGAGATCCAATTTTATACCAACATTTATTTAATAATTAATAATTCCATAAGTAGAAATATATGGTATAAACCACAT
AGAACCTAAAAAATAAGAAAAATTATAATTATTTAAAGCCTTATTATAAAAAAATAAAGA
AACATTAGAAATTATATAACCTATAATACCCCCAACAATACAAACAAATAATGTTAATAA
TTTTAAATAAGAAGGAAGAACAACTACTATAGGAGTAGGAAAAATCAATCATCTTAACAT
TCTCCCACCAAAAATTCTTAAAATTAATAAACCTATTATACTTTTCAATATTACTCAACC
TTCATCATTTAATATATTTAAAGAAGAAAAATTAGAATCACCA

>ITMOII9Ls
AGAATTCTAATTCGAGCTGAATTAGGACATCCT
GGAGCTTTAATTGGAGATGATCAAATTTATAATGTAATTGTTACAGCTCATGCTTTTATT
ATAATTTTTTTTATAGTAATGCCAATTATAATTGGAGGATTTGGAAATTGATTAGTTCCA
TTAATACTAGGAGCTCCAGATATAGCATTCCCTCGAATAAATAATATAAGTTTTTGACTT
TTACCTCCTGCATTAACTTTATTATTAGTTAGTAGTATAGTAGAAAACGGAGCTGGAACA
GGATGAACAGTTTACCCTCCTCTATCTTCTAATATTGCTCATGGAGGAGCTTCTGTTGAT
TTAGCTATTTTCTCTCTTCATTTAGCAGGAATTTCTTCAATTTTAGGAGCTGTAAATTTT
ATTACTACAGTTATTAATATACGATCAACAGGAATTACTTTTGATCGAATACCTTTATTT
GTTTGATCAGTAGTAATTACAGCTTTATTACTTTTATTATCATTACCAGTATTAGCAGGA
GCTATTACAATACTTTTAACAGACCGAAATCTTAATACATCATTCTTTGACCCTGCAGGA
GGAGGAGATCCAATTTTATACCAACATTTATTTAATAATTAATAATTCCATAAGTAGAAATATAAGGTATAAACCA
CATAGAACCTAAAAAATAAGAAAAATTATAATTATTTAAAGCCTTATTATAAAAAAATAA
AGAAACATTAGAAATTATATAACCTATAATACCCCCAACAATACAAACAAATAATGTTAA
TAATTTTAAATAAGAAGGAAGAACAACTACTATAGGAGTAGGAAAAATCAATCATCTTAA
CATTCTCCCACCAAAAATTCTTAAAATTAATAAACCTATTATACTTTTCAATATTACTCA
ACCTTCATCATTTAATATATTTAAAGAAGAAAAATTAGAATCACCA

>ITMOLi1
AGAATTCTAATTCGAGCTGAATTAGGACACC
CTGGTGCACTAATTGGAGATGACCAAATTTATAATGTAATTGTTACAGCTCATGCTTTTA
TTATAATTTTCTTTATAGTAATACCAATTATAATTGGAGGATTTGGTAATTGATTAGTTC
CTTTAATGTTAGGAGCCCCAGATATAGCCTTCCCTCGATTAAATAATATAAGATTTTGAC
TTTTACCTCCTGCACTAACTTTATTATTAGTAAGTAGTATAGTAGAAAACGGAGCTGGAA
CAGGATGAACAGTTTACCCCCCTTTATCATCTAATATCGCTCATGGAGGAGCTTCTGTTG
ATTTAGCTATTTTTTCTCTTCATTTAGCAGGAATTTCTTCAATTTTAGGGGCTGTAAATT
TTATTACTACAGTCATTAATATACGATCAACAGGAATTACTTTTGACCGAATACCTTTAT
TTGTTTGATCAGTTGTAATTACAGCTCTATTACTTTTATTATCATTACCAGTATTAGCAG
GAGCTATTACAATACTTTTAACTGACCGAAACCTTAATACTTCATTTTTTGACCCTGCAG
GAGGAGGAGACCCAATTCTATACCAACATCTATTTAATAATTAATAATTCCGTAAGTAGAAATATAAGGTATAAATCATATT

GACCCTAAAAAATAAGAAAAATTATAATTATTTAAAGCCTTATTATAAAAAAATAAAGAA

ATATTTGAAATTATATAACCTCTTACTCCTCCTACAATACAAACAAATAATGTTAACAAC

TTTAAATATGAAGGTAATACTACTACTACTGGGGTAGGAAAAATTAATCATCTTAACATT

CTCCCCCCAAAAATTCTTAAAATTAATAAACCTATTATACTTTTTAATATAACTCAACCT

TCATCATTTAATATATTTAAAGAAGAAAAATTTGAATCACCA

>UKPio
AGTATCTTAATCCGAGCAGAGCTA
GGACATCCAGGAGCTTTAATTGGTGATGATCAAATTTATAATGTAATTGTCACCGCCCAT
GCTTTTGTCATAATTTTTTTTATAGTAATACCAATTATAATTGGGGGATTTGGAAATTGA
TTAGTTCCTTTAATACTAGGAGCTCCTGATATAGCTTTCCCACGAATAAATAATATAAGT
TTTTGAATATTACCTCCTTCTCTTACACTACTATTAGTAAGAAGTATAGTAGAAAACGGA
GCTGGTACAGGTTGAACAGTTTACCCTCCTCTTTCTTCTGTTATTGCTCATGGAGGGGCT
TCTGTTGATTTAGCTATTTTTTCCCTTCACCTAGCAGGAATTTCTTCAATTTTAGGAGCT
GTAAATTTTATCACAACTGTAATTAATATACGATCTACAGGTATCACTTTTGATCGAATA
CCTTTATTTGTTTGATCTGTTGTAATTACAGCTTTATTATTACTTTTATCTCTACCTGTT
CTAGCAGGAGCAATTACAATATTATTAACAGATCGAAATTTAAATACTTCATTCTTTGAC
CCAGCAGGAGGAGGAGACCCAATTCTTTACCAACATTTATTT GATAACTCACAATTCCGTAAGTCGAAATAAAAGGTATAAA

TCATATTGAGCCAAAATAATATCTTAAAACATATGTTATTAAAGACTTATTAACATAAAA

TAAAGAAACGTTAGAAATTAAATAACCTAAAATACCCCCTACGATACAAACAAATAATGT

TAATAACTTTAAATAATAAGGTAAACACACTATTAAAGGAGTAGGAAAAATTAATCAATT

TAATATTCTACCTCCAATAATTCTTATAAATAATAATCCTAGTATACCCTTTAATATAAT

TCATCCCTCATCATTAAGTATATTTAAAGACCCGCAATTTAAATCCCCA

>ITTVCvi1
AGAATTCTAATTCGAGCCGAACTAGGGCATCCTGGAG
CATTAATTGGAGATGACCAAATTTATAATGTAATTGTTACAGCTCATGCTTTTATTATAA
TTTTTTTTATAGTAATACCAATTATAATTGGAGGATTTGGTAATTGATTAGTCCCTTTAA
TATTAGGAGCTCCAGACATAGCCTTCCCTCGGATAAACAATATAAGTTTCTGACTTTTAC
CTCCTGCATTAACTTTACTATTAGTAAGTAGTATAGTAGAAAACGGAGCTGGAACAGGAT
GAACTGTTTACCCACCTTTATCTTCTAATATCGCTCATGGAGGAGCTTCTGTTGATTTAG
CTATTTTTTCTTTACACTTGGCAGGAATTTCTTCAATTTTAGGAGCTGTAAATTTTATTA
CTACAGTTATTAATATACGATCAACAGGAATTACATTCGACCGAATACCATTATTTGTTT
GATCTGTAGTAATTACAGCTTTATTACTTTTATTATCTTTACCAGTATTAGCAGGTGCTA
TTACTATATTATTAACAGATCGAAATCTTAATACATCATTCTTTGACCCAGCAGGAGGAG
GAGACCCAATCTTGTACCAACATTTATTTAATAATTAATAATTCCATATGTAGAAATATAAGGTATAAATCATATT

GAACCTAAAAAGTAAGAAGAATTATAATTATTTAAAGCCTTATTATAAAAAAATAAAGAA

ATATTAGAAATTAAATAACCTCTAACTCCTCCTACAATACAAACAAATAAAGTTAATAAT

TTTAAATAAGAAGGAAGAACTACTACTACTGGTGTAGGAAAAATTAATCAACTTAATATA

CTACCCCCAAAAATTCTCAAAATTAATAGCCCTATTATACTTTTTAATATAACTCAACCT

TCATCATTTAGTATATTTAAAGAAGAAAAATTTGAATCCCCA

>ITTVCvi2
AGAATTCTAATTCGAGCCGAACTAGGACATCCTGGAG
CATTAATTGGAGATGACCAAATTTATAATGTAATTGTTACAGCTCATGCTTTTATTATAA
TTTTTTTTATAGTAATACCAATTATAATTGGAGGATTTGGTAATTGATTAGTCCCTTTAA
TATTAGGAGCTCCAGATATAGCCTTCCCTCGGATAAACAATATAAGTTTCTGACTTTTAC
CTCCTGCATTAACTTTACTATTAGTAAGTAGTATAGTAGAAAACGGAGCTGGAACAGGAT
GAACTGTTTACCCACCTTTATCTTCTAATATCGCTCATGGAGGAGCTTCTGTTGATTTAG
CTATTTTTTCTTTACACTTAGCAGGAATTTCTTCAATTTTAGGAGCTGTAAATTTTATTA
CTACAGTTATTAATATACGATCAACAGGAATTACATTCGACCGAATACCATTATTTGTTT
GATCTGTAGTAATTACAGCTTTATTACTTTTATTATCTTTACCAGTATTAGCAGGTGCTA
TTACTATATTATTAACAGATCGAAATCTTAATACATCATTCTTTGACCCAGCAGGAGGAG
GAGACCCAATCTTGTACCAACATTTATTTAATAATTAATAATTCCATATGTAGAAATATAAGGTATAAATCATATT

GAACCTAAAAAGTAAGAAGAATTATAATTATTTAAAGCCTTATTATAAAAAAATAAAGAA

ATATTAGAAATTAAATAACCTCTAACTCCTCCTACAATACAAACAAATAAAGTTAATAAT

TTTAAATAAGAAGGAAGAACTACTACTACTGGTGTAGGAAAAATTAATCAACTTAATATA

CTACCCCCAAAAATTCTCAAAATTAATAGTCCTATTATACTTTTTAATATAACTCAACCT

TCATCATTTAGTATATTTAAAGAAGAAAAATTTGAATCCCCA
>ITTVCvi3
AGAATTCTAATTCGAGCCGAACTAGGAC
ATCCTGGAGCATTAATTGGAGATGACCAAATTTATAATGTAATTGTTACAGCTCATGCTT
TTATTATAATTTTTTTTATAGTAATACCAATTATAATTGGAGGATTTGGTAATTGATTAG
TCCCTTTAATATTAGGAGCTCCAGATATAGCCTTCCCTCGGATAAACAATATAAGTTTCT
GACTTTTACCTCCTGCATTAACTTTACTATTAGTAAGTAGTATAGTAGAAAACGGAGCTG
GAACAGGATGAACTGTTTACCCACCTTTATCTTCTAATATCGCTCATGGAGGAGCTTCTG
TTGATTTAGCTATTTTTTCTTTACACTTAGCAGGAATTTCTTCAATTTTAGGGGCTGTAA
ATTTTATTACTACAGTTATTAATATACGATCAACAGGAATTACATTCGACCGAATACCAT
TATTTGTTTGATCTGTAGTAATTACAGCTTTATTACTTTTATTATCTTTACCAGTATTAG
CAGGTGCTATTACTATATTATTAACAGATCGAAATCTTAATACATCATTCTTTGACCCAG
CAGGAGGAGGAGACCCAATCTTGTACCAACATTTATTTAATAATTAATAATTCCATATGTAGAAATATAAGGTATAAATCATATTGAA

CCTAAAAAGTAAGAAGAATTATAATTATTTAAAGCCTTATTATAAAAAAATAAAGAAATA

TTAGAAATTAAATAACCTCTAACTCCTCCTACAATACAAACAAATAAAGTTAATAATTTT

AAATAAGAAGGAAGAACTACTACTACTGGTGTAGGAAAAATTAATCAACTTAATATACTA

CCCCCAAAAATTCTCAAAATTAATAGTCCTATTATACTTTTTAATATAACTCAACCTTCA

TCATTTAGTATATTTAAAGAAGAAAAATTTGAATCCCCA
>ITTVCvo1
AGAATTTTAATTCGAGCTGAACTAGGGCA
TCCTGGAGCATTAATTGGAGATGATCAAATTTATAATGTAATTGTTACAGCTCATGCTTT
TATTATAATTTTTTTTATAGTAATACCAATTATAATTGGAGGGTTTGGAAATTGATTAGT
TCCTTTAATATTAGGAGCCCCAGATATAGCATTCCCTCGAATAAATAATATAAGTTTCTG
ACTTTTACCTCCTGCATTAACTTTACTATTAGTAAGTAGTATAGTAGAAAACGGAGCTGG
AACTGGATGAACTGTTTATCCACCTTTATCTTCTAATATTGCACATGGAGGAGCTTCTGT
TGATTTAGCTATTTTTTCTTTACATTTAGCAGGAATTTCTTCAATTTTAGGAGCTGTAAA
TTTTATTACTACAGTTATTAATATACGATCAACAGGTATTACCTTCGACCGAATACCATT
ATTTGTTTGATCAGTAGTAATTACAGCCTTATTACTTTTATTATCTTTACCAGTATTAGC
AGGAGCTATTACTATATTATTAACAGATCGAAATCTTAATACTTCATTCTTTGACCCAGC
AGGAGGAGGAGATCCAATTTTATACCAACACTTATTTAGTAATTAATAATTCCGTATGTAGAAATATAAGAGCATAAACCACATTGA

CCCTAAAAAATAAGAAGAATTATAGTTATTTAAAGCCTTATTATAAAAAAATAAAGAAAT

ATTAGAAATTAAATACCCTCTAATTCCTCCTACAATACAAACAAATAAAGTTAATAACTT

TAAATAAGAAGGTAGAACTACTACTACTGGTGTAGGAAAAATTAATCAACTTAACATTCT

ACCCCCAAAAATTCTTAAAATTAATAACCCTATTATACTCTTTAACATAACTCAACCTTC

ATCATTTAATATATTTAAAGAAGAAAAATTTGAATCCCCA

>ITTVCvo2
AGAATTTTAATTCGAGCTGAACTAGGGCATCC
TGGAGCATTAATTGGAGATGATCAAATTTATAATGTAATTGTTACAGCTCATGCTTTTAT
TATAATTTTTTTTATAGTAATACCAATTATAATTGGAGGGTTTGGAAATTGATTAGTTCC
TTTAATATTAGGAGCCCCAGATATAGCATTCCCTCGAATAAATAATATAAGTTTCTGACT
TTTACCTCCTGCATTAACTTTACTATTAGTAAGTAGTATAGTAGAAAACGGAGCTGGAAC
TGGATGAACTGTTTATCCACCTTTATCTTCTAATATTGCACATGGAGGAGCTTCTGTTGA
TTTAGCTATTTTTTCTTTACATTTAGCAGGAATTTCTTCAATTTTAGGAGCTGTAAATTT
TATTACTACAGTTATTAATATACGATCAACAGGTATTACCTTCGACCGAATACCATTATT
TGTTTGATCAGTAGTAATTACAGCCTTATTACTTTTATTATCTTTACCAGTATTAGCAGG
AGCTATTACTATATTATTAACAGATCGAAATCTTAATACTTCATTCTTTGACCCAGCAGG
AGGAGGAGATCCAATTTTATACCAACACTTATTTAGTAATTAATAATTCCGTATGTAGAAATATAAGGCATAAACCACA

TTGACCCTAAAAAATAAGAAGAATTATAGTTATTTAAAGCCTTATTATAAAAAAATAAAG

AAATATTAGAAATTAAATACCCTCTAATTCCTCCTACAATACAAACAAATAAAGTTAATA

ACTTTAAATAAGAAGGTAGAACTACTACTACTGGTGTAGGAAAAATTAATCAACTTAACA

TTCTACCCCCAAAAATTCTTAAAATTAATAACCCTATTATACTCTTTAACATAACTCAAC

CTTCATCATTTAATATATTTAAAGAAGAAAAATTTGAATCCCCA

>ITTVCvo3
AGAATTTTAATTCGAGCTGAACTAGGGCAT
CCTGGAGCATTAATTGGAGATGATCAAATTTATAATGTAATTGTTACAGCTCATGCTTTT
ATTATAATTTTTTTTATAGTAATACCAATTATAATTGGAGGGTTTGGAAATTGATTAGTT
CCTTTAATATTAGGAGCCCCAGATATAGCATTCCCTCGAATAAATAATATAAGTTTCTGA
CTTTTACCTCCTGCATTAACTTTACTATTAGTAAGTAGTATAGTAGAAAACGGAGCTGGA
ACTGGATGAACTGTTTATCCACCTTTATCTTCTAATATTGCACATGGAGGAGCTTCTGTT
GATTTAGCTATTTTTTCTTTACATTTAGCAGGAATTTCTTCAATTTTAGGAGCTGTAAAT
TTTATTACTACAGTTATTAATATACGATCAACAGGTATTACCTTCGACCGAATACCATTA
TTTGTTTGATCAGTAGTAATTACAGCCTTATTACTTTTATTATCTTTACCAGTATTAGCA
GGAGCTATTACTATATTATTAACAGATCGAAATCTTAATACTTCATTCTTTGACCCAGCA
GGAGGAGGAGATCCAATTTTATACCAACACTTATTTAGTAATTAATAATTCCGTATGTAGAAATATAAGGCATAAACCACATT

GACCCTAAAAAATAAGAAGAATTATAGTTATTTAAAGCCTTATTATAAAAAAATAAAGAA

ATATTAGAAATTAAATACCCTCTAATTCCTCCTACAATACAAACAAATAAAGTTAATAAC

TTTAAATAAGAAGGTAGAACTACTACTACTGGTGTAGGAAAAATTAATCAACTTAACATT

CTACCCCCAAAAATTCTTAAAATTAATAACCCTATTATACTCTTTAACATAACTCAACCT

TCATCATTTAATATATTTAAAGAAGAAAAATTTGAATCCCCA
>ITTVLc1
AGAATTTTAATTCGAGCTGAATTAGGACACCCTGGTG
CATTAATTGGAGATGACCAAATTTATAATGTAATTGTTACAGCTCATGCTTTTATTATAA
TTTTCTTTATAGTAATACCAATTATAATTGGAGGATTTGGAAATTGATTAGTTCCTTTAA
TATTAGGAGCCCCAGATATGGCATTCCCTCGAATAAATAATATAAGATTTTGACTTTTAC
CTCCTGCATTAACTTTATTATTAGTAAGTAGTATAGTAGAAAACGGAGCTGGAACAGGAT
GAACAGTTTACCCTCCTTTATCATCTAATATCGCTCATGGAGGAGCTTCTGTTGATTTAG
CTATTTTTTCTCTTCATTTAGCAGGAATCTCATCAATTTTAGGGGCTGTAAATTTTATTA
CTACAGTTATTAATATACGATCAACAGGAATTACTTTTGACCGAATACCTTTATTCGTTT
GATCAGTAGTAATTACAGCTTTATTACTTTTATTATCATTACCAGTATTAGCTGGAGCTA
TTACTATACTTTTAACTGACCGAAATCTTAATACTTCATTCTTTGACCCAGCAGGAGGAG
GAGACCCAATTTTATACCAACATTTATTTAATAATTAATAATTCCGTAAGTAGAAATATAAGGTATAAACCACATT

GACCCTAAAAAATAAGAAAAATTATAATTATTTAAAGCCTTATTATAAAAAAATAAAGAA

ATATTTGAAATTATATACCCTCTTACTCCTCCTACAATACAAACAAATAATGTTAATAAC

TTCAAATATGAAGGAAGAACTACAACTACTGGAGTTGGAAAAATTAATCATCTTAATATT

CTTCCCCCAAAAATTCTTAAAATTAATAACCCTATTATACTTTTTAATATTACTCAACCT

TCATCATTTAATATATTTAAAGAAGAAAAATTTGAATCACCA
>ITTVLc2
AGAATTTTAATTCGAGCTGAATTAGGACACC
CTGGTGCATTAATTGGAGATGACCAAATTTATAATGTAATTGTTACAGCTCATGCTTTTA
TTATAATTTTCTTTATAGTAATACCAATTATAATTGGAGGATTTGGAAATTGATTAGTTC
CTTTAATATTAGGAGCCCCAGATATAGCATTCCCTCGAATAAATAATATAAGTTTTTGAC
TTTTACCTCCTGCATTAACTTTATTATTAGTAAGTAGTATAGTAGAAAACGGAGCTGGAA
CAGGATGAACAGTTTACCCTCCTTTATCATCTAATATCGCTCATGGAGGAGCTTCTGTTG
ATTTAGCTATTTTTTCTCTTCATTTAGCAGGAATTTCATCAATTTTAGGAGCTGTAAATT
TTATTACTACAGTTATTAATATACGATCAACAGGAATTACTTTTGACCGAATACCTTTAT
TCGTTTGATCAGTAGTAATTACAGCTTTATTACTTTTATTATCATTACCAGTATTAGCTG
GAGCTATTACTATACTTTTAACTGATCGAAATCTTAATACTTCATTCTTTGATCCAGCAG
GAGGAGGAGACCCAATTTTATACCAACATTTATTTAATAATTAATAATCCCGTAAGTAGAAATATAAGGTATAAACCATAT

TGACCCTAAAAAATAAGAAAAATTATAATTATTTAAAGCCTTATTATAAAAAAATAAAGA

AATATTTGAAATTATATACCCTCTTACTCCTCCTACAATACAAACAAATAATGTTAATAA

CTTCAAATATGAAGGAAGAACTACAACTACTGGAGTTGGAAAAATTAATCATCTTAATAT

TCTTCCTCCAAAAATTCTTAAAATTAATAACCCTATTATACTCTTTAATATTACTCAACC

TTCATCATTTAATATATTTAAAGAAGAAAAATTTGAATCACCA

>ITTVLc3
AGAATTTTAATTCGAGCTGAATTAGGACACCCTGGT
GCATTAATTGGAGATGACCAAATTTATAATGTAATTGTTACAGCTCATGCTTTTATTATA
ATTTTCTTTATAGTAATACCAATTATAATTGGAGGATTTGGAAATTGATTAGTTCCTTTA
ATATTAGGAGCCCCAGATATAGCATTCCCTCGAATAAATAATATAAGATTTTGACTTTTA
CCTCCTGCATTAACTTTATTGTTAGTAAGTAGTATAGTAGAAAACGGAGCTGGAACAGGA
TGAACAGTTTACCCTCCTTTATCATCTAATATCGCTCATGGAGGAGCTTCTGTTGATTTA
GCTATTTTTTCTCTTCATTTAGCAGGAATTTCATCAATTTTAGGAGCTGTAAATTTTATT
ACTACAGTTATTAATATACGATCAACAGGAATTACTTTTGACCGAATACCTTTATTCGTT
TGATCAGTAGTAATTACAGCTTTATTACTTTTATTATCATTACCAGTATTAGCTGGAGCT
ATTACTATACTTTTAACTGATCGAAATCTTAATACTTCATTCTTTGACCCAGCAGGAGGA
GGAGACCCAATTTTATACCAACATTTATTTAATAATTAATAATCCCGTAAGTAGAAATATAAGGGTATAAACCATATTGACC

CTAAAAAATAAGAAAAATTATAATTATTTAAAGCCTTATTATAAAAAAATAAAGAAATAT

TTGAAATTATATACCCTCTTACTCCTCCTACAATACAAACAAATAATGTTAATAACTTCA

AATATGAAGGAAGAACTACAACTACTGGAGTTGGAAAAATTAATCATCTTAATATTCTTC

CTCCAAAAATTCTTAAAATTAATAACCCTATTATACTCTTTAATATTACTCAACCTTCAT

CATTTAATATATTTAAAGAAGAAAAATTTGAATCACCA

>ITTVLill1
AGAATTTTAATTCGAGCTGAATTAGGACACCCTGGTGC
ATTAATTGGAGATGACCAAATTTATAATGTAATTGTTACAGCTCATGCTTTTATTATAAT
TTTCTTTATAGTAATGCCAATTATAATTGGAGGATTTGGAAATTGATTAGTTCCTTTAAT
ATTAGGAGCTCCAGATATAGCATTCCCTCGAATAAATAATATAAGATTTTGACTTTTACC
TCCTGCATTAACTTTACTATTAGTAAGTAGTATAGTAGAAAACGGAGCTGGAACAGGATG
AACAGTTTACCCTCCTTTATCATCTAATATCGCTCATGGAGGAGCTTCTGTTGATTTAGC
TATTTTTTCTCTTCATTTAGCAGGAATTTCATCAATTTTAGGAGCTGTAAATTTTATTAC
TACAGTTATTAATATACGATCAACAGGGATTACTTTTGATCGAATACCTTTATTCGTTTG
ATCAGTAGTAATTACAGCTTTATTACTTTTATTATCATTACCAGTATTAGCTGGAGCTAT
TACTATACTTTTAACTGATCGAAATCTTAATACTTCATTCTTTGATCCAGCAGGAGGAGG
AGACCCAATTTTATACCAACATTTATTTGATATTAATGAATGTCCATAAGTAGAAATATAAGGTATAAACCACATTGAACCTAAAA

AATAAGAAAAATTATAATTATTTAAAGCCTTATTATAAAAAAATAAGGAAATATTTGAAA

TTATATACCCTCTTACTCCTCCTACAATACAAACAAATAATGTTAATAACTTTAAATATG

AAGGAAGAACTACAACTACTGGAGTTGGAAAAATTAATCATCTTAATATTCTTCCTCCAA

AAATTCTTAAAATTAAGAATCCTATTATACTTTTTAATATTACTCAACCTTCATCATTTA

ATATATTTAAAGAAGAAAAATTTGAATCACCA

>ITTVLs1
AGAATTCTAATTCGAGCTGAATTAGGACAT
CCTGGAGCTTTAATTGGAGATGATCAAATTTATAATGTAATTGTTACAGCTCATGCTTTT
ATTATAATTTTTTTTATAGTAATGCCAATTATAATTGGAGGATTTGGAAATTGATTAGTT
CCATTAATACTAGGAGCTCCAGATATAGCATTCCCTCGAATAAATAATATAAGTTTTTGA
CTTTTACCTCCTGCATTAACTTTATTATTAGTTAGTAGTATAGTAGAAAACGGAGCTGGA
ACAGGATGAACAGTTTACCCTCCTCTATCTTCTAATATTGCTCATGGAGGAGCTTCTGTT
GATTTAGCTATTTTCTCTCTTCATTTAGCAGGAATTTCTTCAATTTTAGGAGCTGTAAAT
TTTATTACTACAGTTATTAATATACGATCAACAGGAATTACTTTTGATCGAATACCTTTA
TTTGTTTGATCAGTAGTAATTACAGCTTTATTACTTTTATTATCATTACCAGTATTAGCA
GGAGCTATTACAATACTTTTAACAGACCGAAATCTTAATACATCATTCTTTGACCCTGCA
GGAGGAGGAGATCCAATTTTATACCAACATTTATTTAATAATTAATAATTCCATAAGTAGAAATATAAGAGTATAAACCACATAGA

ACCTAAAAAATAAGAAAAATTATAATTATTTAAAGCCTTATTATAAAAAAATAAAGAAAC

ATTAGAAATTATATAACCTATAATACCCCCAACAATACAAACAAATAATGTTAATAATTT

TAAATAAGAAGGAAGAACAACTACTATAGGAGTAGGAAAAATCAATCATCTTAACATTCT

CCCACCAAAAATTCTTAAAATTAATAAACCTATTATACTTTTCAATATTACTCAACCTTC

ATCATTTAATATATTTAAAGAAGAAAAATTAGAATCACCA
>ITTVLs2
AGAATTCTAATTCGAGCTGAATTAGGACATC
CTGGAGCTTTAATTGGAGATGATCAAATTTATAATGTAATTGTTACAGCTCATGCTTTTA
TTATAATTTTTTTTATGGTAATGCCAATTATAATTGGAGGATTTGGAAATTGATTAGTTC
CATTAATACTAGGAGCTCCAGATATAGCATTCCCTCGAATAAATAATATAAGTTTTTGAC
TTTTACCTCCTGCATTAACTTTATTATTAGTTAGTAGTATAGTAGAAAACGGAGCTGGAA
CAGGATGAACAGTTTACCCTCCTCTATCTTCTAATATTGCTCATGGAGGAGCTTCTGTTG
ATTTAGCTATTTTCTCTCTTCATTTAGCAGGAATTTCTTCAATTTTAGGAGCTGTAAATT
TTATTACTACAGTTATTAATATACGATCAACAGGAATTACTTTTGATCGAATACCTTTAT
TTGTTTGATCAGTAGTAATTACAGCTTTATTACTTTTATTATCATTACCAGTATTAGCAG
GAGCTATTACAATACTTTTAACAGACCGAAATCTTAATACATCATTCTTTGACCCTGCAG
GAGGAGGAGATCCAATTTTATACCAACATTTATTTAATAATTAATAATTCCATAAGTAGAAATATAAGAGTATAAACCACATAGAACC

TAAAAAATAAGAAAAATTATAATTATTTAAAGCCTTATTATAAAAAAATAAAGAAACATT

AGAAATTATATAACCTATAATACCCCCAACAATACAAACAAATAATGTTAATAATTTTAA

ATAAGAAGGAAGAACAACTACTATAGGAGTAGGAAAAATCAATCATCTTAACATTCTCCC

ACCAAAAATTCTTAAAATTAATAAACCTATTATACTTTTCAATATTACTCAACCTTCATC

ATTTAATATATTTAAAGAAGAAAAATTAGAATCACCA

>ITTVLs3
AGAATTCTAATTCGAGCTGAATTAGGACATC
CTGGAGCTTTAATTGGAGATGATCAAATTTATAATGTAATTGTTACAGCTCATGCTTTTA
TTATAATTTTTTTTATAGTAATGCCAATTATAATTGGAGGATTTGGAAATTGATTAGTTC
CATTAATACTAGGAGCTCCAGATATAGCATTCCCTCGAATAAATAATATAAGTTTTTGAC
TTTTACCTCCTGCATTAACTTTATTATTAGTTAGTAGTATAGTAGAAAACGGAGCTGGAA
CAGGATGAACAGTTTACCCTCCTCTATCTTCTAATATTGCTCATGGAGGAGCTTCTGTTG
ATTTAGCTATTTTCTCTCTTCATTTAGCAGGAATTTCTTCAATTTTAGGAGCTGTAAATT
TTATTACTACAGTTATTAATATACGATCAACAGGAATTACTTTTGATCGAATACCTTTAT
TTGTTTGATCAGTAGTAATTACAGCTTTATTACTTTTATTATCATTACCAGTATTAGCAG
GAGCTATTACAATACTTTTAACAGACCGAAATCTTAATACATCATTCTTTGACCCTGCAG
GAGGAGGAGATCCAATTTTATACCAACATTTATTTAATAATTAATAATTCCATAAGTAGAAATATAAGAGTATAAACCACATAGAACC

TAAAAAATAAGAAAAATTATAATTATTTAAAGCCTTATTATAAAAAAATAAAGAAACATT

AGAAATTATATAACCTATAATACCCCCAACAATACAAACAAATAATGTTAATAATTTTAA

ATAAGAAGGAAGAACAACTACTATAGGAGTAGGAAAAATCAATCATCTTAACATTCTCCC

ACCAAAAATTCTTAAAATTAATAAACCTATTATACTTTTTAATATTACTCAACCTTCATC

ATTTAATATATTTAAAGAAGAAAAATTAGAATCACCA

>ITTVSA
AGAATTCTTATTCGAGCAGAATTAGGTCAC
CCTGGTGCATTAATTGGTGATGATCAAATTTATAATGTAATTGTTACAGCCCATGCTTTC
ATTATAATTTTTTTTATAGTAATACCAATTATAATTGGAGGATTTGGAAATTGATTAGTG
CCAATTATACTAGGAGCTCCAGATATAGCCTTCCCTCGGATAAACAATATAAGTTTTTGA
CTTTTACCTCCTGCATTAACATTGCTTCTAGTAAGTAGTATAGTAGAAAATGGAGCTGGA
ACAGGTTGAACTGTATACCCTCCTTTATCTTCTAATATTGCTCATGGAGGAGCTTCTGTT
GATTTAGCTATTTTTTCTCTCCATTTAGCTGGAATTTCTTCAATTCTAGGAGCAGTAAAT
TTTATTACTACAGTTATTAATATACGATCAACAGGAATCACTTTTGATCGAATACCTTTA
TTTGTATGATCTGTAGTAATCACAGCCCTACTTTTATTACTTTCTTTACCTGTACTTGCC
GGAGCTATTACTATATTATTAACTGATCGAAATATTAATACTTCATTTTTTGACCCGGCA
GGAGGAGGAGATCCTATTCTATATCAACATTTATTTAATAATTTATAATCCCATAAGTAGAAATATAAGGCATAAATCAT

ATTGAACCTAAAAAATAAGAACTATTATATATATTTAAAGCCTTATTAGAAAAAAATAAA

GAAATATTAGAAATTAAATAACCTATTAATCCTCCTACAATACACACAAATAAAGTTAAT

AACTTTAAATAATAAGGTAAAACAATTACCATAGGACTAGGAAAAATTAATCAACTTAAT

ATTCTACCCCCAAAAATTCTTAAAATTAATAATCCTATTATACTTTTTAATATAACTCAA

CCCTCATCATTTAATATATTTAAAGAAGAAAAATTCGAATCTCCA
>UKCvo
AGAATTTTAATTCGAGCTGAACTAGGGCATC
CTGGAGCATTAATTGGAGATGACCAAATTTATAATGTAATTGTTACAGCTCATGCTTTTA
TTATAATTTTTTTTATAGTAATACCAATTATAATTGGAGGGTTTGGAAATTGATTAGTTC
CTTTAATATTAGGAGCCCCAGATATAGCATTCCCTCGAATAAATAATATAAGTTTCTGAC
TTTTACCTCCTGCATTAACTTTATTATTAGTAAGTAGTATAGTAGAAAACGGAGCTGGAA
CTGGATGAACTGTTTATCCACCTTTATCTTCTAATATTGCACATGGAGGAGCTTCTGTTG
ATTTAGCTATTTTTTCTTTACATTTAGCAGGAATTTCTTCAATTTTAGGAGCTGTAAATT
TTATTACTACAGTTATTAATATACGATCAACAGGTATTACCTTCGACCGAATACCATTAT
TTGTTTGATCAGTAGTAATTACAGCCTTATTACTTTTATTATCTTTACCAGTATTAGCAG
GAGCTATTACTATATTATTAACAGATCGAAATCTTAATACTTCATTCTTTGACCCAGCAG
GAGGAGGAGATCCAATTTTATACCAACACTTATTTAATAATTAATAATTCCGTATGTAGAAATATAAGGCATAAACCACAT

TGACCCTAAAAAATAAGAAGAATTATAGTTATTTAAAGCCTTATTATAAAAAAATAAAGA

AATATTAGAAATTAAATACCCTCTAATTCCTCCTACAATACAAACAAATAAAGTTAATAA

CTTTAAATAAGAAGGTAGAACTACTACTACTGGTGTAGGAAAAATTAATCAACTTAACAT

TCTACCCCCAAAAATTCTTAAAATTAATAACCCTATTATACTCTTTAACATAACTCAACC

TTCATCATTTAATATATTTAAAGAAGAAAAATTTGAATCCCCA

>ITChalb

AGAATTCTAATTCGAGCTGAATTAGGACAT

CCTGGAGCACTAATTGGAGATGACCAAATTTATAATGTAATTGTAACAGCTCATGCCTTT

ATTATAATTTTCTTTATAGTAATACCAATTATAATTGGAGGATTTGGAAATTGACTAGTT

CCTTTAATATTAGGAGCCCCAGATATAGCTTTCCCACGAATAAATAATATAAGTTTCTGA

CTTTTACCTCCTGCATTAACTTTACTATTAGTAAGTAGTATAGTAGAAAATGGAGCTGGA

ACAGGATGAACTGTTTATCCACCTTTATCATCTAATATTGCTCATGGTGGAGCATCAGTT

GATTTAGCTATTTTTTCTTTACACTTAGCTGGAATTTCATCAATTTTAGGAGCTGTAAAT

TTTATTACAACTGTTATTAATATACGATCTACAGGAATCACATTTGATCGAATACCTTTA

TTCGTATGATCTGTAGTTATTACTGCTCTTCTTTTATTATTATCATTACCAGTATTAGCC

GGTGCAATTACTATATTATTAACTGATCGAAATTTAAATACTTCATTCTTTGATCCAGCA

GGAGGAGGAGATCCTATTTTATATCAACATTTATTTAATAATTAATAATACCATATGTAGAAATATATGGTATAAATCAT

ATTGAACCTAAAAAATAAGAAGAATTATAATTATTTAATGCCTTATTATAAAAAAATAAT

GAAACATTAGAAATTATATAACCACTTACTCCTCCTACAATACAAACAAATATAGTTAAC

AACTTTAAATATATTGGAAGAACTACTAACACTGGAGTAGGAAAAATTAATCATCTTAAT

ATTCTCCCTCCAAAAATTCTTAGAATCAATAAACCTATTATACTTTTTAATATTACTCAA

CCTTCATCATTTAATATATTTAAAGATGAAAAATTTGAATCTCCA

>ITMOII3Cvi

AGAATTCTAATTCGAGCCGAACTAGGACATCCTG

GAGCATTAATTGGAGATGACCAAATTTATAATGTAATTGTTACAGCTCATGCTTTTATTA

TAATTTTTTTTATAGTAATACCAATTATAATTGGAGGATTTGGTAATTGATTAGTCCCTT

TAATATTAGGAGCTCCAGATATAGCCTTCCCTCGGATAAACAATATAAGTTTCTGACTTT

TACCTCCTGCATTAACTTTACTATTAGTAAGTAGTATAGTAGAAAACGGAGCTGGAACAG

GATGAACTGTTTACCCACCTTTATCTTCTAATATCGCTCATGGAGGAGCTTCTGTTGATT

TAGCTATTTTTTCTTTACACTTAGCAGGAATTTCTTCAATTTTAGGAGCTGTAAATTTTA

TTACTACAGTTATTAATATACGATCAACAGGAATTACATTCGACCGAATACCATTATTTG

TTTGATCTGTAGTAATTACAGCTTTATTACTTTTATTATCTTTACCAGTATTAGCAGGTG

CTATTACTATATTATTAACAGATCGAAATCTTAATACATCATTCTTTGACCCAGCAGGAG

GAGGAGACCCAATCTTGTACCAACATTTATTTAATAATTGATAATTCCATATGTAGAAATATAAGGTATAAATCATATT
GAACCTAAAAAGTAAGAAGAATTATAATTATTTAAAGCCTTATTATAAAAAAATAAAGAA
ATATTAGAAATTAAATAACCTCTAACTCCTCCTACAATACAAACAAATAAAGTTAATAAT
TTTAAATAAGAAGGAAGAACTACTACTACTGGTGTAGGAAAAATTAATCAACTTAATATA
CTACCCCCAAAAATTCTCAAAATTAATAGTCCTATTATACTTTTTAATATAACTCAACCT
TCATCATTTAGTATATTTAAAGAAGAAAAATTTGAATCCCCA

>FanniaL

AGTATTCTAATTCGAGCTGAATTAGGACATCCAGGAGC

ACTAATTGGAGATGATCAAATTTATAATGTAATTGTAACAGCTCATGCTTTTATTATAAT

TTTTTTCATAGTAATACCTATTATAATTGGAGGATTTGGTAATTGATTAGTTCCACTTAT

ACTAGGAGCTCCTGATATAGCATTTCCTCGAATAAACAATATAAGCTTTTGATTATTACC

TCCAGCATTAACTTTACTTCTGGTAAGTAGTATAGTAGAAAATGGAGCTGGTACAGGATG

AACTGTCTACCCTCCACTTTCATCTAATATCGCACATGGAGGAGCTTCAGTTGATTTAGC

AATTTTCTCTCTTCATTTAGCTGGAATTTCATCTATTCTAGGAGCTGTAAATTTTATTAC

TACTGTAATTAATATACGATCAACTGGAATTACCTTAGATCGAATACCTTTATTTGTTTG

ATCTGTAGTAATTACAGCACTATTACTTCTTTTATCTTTACCAGTCTTAGCTGGAGCAAT

TACTATATTATTAACAGATCGTAATTTAAATACCTCATTTTTTGATCCAGCAGGAGGTGG

TGATCCAATTTTATATCAACATTTATTTGATGATTAATTAAACCATAAGTAGAAATATAAGGTATAAACCATATTGAACCTAAA

AAATATGAAAAATTATATATATTTAAAGCCCTATTATAAAAAAATAATGAAATATTTGAA

ATAAAATACCCTATAATCCCACCAATTAAACAAACAAATAAAGTTAATATTTTTAAATGT

ATTGGTAAAACTACAACAATAGGAGTTGAAAATATTAATCATCTTAGTATTCTTCCTCCA

AAAATTCTTAAAATTAAAAGTCCCATTATTCTTTTTAGTATAATTCATCTTTCATCATTC

AACAAATTTAAAGAACTAAAATTTGAATCCCCA

>FanniaP

AGTATTCTAATTCGAGCTGAATTAGG

ACATCCAGGAGCACTAATTGGAGATGATCAAATTTATAATGTAATTGTAACAGCTCATGC

TTTTATTATAATTTTTTTCATAGTAATACCTATTATAATTGGAGGATTTGGTAATTGATT

AGTTCCACTTATACTAGGAGCTCCTGATATAGCATTTCCTCGAATAAACAATATAAGCTT

TTGATTATTACCTCCAGCATTAACTTTACTTCTGGTAAGTAGTATAGTAGAAAATGGAGC

TGGTACAGGATGAACTGTCTACCCTCCACTTTCATCTAATATCGCACATGGAGGAGCTTC

AGTTGATTTAGCAATTTTCTCTCTTCATTTAGCTGGAATTTCATCTATTCTAGGAGCTGT

AAATTTTATTACTACTGTAATTAATATACGATCAACTGGAATTACCTTAGATCGAATACC

TTTATTTGTTTGATCTGTAGTAATTACAGCACTATTACTTCTTTTATCTTTACCAGTCTT

AGCTGGAGCAATTACTATATTATTAACAGATCGTAATTTAAATACCTCATTTTTTGATCC

AGCAGGAGGTGGTGATCCAATTTTATATCAACATTTATTT AATGATTAATTAAACCATAAGTAGAAATATAAGGTATAAACCATATTGAACCTAAA

AAATATGAAAAATTATATATATTTAAAGCCCTATTATAAAAAAATAATGAAATATTTGAA

ATAAAATACCCTATAATCCCACCAATTAAACAAACAAATAAAGTTAATATTTTTAAATGT

ATTGGTAAAACTACAACAATAGGAGTTGAAAATATTAATCATCTTAGTATTCTTCCTCCA

AAAATTCTTAAAATTAAAAGTCCCATTATTCTTTTTAGTATAATTCATCTTTCATCATTC

AACAAATTTAAAGAACTAAAATTTGAATCCCCA

>DataCvi1

AGAATTCTAATTCGAGCC

GAACTAGGACATCCTGGAGCATTAATTGGAGATGACCAAATTTATAATGTAATTGTTACAGCTCATGCTT

TTATTATAATTTTTTTTATAGTAATACCAATTATAATTGGAGGATTTGGTAATTGATTAGTCCCTTTAAT

ATTAGGAGCTCCAGATATAGCCTTCCCTCGGATAAACAATATAAGTTTCTGACTTTTACCTCCTGCATTA

ACTTTACTATTAGTAAGTAGTATAGTAGAAAACGGAGCTGGAACAGGATGAACTGTTTACCCACCTTTAT

CTTCTAATATCGCTCATGGAGGAGCTTCTGTTGATTTAGCTATTTTTTCTTTACACTTAGCAGGAATTTC

TTCAATTTTAGGAGCTGTAAATTTTATTACTACAGTTATTAATATACGATCAACAGGAATTACATTCGAC

CGAATACCATTATTTGTTTGATCTGTAGTAATTACAGCTTTATTACTTTTATTATCTTTACCAGTATTAG

CAGGTGCTATTACTATATTATTAACAGATCGAAATCTTAATACATCATTCTTTGACCCAGCAGGAGGAGG

AGACCCAATCTTGTACCAACATTTATTT AATAATTAATAATTCCATATGTAGAAATATAAGGTATAAATCATATTGAACCTAAAAAGTAAG

AAGAATTATAATTATTTAAAGCCTTATTATAAAAAAATAAAGAAATATTAGAAATTAAATAACCTCTAAC

TCCTCCTACAATACAAACAAATAAAGTTAATAATTTTAAATAAGAAGGAAGAACTACTACTACTGGTGTA

GGAAAAATTAATCAACTTAATATACTACCCCCAAAAATTCTCAAAATTAATAGTCCTATTATACTTTTTA

ATATAACTCAACCTTCATCATTTAGTATATTTAAAGAAGAAAAATTTGAATCCCCA

>DataLse1

AGAATTCTAATTCGAGCT

GAATTAGGACATCCTGGAGCTTTAATTGGAGATGATCAAATTTATAATGTAATTGTTACAGCTCATGCTT

TTATTATAATTTTTTTTATAGTAATGCCAATTATAATTGGAGGATTTGGAAATTGATTAGTTCCATTAAT

ACTAGGAGCTCCAGATATAGCATTCCCTCGAATAAATAATATAAGTTTTTGACTTTTACCTCCTGCATTA

ACTTTATTATTAGTTAGTAGTATAGTAGAAAACGGAGCTGGAACAGGATGAACAGTTTACCCTCCTCTAT

CTTCTAATATTGCTCATGGAGGAGCTTCTGTTGATTTAGCTATTTTCTCTCTTCATTTAGCAGGAATTTC

TTCAATTTTAGGAGCTGTAAATTTTATTACTACAGTTATTAATATACGATCAACAGGAATTACTTTTGAT

CGAATACCTTTATTTGTTTGATCAGTAGTAATTACAGCTTTATTACTTTTATTATCATTACCAGTATTAG

CAGGAGCTATTACAATACTTTTAACAGACCGAAATCTTAATACATCATTCTTTGACCCTGCAGGAGGAGG

AGATCCAATTTTATACCAACATTTATTTAATAATTAATAATTCCATAAGTAGAAATATAAGGTATAAACCACATAGAACCTAAAA

AATAAGAAAAATTATAATTATTTAAAGCCTTATTATAAAAAAATAAAGAAACATGAGAAATTATATAACC

TATAATACCCCCAACAATACAAACAAATAATGTTAATAATTTTAAATAAGAAGGAAGAACAACTACTATA

GGAGTAGGAAAAATCAATCATCTTAACATTCTCCCACCAAAAATTCTTAAAATTAATAAACCTATTATAC

TTTTCAATATTACTCAACCTTCATCATTTAATATATTTAAAGAAGAAAAATTAGAATCACCA

>DataLse2

AGAATTCTAATTCGAGCT

GAATTAGGACATCCTGGAGCTTTAATTGGAGATGATCAAATTTATAATGTAATTGTTACAGCTCATGCTT

TTATTATAATTTTTTTTATAGTAATGCCAATTATAATTGGAGGATTTGGAAATTGATTAGTTCCATTAAT

ACTAGGAGCTCCAGATATAGCATTCCCTCGAATAAATAATATAAGTTTTTGACTTTTACCTCCTGCATTA

ACTTTATTATTAGTTAGTAGTATAGTAGAAAACGGAGCTGGAACAGGATGAACAGTTTACCCTCCTCTAT

CTTCTAATATTGCTCATGGAGGAGCTTCTGTTGATTTAGCTATTTTCTCTCTTCATTTAGCAGGAATTTC

TTCAATTTTAGGAGCTGTAAATTTTATTACTACAGTTATTAATATACGATCAACAGGAATTACTTTTGAT

CGAATACCTTTATTTGTTTGATCAGTAGTAATTACAGCTTTATTACTTTTATTATCATTACCAGTATTAG

CAGGAGCTATTACAATACTTTTAACAGACCGAAATCTTAATACATCATTCTTTGACCCTGCAGGAGGAGG

AGATCCAATTTTATATCAACATTTATTTAATAATTAATAATTCCATAAGTAGAAATATAAGGTATAAACCACATAGAACCTAAAA

AATAAGAAAAATTATAATTATTTAAAGCCTTATTATAAAAAAATAAAGAAACATGAGAAATTATATAACC

TATAATACCCCCAACAATACAAACAAATAATGTTAATAATTTTAAATAAGAAGGAAGAACAACTACTATA

GGAGTAGGAAAAATCAATCATCTTAACATTCTCCCACCAAAAATTCTTAAAATTAATAAACCTATTATAC

TTTTCAATATTACTCAACCTTCATCATTTAATATATTTAAAGAAGAAAAATTAGAATCACCA

>DataLill1

AGAATTTTAATTCGAGCT

GAATTAGGACACCCTGGTGCATTAATTGGAGATGACCAAATTTATAATGTAATTGTTACAGCTCATGCTT

TTATTATAATTTTCTTTATAGTAATGCCAATTATAATTGGAGGATTTGGAAATTGATTAGTTCCTTTAAT

ATTAGGAGCTCCAGATATAGCATTCCCTCGAATAAATAATATAAGATTTTGACTTTTACCTCCTGCATTA

ACTTTACTATTAGTAAGTAGTATAGTAGAAAACGGAGCTGGAACAGGATGAACAGTTTACCCTCCTTTAT

CATCTAATATCGCTCATGGAGGAGCTTCTGTTGATTTAGCTATTTTTTCTCTTCATTTAGCAGGAATTTC

ATCAATTTTAGGAGCTGTAAATTTTATTACTACAGTTATTAATATACGATCAACAGGGATTACTTTTGAT

CGAATGCCTTTATTCGTTTGATCAGTAGTAATTACAGCTTTATTACTTTTATTATCATTACCAGTATTAG

CTGGAGCTATTACTATACTTTTAACTGATCGAAATCTTAATACTTCATTCTTTGACCCAGCAGGAGGAGG

AGACCCAATTTTATACCAACATTTATTTAATAATTAATAATTCCGTAAGTAGAAATATAAGGTATAAACCACATTGACCCTAAAA

AATAAGAAAAATTATAATTATTTAAAGCCTTATTATAAAAAAATAAGGAAATATTTGAAATTATATACCC

TCTTACTCCTCCTACAATACAAACAAATAATGTTAATAACTTTAAATATGAAGGAAGAACTACAACTACT

GGAGTTGGAAAAATTAATCATCTTAATATTCTTCCTCCAAAAATTCTTAAAATTAATAATCCTATTATAC

TTTTTAATATTACTCAACCTTCATCATTTAATATATTTAAAGAAGAAAAATTTGAATCACCA

>DataLill2

AGAATTTTAATTCGAGCT

GAATTAGGACACCCTGGTGCATTAATTGGAGATGACCAAATTTATAATGTAATTGTTACAGCTCATGCTT

TTATTATAATTTTCTTTATAGTAATGCCAATTATAATTGGAGGATTTGGAAATTGATTAGTTCCTTTAAT

ATTAGGAGCTCCAGATATAGCATTCCCTCGAATAAATAATATAAGATTTTGACTTTTACCTCCTGCATTA

ACTTTACTATTAGTAAGTAGTATAGTAGAAAACGGAGCTGGAACAGGATGAACAGTTTACCCTCCTTTAT

CATCTAATATCGCTCATGGAGGAGCTTCTGTTGATTTAGCTATTTTTTCTCTTCATTTAGCAGGAATTTC

ATCAATTTTAGGAGCTGTAAATTTTATTACTACAGTTATTAATATACGATCAACAGGGATTACTTTTGAT

CGAATGCCTTTATTCGTTTGATCAGTAGTAATTACAGCTTTATTACTTTTATTATCATTACCAGTATTAG

CTGGAGCTATTACTATACTTTTAACTGATCGAAATCTTAATACTTCATTCTTTGACCCAGCAGGAGGAGG

AGACCCAATTTTATACCAACATTTATTTAATAATTAATAATTCCGTAAGTAGAAATATAAGGTATAAACCACATTGACCCTAAAA

AATAAGAAAAATTATAATTATTTAAAGCCTTATTATAAAAAAATAAGGAAATATTTGAAATTATATACCC

TCTTACTCCTCCTACAATACAAACAAATAATGTTAATAACTTTAAATATGAAGGAAGAACTACAACTACT

GGAGTTGGAAAAATTAATCATCTTAATATTCTTCCTCCAAAAATTCTTAAAATTAATAATCCTATTATAC

TTTTTAATATTACTCAACCTTCATCATTTAATATATTTAAAGAAGAAAAATTTGAATCACCA

>DataPrte1

AGAATCCTAATTCGAGCT

GAATTAGGGCACCCTGGAGCACTAATTGGAGATGACCAAATTTATAATGTAATTGTAACGGCTCACGCTT

TTATTATAATTTTCTTTATAGTAATACCAATTATAATTGGAGGATTTGGAAATTGACTAGTTCCCCTTAT

ATTAGGGGCTCCTGATATAGCATTCCCTCGAATAAATAATATAAGTTTCTGACTTTTACCTCCTGCATTA

ACTTTATTATTAGTAAGTAGTATAGTAGAAAATGGGGCTGGAACAGGATGAACTGTTTACCCACCTTTAT

CTTCTAATATTGCTCACGGAGGAGCATCTGTTGATTTAGCTATTTTCTCTCTTCACTTGGCCGGAATTTC

TTCAATTTTAGGAGCTGTAAATTTCATTACAACTGTAATTAATATACGATCTACAGGAATTACATTTGAT

CGAATACCTTTATTTGTTTGATCTGTAGTTATTACTGCTCTTTTACTTTTATTATCTTTACCAGTATTAG

CAGGTGCTATTACTATATTATTAACTGACCGAAATTTAAATACTTCATTTTTTGATCCAGCTGGAGGAGG

AGACCCTATTTTATACCAACATTTATTT AATAATT

AATAATTCCATAAGTAGAAATATAAGGTATAAATCACATTGAACCTAAAAAATAAGAAGAATGATAATTA

TTTAAAGCCTTGTTATAAAAAAATAAAGAAACATTAGAAATTATATACCCTACTAACCCTCCAACAATAC

AAACAAATAATGTTAATAACTTTAAATAAAAAGGTAAAACTACTACTATAGGAGTAGGAAAAATTAACCA

TCTTAACATTCTACCTCCAAAAATTCTTAAAATTAATAAACCTATTATACTCTTTAATATTACTCAACCT

TCATCATTTAACATATTTAAAGAAGAAAAATTAGAATCCCCA

>DataChal1

AGAATTCTAATTCGAGCT

GAATTAGGACATCCTGGAGCACTAATTGGAGATGACCAAATTTATAATGTAATTGTAACAGCTCATGCCT

TTATTATAATTTTCTTTATAGTAATACCAATTATAATTGGAGGATTTGGAAATTGACTAGTTCCTTTAAT

ATTAGGAGCCCCAGATATAGCTTTCCCACGAATAAATAATATAAGTTTCTGACTTTTACCTCCTGCATTA

ACTTTACTATTAGTAAGTAGTATAGTAGAAAATGGAGCTGGAACAGGATGAACTGTTTATCCACCTTTAT

CATCTAATATTGCTCATGGTGGAGCATCAGTTGATTTAGCTATTTTTTCTTTACACTTAGCTGGAATTTC

ATCAATTTTAGGAGCTGTAAATTTTATTACAACTGTTATTAATATACGATCTACAGGAATCACATTTGAT

CGAATACCTTTATTCGTATGATCTGTAGTTATTACTGCTCTTCTTTTATTATTATCATTACCAGTATTAG

CCGGTGCAATTACTATATTATTAACTGATCGAAATTTAAATACTTCATTCTTTGATCCAGCAGGAGGAGG

AGATCCTATTTTATATCAACATTTATTT AATAATTAATAATACCATATGTAGAAATATATGGTATAAATCATATTGAACCTAAAAAATAAGAAGAA

TTATAATTATTTAATGCCTTATTATAAAAAAATAATGAAACATTAGAAATTATATAACCACTTACTCCTC

CTACAATACAAACAAATATAGTTAACAACTTTAAATATATTGGAAGAACTACTAATACTGGAGTAGGAAA

AATTAATCATCTTAATATTCTCCCTCCAAAAATTCTTAAAATCAATAAACCTATTATACTTTTTAATATT

ACTCAACCTTCATCATTTAATATATTTAAAGATGAAAAATTTGAATCTCCA

>DataPhre1

AGAATTCTAATTCGAGCT

GAACTAGGGCACCCTGGAGCTCTAATTGGAGATGACCAAATTTATAACGTAATTGTAACAGCTCATGCTT

TTATTATAATTTTCTTTATAGTTATACCAATTATAATTGGAGGATTTGGAAATTGATTAGTTCCTTTAAT

ATTAGGGGCTCCTGATATAGCTTTCCCACGAATAAACAATATAAGTTTTTGACTTTTACCTCCTGCATTA

ACTCTATTGTTAGTTAGTAGTATAGTAGAAAATGGGGCTGGAACAGGATGAACTGTTTACCCACCTTTAT

CATCTAATATTGCTCATGGAGGAGCATCTGTTGATCTAGCTATTTTCTCTCTTCACTTAGCAGGAATTTC

TTCAATTTTAGGAGCTGTAAATTTCATTACAACTGTAATTAATATACGATCAACTGGAATTACATTTGAT

CGAATACCTTTATTTGTTTGATCTGTAGTTATTACTGCTCTATTACTTTTATTATCTTTACCTGTATTAG

CCGGTGCTATTACTATATTATTAACTGATCGAAATTTAAACACTTCATTCTTTGACCCAGCAGGAGGAGG

AGATCCTATTTTATATCAACACTTATTCAATAATTAATAATTCCATAAGTAGAAATATAAGGTATAAATCATATAGAACCTAAAAAATAAG

AAGAATGATAATTATTTAAAGCCTTATTATAAAAAAATAAAGAAACATGAGAAATCATGTAACCTATTAA

TCCCCCTACAATACAAACAAATAAAGTTAACAACTTTAAATAAAAAGGAAGAACAACTACTATTGGTGTA

GGAAAAATCAACCATCTTAACATTCTTCCCCCAAAAATTCTTAAAATCAATAAACCTAATATACTTTTTA

ATATCACTCAACCTTCATCATTAAGCATGTTCAAAGATGAAAAATTTGAATCTCCA

>DataSaf1

AGAATTCTTATTCGAGCA

GAATTAGGTCATCCTGGTGCATTAATTGGAGATGATCAAATTTATAACGTAATCGTTACAGCTCATGCTT

TTATTATAATTTTCTTTATAGTAATACCAATTATAATTGGAGGGTTTGGAAATTGATTAGTACCAATTAT

ACTAGGAGCCCCAGATATAGCTTTCCCTCGAATAAATAATATAAGATTTTGACTTTTACCTCCAGCATTA

ACACTACTTCTAGTAAGCAGTATAGTAGAAAACGGAGCTGGAACAGGATGAACTGTTTACCCTCCTTTAT

CATCTAATATTGCTCATGGAGGTGCTTCTGTTGATTTAGCTATTTTCTCTCTTCATCTAGCTGGAATCTC

TTCAATTTTAGGAGCCGTAAATTTTATTACTACAGTTATTAATATACGATCTACAGGAATTACTTTTGAT

CGAATACCTTTATTTGTATGATCAGTAGTAATTACAGCTTTACTTCTATTACTCTCATTACCTGTACTTG

CGGGAGCAATTACTATATTATTAACTGACCGAAATATTAATACCTCATTTTTTGATCCTGCAGGAGGAGG

AGACCCTATTTTATATCAACATTTATTTAATAATTTA

TAATCCCATAAGTAGAAATATAAGGCATAAATCATATTGAACCTAAAAAATAAGAACTATTATATATATT

TAAAGCCTTATTAGAAAAAAATAAAGAAATATTAGAAATTAAATAACCTATTAATCCTCCTACAATACAC

ACAAATAAAGTTAATAACTTTAAATAATAAGGTAAAACAATTACCATAGGACTAGGAAAAATTAATCAAC

TTAATATTCTACCCCCAAAAATTCTTAAAATTAATAATCCTATTATACTTTTTAATATAACTCAACCCTC

ATCATTTAATATATTTAAAGAAGAAAAATTCGAATCTCCA

>DataMsc1

AGTATTATAATTCGAGCT

GAATTAGGACACCCCGGTGCTTTAATTGGTGATGATCAAATTTATAATGTAATTGTTACTGCCCATGCAT

TTATTATAATTTTTTTTATAGTAATACCTATTATAATAGGGGGATTTGGAAATTGATTAGTTCCCTTAAT

ATTAGGAGCACCTGATATGGCTTTTCCACGAATAAATAATATAAGTTTTTGAATACTTCCCCCTTCTCTA

ACTCTTTTATTAGCAAGAAGTATAGTAGAAAATGGAGCCGGAACTGGTTGAACAGTTTATCCACCCCTAT

CTTCTAGAATTGCCCATAGAGGAGCTTCAGTCGATTTAGCAATTTTTTCGTTACATCTTGCCGGAATTTC

TTCTATTCTTGGAGCGGTAAATTTTATTACTACAATTATTAATATACGGTCTACAGGAATTACTTTTGAT

CGAATACCTTTATTTGTATGATCAGTAGGTATTACTGCTCTTTTATTATTACTTTCACTACCTGTTCTAG

CAGGTGCTATTACTATACTATTAACAGACCGAAATTTTAATACATCATTCTTTGATCCTGCGGGAGGGGG

AGACCCAATCCTATATCAACATCTATTTGATAATTAATAATTCCATAAGTTGAAATATAAGGCATAAATCATATAGACCCTAAAAAATAAGAAATATTAT

ATAGGTCTAAAGATTTATTATAAAAATATAAATTAACTAATGAAATGAAATAGCCAAATAAACCTCCAAT

AATACAAACAAATAAAGTTAACTGCTTTAGATAAATAGGTAGACAAATCATATAAGGAGTAGGAAAAATT

AATCAATTTAAAATTCTTCCCCCAATAATTGATATAATAAGTAAACCTATTATACCCTTCAATATTACTC

ATCCTTCATCTCTTAAAACATTTAATCTTCTACAATTTAAATCTCCA

**COI+ND5+EF1a sequences**

>ITMOII10Ls
AGAATTCTAATTCGAGCTGAATTAGGACATCCTGGAG
CTTTAATTGGAGATGATCAAATTTATAATGTAATTGTTACAGCTCATGCTTTTATTATAA
TTTTTTTTATAGTAATGCCAATTATAATTGGAGGATTTGGAAATTGATTAGTTCCATTAA
TACTAGGAGCTCCAGATATAGCATTCCCTCGAATAAATAATATAAGTTTTTGACTTTTAC
CTCCTGCATTAACTTTATTATTAGTTAGTAGTATAGTAGAAAACGGAGCTGGAACAGGAT
GAACAGTTTACCCTCCTCTATCTTCTAATATTGCTCATGGAGGAGCTTCTGTTGATTTAG
CTATTTTCTCTCTTCATTTAGCAGGAATTTCTTCAATTTTAGGAGCTGTAAATTTTATTA
CTACAGTTATTAATATACGATCAACAGGAATTACTTTTGATCGAATACCTTTATTTGTTT
GATCAGTAGTAATTACAGCTTTATTACTTTTATTATCATTACCAGTATTAGCAGGAGCTA
TTACAATACTTTTAACAGACCGAAATCTTAATACATCATTCTTTGATCCTGCAGGAGGAG
GAGATCCAATTTTATACCAACATTTATTT GATAATTAATAATTCCATAAGTAGAAATATAAGGTATAAACCACATAGAACCTA
AAAAATAAGAAAAATTATAATTATTTAAAGCCTTATTATAAAAAAATAAAGAAACATGAG
AAATTATATAACCTATAATACCCCCAACAATACAAACAAATAATGTTAATAATTTTAAAT
AAGAAGGAAGAACAACTACTATAGGAGTAGGAAAAATTAATCATCTTAACATTCTCCCAC
CAAAAATTCTTAAAATTAATAAACCTATTATACTTTTCAATATTACTCAACCTTCATCAT
TTAATATATTTAAAGAAGAAAAATTAGAATCACCA GTTGTCTTC
GCTCCCGCTAACATCACCACTGAAGTCAAGTCCGTTGAAATGCACCACGAAGCTCTCGTT
GAAGCCGTCCCCGGTGACAACGTTGGTTTCAACGTAAAGAACGTCTCCGTCAAGGAATTG
CGTCGTGGTTACGTCGCTGGTGACTCCAAGGCTAACCCCCCCAAGGGCGCCGCTGACTTC
ACCGCTCAAGTCATCGTCTTGAACCACCCTGGTCAAATCTCCAACGGTTACACCCCCGTT
TTGGATTGTCACACCGCTCACATTGCTTGCAAATTCGCCGAAATCAAGGAGAAGGTCGAT
>ITMOII1Cvi
AGAATTCTAATTCGAGCCGAACTAGGACATCCTGG
AGCATTAATTGGAGATGACCAAATTTATAATGTAATTGTTACAGCTCATGCTTTTATTAT
AATTTTTTTTATAGTAATACCAATTATAATTGGAGGATTTGGTAATTGATTAGTCCCTTT
AATATTAGGAGCTCCAGATATAGCCTTCCCTCGGATAAACAATATAAGTTTCTGACTTTT
ACCTCCTGCATTAACTTTACTATTAGTAAGTAGTATAGTAGAAAACGGAGCTGGAACAGG
ATGAACTGTTTACCCACCTTTATCTTCTAATATTGCTCATGGAGGAGCTTCTGTTGATTT
AGCTATTTTTTCTTTACACTTAGCAGGAATTTCTTCAATTTTAGGAGCTGTAAATTTTAT
TACTACAGTTATTAATATACGATCAACAGGAATTACATTCGACCGAATACCATTATTTGT
TTGATCTGTAGTAATTACAGCTTTATTACTTTTATTATCTTTACCAGTATTAGCAGGTGC
TATTACTATATTATTAACAGATCGAAATCTTAATACATCATTCTTTGACCCAGCAGGAGG
AGGAGACCCAATCTTGTACCAACATTTATTTAATAATTAATAATTCCATATGTAGAAATATAAGGTATAAATCAT
ATTGAACCTAAAAAGTAAGAAGAATTATAATTATTTAAAGCCTTATTATAAAAAAATAAA
GAAATATTAGAAATTAAATAACCTCTAACTCCTCCTACAATACAAACAAATAAAGTTAAT
AATTTTAAATAAGAAGGAAGAACTACTACTACTGGTGTAGGAAAAATTAATCAACTTAAT
ATACTACCCCCAAAAATTCTCAAAATTAATAGTCCTATTATACTTTTTAATATAACTCAA
CCTTCATCATTTAGTATATTTAAAGAAGAAAAATTTGAATCCCCA

GTTGTCTTCGCTCCCGCTAACATCACCACTGAAGTCAAG
TCCGTAGAAATGCACCACGAAGCTCTCGTTGAAGCTGTTCCCGGTGACAACGTTGGTTTC
AACGTTAAGAACGTCTCCGTCAAGGAATTGCGTCGTGGTTACGTCGCTGGTGACTCCAAG
GCTAGCCCCCCCAAGGGTGCTGCTGACTTCACCGCTCAAGTCATCGTCTTGAACCACCCT
GGTCAAATCTCCAACGGTTACACTCCCGTTTTGGATTGTCACACCGCTCACATTGCTTGC
AAATTCGCCGAAATCAAGGAGAAGGTCGAT

>ITMOII2Cvi
AGAATTCTAATTCGAGCCGAACTAGGACATCCTGG
AGCATTAATTGGAGATGACCAAATTTATAATGTAATTGTTACAGCTCATGCTTTTATTAT
AATTTTTTTTATAGTAATACCAATTATAATTGGAGGATTTGGTAATTGATTAGTCCCTTT
AATATTAGGAGCTCCAGATATAGCCTTCCCTCGGATAAACAATATAAGTTTCTGACTTTT
ACCTCCTGCATTAACTTTACTATTAGTAAGTAGTATAGTAGAAAACGGAGCTGGAACAGG
ATGAACTGTTTACCCACCTTTATCTTCTAATATTGCTCATGGAGGAGCTTCTGTTGATTT
AGCTATTTTTTCTTTACACTTAGCAGGAATTTCTTCAATTTTAGGAGCTGTAAATTTTAT
TACTACAGTTATTAATATACGATCAACAGGAATTACATTCGACCGAATACCATTATTTGT
TTGATCTGTAGTAATTACAGCTTTATTACTTTTATTATCTTTACCAGTATTAGCAGGTGC
TATTACTATATTATTAACAGATCGAAATCTTAATACATCATTCTTTGACCCAGCAGGAGG
AGGAGACCCAATCTTGTACCAACATTTATTT AATAATTAATAATTCCATATGTAGAAATATAAGGTATAAATCATAT
TGAACCTAAAAAGTAAGAAGAATTATAATTATTTAAAGCCTTATTATAAAAAAATAAAGA
AATATTAGAAATTAAATAACCTCTAACTCCTCCTACAATACAAACAAATAAAGTTAATAA
TTTTAAATAAGAAGGAAGAACTACTACTACTGGTGTAGGAAAAATTAATCAACTTAATAT
ACTACCCCCAAAAATTCTCAAAATTAATAGTCCTATTATACTTTTTAATATAACTCAACC
TTCATCATTTAGTATATTTAAAGAAGAAAAATTTGAATCCCCA

GTTGTCTTCGCTCCCGCTAACATCACCACTGAAGTCAAG
TCCGTAGAAATGCACCACGAAGCTCTCGTTGAAGCTGTTCCCGGTGACAACGTTGGTTTC
AACGTTAAGAACGTCTCCGTCAAGGAATTGCGTCGTGGTTACGTCGCTGGTGACTCCAAG
GCTAGCCCCCCCAAGGGTGCTGCTGACTTCACCGCTCAAGTCATCGTCTTGAACCACCCT
GGTCAAATCTCCAACGGTTACACTCCCGTTTTGGATTGTCACACCGCTCACATTGCTTGC
AAATTCGCCGAAATCAAGGAGAAGGTCGAT

>ITMOII3Cvi
AGAATTCTAATTCGAGCCGAACTAGGACATCCTG

GAGCATTAATTGGAGATGACCAAATTTATAATGTAATTGTTACAGCTCATGCTTTTATTA

TAATTTTTTTTATAGTAATACCAATTATAATTGGAGGATTTGGTAATTGATTAGTCCCTT

TAATATTAGGAGCTCCAGATATAGCCTTCCCTCGGATAAACAATATAAGTTTCTGACTTT

TACCTCCTGCATTAACTTTACTATTAGTAAGTAGTATAGTAGAAAACGGAGCTGGAACAG

GATGAACTGTTTACCCACCTTTATCTTCTAATATCGCTCATGGAGGAGCTTCTGTTGATT

TAGCTATTTTTTCTTTACACTTAGCAGGAATTTCTTCAATTTTAGGAGCTGTAAATTTTA

TTACTACAGTTATTAATATACGATCAACAGGAATTACATTCGACCGAATACCATTATTTG

TTTGATCTGTAGTAATTACAGCTTTATTACTTTTATTATCTTTACCAGTATTAGCAGGTG

CTATTACTATATTATTAACAGATCGAAATCTTAATACATCATTCTTTGACCCAGCAGGAG

GAGGAGACCCAATCTTGTACCAACATTTATTTAATAATTGATAATTCCATATGTAGAAATATAAGGTATAAATCATATT
GAACCTAAAAAGTAAGAAGAATTATAATTATTTAAAGCCTTATTATAAAAAAATAAAGAA
ATATTAGAAATTAAATAACCTCTAACTCCTCCTACAATACAAACAAATAAAGTTAATAAT
TTTAAATAAGAAGGAAGAACTACTACTACTGGTGTAGGAAAAATTAATCAACTTAATATA
CTACCCCCAAAAATTCTCAAAATTAATAGTCCTATTATACTTTTTAATATAACTCAACCT
TCATCATTTAGTATATTTAAAGAAGAAAAATTTGAATCCCCA

GTTGTCTTCGCTCCCGCTAACATCACCACTGAAGT
CAAGTCCGTAGAAATGCACCACGAAGCTCTCGTTGAAGCTGTTCCCGGTGACAACGTTGG
TTTCAACGTTAAGAACGTCTCCGTCAAGGAATTGCGTCGTGGTTACGTCGCTGGTGACTC
CAAGGCTAGCCCCCCCAAGGGTGCTGCTGACTTCACCGCTCAAGTCATCGTCTTGAACCA
CCCTGGTCAAATCTCCAACGGTTACACTCCCGTTTTGGATTGTCACACCGCTCACATTGC
TTGCAAATTCGCCGAAATCAAGGAGAAGGTCGAT

>ITMOII4Cvi
AGAATTCTAATTCGAGCCGAACTAGGACATCCTGGAG
CATTAATTGGAGATGACCAAATTTATAATGTAATTGTTACAGCTCATGCTTTTATTATAA
TTTTTTTTATAGTAATACCAATTATAATTGGAGGATTTGGTAATTGATTAGTCCCTTTAA
TATTAGGAGCTCCAGATATAGCCTTCCCTCGGATAAACAATATAAGTTTCTGACTTTTAC
CTCCTGCATTAACTTTACTATTAGTAAGTAGTATAGTAGAAAACGGAGCTGGAACAGGAT
GAACTGTTTACCCACCTTTATCTTCTAATATCGCTCATGGAGGAGCTTCTGTTGATTTAG
CTATTTTTTCTTTACACTTAGCAGGAATTTCTTCAATTTTAGGAGCTGTAAATTTTATTA
CTACAGTTATTAATATACGATCAACAGGAATTACATTCGACCGAATACCATTATTTGTTT
GATCTGTAGTAATTACAGCTTTATTACTTTTATTATCTTTACCAGTATTAGCAGGTGCTA
TTACTATATTATTAACAGATCGAAATCTTAATACATCATTCTTTGACCCAGCAGGAGGAG
GAGACCCAATCTTGTACCAACATTTATTT AATAATTAATAATTCCATATGTAGAAATATAAGGTATAAATCATATTGAAC
CTAAAAAGTAAGAAGAATTATAATTATTTAAAGCCTTATTATAAAAAAATAAAGAAATAT
TAGAAATTAAATAACCTCTAACTCCTCCTACAATACAAACAAATAAAGTTAATAATTTTA
AATAAGAAGGAAGAACTACTACTACTGGTGTAGGAAAAATTAATCAACTTAATATACTAC
CCCCAAAAATTCTCAAAATTAATAGTCCTATTATACTTTTTAATATAACTCAACCTTCAT
CATTTAGTATATTTAAAGAAGAAAAATTTGAATCCCCAGTTGTCTTCGCTCCCGCTAACATCACCACTGAAG
TCAAGTCCGTAGAAATGCACCACGAAGCTCTCGTTGAAGCTGTTCCCGGTGACAACGTTG
GTTTCAACGTTAAGAACGTCTCCGTCAAGGAATTGCGTCGTGGTTACGTCGCTGGTGACT
CCAAGGCTAGCCCCCCCAAGGGTGCTGCTGACTTCACCGCTCAAGTCATCGTCTTGAACC
ACCCTGGTCAAATCTCCAACGGTTACACTCCCGTTTTGGATTGTCACACCGCTCACATTG
CTTGCAAATTCGCCGAAATCAAGGAGAAGGTCGAT

>ITMOII5Cvi
AGAATTCTAATTCGAGCCGAACTAGGACATC
CTGGAGCATTAATTGGAGATGACCAAATTTATAATGTAATTGTTACAGCTCATGCTTTTA
TTATAATTTTTTTTATAGTAATACCAATTATAATTGGAGGATTTGGTAATTGATTAGTCC
CTTTAATATTAGGAGCTCCAGATATAGCCTTCCCTCGGATAAACAATATAAGTTTCTGAC
TTTTACCTCCTGCATTAACTTTACTATTAGTAAGTAGTATAGTAGAAAACGGAGCTGGAA
CAGGATGAACTGTTTACCCACCTTTATCTTCTAATATTGCTCATGGAGGAGCTTCTGTTG
ATTTAGCTATTTTTTCTTTACACTTAGCAGGAATTTCTTCAATTTTAGGAGCTGTAAATT
TTATTACTACAGTTATTAATATACGATCAACAGGAATTACATTCGACCGAATACCATTAT
TTGTTTGATCTGTAGTAATTACAGCTTTATTACTTTTATTATCTTTACCAGTATTAGCAG
GTGCTATTACTATATTATTAACAGATCGAAATCTTAATACATCATTCTTTGACCCAGCAG
GAGGAGGAGACCCAATCTTGTACCAACATTTATTTAATAATTAATAATTCCATATGTAGAAATATAAGGTATAAATCATATTGAAC
CTAAAAAATAAGAAGAATTATAATTATTTAAAGCCTTATTATAAAAAAATAAAGAAATAT
TAGAAATTAAATAACCTCTAACTCCTCCTACAATACAAACAAATAAAGTTAATAATTTTA
AATAAGAAGGAAGAACTACTACTACTGGTGTAGGAAAAATTAATCAACTTAATATACTAC
CCCCAAAAATTCTCAAAATTAATAGTCCTATTATACTTTTTAATATAACTCAACCTTCAT
CATTTAGTATATTTAAAGAAGAAAAATTTGAATCCCCA

GTTGTCTTCGCTCCCGCTAACATCACCACTGAAGT
CAAGTCCGTAGAAATGCACCACGAAGCTCTCGTTGAAGCTGTTCCCGGTGACAACGTTGG
TTTCAACGTTAAGAACGTCTCCGTCAAGGAATTGCGTCGTGGTTACGTCGCTGGTGACTC
CAAGGCTAGCCCCCCCAAGGGTGCTGCTGACTTCACCGCTCAAGTCATCGTCTTGAACCA
CCCTGGTCAAATCTCCAACGGTTACACTCCCGTTTTGGATTGTCACACCGCTCACATTGC
TTGCAAATTCGCCGAAATCAAGGAGAAGGTCGAT

>ITMOII6Ls
AGAATTCTAATTCGAGCTGAATTAGGACATCCT
GGAGCTTTAATTGGAGATGATCAAATTTATAATGTAATTGTTACAGCTCATGCTTTTATT
ATAATTTTTTTTATAGTAATGCCAATTATAATTGGAGGATTTGGAAATTGATTAGTTCCA
TTAATACTAGGAGCTCCAGATATAGCATTCCCTCGAATAAATAATATAAGTTTTTGACTT
TTACCTCCTGCATTAACTTTATTATTAGTTAGTAGTATAGTAGAAAACGGAGCTGGAACA
GGATGAACAGTTTACCCTCCTCTATCTTCTAATATTGCTCATGGAGGAGCTTCTGTTGAT
TTAGCTATTTTCTCTCTTCATTTAGCAGGAATTTCTTCAATTTTAGGAGCTGTAAATTTT
ATTACTACAGTTATTAATATACGATCAACAGGAATTACTTTTGATCGAATACCTTTATTT
GTTTGATCAGTAGTAATTACAGCTTTATTACTTTTATTATCATTACCAGTATTAGCAGGA
GCTATTACAATACTTTTAACAGACCGAAATCTTAATACATCATTCTTTGACCCTGCAGGA
GGAGGAGATCCAATTTTATACCAACATTTATTTAATAATTAATAATTCCATAAGTAGAAATATAAGGTATAAACCAC
ATAGAACCTAAAAAATAAGAAAAATTATAATTATTTAAAGCCTTGTTATAAAAAAATAAA
GAAACATTAGAAATTATATAACCTATAATACCCCCAACAATACAAACAAATAATGTTAAT
AATTTTAAATAAGAAGGAAGAACAATTACTATAGGAGTAGGAAAAATCAATCATCTTAAC
ATTCTCCCACCAAAAATTCTTAAAATTAATAAACCTATTATACTTTTCAATATTACTCAA
CCTTCATCATTTAATATATTTAAAGAAGAAAAATTAGAATCACCA

GTTGTCTTCGCTCCCGCTAACATCACCACTGAAGTCAAG
TCCGTTGAAATGCACCACGAAGCTCTCGTTGAAGCCGTCCCCGGTGACAACGTTGGTTTC
AACGTAAAGAACGTCTCCGTCAAGGAATTGCGTCGTGGTTACGTCGCTGGTGACTCCAAG
GCTAACCCCCCCAAGGGCGCCGCTGACTTCACCGCTCAAGTCATCGTCTTGAACCACCCT
GGTCAAATCTCCAACGGTTACACCCCCGTTTTGGATTGTCACACCGCTCACATTGCTTGC
AAATTCGCCGAAATCAAGGAGAAGGTCGAT

>ITMOII7Ls
AGAATTCTAATTCGAGCTGAATTAGGA
CATCCTGGAGCTTTAATTGGAGATGATCAAATTTATAATGTAATTGTTACAGCTCATGCT
TTTATTATAATTTTTTTTATAGTAATGCCAATTATAATTGGAGGATTTGGAAATTGATTA
GTTCCATTAATACTAGGAGCTCCAGATATAGCATTCCCTCGAATAAATAATATAAGTTTT
TGACTTTTACCTCCTGCATTAACTTTATTATTAGTTAGTAGTATAGTAGAAAACGGAGCT
GGAACAGGATGAACAGTTTACCCTCCTCTATCTTCTAATATTGCTCATGGAGGAGCTTCT
GTTGATTTAGCTATTTTCTCTCTTCATTTAGCAGGAATTTCTTCAATTTTAGGAGCTGTA
AATTTTATTACTACAGTTATTAATATACGATCAACAGGAATTACTTTTGATCGAATACCT
TTATTTGTTTGATCAGTAGTAATTACAGCTTTATTACTTTTATTATCATTACCAGTATTA
GCAGGAGCTATTACAATACTTTTAACAGACCGAAATCTTAATACATCATTCTTTGACCCT
GCAGGAGGAGGAGATCCAATTTTTATACCAACATTTATTAATAATTAATAATTCCATAAGTAGAAATATAAGGTATAAACCACATAGAACCT
AAAAAATAAGAAAAATTATAATTATTTAAAGCCTTATTATAAAAAAATAAAGAAACATTA
GAAATTATATAACCTATAATACCCCCAACAATACAAACAAATAATGTTAATAATTTTAAA
TAAGAAGGAAGAACAACTACTATAGGAGTAGGAAAAATCAATCATCTTAACATTCTCCCA
CCAAAAATTCTTAAAATTAATAAACCTATTATACTTTTCAATATTACTCAACCTTCATCA
TTTAATATATTTAAAGAAGAAAAATTAGAATCACCAGTTGTCTTCGCTCCCGCTAACATCACCACTGAAGTCAAGT
CCGTTGAAATGCACCACGAAGCTCTCGTTGAAGCCGTCCCCGGTGACAACGTTGGTTTCA
ACGTAAAGAACGTCTCCGTCAAGGAATTGCGTCGTGGTTACGTCGCTGGTGACTCCAAGG
CCAACCCCCCCAAGGGCGCCGCTGACTTCACCGCTCAAGTCATCGTCTTGAACCACCCTG
GTCAAATCTCCAACGGTTACACCCCCGTTTTGGATTGTCACACCGCTCACATTGCTTGCA
AATTCGCCGAAATCAAGGAGAAGGTCGAT

>ITMOII8Ls
AGAATTCTAATTCGAGCTGAATTAGGACATCCT
GGAGCTTTAATTGGAGATGATCAAATTTACAATGTAATTGTTACAGCTCATGCTTTTATT
ATAATTTTTTTTATAGTAATGCCAATTATAATTGGAGGATTTGGAAATTGATTAGTTCCA
TTAATACTAGGAGCTCCAGATATAGCATTCCCTCGAATAAATAATATAAGTTTTTGACTT
TTACCTCCTGCATTAACTTTATTATTAGTTAGTAGTATAGTAGAAAACGGAGCTGGAACA
GGATGAACAGTTTACCCTCCTCTATCTTCTAATATTGCTCATGGAGGAGCTTCTGTTGAT
TTAGCTATTTTCTCTCTTCATTTAGCAGGAATTTCTTCAATTTTAGGAGCTGTAAATTTT
ATTACTACAGTTATTAATATACGATCAACAGGAATTACTTTTGATCGAATACCTTTATTT
GTTTGATCAGTAGTAATTACAGCTTTATTACTTTTATTATCATTACCAGTATTAGCGGGA
GCTATTACAATACTTTTAACAGACCGAAATCTTAATACATCATTCTTTGACCCTGCAGGA
GGAGGAGATCCAATTTTATACCAACATTTATTTAATAATTAATAATTCCATAAGTAGAAATATATGGTATAAACCACAT
AGAACCTAAAAAATAAGAAAAATTATAATTATTTAAAGCCTTATTATAAAAAAATAAAGA
AACATTAGAAATTATATAACCTATAATACCCCCAACAATACAAACAAATAATGTTAATAA
TTTTAAATAAGAAGGAAGAACAACTACTATAGGAGTAGGAAAAATCAATCATCTTAACAT
TCTCCCACCAAAAATTCTTAAAATTAATAAACCTATTATACTTTTCAATATTACTCAACC
TTCATCATTTAATATATTTAAAGAAGAAAAATTAGAATCACCA

GTTGTCTTCGCTCCCGCTAACATCACCACTGAAGTCAAG
TCCGTTGAAATGCACCACGAAGCTCTCGTTGAAGCCGTCCCCGGTGACAACGTTGGTTTC
AACGTAAAGAACGTCTCCGTCAAGGAATTGCGTCGTGGTTACGTCGCTGGTGACTCCAAG
GCTAACCCCCCCAAGGGCGCCGCTGACTTCACCGCTCAAGTCATCGTCTTGAACCACCCT
GGTCAAATCTCCAACGGTTACACCCCCGTTTTGGATTGTCACACCGCTCACATTGCTTGC
AAATTCGCCGAAATCAAGGAGAAGGTCGAT

>ITMOII9Ls
AGAATTCTAATTCGAGCTGAATTAGGACATCCT
GGAGCTTTAATTGGAGATGATCAAATTTATAATGTAATTGTTACAGCTCATGCTTTTATT
ATAATTTTTTTTATAGTAATGCCAATTATAATTGGAGGATTTGGAAATTGATTAGTTCCA
TTAATACTAGGAGCTCCAGATATAGCATTCCCTCGAATAAATAATATAAGTTTTTGACTT
TTACCTCCTGCATTAACTTTATTATTAGTTAGTAGTATAGTAGAAAACGGAGCTGGAACA
GGATGAACAGTTTACCCTCCTCTATCTTCTAATATTGCTCATGGAGGAGCTTCTGTTGAT
TTAGCTATTTTCTCTCTTCATTTAGCAGGAATTTCTTCAATTTTAGGAGCTGTAAATTTT
ATTACTACAGTTATTAATATACGATCAACAGGAATTACTTTTGATCGAATACCTTTATTT
GTTTGATCAGTAGTAATTACAGCTTTATTACTTTTATTATCATTACCAGTATTAGCAGGA
GCTATTACAATACTTTTAACAGACCGAAATCTTAATACATCATTCTTTGACCCTGCAGGA
GGAGGAGATCCAATTTTATACCAACATTTATTTAATAATTAATAATTCCATAAGTAGAAATATAAGGTATAAACCA
CATAGAACCTAAAAAATAAGAAAAATTATAATTATTTAAAGCCTTATTATAAAAAAATAA
AGAAACATTAGAAATTATATAACCTATAATACCCCCAACAATACAAACAAATAATGTTAA
TAATTTTAAATAAGAAGGAAGAACAACTACTATAGGAGTAGGAAAAATCAATCATCTTAA
CATTCTCCCACCAAAAATTCTTAAAATTAATAAACCTATTATACTTTTCAATATTACTCA
ACCTTCATCATTTAATATATTTAAAGAAGAAAAATTAGAATCACCA

GTTGTCTTCGCTCCCGCTAACATCACCACTGAAGTCAAGTCCGTTGAAATGC
ACCACGAAGCTCTCGTTGAAGCCGTCCCCGGTGACAACGTTGGTTTCAACGTAAAGAACG
TCTCCGTCAAGGAATTGCGTCGTGGTTACGTCGCTGGTGACTCCAAGGCCAACCCCCCCA
AGGGCGCCGCTGACTTCACCGCTCAAGTCATCGTCTTGAACCACCCTGGTCAAATCTCCA
ACGGTTATACCCCCGTTTTGGATTGTCACACCGCTCACATTGCTTGCAAATTCGCCGAAA
TCAAGGAGAAGGTCGAT

>ITTVLs1

AGAATTCTAATTCGAGCTGAATTAGGACAT
CCTGGAGCTTTAATTGGAGATGATCAAATTTATAATGTAATTGTTACAGCTCATGCTTTT
ATTATAATTTTTTTTATAGTAATGCCAATTATAATTGGAGGATTTGGAAATTGATTAGTT
CCATTAATACTAGGAGCTCCAGATATAGCATTCCCTCGAATAAATAATATAAGTTTTTGA
CTTTTACCTCCTGCATTAACTTTATTATTAGTTAGTAGTATAGTAGAAAACGGAGCTGGA
ACAGGATGAACAGTTTACCCTCCTCTATCTTCTAATATTGCTCATGGAGGAGCTTCTGTT
GATTTAGCTATTTTCTCTCTTCATTTAGCAGGAATTTCTTCAATTTTAGGAGCTGTAAAT
TTTATTACTACAGTTATTAATATACGATCAACAGGAATTACTTTTGATCGAATACCTTTA
TTTGTTTGATCAGTAGTAATTACAGCTTTATTACTTTTATTATCATTACCAGTATTAGCA
GGAGCTATTACAATACTTTTAACAGACCGAAATCTTAATACATCATTCTTTGACCCTGCA
GGAGGAGGAGATCCAATTTTATACCAACATTTATTTAATAATTAATAATTCCATAAGTAGAAATATAAGAGTATAAACCACATAGA

ACCTAAAAAATAAGAAAAATTATAATTATTTAAAGCCTTATTATAAAAAAATAAAGAAAC

ATTAGAAATTATATAACCTATAATACCCCCAACAATACAAACAAATAATGTTAATAATTT

TAAATAAGAAGGAAGAACAACTACTATAGGAGTAGGAAAAATCAATCATCTTAACATTCT

CCCACCAAAAATTCTTAAAATTAATAAACCTATTATACTTTTCAATATTACTCAACCTTC

ATCATTTAATATATTTAAAGAAGAAAAATTAGAATCACCAGTTGTCTTCGCTCCCGCTAACATCACCACTGAAGTCAAG

TCCGTTGAAATGCACCACGAAGCTCTCGTTGAAGCCGTCCCCGGTGACAACGTTGGTTTC

AACGTAAAGAACGTCTCCGTCAAGGAATTGCGTCGTGGTTACGTCGCTGGTGACTCCAAG

GCTAACCCCCCCAAGGGCGCCGCTGACTTCACCGCTCAAGTCATCGTCTTGAACCACCCT

GGTCAAATCTCCAACGGTTACACCCCCGTTTTGGATTGTCACACCGCTCACATTGCTTGC

AAATTCGCCGAAATCAAGGAGAAGGTCGAT

>UKCvo

AGAATTTTAATTCGAGCTGAACTAGGGCATC
CTGGAGCATTAATTGGAGATGACCAAATTTATAATGTAATTGTTACAGCTCATGCTTTTA
TTATAATTTTTTTTATAGTAATACCAATTATAATTGGAGGGTTTGGAAATTGATTAGTTC
CTTTAATATTAGGAGCCCCAGATATAGCATTCCCTCGAATAAATAATATAAGTTTCTGAC
TTTTACCTCCTGCATTAACTTTATTATTAGTAAGTAGTATAGTAGAAAACGGAGCTGGAA
CTGGATGAACTGTTTATCCACCTTTATCTTCTAATATTGCACATGGAGGAGCTTCTGTTG
ATTTAGCTATTTTTTCTTTACATTTAGCAGGAATTTCTTCAATTTTAGGAGCTGTAAATT
TTATTACTACAGTTATTAATATACGATCAACAGGTATTACCTTCGACCGAATACCATTAT
TTGTTTGATCAGTAGTAATTACAGCCTTATTACTTTTATTATCTTTACCAGTATTAGCAG
GAGCTATTACTATATTATTAACAGATCGAAATCTTAATACTTCATTCTTTGACCCAGCAG
GAGGAGGAGATCCAATTTTATACCAACACTTATTTAATAATTAATAATTCCGTATGTAGAAATATAAGGCATAAACCACAT

TGACCCTAAAAAATAAGAAGAATTATAGTTATTTAAAGCCTTATTATAAAAAAATAAAGA

AATATTAGAAATTAAATACCCTCTAATTCCTCCTACAATACAAACAAATAAAGTTAATAA

CTTTAAATAAGAAGGTAGAACTACTACTACTGGTGTAGGAAAAATTAATCAACTTAACAT

TCTACCCCCAAAAATTCTTAAAATTAATAACCCTATTATACTCTTTAACATAACTCAACC

TTCATCATTTAATATATTTAAAGAAGAAAAATTTGAATCCCCAGTTGTCTTCGCTCCCGCTAACATCACCACTGAAGTCAAATCCGTAGAAATGCACCACGAAGCTCTCGTT

GAAGCCGTCCCCGGTGACAACGTTGGTTTCAACGTTAAGAACGTCTCCGTCAAGGAATTG

CGTCGTGGTTACGTCGCTGGTGACTCCAAGGCTAGCCCCCCCAAGGGTGCTGCTGACTTC

ACCGCTCAAGTCATCGTCTTGAACCACCCTGGTCAAATCTCCAACGGTTACACTCCCGTT

TTGGATTGTCACACCGCTCACATTGCTTGCAAATTCGCCGAAATCAAGGAGAAGGTCGAT

>ITTVLs2

AGAATTCTAATTCGAGCTGAATTAGGACATC
CTGGAGCTTTAATTGGAGATGATCAAATTTATAATGTAATTGTTACAGCTCATGCTTTTA
TTATAATTTTTTTTATGGTAATGCCAATTATAATTGGAGGATTTGGAAATTGATTAGTTC
CATTAATACTAGGAGCTCCAGATATAGCATTCCCTCGAATAAATAATATAAGTTTTTGAC
TTTTACCTCCTGCATTAACTTTATTATTAGTTAGTAGTATAGTAGAAAACGGAGCTGGAA
CAGGATGAACAGTTTACCCTCCTCTATCTTCTAATATTGCTCATGGAGGAGCTTCTGTTG
ATTTAGCTATTTTCTCTCTTCATTTAGCAGGAATTTCTTCAATTTTAGGAGCTGTAAATT
TTATTACTACAGTTATTAATATACGATCAACAGGAATTACTTTTGATCGAATACCTTTAT
TTGTTTGATCAGTAGTAATTACAGCTTTATTACTTTTATTATCATTACCAGTATTAGCAG
GAGCTATTACAATACTTTTAACAGACCGAAATCTTAATACATCATTCTTTGACCCTGCAG
GAGGAGGAGATCCAATTTTATACCAACATTTATTTAATAATTAATAATTCCATAAGTAGAAATATAAGAGTATAAACCACATAGAACC

TAAAAAATAAGAAAAATTATAATTATTTAAAGCCTTATTATAAAAAAATAAAGAAACATT

AGAAATTATATAACCTATAATACCCCCAACAATACAAACAAATAATGTTAATAATTTTAA

ATAAGAAGGAAGAACAACTACTATAGGAGTAGGAAAAATCAATCATCTTAACATTCTCCC

ACCAAAAATTCTTAAAATTAATAAACCTATTATACTTTTCAATATTACTCAACCTTCATC

ATTTAATATATTTAAAGAAGAAAAATTAGAATCACCAGTTGTCTTCGCTCCCGCTAACATCACCACTGAAG

TCAAGTCCGTTGAAATGCACCACGAAGCTCTCGTTGAAGCCGTCCCCGGTGACAACGTTG

GTTTCAACGTAAAGAACGTCTCCGTTAAGGAATTGCGTCGTGGTTACGTCGCTGGTGACT

CCAAGGCCAACCCCCCCAAGGGCGCCGCTGACTTCACCGCTCAAGTCATCGTCTTGAACC

ACCCTGGTCAAATCTCCAACGGTTACACCCCCGTTTTGGATTGTCACACCGCTCACATTG

CTTGCAAATTCGCCGAAATCAAGGAGAAGGTCGAT

>ITTVLs3

AGAATTCTAATTCGAGCTGAATTAGGACATC
CTGGAGCTTTAATTGGAGATGATCAAATTTATAATGTAATTGTTACAGCTCATGCTTTTA
TTATAATTTTTTTTATAGTAATGCCAATTATAATTGGAGGATTTGGAAATTGATTAGTTC
CATTAATACTAGGAGCTCCAGATATAGCATTCCCTCGAATAAATAATATAAGTTTTTGAC
TTTTACCTCCTGCATTAACTTTATTATTAGTTAGTAGTATAGTAGAAAACGGAGCTGGAA
CAGGATGAACAGTTTACCCTCCTCTATCTTCTAATATTGCTCATGGAGGAGCTTCTGTTG
ATTTAGCTATTTTCTCTCTTCATTTAGCAGGAATTTCTTCAATTTTAGGAGCTGTAAATT
TTATTACTACAGTTATTAATATACGATCAACAGGAATTACTTTTGATCGAATACCTTTAT
TTGTTTGATCAGTAGTAATTACAGCTTTATTACTTTTATTATCATTACCAGTATTAGCAG
GAGCTATTACAATACTTTTAACAGACCGAAATCTTAATACATCATTCTTTGACCCTGCAG
GAGGAGGAGATCCAATTTTATACCAACATTTATTTAATAATTAATAATTCCATAAGTAGAAATATAAGAGTATAAACCACATAGAACC

TAAAAAATAAGAAAAATTATAATTATTTAAAGCCTTATTATAAAAAAATAAAGAAACATT

AGAAATTATATAACCTATAATACCCCCAACAATACAAACAAATAATGTTAATAATTTTAA

ATAAGAAGGAAGAACAACTACTATAGGAGTAGGAAAAATCAATCATCTTAACATTCTCCC

ACCAAAAATTCTTAAAATTAATAAACCTATTATACTTTTTAATATTACTCAACCTTCATC

ATTTAATATATTTAAAGAAGAAAAATTAGAATCACCAGTTGTCTTCGCTCCCGCTAACATCACCACTGAAGTCAAG

TCCGTTGAAATGCACCACGAAGCTCTCGTTGAAGCCGTCCCCGGTGACAACGTTGGTTTC

AACGTAAAGAACGTCTCCGTCAAGGAATTGCGTCGTGGTTACGTCGCTGGTGACTCCAAG

GCCAACCCCCCCAAGGGCGCCGCTGACTTCACCGCTCAAGTCATCGTCTTGAACCACCCT

GGTCAAATCTCCAACGGTTACACCCCCGTTTTGGATTGTCACACCGCTCACATTGCTTGC

AAATTCGCCGAAATCAAGGAGAAGGTCGAT

>ITTVLc1

AGAATTTTAATTCGAGCTGAATTAGGACACCCTGGTG
CATTAATTGGAGATGACCAAATTTATAATGTAATTGTTACAGCTCATGCTTTTATTATAA
TTTTCTTTATAGTAATACCAATTATAATTGGAGGATTTGGAAATTGATTAGTTCCTTTAA
TATTAGGAGCCCCAGATATGGCATTCCCTCGAATAAATAATATAAGATTTTGACTTTTAC
CTCCTGCATTAACTTTATTATTAGTAAGTAGTATAGTAGAAAACGGAGCTGGAACAGGAT
GAACAGTTTACCCTCCTTTATCATCTAATATCGCTCATGGAGGAGCTTCTGTTGATTTAG
CTATTTTTTCTCTTCATTTAGCAGGAATCTCATCAATTTTAGGGGCTGTAAATTTTATTA
CTACAGTTATTAATATACGATCAACAGGAATTACTTTTGACCGAATACCTTTATTCGTTT
GATCAGTAGTAATTACAGCTTTATTACTTTTATTATCATTACCAGTATTAGCTGGAGCTA
TTACTATACTTTTAACTGACCGAAATCTTAATACTTCATTCTTTGACCCAGCAGGAGGAG
GAGACCCAATTTTATACCAACATTTATTTAATAATTAATAATTCCGTAAGTAGAAATATAAGGTATAAACCACATT

GACCCTAAAAAATAAGAAAAATTATAATTATTTAAAGCCTTATTATAAAAAAATAAAGAA

ATATTTGAAATTATATACCCTCTTACTCCTCCTACAATACAAACAAATAATGTTAATAAC

TTCAAATATGAAGGAAGAACTACAACTACTGGAGTTGGAAAAATTAATCATCTTAATATT

CTTCCCCCAAAAATTCTTAAAATTAATAACCCTATTATACTTTTTAATATTACTCAACCT

TCATCATTTAATATATTTAAAGAAGAAAAATTTGAATCACCA
GTTGTCATCGCTCCCGCTAACATCACCACTGAAGTCAAGTCCGTTGAAAT

GCACCACGAAGCTCTCGTTGAAGCCGTCCCCGGTGACAACGTTGGTTTCAACGTAAAGAA

CGTCTCCGTCAAGGAATTGCGTCGTGGTTACGTCGCTGGTGACTCCAAGGCCAACCCCCC

CAAGGGTGCCGCTGACTTCACCGCTCAAGTCATCGTCTTGAACCATCCCGGTCAAATCTC

CAACGGTTACACTCCCGTTTTGGATTGTCACACCGCTCACATTGCTTGCAAATTCGCCGA

AATCAAGGAGAAGGTCGAT

>ITVVChalbA

AGAATTCTAATTCGAGCTGAATTAGGACA
TCCTGGAGCACTAATTGGAGATGACCAAATTTATAATGTAATTGTAACAGCTCATGCCTT
TATTATAATTTTCTTTATAGTAATACCAATTATAATTGGAGGATTTGGAAATTGACTAGT
TCCTTTAATATTAGGAGCCCCAGATATAGCTTTCCCACGAATAAATAATATAAGTTTCTG
ACTTTTACCTCCTGCATTAACTTTACTATTAGTAAGTAGTATAGTAGAAAATGGAGCTGG
AACAGGATGAACTGTTTATCCACCTTTATCATCTAATATTGCTCATGGTGGAGCATCAGT
TGATTTAGCTATTTTTTCTTTACACTTAGCTGGAATTTCATCAATTTTAGGAGCTGTAAA
TTTTATTACAACTGTTATTAATATACGATCTACAGGAATCACATTTGATCGAATACCTTT
ATTCGTATGATCTGTAGTTATTACTGCTCTTCTTTTATTATTATCATTACCAGTATTAGC
CGGTGCAATTACTATATTATTAACTGATCGAAATTTAAATACTTCATTCTTTGATCCAGC
AGGAGGAGGAGATCCTATTTTATATCAACATTTATTT AATAATTAATAATACCATATGTAGAAATATATGGTATAAATCA

TATTGAACCTAAAAAATAAGAAGAATTATAATTATTTAATGCCTTATTATAAAAAAATAA

TGAAACATTAGAAATTATATAACCACTTACTCCTCCTACAATACAAACAAATATAGTTAA

CAACTTTAAATATATTGGAAGAACTACTAACACTGGAGTAGGAAAAATTAATCATCTTAA

TATTCTCCCTCCAAAAATTCTTAGAATCAATAAACCTATTATACTTTTTAATATTACTCA

ACCTTCATCATTTAATATATTTAAAGATGAAAAATTTGAATCTCCAGTTGTCTTCGCTCCCGCTAACATCACCACTGAAGTTAAG

TCCGTTGAAATGCACCACGAAGCTCTCGCTGAAGCCGTACCCGGTGACAACGTTGGTTTC

AACGTAAAGAACGTCTCCGTCAAGGAATTGCGTCGTGGTTACGTTGCTGGTGACTCCAAG

GCTAACCCACCCAAGGGTGCTGCTGACTTCACCGCTCAAGTCATTGTCTTGAACCACCCT

GGTCAAATCTCCAACGGTTACACCCCCGTTTTGGATTGTCACACCGCTCACATTGCTTGC

AAATTCGCTGAAATCAAGGAGAAGGTCGAT

>ITTVCvo3

AGAATTTTAATTCGAGCTGAACTAGGGCAT
CCTGGAGCATTAATTGGAGATGATCAAATTTATAATGTAATTGTTACAGCTCATGCTTTT
ATTATAATTTTTTTTATAGTAATACCAATTATAATTGGAGGGTTTGGAAATTGATTAGTT
CCTTTAATATTAGGAGCCCCAGATATAGCATTCCCTCGAATAAATAATATAAGTTTCTGA
CTTTTACCTCCTGCATTAACTTTACTATTAGTAAGTAGTATAGTAGAAAACGGAGCTGGA
ACTGGATGAACTGTTTATCCACCTTTATCTTCTAATATTGCACATGGAGGAGCTTCTGTT
GATTTAGCTATTTTTTCTTTACATTTAGCAGGAATTTCTTCAATTTTAGGAGCTGTAAAT
TTTATTACTACAGTTATTAATATACGATCAACAGGTATTACCTTCGACCGAATACCATTA
TTTGTTTGATCAGTAGTAATTACAGCCTTATTACTTTTATTATCTTTACCAGTATTAGCA
GGAGCTATTACTATATTATTAACAGATCGAAATCTTAATACTTCATTCTTTGACCCAGCA
GGAGGAGGAGATCCAATTTTATACCAACACTTATTTAGTAATTAATAATTCCGTATGTAGAAATATAAGGCATAAACCACATT

GACCCTAAAAAATAAGAAGAATTATAGTTATTTAAAGCCTTATTATAAAAAAATAAAGAA

ATATTAGAAATTAAATACCCTCTAATTCCTCCTACAATACAAACAAATAAAGTTAATAAC

TTTAAATAAGAAGGTAGAACTACTACTACTGGTGTAGGAAAAATTAATCAACTTAACATT

CTACCCCCAAAAATTCTTAAAATTAATAACCCTATTATACTCTTTAACATAACTCAACCT

TCATCATTTAATATATTTAAAGAAGAAAAATTTGAATCCCCAGTTGTCTTCGCTCCCGCTAACATCACCACTGAAGTCAAATCCGTAG

AAATGCACCACGAAGCTCTCGTTGAAGCCGTCCCCGGTGACAACGTTGGTTTCAACGTTA

AGAACGTCTCCGTCAAGGAATTGCGTCGTGGTTACGTCGCTGGTGACTCCAAGGCTAGCC

CCCCCAAGGGTGCTGCTGACTTCACCGCTCAAGTCATCGTCTTGAACCACCCTGGTCAAA

TCTCCAACGGTTACACTCCCGTTTTGGATTGTCACACCGCTCACATTGCTTGCAAATTCG

CCGAAATCAAGGAGAAGGTCGAT

>ITTVCvi3

AGAATTCTAATTCGAGCCGAACTAGGAC
ATCCTGGAGCATTAATTGGAGATGACCAAATTTATAATGTAATTGTTACAGCTCATGCTT
TTATTATAATTTTTTTTATAGTAATACCAATTATAATTGGAGGATTTGGTAATTGATTAG
TCCCTTTAATATTAGGAGCTCCAGATATAGCCTTCCCTCGGATAAACAATATAAGTTTCT
GACTTTTACCTCCTGCATTAACTTTACTATTAGTAAGTAGTATAGTAGAAAACGGAGCTG
GAACAGGATGAACTGTTTACCCACCTTTATCTTCTAATATCGCTCATGGAGGAGCTTCTG
TTGATTTAGCTATTTTTTCTTTACACTTAGCAGGAATTTCTTCAATTTTAGGGGCTGTAA
ATTTTATTACTACAGTTATTAATATACGATCAACAGGAATTACATTCGACCGAATACCAT
TATTTGTTTGATCTGTAGTAATTACAGCTTTATTACTTTTATTATCTTTACCAGTATTAG
CAGGTGCTATTACTATATTATTAACAGATCGAAATCTTAATACATCATTCTTTGACCCAG
CAGGAGGAGGAGACCCAATCTTGTACCAACATTTATTTAATAATTAATAATTCCATATGTAGAAATATAAGGTATAAATCATATTGAA

CCTAAAAAGTAAGAAGAATTATAATTATTTAAAGCCTTATTATAAAAAAATAAAGAAATA

TTAGAAATTAAATAACCTCTAACTCCTCCTACAATACAAACAAATAAAGTTAATAATTTT

AAATAAGAAGGAAGAACTACTACTACTGGTGTAGGAAAAATTAATCAACTTAATATACTA

CCCCCAAAAATTCTCAAAATTAATAGTCCTATTATACTTTTTAATATAACTCAACCTTCA

TCATTTAGTATATTTAAAGAAGAAAAATTTGAATCCCCAGTTGTCTTCGCTCCCGCTAACATCACCACTGAAGTCAAGT

CCGTAGAAATGCACCACGAAGCTCTCGTTGAAGCTGTTCCCGGTGACAACGTTGGTTTCA

ACGTTAAGAACGTCTCCGTCAAGGAATTGCGTCGTGGTTACGTCGCTGGTGACTCCAAGG

CTAGCCCCCCCAAGGGTGCTGCTGACTTCACCGCTCAAGTCATCGTCTTGAACCACCCTG

GTCAAATCTCCAACGGTTACACTCCCGTTTTGGATTGTCACACCGCTCACATTGCTTGCA

AATTCGCCGAAATCAAGGAGAAGGTCGAT

>ITMOCvi4

AGAATTCTAATTCGAGCCGAACTAGGACA
TCCTGGAGCATTAATTGGAGATGACCAAATTTATAATGTAATTGTTACAGCTCATGCTTT
TATTATAATTTTTTTTATAGTAATACCAATTATAATTGGAGGATTTGGTAATTGATTAGT
CCCTTTAATATTAGGAGCTCCAGATATAGCCTTCCCTCGGATAAACAATATAAGTTTCTG
ACTTTTACCTCCTGCATTAACTTTACTATTAGTAAGTAGTATAGTAGAAAACGGAGCTGG
AACAGGATGAACTGTTTACCCACCTTTATCTTCTAATATCGCTCATGGAGGAGCTTCTGT
TGATTTAGCTATTTTTTCTTTACACTTAGCAGGAATTTCTTCAATTTTAGGAGCTGTAAA
TTTTATTACTACAGTTATTAATATACGATCAACAGGAATTACATTCGACCGAATACCATT
ATTTGTTTGATCTGTAGTAATTACAGCTTTATTACTTTTATTATCTTTACCAGTATTAGC
AGGTGCTATTACTATATTATTAACAGATCGAAATCTTAATACATCATTCTTTGACCCAGC
AGGAGGAGGAGACCCAATCTTGTACCAACATTTATTTAATAATTAATAATTCCATATGTAGAAATATAAGGTATAAATCATATT

GAACCTAAAAAGTAAGAAGAATTATAATTATTTAAAGCCTTATTATAAAAAAATAAAGAA

ATATTAGAAATTAAATAACCTCTAACTCCTCCTACAATACAAACAAATAAAGTTAATAAT

TTTAAATAAGAAGGAAGAACTACTACTACTGGTGTAGGAAAAATTAATCAACTTAATATA

CTACCCCCAAAAATTCTCAAAATTAATAGTCCTATTATACTTTTTAATATAACTCAACCT

TCATCATTTAGTATATTTAAAGAAGAAAAATTTGAATCCCCAGTTGTCTTCGCTCCCGCTAACATCACCACTGAAGTCAAGTC

CGTAGAAATGCACCACGAAGCTCTCGTTGAAGCTGTTCCCGGTGACAACGTTGGTTTCAA

CGTTAAGAACGTCTCCGTCAAGGAATTGCGTCGTGGTTACGTCGCTGGTGACTCCAAGGC

TAGCCCCCCCAAGGGTGCTGCTGACTTCACCGCTCAAGTCATCGTCTTGAACCACCCTGG

TCAAATCTCCAACGGTTACACTCCCGTTTTGGATTGTCACACCGCTCACATTGCTTGCAA

ATTCGCCGAAATCAAGGAGAAGGTCGAT

>ITTVSA

AGAATTCTTATTCGAGCAGAATTAGGTCAC
CCTGGTGCATTAATTGGTGATGATCAAATTTATAATGTAATTGTTACAGCCCATGCTTTC
ATTATAATTTTTTTTATAGTAATACCAATTATAATTGGAGGATTTGGAAATTGATTAGTG
CCAATTATACTAGGAGCTCCAGATATAGCCTTCCCTCGGATAAACAATATAAGTTTTTGA
CTTTTACCTCCTGCATTAACATTGCTTCTAGTAAGTAGTATAGTAGAAAATGGAGCTGGA
ACAGGTTGAACTGTATACCCTCCTTTATCTTCTAATATTGCTCATGGAGGAGCTTCTGTT
GATTTAGCTATTTTTTCTCTCCATTTAGCTGGAATTTCTTCAATTCTAGGAGCAGTAAAT
TTTATTACTACAGTTATTAATATACGATCAACAGGAATCACTTTTGATCGAATACCTTTA
TTTGTATGATCTGTAGTAATCACAGCCCTACTTTTATTACTTTCTTTACCTGTACTTGCC
GGAGCTATTACTATATTATTAACTGATCGAAATATTAATACTTCATTTTTTGACCCGGCA
GGAGGAGGAGATCCTATTCTATATCAACATTTATTTAATAATTTATAATCCCATAAGTAGAAATATAAGGCATAAATCAT

ATTGAACCTAAAAAATAAGAACTATTATATATATTTAAAGCCTTATTAGAAAAAAATAAA

GAAATATTAGAAATTAAATAACCTATTAATCCTCCTACAATACACACAAATAAAGTTAAT

AACTTTAAATAATAAGGTAAAACAATTACCATAGGACTAGGAAAAATTAATCAACTTAAT

ATTCTACCCCCAAAAATTCTTAAAATTAATAATCCTATTATACTTTTTAATATAACTCAA

CCCTCATCATTTAATATATTTAAAGAAGAAAAATTCGAATCTCCA
GTTGTCTTCGCCCCCGCTAACATCACCACTGAAGT

CAAGTCCGTTGAAATGCACCACGAAGCTCTTGTTGAAGCCGTTCCCGGTGACAACGTTGG

TTTCAACGTTAAGAACGTCTCCGTCAAGGAATTGCGTCGTGGTTACGTCGCTGGTGACTC

CAAGGCTTCTCCCCCCAAGGGTGCTGCTGACTTCACCGCTCAAGTTATTGTGTTGAACCA

TCCTGGTCAAATCTCCAACGGTTATACCCCCGTCTTGGATTGTCACACCGCTCACATTGC

TTGCAAATTCGCTGAAATCAAGGAGAAGGTCGAT

>UKPio

AGTATCTTAATCCGAGCAGAGCTA
GGACATCCAGGAGCTTTAATTGGTGATGATCAAATTTATAATGTAATTGTCACCGCCCAT
GCTTTTGTCATAATTTTTTTTATAGTAATACCAATTATAATTGGGGGATTTGGAAATTGA
TTAGTTCCTTTAATACTAGGAGCTCCTGATATAGCTTTCCCACGAATAAATAATATAAGT
TTTTGAATATTACCTCCTTCTCTTACACTACTATTAGTAAGAAGTATAGTAGAAAACGGA
GCTGGTACAGGTTGAACAGTTTACCCTCCTCTTTCTTCTGTTATTGCTCATGGAGGGGCT
TCTGTTGATTTAGCTATTTTTTCCCTTCACCTAGCAGGAATTTCTTCAATTTTAGGAGCT
GTAAATTTTATCACAACTGTAATTAATATACGATCTACAGGTATCACTTTTGATCGAATA
CCTTTATTTGTTTGATCTGTTGTAATTACAGCTTTATTATTACTTTTATCTCTACCTGTT
CTAGCAGGAGCAATTACAATATTATTAACAGATCGAAATTTAAATACTTCATTCTTTGAC
CCAGCAGGAGGAGGAGACCCAATTCTTTACCAACATTTATTT GATAACTCACAATTCCGTAAGTCGAAATAAAAGGTATAAA

TCATATTGAGCCAAAATAATATCTTAAAACATATGTTATTAAAGACTTATTAACATAAAA

TAAAGAAACGTTAGAAATTAAATAACCTAAAATACCCCCTACGATACAAACAAATAATGT

TAATAACTTTAAATAATAAGGTAAACACACTATTAAAGGAGTAGGAAAAATTAATCAATT

TAATATTCTACCTCCAATAATTCTTATAAATAATAATCCTAGTATACCCTTTAATATAAT

TCATCCCTCATCATTAAGTATATTTAAAGACCCGCAATTTAAATCCCCA

GTTGTCTTTGCCCCAGCGAACATCACCACTGAAGTTAAGTCC

GTTGAAATGCATCACGAAGCTCTCATCGAGGCTGTTCCCGGTGACAATGTTGGCTTCAAC

GTCAAGAACGTGTCCGTCAAGGAATTGCGTCGTGGCTATGTTGCCGGTGACACCAAGAAC

AACCCACCCCGCGGTACTGCCGATTTCAACGCTCAGGTTATTGTATTGAACCATCCCGGT

CAAATTGCCAACGGTTACACTCCAGTCTTGGATTGCCACACCGCTCACATTGCCTGCAAA

TTCGCTGAGATCAAGGAGAAGGTCGAT

>ITMOCvi3

AGAATTCTAATTCGAGCCGAACTAGGACATCCTGGA
GCATTAATTGGAGATGACCAAATTTATAATGTAATTGTTACAGCTCATGCTTTTATTATA
ATTTTTTTTATAGTAATACCAATTATAATTGGAGGATTTGGTAATTGATTAGTCCCTTTA
ATATTAGGAGCTCCAGATATAGCCTTCCCTCGGATAAACAATATAAGTTTCTGACTTTTA
CCTCCTGCATTAACTTTACTATTAGTAAGTAGTATAGTAGAAAACGGAGCTGGAACAGGA
TGAACTGTTTACCCACCTTTATCTTCTAATATCGCTCATGGAGGAGCTTCTGTTGATTTA
GCTATTTTTTCTTTACACTTAGCAGGAATTTCTTCAATTTTAGGAGCTGTAAATTTTATT
ACTACAGTTATTAATATACGATCAACAGGAATTACATTCGACCGAATACCATTATTTGTT
TGATCTGTAGTAATTACAGCTTTATTACTTTTATTATCTTTACCAGTATTAGCAGGTGCT
ATTACTATATTATTAACAGATCGAAATCTTAATACATCATTCTTTGACCCAGCAGGAGGA
GGAGACCCAATCTTGTACCAACATTTATTTAATAATTAATAATTCCATATGTAGAAATATAAGGTATAAATCATATTGA

ACCTAAAAAGTAAGAAGAATTATAATTATTTAAAGCCTTATTATAAAAAAATAAAGAAAT

ATTAGAAATTAAATAACCTCTAATTCCTCCTACAATACAAACAAATAAAGTTAATAATTT

TAAATAAAAAGGAAGAACTACTACTACTGGTGTAGGAAAAATTAATCAACTTAATATACT

ACCCCCAAAAATTCTCAAAATTAATAGTCCTATTATACTTTTTAATATAACTCAACCTTC

ATCATTTAGTATATTTAAAGAAGAAAAATTTGAATCTCCAGTTGTCTTCGCTCCCGCTAACATCACCACTGAAGTCAAGTCCGTAGAAATGCACCACGAA

GCTCTCGTTGAAGCTGTTCCCGGTGACAACGTTGGTTTCAACGTTAAGAACGTCTCCGTC

AAGGAATTGCGTCGTGGTTACGTCGCTGGTGACTCCAAGGCTAGCCCCCCCAAGGGTGCT

GCTGACTTCACCGCTCAAGTCATCGTCTTGAACCACCCTGGTCAAATCTCCAACGGTTAC

ACTCCCGTTTTGGATTGTCACACCGCTCACATTGCTTGCAAATTCGCCGAAATCAAGGAG

AAGGTCGAT

>ITVVLs

AGAATTCTAATTCGAGCTGAATTAGGACATCCT
GGAGCTTTAATTGGAGATGATCAAATTTATAATGTAATTGTTACAGCTCATGCTTTTATT
ATAATTTTTTTTATAGTAATGCCAATTATAATTGGAGGATTTGGAAATTGATTAGTTCCA
TTAATACTAGGAGCTCCAGATATAGCATTCCCTCGAATAAATAATATAAGTTTTTGACTT
TTACCTCCTGCATTAACTTTATTATTAGTTAGTAGTATAGTAGAAAACGGAGCTGGAACA
GGATGAACAGTTTACCCTCCTCTATCTTCTAATATTGCTCATGGAGGAGCTTCTGTTGAT
TTAGCTATTTTCTCTCTTCATTTAGCAGGAATTTCTTCAATTTTAGGAGCTGTAAATTTT
ATTACTACAGTTATTAATATACGATCAACAGGAATTACTTTTGATCGAATACCTTTATTT
GTTTGATCAGTAGTAATTACAGCTTTATTACTTTTATTATCATTACCAGTATTAGCAGGA
GCTATTACAATACTTTTAACAGACCGAAATCTTAATACATCATTCTTTGACCCTGCAGGA
GGAGGAGATCCAATTTTATACCAACATTTATTTATAATTTAATAATTCCATAAGTAGAAATATAAGAGGTATAAACCACATAGAA

CCTAAAAAATAAGAAAAATTATAATTATTTAAAGCCTTATTATAAAAAAATAAAGAAACA

TTAGAAATTATATAACCTATAATACCCCCAACAATACAAACAAATAATGTTAATAATTTT

AAATAAGAAGGAAGAACAACTACTATAGGAGTAGGAAAAATCAATCATCTTAACATTCTC

CCACCAAAAATTCTTAAAATTAATAAACCTATTATACTTTTCAATATTACTCAACCTTCA

TCATTTAATATATTTAAAGAAGAAAAATTAGAATCACCAGTTGTCTTCGCTCCCGCTAACATCACCACTGAAGTCA

AGTCCGTTGAAATGCACCACGAAGCTCTCGTTGAAGCCGTCCCCGGTGACAACGTTGGTT

TCAACGTAAAGAACGTCTCCGTCAAGGAATTGCGTCGTGGTTACGTCGCTGGTGACTCCA

AGGCCAACCCCCCCAAGGGCGCCGCTGACTTCACCGCTCAAGTCATCGTCTTGAACCACC

CTGGTCAAATCTCCAACGGTTACACCCCCGTTTTGGATTGTCACACCGCTCACATTGCTT

GCAAATTCGCCGAAATCAAGGAGAAGGTCGAT

>1ITLs

AGAATTCTAATTCGAGCTGAATTAGGACATC
CTGGAGCTTTAATTGGAGACGATCAAATTTATAATGTAATTGTTACAGCTCATGCTTTTA
TTATAATTTTTTTTATAGTAATGCCAATTATAATTGGAGGATTTGGAAATTGATTAGTTC
CATTAATACTAGGAGCTCCAGATATAGCATTCCCTCGAATAAATAATATAAGTTTTTGAC
TTTTACCTCCTGCATTAACTTTATTATTAGTTAGTAGTATAGTAGAAAACGGAGCTGGAA
CAGGATGAACAGTTTACCCTCCTCTATCTTCTAATATTGCTCATGGAGGAGCTTCTGTTG
ATTTAGCTATTTTCTCTCTTCATTTAGCAGGAATTTCTTCAATTTTAGGAGCTGTAAATT
TTATTACTACAGTTATTAATATACGATCAACAGGAATTACTTTTGATCGAATACCTTTAT
TTGTTTGATCAGTAGTAATTACAGCTTTATTACTTTTATTATCATTACCAGTATTAGCAG
GAGCTATTACAATACTTTTAACAGACCGAAATCTTAATACATCATTCTTTGACCCTGCAG
GAGGAGGAGATCCAATTTTATACCAACATTTATTTAATAATTAATAATTCCATAAGTAGAAATATAAGAGTATAAACCACATAGAAC

CTAAAAAATAAGAAAAATTATAATTATTTAAAGCCTTATTATAAAAAAATAAAGAAACAT

TAGAAATTATATAACCTATAATACCCCCAACAATACAAACAAATAATGTTAATAATTTTA

AATAAGAAGGAAGAACAACTACTATAGGAGTAGGAAAAATCAATCATCTTAACATTCTCC

CACCAAAAATTCTTAAAATTAATAAACCTATTATACTTTTCAATATTACTCAACCTTCAT

CATTTAATATATTTAAAGAAGAAAAATTAGAATCACCAGTTGTCTTCGCTCCCGCTAACATCACCACTGAAGTCAAGT

CCGTTGAAATGCACCACGAAGCTCTCGTTGAAGCCGTCCCCGGTGACAACGTTGGTTTCA

ACGTAAAGAACGTCTCCGTTAAGGAATTGCGTCGTGGTTACGTCGCTGGTGACTCCAAGG

CCAACCCCCCCAAGGGCGCCGCTGACTTCACCGCTCAAGTCATCGTCTTGAACCACCCTG

GTCAAATCTCCAACGGTTACACCCCCGTTTTGGATTGTCACACCGCTCACATTGCTTGCA

AATTCGCCGAAATCAAGGAGAAGGTCGAT

>2ITLs

AGAATTCTAATTCGAGCTGAATTAGGACATCCT
GGAGCTTTAATTGGAGATGATCAAATTTATAATGTAATTGTTACAGCTCATGCTTTTATT
ATAATTTTTTTTATAGTAATGCCAATTATAATTGGAGGATTTGGAAATTGATTAGTTCCA
TTAATACTAGGAGCTCCAGATATAGCATTCCCTCGAATAAATAATATAAGTTTTTGACTT
TTACCTCCTGCATTAACTTTATTATTAGTTAGTAGTATAGTAGAAAACGGAGCTGGAACA
GGATGAACAGTTTACCCTCCTCTATCTTCTAATATTGCTCATGGAGGAGCTTCTGTTGAT
TTAGCTATTTTCTCTCTTCATTTAGCAGGAATTTCTTCAATTTTAGGAGCTGTAAATTTT
ATTACTACAGTTATTAATATACGATCAACAGGAATTACTTTTGATCGAATACCTTTATTT
GTTTGATCAGTAGTAATTACAGCTTTATTACTTTTATTATCATTACCAGTATTAGCAGGA
GCTATTACAATACTTTTAACAGACCGAAATCTTAATACATCATTCTTTGACCCTGCAGGA
GGAGGAGATCCAATTTTATACCAACATTTATTTAATAATTAATAATTCCATAAGTAGAAATATAAGGTATAAACCAC

ATAGAACCTAAAAAATAAGAAAAATTATAATTATTTAAAGCCTTATTATAAAAAAATAAA

GAAACATTAGAAATTATATAACCTATAATACCCCCAACAATACAAACAAATAATGTTAAT

AATTTTAAATAAGAAGGAAGAACAACTACTATAGGAGTAGGAAAAATCAATCATCTTAAC

ATTCTCCCACCAAAAATTCTTAAAATTAATAAACCTATTATACTTTTCAATATTACTCAA

CCTTCATCATTTAATATATTTAAAGAAGAAAAATTAGAATCACCA
GTTGTCTTCGCTCCCGCTAACATCACCAC

TGAAGTCAAGTCCGTTGAAATGCACCACGAAGCTCTCGTTGAAGCCGTCCCCGGTGACAA

CGTTGGTTTCAACGTAAAGAACGTCTCCGTCAAGGAATTGCGTCGTGGTTACGTCGCTGG

TGACTCCAAGGCCAACCCCCCCAAGGGCGCCGCTGACTTCACCGCTCAAGTCATCGTCTT

GAACCACCCTGGTCAAATCTCCAACGGTTACACCCCCGTTTTGGATTGTCACACCGCTCA

CATTGCTTGCAAATTCGCCGAAATCAAGGAGAAGGTCGAT

>3ITLs

AGAATTCTAATTCGAGCTGAATTAGGACATCCTGG
AGCTTTAATTGGAGATGATCAAATTTATAATGTAATTGTTACAGCTCATGCTTTTATTAT
AATTTTTTTTATAGTAATGCCAATTATAATTGGAGGATTTGGAAATTGATTAGTTCCATT
AATACTAGGAGCTCCAGATATAGCATTCCCTCGAATAAATAATATAAGTTTTTGACTTTT
ACCTCCTGCATTAACTTTATTATTAGTTAGTAGTATAGTAGAAAACGGAGCTGGAACAGG
ATGAACAGTTTACCCTCCTCTATCTTCTAATATTGCTCATGGAGGAGCTTCTGTTGATTT
AGCTATTTTCTCTCTTCATTTAGCAGGAATTTCTTCAATTTTAGGAGCTGTAAATTTTAT
TACTACAGTTATTAATATACGATCAACAGGAATTACTTTTGATCGAATACCTTTATTTGT
TTGATCAGTAGTAATTACAGCTTTATTACTTTTATTATCATTACCAGTATTAGCAGGAGC
TATTACAATACTTTTAACAGACCGAAATCTTAATACATCATTCTTTGACCCTGCAGGAGG
AGGAGATCCAATTTTATACCAACATTTATTT AATAATTAATAATTCCATAAGTAGAAATATAAGGTATAAACCACATAGAAC

CTAAAAAATAAGAAAAATTATAATTATTTAAAGCCTTATTATAAAAAAATAAAGAAACAT

TAGAAATTATATAACCTATAATACCCCCAACAATACAAACAAATAATGTTAATAATTTTA

AATAAGAAGGAAGAACAACTACTATAGGAGTAGGAAAAATCAATCATCTTAACATTCTCC

CACCAAAAATTCTTAAAATTAATAAACCTATTATACTTTTCAATATTACTCAACCTTCAT

CATTTAATATATTTAAAGAAGAAAAATTAGAATCACCA

GTTGTCTTCGCTCCCGCTAACATCACCACTGAAG

TCAAGTCCGTTGAAATGCACCACGAAGCTCTCGTTGAAGCCGTCCCCGGTGACAACGTTG

GTTTCAACGTAAAGAACGTCTCCGTCAAGGAATTGCGTCGTGGTTACGTCGCTGGTGACT

CCAAGGCCAACCCCCCCAAGGGCGCCGCTGACTTCACCGCTCAAGTCATCGTCTTGAACC

ACCCTGGTCAAATCTCCAACGGTTACACCCCCGTTTTGGATTGTCACACCGCTCACATTG

CTTGCAAATTCGCCGAAATCAAGGAGAAGGTCGAT

>4ITLs

AGAATTCTAATTCGAGCTGAATTAGGACATCC
TGGAGCTTTAATTGGAGATGATCAAATTTATAATGTAATTGTTACAGCTCATGCTTTTAT
TATAATTTTTTTTATAGTAATGCCAATTATAATTGGAGGATTTGGAAATTGATTAGTTCC
ATTAATACTAGGAGCTCCAGATATAGCATTCCCTCGAATAAATAATATAAGTTTTTGACT
TTTACCTCCTGCATTAACTTTATTATTAGTTAGTAGTATAGTAGAAAACGGAGCTGGAAC
AGGATGAACAGTTTACCCTCCTCTATCTTCTAATATTGCTCATGGAGGAGCTTCTGTTGA
TTTAGCTATTTTCTCTCTTCATTTAGCAGGAATTTCTTCAATTTTAGGAGCTGTAAATTT
TATTACTACAGTTATTAATATACGATCAACAGGAATTACTTTTGATCGAATACCTTTATT
TGTTTGATCAGTAGTAATTACAGCTTTATTACTTTTATTATCATTACCAGTATTAGCAGG
AGCTATTACAATACTTTTAACAGACCGAAATCTTAATACATCATTCTTTGACCCTGCAGG
AGGAGGAGATCCAATTTTATACCAACATTTATTTAATAATTAATAATTCCATAAGTAGAAATATAAGAGTATAAACCA

CATAGAACCTAAAAAATAAGAAAAATTATAATTATTTAAAGCCTTATTATAAAAAAATAA

AGAAACATTAGAAATTATATAACCTATAATACCCCCAACAATACAAACAAATAATGTTAA

TAATTTTAAATAAGAAGGAAGAACAACTACTATAGGGGTAGGAAAAATCAATCATCTTAA

CATTCTCCCACCAAAAATTCTTAAAATTAATAAACCTATTATACTTTTCAATATTACTCA

ACCTTCATCATTTAATATATTTAAAGAAGAAAAATTAGAATCACCA

GTTGTTTTCGCTCCCGCTAACATCACCACT

GAAGTCAAGTCCGTTGAAATGCACCACGAAGCTCTCGTTGAAGCCGTCCCCGGTGACAAC

GTTGGTTTCAACGTAAAGAACGTCTCCGTCAAGGAATTGCGTCGTGGTTACGTCGCTGGT

GACTCCAAGGCTAACCCCCCCAAGGGCGCCGCTGACTTCACCGCTCAAGTCATCGTCTTG

AACCACCCTGGTCAAATCTCCAACGGTTACACCCCCGTTTTGGATTGTCACACCGCTCAC

ATTGCTTGCAAATTCGCCGAAATCAAGGAGAAGGTCGAT

>5ITLs

AGAATTCTAATTCGAGCTGAATTAGGACATCCTGGAGC
TTTAATTGGAGATGATCAAATTTATAATGTAATTGTTACAGCTCATGCTTTTATTATAAT
TTTTTTTATAGTAATGCCAATTATAATTGGAGGATTTGGAAATTGATTAGTTCCATTAAT
ACTAGGAGCTCCAGATATAGCATTCCCTCGAATAAATAATATAAGTTTTTGACTTTTACC
TCCTGCATTAACTTTATTATTAGTTAGTAGTATAGTAGAAAACGGAGCTGGAACAGGATG
AACAGTTTACCCTCCTCTATCTTCTAATATTGCTCATGGAGGAGCTTCTGTTGATTTAGC
TATTTTCTCTCTTCATTTAGCAGGAATTTCTTCAATTTTAGGAGCTGTAAATTTTATTAC
TACAGTTATTAATATACGATCAACAGGAATTACTTTTGATCGAATACCTTTATTTGTTTG
ATCAGTAGTAATTACAGCTTTATTACTTTTATTATCATTACCAGTATTAGCAGGAGCTAT
TACAATACTTTTAACAGACCGAAATCTTAATACATCATTCTTTGACCCTGCAGGAGGAGG
AGATCCAATTTTATACCAACATTTATTTAATAATTAATAATTCCATAAGTAGAAATATAAGGTATAAACCACATAGAACC

TAAAAAATAAGAAAAATTATAATTATTTAAAGCCTTATTATAAAAAAATAAAGAAACATT

AGAAATTATATAACCTATAATACCCCCAACAATACAAACAAATAATGTTAATAATTTTAA

ATAAGAAGGAAGAACAACTACTATAGGAGTAGGAAAAATCAATCATCTTAACATTCTCCC

ACCAAAAATTCTTAAAATTAATAAACCTATTATACTTTTCAATATTACTCAACCTTCATC

ATTTAATATATTTAAAGAAGAAAAATTAGAATCACCA

GTTGTCTTCGCTCCCGCTAACATCACCACTGAAGTCAAGTCCGTTGA

AATGCACCACGAAGCTCTCGTTGAAGCCGTCCCCGGTGACAACGTTGGTTTCAACGTAAA

GAACGTCTCCGTCAAGGAATTGCGTCGTGGTTACGTCGCTGGTGACTCCAAGGCTAACCC

CCCCAAGGGCGCCGCTGACTTCACCGCTCAAGTCATCGTCTTGAACCACCCTGGTCAAAT

CTCCAACGGTTACACCCCCGTTTTGGATTGTCACACCGCTCACATTGCTTGCAAATTCGC

CGAAATCAAGGAGAAGGTCGAT

>6ITLs

AGAATTCTAATTCGAGCTGAATTAGGACATCCTGGAGC
TTTAATTGGAGATGATCAAATTTATAATGTAATTGTTACAGCTCATGCTTTTATTATAAT
TTTTTTTATAGTAATGCCAATTATAATTGGAGGATTTGGAAATTGATTAGTTCCATTAAT
ACTAGGAGCTCCAGATATAGCATTCCCTCGAATAAATAATATAAGTTTTTGACTTTTACC
TCCTGCATTAACTTTATTATTAGTTAGTAGTATAGTAGAAAACGGAGCTGGAACAGGATG
AACAGTTTACCCTCCTCTATCTTCTAATATTGCTCATGGAGGAGCTTCTGTTGATTTAGC
TATTTTCTCTCTTCATTTAGCAGGAATTTCTTCAATTTTAGGAGCTGTAAATTTTATTAC
TACAGTTATTAATATACGATCAACAGGAATTACTTTTGATCGAATACCTTTATTTGTTTG
ATCAGTAGTAATTACAGCTTTATTACTTTTATTATCATTACCAGTATTAGCAGGAGCTAT
TACAATACTTTTAACAGACCGAAATCTTAATACATCATTCTTTGACCCTGCAGGAGGAGG
AGATCCAATTTTATACCAACATTTATTTAATAATTAATAATTCCATAAGTAGAAATATAAGGTATAAACCACATAGAA

CCTAAAAAATAAGAAAAATTATAATTATTTAAAGCCTTATTATAAAAAAATAAAGAAACA

TTAGAAATTATATAACCTATAATACCCCCAACAATACAAACAAATAATGTTAATAATTTT

AAATAAGAAGGAAGAACAACTACTATAGGAGTAGGAAAAATCAATCATCTTAACATTCTC

CCACCAAAAATTCTTAAAATTAATAAACCTATTATACTTTTCAATATTACTCAACCTTCA

TCATTTAATATATTTAAAGAAGAAAAATTAGAATCACCA

GTTGTCTTCGCTCCCGCTAACATCACCACTGAAGTCAAGT

CCGTTGAAATGCACCACGAAGCTCTCGTTGAAGCCGTCCCCGGTGACAACGTTGGTTTCA

ACGTAAAGAACGTCTCCGTTAAGGAATTGCGTCGTGGTTACGTCGCTGGTGACTCCAAGG

CCAACCCCCCCAAGGGCGCCGCTGACTTCACCGCTCAAGTCATCGTCTTGAACCACCCTG

GTCAAATCTCCAACGGTTACACCCCCGTTTTGGATTGTCACACCGCTCACATTGCTTGCA

AATTCGCCGAAATCAAGGAGAAGGTCGAT

>7ITLs

AGAATTCTAATTCGAGCTGAATTAGGACATC
CTGGAGCTTTAATTGGAGATGATCAAATTTATAATGTAATTGTTACAGCTCATGCTTTTA
TTATAATTTTTTTTATAGTAATGCCAATTATAATTGGAGGATTTGGAAATTGATTAGTTC
CATTAATACTAGGAGCTCCAGATATAGCATTCCCTCGAATAAATAATATAAGTTTTTGAC
TTTTACCTCCTGCATTAACTTTATTATTAGTTAGTAGTATAGTAGAAAACGGAGCTGGAA
CAGGATGAACAGTTTACCCTCCTCTATCTTCTAATATTGCTCATGGAGGAGCTTCTGTTG
ATTTAGCTATTTTCTCTCTTCATTTAGCAGGAATTTCTTCAATTTTAGGAGCTGTAAATT
TTATTACTACAGTTATTAATATACGATCAACAGGAATTACTTTTGATCGAATACCTTTAT
TTGTTTGATCAGTAGTAATTACAGCTTTATTACTTTTATTATCATTACCAGTATTAGCAG
GAGCTATTACAATACTTTTAACAGACCGAAATCTTAATACATCATTCTTTGACCCTGCAG
GAGGAGGAGATCCAATTTTATACCAACATTTATTTAATAATTAATAATTCCATAAGTAGAAATATAAGGTATAAACCACATAG

AACCTAAAAAATAAGAAAAATTATAATTATTTAAAGCCTTATTATAAAAAAATAAAGAAA

CATTAGAAATTATATAACCTATAATACCCCCAACAATACAAACAAATAATGTTAATAATT

TTAAATAAGAAGGAAGAACAACTACTATAGGAGTAGGAAAAATCAATCATCTTAACATTC

TCCCACCAAAAATTCTTAAAATTAATAAACCTATTATACTTTTCAATATTACTCAACCTT

CATCATTTAATATATTTAAAGAAGAAAAATTAGAATCACCA

GTTGTCTTCGCTCCCGCTAACATCACCACTGAAGTCAAGTCCGTTG

AAATGCACCACGAAGCTCTCGTTGAAGCCGTCCCCGGTGACAACGTTGGTTTCAACGTAA

AGAACGTCTCCGTCAAGGAATTGCGTCGTGGTTACGTCGCTGGTGACTCCAAGGCCAACC

CCCCCAAGGGCGCCGCTGACTTCACCGCTCAAGTCATCGTCTTGAACCACCCTGGTCAAA

TCTCCAACGGTTACACCCCCGTTTTGGATTGTCACACCGCTCACATTGCTTGCAAATTCG

CCGAAATCAAGGAGAAGGTCGAT

>8ITLs

AGAATTCTAATTCGAGCTGAATTAGGACATCCTGGAG
CTTTAATTGGAGACGATCAAATTTATAATGTAATTGTTACAGCTCATGCTTTTATTATAA
TTTTTTTTATAGTAATGCCAATTATAATTGGAGGATTTGGAAATTGATTAGTTCCATTAA
TACTAGGAGCTCCAGATATAGCATTCCCTCGAATAAATAATATAAGTTTTTGACTTTTAC
CTCCTGCATTAACTTTATTATTAGTTAGTAGTATAGTAGAAAACGGAGCTGGAACAGGAT
GAACAGTTTACCCTCCTCTATCTTCTAATATTGCTCATGGAGGAGCTTCTGTTGATTTAG
CTATTTTCTCTCTTCATTTAGCAGGAATTTCTTCAATTTTAGGAGCTGTAAATTTTATTA
CTACAGTTATTAATATACGATCAACAGGAATTACTTTTGATCGAATACCTTTATTTGTTT
GATCAGTAGTAATTACAGCTTTATTACTTTTATTATCATTACCAGTATTAGCAGGAGCTA
TTACAATACTTTTAACAGACCGAAATCTTAATACATCATTCTTTGACCCTGCAGGAGGAG
GAGATCCAATTTTATACCAACATTTATTTAATAATTAATAATTCCATAAGTAGAAATATAAGAGT

ATAAACCACATAGAACCTAAAAAATAAGAAAAATTATAATTATTTAAAGCCTTATTATAA

AAAAATAAAGAAACATTAGAAATTATATAACCTATAATACCCCCAACAATACAAACAAAT

AATGTTAATAATTTTAAATAAGAAGGAAGAACAACTACTATAGGAGTAGGAAAAATCAAT

CATCTTAACATTCTCCCACCAAAAATTCTTAAAATTAATAAACCTATTATACTTTTCAAT

ATTACTCAACCTTCATCATTTAATATATTTAAAGAAGAAAAATTAGAATCACCA

GTTGTCTTCGCCCCCGCTAACATCACCACTGAAGTCAAGTC

CGTTGAAATGCACCACGAAGCTCTCGTTGAAGCCGTCCCCGGTGACAACGTTGGTTTCAA

CGTAAAGAACGTCTCCGTCAAGGAATTGCGTCGTGGTTACGTCGCTGGTGACTCCAAGGC

CAACCCCCCCAAGGGCGCCGCTGACTTCACCGCTCAAGTCATCGTCTTGAACCACCCTGG

TCAAATCTCCAACGGTTACACCCCCGTTTTGGATTGTCACACCGCTCACATTGCTTGCAA

ATTCGCCGAAATCAAGGAGAAGGTCGAT

>9ITLs

AGAATTCTAATTCGAGCTGAATTAGGACATCCT
GGAGCTTTAATTGGAGATGATCAAATTTATAATGTAATTGTTACAGCTCATGCTTTTATT
ATAATTTTTTTTATAGTAATGCCAATTATAATTGGAGGATTTGGAAATTGATTAGTTCCA
TTAATACTAGGAGCTCCAGATATAGCATTCCCTCGAATAAATAATATAAGTTTTTGACTT
TTACCTCCTGCATTAACTTTATTATTAGTTAGTAGTATAGTAGAAAACGGAGCTGGAACA
GGATGAACAGTTTACCCTCCTCTATCTTCTAATATTGCTCATGGAGGAGCTTCTGTTGAT
TTAGCTATTTTCTCTCTTCATTTAGCAGGAATTTCTTCAATTTTAGGAGCTGTAAATTTT
ATTACTACAGTTATTAATATACGATCAACAGGAATTACTTTTGATCGAATACCTTTATTT
GTTTGATCAGTAGTAATTACAGCTTTATTACTTTTATTATCATTACCAGTATTAGCAGGA
GCTATTACAATACTTTTAACAGACCGAAATCTTAATACATCATTCTTTGACCCTGCAGGA
GGAGGAGATCCAATTTTATACCAACATTTATTTAATAATTAATAATTCCATAAGTAGAAATATAAGGTATAAACCAC

ATAGAACCTAAAAAATAAGAAAAATTATAATTATTTAAAGCCTTATTATAAAAAAATAAA

GAAACATTAGAAATTATATAACCTATAATACCCCCAACAATACAAACAAATAATGTTAAT

AATTTTAAATAAGAAGGAAGAACAACTACTATAGGAGTAGGAAAAATCAATCATCTTAAC

ATTCTCCCACCAAAAATTCTTAAAATTAATAAACCTATTATACTTTTCAATATTACTCAA

CCTTCATCATTTAATATATTTAAAGAAGAAAAATTAGAATCACCA

GTTGTCTTCGCTCCCGCTAACATCACCACTGAAGTCAAGT

CCGTTGAAATGCACCACGAAGCTCTCGTTGAAGCCGTCCCCGGTGACAACGTTGGTTTCA

ACGTAAAGAACGTCTCCGTCAAGGAATTGCGTCGTGGTTACGTCGCTGGTGACTCCAAGG

CCAACCCCCCCAAGGGCGCCGCTGACTTCACCGCTCAAGTCATCGTCTTGAACCACCCTG

GTCAAATCTCCAACGGTTACACCCCCGTTTTGGATTGTCACACCGCTCACATTGCTTGCA

AATTCGCCGAAATCAAGGAGAAGGTCGAT

>ITChalb

AGAATTCTAATTCGAGCTGAATTAGGACAT

CCTGGAGCACTAATTGGAGATGACCAAATTTATAATGTAATTGTAACAGCTCATGCCTTT

ATTATAATTTTCTTTATAGTAATACCAATTATAATTGGAGGATTTGGAAATTGACTAGTT

CCTTTAATATTAGGAGCCCCAGATATAGCTTTCCCACGAATAAATAATATAAGTTTCTGA

CTTTTACCTCCTGCATTAACTTTACTATTAGTAAGTAGTATAGTAGAAAATGGAGCTGGA

ACAGGATGAACTGTTTATCCACCTTTATCATCTAATATTGCTCATGGTGGAGCATCAGTT

GATTTAGCTATTTTTTCTTTACACTTAGCTGGAATTTCATCAATTTTAGGAGCTGTAAAT

TTTATTACAACTGTTATTAATATACGATCTACAGGAATCACATTTGATCGAATACCTTTA

TTCGTATGATCTGTAGTTATTACTGCTCTTCTTTTATTATTATCATTACCAGTATTAGCC

GGTGCAATTACTATATTATTAACTGATCGAAATTTAAATACTTCATTCTTTGATCCAGCA

GGAGGAGGAGATCCTATTTTATATCAACATTTATTTAATAATTAATAATACCATATGTAGAAATATATGGTATAAATCAT

ATTGAACCTAAAAAATAAGAAGAATTATAATTATTTAATGCCTTATTATAAAAAAATAAT

GAAACATTAGAAATTATATAACCACTTACTCCTCCTACAATACAAACAAATATAGTTAAC

AACTTTAAATATATTGGAAGAACTACTAACACTGGAGTAGGAAAAATTAATCATCTTAAT

ATTCTCCCTCCAAAAATTCTTAGAATCAATAAACCTATTATACTTTTTAATATTACTCAA

CCTTCATCATTTAATATATTTAAAGATGAAAAATTTGAATCTCCA

GTTGTCTTCGCTCCCGCTAACATCACCAC

TGAAGTTAAGTCCGTTGAAATGCACCACGAAGCTCTCGCTGAAGCCGTACCCGGTGACAA

CGTTGGTTTCAACGTAAAGAACGTCTCCGTCAAGGAATTGCGTCGTGGTTACGTTGCTGG

TGACTCCAAGGCTAACCCACCCAAGGGTGCTGCTGACTTCACCGCTCAAGTCATTGTCTT

GAACCACCCTGGTCAAATCTCCAACGGTTACACCCCCGTTTTGGATTGTCACACCGCTCA

CATTGCTTGCAAATTCGCTGAAATCAAGGAGAAGGTCGAT

>3ITCvi

AGAATTCTAATTCGAGCCGAACTAGGACATCCTGGAGC
ATTAATTGGAGATGACCAAATTTATAATGTAATTGTTACAGCTCATGCTTTTATTATAAT
TTTTTTTATAGTAATACCAATTATAATTGGAGGATTTGGTAATTGATTAGTCCCTTTAAT
ATTAGGAGCTCCAGATATAGCCTTCCCTCGGATAAACAATATAAGTTTCTGACTTTTACC
TCCTGCATTAACTTTACTATTAGTAAGTAGTATAGTAGAAAACGGAGCTGGAACAGGATG
AACTGTTTACCCACCTTTATCTTCTAATATCGCTCATGGAGGAGCTTCTGTTGATTTAGC
TATTTTTTCTTTACACTTAGCAGGAATTTCTTCAATTTTAGGAGCTGTAAATTTTATTAC
TACAGTTATTAATATACGATCAACAGGAATTACATTCGACCGAATACCATTATTTGTTTG
ATCTGTAGTAATTACAGCTTTATTACTTTTATTATCTTTACCAGTATTAGCAGGTGCTAT
TACTATATTATTAACAGATCGAAATCTTAATACATCATTCTTTGACCCAGCAGGAGGAGG
AGACCCAATCTTGTACCAACATTTATTT AATAATTAATAATTCCATATGTAGAAATATAAGGTATAAATCATATTGAACCTAA

AAAGTAAGAAGAATTATAATTATTTAAAGCCTTATTATAAAAAAATAAAGAAATATTAGA

AATTAAATAACCTCTAACTCCTCCTACAATACAAACAAATAAAGTTAATAATTTTAAATA

AGAAGGAAGAACTACTACTACTGGTGTAGGAAAAATTAATCAACTTAATATACTACCCCC

AAAAATTCTCAAAATTAATAGTCCTATTATACTTTTTAATATAACTCAACCTTCATCATT

TAGTATATTTAAAGAAGAAAAATTTGAATCCCCA

GTTGTCTTCGCTCCCGCTAACATCACCAC

TGAAGTCAAGTCCGTAGAAATGCACCACGAAGCTCTCGTTGAAGCTGTTCCCGGTGACAA

CGTTGGTTTCAACGTTAAGAACGTCTCCGTCAAGGAATTGCGTCGTGGTTACGTCGCTGG

TGACTCCAAGGCTAGCCCCCCCAAGGGTGCTGCTGACTTCACCGCTCAAGTCATCGTCTT

GAACCACCCTGGTCAAATCTCCAACGGTTACACTCCCGTTTTGGATTGTCACACCGCTCA

CATTGCTTGCAAATTCGCCGAAATCAAGGAGAAGGTCGAT

>1BGLs

AGAATTCTAATTCGAGCTGAATTAGGACATCCTGG
AGCTTTAATTGGAGATGATCAAATTTATAATGTAATTGTTACAGCTCATGCTTTTATTAT
AATTTTTTTTATAGTAATGCCAATTATAATTGGAGGATTTGGAAATTGATTAGTTCCATT
AATACTAGGAGCTCCAGATATAGCATTCCCTCGAATAAATAATATAAGTTTTTGACTTTT
ACCTCCTGCATTAACTTTATTATTAGTTAGTAGTATAGTAGAAAACGGAGCTGGAACAGG
ATGAACAGTTTACCCTCCTCTATCTTCTAATATTGCTCATGGAGGAGCTTCTGTTGATTT
AGCTATTTTCTCTCTTCATTTAGCAGGAATTTCTTCAATTTTAGGAGCTGTAAATTTTAT
TACTACAGTTATTAATATACGATCAACAGGAATTACTTTTGATCGAATACCTTTATTTGT
TTGATCAGTAGTAATTACAGCTTTATTACTTTTATTATCATTACCAGTATTAGCAGGAGC
TATTACAATACTTTTAACAGACCGAAATCTTAATACATCATTCTTTGACCCTGCAGGAGG
AGGAGATCCAATTTTATACCAACATTTATTTAATAATTAATAATTCCATAAGTAGAAATATAAGGTATAAACCACATAGA

ACCTAAAAAATAAGAAAAATTATAATTATTTAAAGCCTTATTATAAAAAAATAAAGAAAC

ATTAGAAATTATATAACCTATAATACCCCCAACAATACAAACAAATAATGTTAATAATTT

TAAATAAGAAGGAAGAACAACTACTATAGGAGTAGGAAAAATCAATCATCTTAACATTCT

CCCACCAAAAATTCTTAAAATTAATAAACCTATTATACTTTTCAATATTACTCAACCTTC

ATCATTTAATATATTTAAAGAAGAAAAATTAGAATCACCA
GTTGTCTTCGCTCCCG

CTAACATCACCACTGAAGTCAAGTCCGTTGAAATGCACCACGAAGCTCTCGTTGAAGCCG

TCCCCGGTGACAACGTTGGTTTCAACGTAAAGAACGTCTCCGTCAAGGAATTGCGTCGTG

GTTACGTCGCTGGTGACTCCAAGGCCAACCCCCCCAAGGGCGCCGCTGACTTCACCGCTC

AAGTCATCGTCTTGAACCACCCTGGTCAAATCTCCAACGGTTACACCCCCGTTTTGGATT

GTCACACCGCTCACATTGCTTGCAAATTCGCCGAAATCAAGGAGAAGGTCGAT

>2BGCvi

AGAATTCTAATTCGAGCCGAACTAGGACA
TCCTGGAGCATTAATTGGAGATGACCAAATTTATAATGTAATTGTTACAGCTCATGCTTT
TATTATAATTTTTTTTATAGTAATACCAATTATAATTGGAGGATTTGGTAATTGATTAGT
CCCTTTAATATTAGGAGCTCCAGATATAGCCTTCCCTCGGATAAACAATATAAGTTTCTG
ACTTTTACCTCCTGCATTAACTTTACTATTAGTAAGTAGTATAGTAGAAAACGGAGCTGG
AACAGGATGAACTGTTTACCCACCTTTATCTTCTAATATCGCTCATGGAGGAGCTTCTGT
TGATTTAGCTATTTTTTCTTTACACTTAGCAGGAATTTCTTCAATTTTAGGAGCTGTAAA
TTTTATTACTACAGTTATTAATATACGATCAACAGGAATTACATTCGACCGAATACCATT
ATTTGTTTGATCTGTAGTAATTACAGCTTTATTACTTTTATTATCTTTACCAGTATTAGC
AGGTGCTATTACTATATTATTAACAGATCGAAATCTTAATACATCATTCTTTGATCCAGC
AGGAGGAGGAGACCCAATCTTGTACCAACATTTATTT AATAATTAATAATTCCATATGTAGAAATATAAGGTATAAATCATATT

GAACCTAAAAAGTAAGAAGAATTATAATTATTTAAAGCCTTATTATAAAAAAATAAAGAA

ATATTAGAAATTAAATAACCTCTAACTCCTCCTACAATACAAACAAATAAAGTTAATAAT

TTTAAATAAGAAGGAAGAACTACTACTACTGGTGTAGGAAAAATTAATCAACTTAATATA

CTACCCCCAAAAATTCTCAAAATTAATAGTCCTATTATACTTTTTAATATAACTCAACCT

TCATCATTTAGTATATTTAAAGAAGAAAAATTTGAATCCCCAGTTGTCTTCGCTCCCGCTAACATCACCACTGAAGTCAAGTCCG

TAGAAATGCACCACGAAGCTCTCGTTGAAGCTGTTCCCGGTGACAACGTTGGTTTCAACG

TTAAGAACGTCTCCGTCAAGGAATTGCGTCGTGGTTACGTCGCTGGTGACTCCAAGGCTA

GCCCCCCCAAGGGTGCTGCTGACTTCACCGCTCAAGTCATCGTCTTGAACCACCCTGGTC

AAATCTCCAACGGTTACACTCCCGTTTTGGATTGTCACACCGCTCACATTGCTTGCAAAT

TCGCCGAAATCAAGGAGAAGGTCGAT

>3BGCym

AGAATTTTAATTCGAGCCGAACTAGGACACCCTGGAGCATTAAT
TGGTGATGACCAAATCTATAATGTAATTGTTACAGCTCATGCTTTTATTATAATTTTTTT
TATAGTAATACCAATTATAATTGGAGGGTTTGGTAATTGATTAGTCCCTTTAATGTTAGG
AGCCCCAGATATAGCTTTCCCTCGGATAAACAATATAAGTTTCTGACTTTTACCTCCTGC
ATTAACTTTACTATTAGTAAGTAGTATAGTGGAAAACGGAGCTGGAACAGGATGAACTGT
TTATCCACCTTTATCATCTAATATTGCTCATGGAGGAGCTTCTGTTGATTTAGCTATTTT
TTCTTTACATTTAGCGGGAATTTCTTCAATTTTAGGAGCTGTAAATTTTATTACAACAGT
TATTAATATACGATCAACAGGAATTACTTTTGACCGAATACCCCTATTCGTTTGATCAGT
AGTAATTACAGCTTTATTACTTTTACTATCTTTACCTGTTCTAGCTGGGGCTATTACTAT
ATTATTAACAGACCGAAACCTTAATACTTCATTCTTCGACCCAGCAGGAGGAGGAGACCC
AATTCTATACCAACACTTATTTAATAATTAATAATTCCGTATGTAGAAATATAAGGTATAAATCACATT

GACCCTAAAAAGTAAGAAGAGTTATAATTATTTAAAGCCTTATTGTAAAAAAATAAAGAA

ATGTTAGAAATTAAATAACCTCTAACTCCCCCTACAATACAAACAAATAAAGTTAATAAT

TTTAAATAATAAGGAAGAACCACTACTACAGGGGTAGGAAAAATCAATCAACTTAATATT

CTACCTCCAAAGATTCTCAAAATTAATAACCCTATTATACTTTTTAATATAACTCAACCT

TCATCATTTAATATATTTAAAGAAGAAAAATTAGAATCCCCAGTTGTCTTCGCTCCCGCTAACATCACCAC

TGAAGTCAAGTCCGTAGAAATGCACCACGAAGCTCTCGTTGAAGCCGTTCCCGGTGACAA

CGTTGGTTTCAACGTTAAGAACGTCTCCGTCAAGGAATTGCGTCGTGGTTACGTCGCTGG

TGACTCCAAGGCTAGCCCCCCCAAGGGTGCTGCTGACTTCACCGCTCAAGTCATCGTCTT

GAACCACCCTGGTCAAATCTCCAACGGTTACACTCCCGTTTTGGATTGTCACACCGCTCA

CATTGCTTGCAAATTCGCCGAAATCAAGGAGAAGGTCGAT

>CvoUK

AGAATTTTAATTCGAGCTGAACTAGGGCAT
CCTGGAGCATTAATTGGAGATGATCAAATTTATAATGTAATTGTTACAGCTCATGCTTTT
ATTATAATTTTTTTTATAGTAATACCAATTATAATTGGAGGGTTTGGAAATTGATTAGTT
CCTTTAATATTAGGAGCCCCAGATATAGCATTCCCTCGAATAAATAATATAAGTTTCTGA
CTTTTACCTCCTGCATTAACTTTACTATTAGTAAGTAGTATAGTAGAAAACGGAGCTGGA
ACTGGATGAACTGTTTATCCACCTTTATCTTCTAATATTGCACATGGAGGAGCTTCTGTT
GATTTAGCTATTTTTTCTTTACATTTAGCAGGAATTTCTTCAATTTTAGGAGCTGTAAAT
TTTATTACTACAGTTATTAATATACGATCAACAGGTATTACCTTCGACCGAATACCATTA
TTTGTTTGATCAGTAGTAATTACAGCCTTATTACTTTTATTATCTTTACCAGTATTAGCA
GGAGCTATTACTATATTATTAACAGATCGAAATCTTAATACTTCATTCTTTGACCCAGCA
GGAGGAGGAGATCCAATTTTATACCAACACTTATTTAGTAATTAATAATTCCGTATGTAGAAATATAAGGCATAAACCACATTGAC

CCTAAAAAATAAGAAGAATTATAGTTATTTAAAGCCTTATTATAAAAAAATAAAGAAATA

TTAGAAATTAAATACCCTCTAATTCCTCCTACAATACAAACAAATAAAGTTAATAACTTT

AAATAAGAAGGTAGAACTACTACTACTGGTGTAGGAAAAATTAATCAACTTAACATTCTA

CCCCCAAAAATTCTTAAAATTAATAACCCTATTATACTCTTTAACATAACTCAACCTTCA

TCATTTAATATATTTAAAGAAGAAAAATTTGAATCCCCAGTTGTCTTCGCTCCCGCTAACATCACCACTGAAGTCAAAT

CCGTAGAAATGCACCACGAAGCTCTCGTTGAAGCCGTCCCCGGTGACAACGTTGGTTTCA

ACGTTAAGAACGTCTCCGTCAAGGAATTGCGTCGTGGTTACGTCGCTGGTGACTCCAAGG

CTAGCCCCCCCAAGGGTGCTGCTGACTTCACCGCTCAAGTCATCGTCTTGAACCACCCTG

GTCAAATCTCCAACGGTTACACTCCCGTTTTGGATTGTCACACCGCTCACATTGCTTGCA

AATTCGCCGAAATCAAGGAGAAGGTCGAT

>ITMO3Cvi

AGAATTCTAATTCGAGCCGAACTAGGACATCCTGGAGCATTAATTGGAGATGACCAAATTTATAATGTAATTGTTACAGCTCATGCTTTTATTA
TAATTTTTTTTATAGTAATACCAATTATAATTGGAGGATTTGGTAATTGATTAGTTCCTT
TAATATTAGGAGCTCCAGATATAGCATTCCCTCGGATAAACAATATAAGTTTCTGACTTT
TACCTCCTGCATTAACTTTACTATTAGTAAGTAGTATAGTAGAAAACGGAGCTGGAACAG
GATGAACTGTTTACCCACCTTTATCTTCTAATATCGCTCATGGAGGAGCTTCTGTTGATT
TAGCTATTTTTTCTTTACACTTGGCAGGAATTTCTTCAATTTTAGGAGCTGTAAATTTTA
TTACTACAGTTATTAATATACGATCAACAGGAATTACATTCGACCGAATACCATTATTTG
TTTGATCTGTAGTAATTACAGCTTTATTACTTTTATTATCTTTACCAGTATTAGCAGGTG
CTATTACTATATTATTAACAGATCGAAATCTTAATACATCATTCTTTGACCCAGCAGGAG
GAGGAGACCCAATCTTGTACCAACATTTATTTAATAATTAATAATTCCATATGTAGAAATATAAGGTATAAATCATATTG

AACCTAAAAAGTAAGAAGAATTATAATTATTTAAAGCCTTATTATAAAAAAATAAAGAAA

TATTAGAAATTAAATAACCTCTAACTCCTCCTACAATACAAACAAATAAAGTTAATAATT

TTAAATAAGAAGGAAGAACTACTACTACTGGTGTAGGAAAAATTAATCAACTTAATATAC

TACCCCCAAAAATTCTCAAAATTAATAGTCCTATTATACTTTTTAATATAACTCAACCTT

CATCATTTAGTATATTTAAAGAAGAAAAATTTGAATCCCCAGTTGTCTTCGCTCCCGCTAACATCACCACTGAAGTCAAGT

CCGTAGAAATGCACCACGAAGCTCTCGTTGAAGCTGTTCCCGGTGACAACGTTGGTTTCA

ACGTTAAGAACGTCTCCGTCAAGGAATTGCGTCGTGGTTACGTCGCTGGTGACTCCAAGG

CTAGCCCCCCCAAGGGTGCTGCTGACTTCACCGCTCAAGTCATCGTCTTGAACCACCCTG

GTCAAATCTCCAACGGTTACACTCCCGTTTTGGATTGTCACACCGCTCACATTGCTTGCA

AATTCGCCGAAATCAAGGAGAAGGTCGAT

>ITMA3Lsi

AGAATTCTAATTCGAGCTGAATTAGGACATCCTGGAGCTTT
AATTGGAGATGATCAAATTTATAATGTAATTGTTACAGCTCATGCTTTTATTATAATTTT
TTTTATAGTAATGCCAATTATAATTGGAGGATTTGGAAATTGATTAGTTCCATTAATACT
AGGAGCTCCAGATATAGCATTCCCTCGAATAAATAATATAAGTTTTTGACTTTTACCTCC
TGCATTAACTTTATTATTAGTTAGTAGTATAGTAGAAAACGGAGCTGGAACAGGATGAAC
AGTTTACCCTCCTCTATCTTCTAATATTGCTCATGGAGGAGCTTCTGTTGATTTAGCTAT
TTTCTCTCTTCATTTAGCAGGAATTTCTTCAATTTTAGGAGCTGTAAATTTTATTACTAC
AGTTATTAATATACGATCAACAGGAATTACTTTTGATCGAATACCTTTATTTGTTTGATC
AGTAGTAATTACAGCTTTATTACTTTTATTATCATTACCAGTATTAGCAGGAGCTATTAC
AATACTTTTAACAGACCGAAATCTTAATACATCATTCTTTGACCCTGCAGGAGGAGGAGA
TCCAATTTTATACCAACATTTATTTGATAATTAATAATTCCATAAGTAGAAATATAAGGTATAAACCACATAGAACCTAA

AAAATAAGAAAAATTATAATTATTTAAAGCCTTATTATAAAAAAATAAAGAAACATTAGA

AATTATATAACCTATAATACCCCCAACAATACAAACAAATAATGTTAATAATTTTAAATA

AAAAGGAAGAACAACTACTATAGGAGTAGGAAAAATCAATCATCTTAACATTCTCCCACC

AAAAATTCTTAAAATTAATAAACCTATTATACTTTTCAATATTACTCAACCTTCATCATT

TAATATATTTAAAGAAGAAAAATTAGAATCACCA

GTTGTCTTCGCTCCCGCTAACATCACCACTGAAGTCAAGTCCGTTGAAATGCACCACGA

AGCTCTCGTTGAAGCCGTCCCCGGTGACAACGTTGGTTTCAACGTAAAGAACGTCTCCGT

CAAGGAATTGCGTCGTGGTTACGTCGCTGGTGACTCCAAGGCCAACCCCCCCAAGGGCGC

CGCTGACTTCACCGCTCAAGTCATCGTCTTGAACCACCCTGGTCAAATCTCCAACGGTTA

CACCCCCGTTTTGGATTGTCACACCGCTCACATTGCTTGCAAATTCGCCGAAATCAAGGA

GAAGGTCGAT

>ITMO2Li

AGAATTCTAATTCGAGCTGAATTAGGACACC
CTGGTGCACTAATTGGAGATGACCAAATTTATAATGTAATTGTTACAGCTCATGCTTTTA
TTATAATTTTCTTTATAGTAATACCAATTATAATTGGAGGATTTGGTAATTGATTAGTTC
CTTTAATGTTAGGAGCCCCAGATATAGCCTTCCCTCGATTAAATAATATAAGATTTTGAC
TTTTACCTCCTGCACTAACTTTATTATTAGTAAGTAGTATAGTAGAAAACGGAGCTGGAA
CAGGATGAACAGTTTACCCCCCTTTATCATCTAATATCGCTCATGGAGGAGCTTCTGTTG
ATTTAGCTATTTTTTCTCTTCATTTAGCAGGAATTTCTTCAATTTTAGGGGCTGTAAATT
TTATTACTACAGTCATTAATATACGATCAACAGGAATTACTTTTGACCGAATACCTTTAT
TTGTTTGATCAGTTGTAATTACAGCTCTATTACTTTTATTATCATTACCAGTATTAGCAG
GAGCTATTACAATACTTTTAACTGACCGAAACCTTAATACTTCATTTTTTGACCCTGCAG
GAGGAGGAGACCCAATTCTATACCAACATCTATTT AATAATTAATAATTCCGTAAGTAGAAATATAAGGTATAAATCATA

TTGATCCTAAAAAATAAGAAAAATTATAATTATTTAAAGCCTTATTATAAAAAAATAAAG

AAATATTTGAAATTATATAACCTCTTACTCCTCCTACAATACAAACAAATAATGTTAACA

ACTTTAAATATGAAGGTAATACTACTACTACTGGGGTAGGAAAAATTAATCATCTTAACA

TTCTCCCCCCAAAAATTCTTAAAATTAATAAACCTATTATACTTTTTAATATAACTCAAC

CTTCATCATTTAATATATTTAAAGAAGAAAAATTTGAATCACCAGTTGTCTTCGCTCCCGCTAA

CATCACCACTGAAGTCAAGTCCGTTGAAATGCACCACGAAGCTCTCGTTGAAGCCGTTCC

CGGTGACAACGTTGGTTTCAACGTAAAGAACGTCTCCGTCAAGGAATTGCGTCGTGGTTA

CGTCGCTGGTGACTCCAAGGCCAGCCCCCCCAAGGGTGCCGCTGACTTCACCGCTCAAGT

CATCGTCTTGAACCATCCCGGTCAAATCTCCAACGGTTACACTCCCGTTTTGGATTGTCA

CACCGCTCACATTGCTTGCAAATTCGCCGAAATCAAGGAGAAGGTCGAT

>4ITCvi

AGAATTCTAATTCGAGCCGAACTAGGAC
ATCCTGGAGCATTAATTGGAGATGACCAAATTTATAATGTAATTGTTACAGCTCATGCTT
TTATTATAATTTTTTTTATAGTAATACCAATTATAATTGGAGGATTTGGTAATTGATTAG
TCCCTTTAATATTAGGAGCTCCAGATATAGCCTTCCCTCGGATAAACAATATAAGTTTCT
GACTTTTACCTCCTGCATTAACTTTACTATTAGTAAGTAGTATAGTAGAAAACGGAGCTG
GAACAGGATGAACTGTTTACCCACCTTTATCTTCTAATATCGCTCATGGAGGAGCTTCTG
TTGATTTAGCTATTTTTTCTTTACACTTAGCAGGAATTTCTTCAATTTTAGGAGCTGTAA
ATTTTATTACTACAGTTATTAATATACGATCAACAGGAATTACATTCGACCGAATACCAT
TATTTGTTTGATCTGTAGTAATTACAGCTTTATTACTTTTATTATCTTTACCAGTATTAG
CAGGTGCTATTACTATATTATTAACAGATCGAAATCTTAATACATCATTCTTTGACCCAG
CAGGAGGAGGAGACCCAATCTTGTACCAACATTTATTTAATAATTAATAATTCCATATGTAGAAATATAAGGTATAAATCATATTGA

ACCTAAAAAGTAAGAAGAATTATAATTATTTAAAGCCTTATTATAAAAAAATAAAGAAAT

ATTAGAAATTAAATAACCTCTAACTCCTCCTACAATACAAACAAATAAAGTTAATAATTT

TAAATAAGAAGGAAGAACTACTACTACTGGTGTAGGAAAAATTAATCAACTTAATATACT

ACCCCCAAAAATTCTCAAAATTAATAGTCCTATTATACTTTTTAATATAACTCAACCTTC

ATCATTTAGTATATTTAAAGAAGAAAAATTTGAATCCCCAGTTGTCTTCGCTCCCGCTAACATCACCACTG

AAGTCAAGTCCGTAGAAATGCACCACGAAGCTCTCGTTGAAGCTGTTCCCGGTGACAACG

TTGGTTTCAACGTTAAGAACGTCTCCGTCAAGGAATTGCGTCGTGGTTACGTCGCTGGTG

ACTCCAAGGCTAGCCCCCCCAAGGGTGCTGCTGACTTCACCGCTCAAGTCATCGTCTTGA

ACCACCCTGGTCAAATCTCCAACGGTTACACTCCCGTTTTGGATTGTCACACCGCTCACA

TTGCTTGCAAATTCGCCGAAATCAAGGAAAAGGTCGAT

>ITMO1Cvo

AGAATTTTAATTCGAGCTGAACTAGGGCATC
CTGGAGCATTAATTGGAGATGATCAAATTTATAATGTAATTGTTACAGCTCATGCTTTTA
TTATAATTTTTTTTATAGTAATACCAATTATAATTGGAGGGTTTGGAAATTGATTAGTTC
CTTTAATATTAGGAGCCCCAGATATAGCATTCCCTCGAATAAATAATATAAGTTTCTGAC
TTTTACCTCCTGCATTAACTTTACTATTAGTAAGTAGTATAGTAGAAAACGGAGCTGGAA
CTGGATGAACTGTTTATCCACCTTTATCTTCTAATATTGCACATGGAGGAGCTTCTGTTG
ATTTAGCTATTTTTTCTTTACATTTAGCAGGAATTTCTTCAATTTTAGGAGCTGTAAATT
TTATTACTACAGTTATTAATATACGATCAACAGGTATTACCTTCGACCGAATACCATTAT
TTGTTTGATCAGTAGTAATTACAGCCTTATTACTTTTATTATCTTTACCAGTATTAGCAG
GAGCTATTACTATATTATTAACAGATCGAAATCTTAATACTTCATTCTTTGACCCAGCAG
GAGGAGGAGATCCAATTTTATACCAACACTTATTT AGTAATTAATAATTCCGTATGTAGAAATATAAGGCATAAACCACA

TTGACCCTAAAAAATAAGAAGAATTATAGTTATTTAAAGCCTTATTATAAAAAAATAAAG

AAATATTAGAAATTAAATACCCTCTAATTCCTCCTACAATACAAACAAATAAAGTTAATA

ACTTTAAATAAGAAGGTAGAACTACTACTACTGGTGTAGGAAAAATTAATCAACTTAACA

TTCTACCCCCAAAAATTCTTAAAATTAATAACCCTATTATACTCTTTAACATAACTCAAC

CTTCATCATTTAATATATTTAAAGAAGAAAAATTTGAATCCCCA

GTTGTCTTCGCTCCCGCTAACATCACCACTGAAGT

CAAATCCGTAGAAATGCACCACGAAGCTCTCGTTGAAGCCGTCCCCGGTGACAACGTTGG

TTTCAACGTTAAGAACGTCTCCGTCAAGGAATTGCGTCGTGGTTACGTCGCTGGTGACTC

CAAGGCTAGCCCCCCCAAGGGTGCTGCTGACTTCACCGCTCAAGTCATCGTCTTGAACCA

CCCTGGTCAAATCTCCAACGGTTACACTCCCGTTTTGGATTGTCACACCGCTCACATTGC

TTGCAAATTCGCCGAAATCAAGGAGAAGGTCGAT

>5ITCvi

AGAATTCTAATTCGAGCCGAACTAGGACA
TCCTGGAGCATTAATTGGAGATGACCAAATTTATAATGTAATTGTTACAGCTCATGCTTT
TATTATAATTTTTTTTATAGTAATACCAATTATAATTGGAGGATTTGGTAATTGATTAGT
CCCTTTAATATTAGGAGCTCCAGATATAGCCTTCCCTCGGATAAACAATATAAGTTTCTG
ACTTTTACCTCCTGCATTAACTTTACTATTAGTAAGTAGTATAGTAGAAAACGGAGCTGG
AACAGGATGAACTGTTTACCCACCTTTATCTTCTAATATCGCTCATGGAGGAGCTTCTGT
TGATTTAGCTATTTTTTCTTTACACTTAGCAGGAATTTCTTCAATTTTAGGAGCTGTAAA
TTTTATTACTACAGTTATTAATATACGATCAACAGGAATTACATTCGACCGAATACCATT
ATTTGTTTGATCTGTAGTAATTACAGCTTTATTACTTTTATTATCTTTACCAGTATTAGC
AGGTGCTATTACTATATTATTAACAGATCGAAATCTTAATACATCATTCTTTGACCCAGC
AGGAGGAGGAGACCCAATCTTGTACCAACATTTATTTAATAATTAATAATTCCATATGTAGAAATATAAGGTATAAATCATATT

GAACCTAAAAAGTAAGAAGAATTATAATTATTTAAAGCCTTATTATAAAAAAATAAAGAA

ATATTAGAAATTAAATAACCTCTAACTCCTCCTACAATACAAACAAATAAAGTTAATAAT

TTTAAATAAGAAGGAAGAACTACTACTACTGGTGTAGGAAAAATTAATCAACTTAATATA

CTACCCCCAAAAATTCTCAAAATTAATAGTCCTATTATACTTTTTAATATAACTCAACCT

TCATCATTTAGTATATTTAAAGAAGAAAAATTTGAATCCCCA

GTTGTCTTCGCTCCCGCTAACATCACCA

CTGAAGTCAAGTCCGTAGAAATGCACCACGAAGCTCTCGTTGAAGCTGTTCCCGGTGACA

ACGTTGGTTTCAACGTTAAGAACGTCTCCGTCAAGGAATTGCGTCGTGGTTACGTCGCTG

GTGACTCCAAGGCTAGCCCCCCCAAGGGTGCTGCTGACTTCACCGCTCAAGTCATCGTCT

TGAACCACCCTGGTCAAATCTCCAACGGTTACACTCCCGTTTTGGATTGTCACACCGCTC

ACATTGCTTGCAAATTCGCCGAAATCAAGGAGAAGGTCGAT

>6ITCvi

AGAATTCTAATTCGAGCCGAACTAGGACAT
CCTGGAGCATTAATTGGAGATGACCAAATTTATAATGTAATTGTTACAGCTCATGCTTTT
ATTATAATTTTTTTTATAGTAATACCAATTATAATTGGAGGATTTGGTAATTGATTAGTC
CCTTTAATATTAGGAGCTCCAGATATAGCCTTCCCTCGGATAAACAATATAAGTTTCTGA
CTTTTACCTCCTGCATTAACTTTACTATTAGTAAGTAGTATAGTAGAAAACGGAGCTGGA
ACAGGATGAACTGTTTACCCACCTTTATCTTCTAATATCGCTCATGGAGGAGCTTCTGTT
GATTTAGCTATTTTTTCTTTACACTTAGCAGGAATTTCTTCAATTTTAGGAGCTGTAAAT
TTTATTACTACAGTTATTAATATACGATCAACAGGAATTACATTCGACCGAATACCATTA
TTTGTTTGATCTGTAGTAATTACAGCTTTATTACTTTTATTATCTTTACCAGTATTAGCA
GGTGCTATTACTATATTATTAACAGATCGAAATCTTAATACATCATTCTTTGACCCAGCA
GGAGGAGGAGACCCAATCTTGTACCAACATTTATTTAATAATTAATAATTCCATATGTAGAAATATAAGGTATAAATCATATTGA

ACCTAAAAAGTAAGAAGAATTATAATTATTTAAAGCCTTATTATAAAAAAATAAAGAAAT

ATTAGAAATTAAATAACCTCTAACTCCTCCTACAATACAAACAAATAAAGTTAATAATTT

TAAATAAGAAGGAAGAACTACTACTACTGGTGTAGGAAAAATTAATCAACTTAATATACT

ACCCCCAAAAATTCTCAAAATTAATAGTCCTATTATACTTTTTAATATAACTCAACCTTC

ATCATTTAGTATATTTAAAGAAGAAAAATTTGAATCCCCAGTTGTCTTCGCTCCCGCTAACATCACCACTGAAG

TCAAGTCCGTAGAAATGCACCACGAAGCTCTCGTTGAAGCTGTTCCCGGTGACAACGTTG

GTTTCAACGTTAAGAACGTCTCCGTCAAGGAATTGCGTCGTGGTTACGTCGCTGGTGACT

CCAAGGCTAGCCCCCCCAAGGGTGCTGCTGACTTCACCGCTCAAGTCATCGTCTTGAACC

ACCCTGGTCAAATCTCCAACGGTTACACTCCCGTTTTGGATTGTCACACCGCTCACATTG

CTTGCAAATTCGCCGAAATCAAGGAGAAGGTCGAT

>7ITCvi

AGAATTCTAATTCGAGCCGAACTAGGACATCCTGGAGC
ATTAATTGGAGATGACCAAATTTATAATGTAATTGTTACAGCTCATGCTTTTATTATAAT
TTTTTTTATAGTAATACCAATTATAATTGGAGGATTTGGTAATTGATTAGTCCCTTTAAT
ATTAGGAGCTCCAGATATAGCCTTCCCTCGGATAAACAATATAAGTTTCTGACTTTTACC
TCCTGCATTAACTTTACTATTAGTAAGTAGTATAGTAGAAAACGGAGCTGGAACAGGATG
AACTGTTTACCCACCTTTATCTTCTAATATCGCTCATGGAGGAGCTTCTGTTGATTTAGC
TATTTTTTCTTTACACTTAGCAGGAATTTCTTCAATTTTAGGAGCTGTAAATTTTATTAC
TACAGTTATTAATATACGATCAACAGGAATTACATTCGACCGAATACCATTATTTGTTTG
ATCTGTAGTAATTACAGCTTTATTACTTTTATTATCTTTACCAGTATTAGCAGGTGCTAT
TACTATATTATTAACAGATCGAAATCTTAATACATCATTCTTTGACCCAGCAGGAGGAGG
AGACCCAATCTTGTACCAACATTTATTTAATAATTAATAATTCCATATGTAGAAATATAAGGTATAAATCATATT

GAACCTAAAAAGTAAGAAGAATTATAATTATTTAAAGCCTTATTATAAAAAAATAAAGAA

ATATTAGAAATTAAATAACCTCTAACTCCTCCTACAATACAAACAAATAAAGTTAATAAT

TTTAAATAAGAAGGAAGAACTACTACTACTGGTGTAGGAAAAATTAATCAACTTAATATA

CTACCCCCAAAAATTCTCAAAATTAATAGTCCTATTATACTTTTTAATATAACTCAACCT

TCATCATTTAGTATATTTAAAGAAGAAAAATTTGAATCCCCAGTTGTCTTCGCTCCCGCTAACATCACCACTGAAGTCAAG

TCCGTAGAAATGCACCACGAAGCTCTCGTTGAAGCTGTTCCCGGTGACAACGTTGGTTTC

AACGTTAAGAACGTCTCCGTCAAGGAATTGCGTCGTGGTTACGTCGCTGGTGACTCCAAG

GCTAGCCCCCCCAAGGGTGCTGCTGACTTCACCGCTCAAGTCATCGTCTTGAACCACCCT

GGTCAAATCTCCAACGGTTACACTCCCGTTTTGGATTGTCACACCGCTCACATTGCTTGC

AAATTCGCCGAAATCAAGGAGAAGGTCGAT

>9ITCvi

AGAATTCTAATTCGAGCCGAACTAGGACATCCTGGA
GCATTAATTGGAGATGACCAAATTTATAATGTAATTGTTACAGCTCATGCTTTTATTATA
ATTTTTTTTATAGTAATACCAATTATAATTGGAGGATTTGGTAATTGATTAGTCCCTTTA
ATATTAGGAGCTCCAGATATAGCCTTCCCTCGGATAAACAATATAAGTTTCTGACTTTTA
CCTCCTGCATTAACTTTACTATTAGTAAGTAGTATAGTAGAAAACGGAGCTGGAACAGGA
TGAACTGTTTACCCACCTTTATCTTCTAATATCGCTCATGGAGGAGCTTCTGTTGATTTA
GCTATTTTTTCTTTACACTTAGCAGGAATTTCTTCAATTTTAGGAGCTGTAAATTTTATT
ACTACAGTTATTAATATACGATCAACAGGAATTACATTCGACCGAATACCATTATTTGTT
TGATCTGTAGTAATTACAGCTTTATTACTTTTATTATCTTTACCAGTATTAGCAGGTGCT
ATTACTATATTATTAACAGATCGAAATCTTAATACATCATTCTTTGACCCAGCAGGAGGA
GGAGACCCAATCTTGTACCAACATTTATTTAATAATTAATAATTCCATATGTAGAAATATAAGGTATAAATCATA

TTGAACCTAAAAAGTAAGAAGAATTATAATTATTTAAAGCCTTATTATAAAAAAATAAAG

AAATATTAGAAATTAAATAACCTCTAACTCCTCCTACAATACAAACAAATAAAGTTAATA

ATTTTAAATAAGAAGGAAGAACTACTACTACTGGTGTAGGAAAAATTAATCAACTTAATA

TACTACCCCCAAAAATTCTCAAAATTAATAGTCCTATTATACTTTTTAATATAACTCAAC

CTTCATCATTTAGTATATTTAAAGAAGAAAAATTTGAATCCCCA

GTTGTCTTCGCTCCCGCTAACATCACCACTGAAGTC

AAGTCCGTAGAAATGCACCACGAAGCTCTCGTTGAAGCTGTTCCCGGTGACAACGTTGGT

TTCAACGTTAAGAACGTCTCCGTCAAGGAATTGCGTCGTGGTTACGTCGCTGGTGACTCC

AAGGCTAGCCCCCCCAAGGGTGCTGCTGACTTCACCGCTCAAGTCATCGTCTTGAACCAC

CCTGGTCAAATCTCCAACGGTTACACTCCCGTTTTGGATTGTCACACCGCTCACATTGCT

TGCAAATTCGCCGAAATCAAGGAGAAGGTCGAT

>10ITCvi

AGAATTCTAATTCGAGCCGAACTAGGACATCCTGGAGCA
TTAATTGGAGATGACCAAATTTATAATGTAATTGTTACAGCTCATGCTTTTATTATAATT
TTTTTTATAGTAATACCAATTATAATTGGAGGATTTGGTAATTGATTAGTCCCTTTAATA
TTAGGAGCTCCAGATATAGCCTTCCCTCGGATAAACAATATAAGTTTCTGACTTTTACCT
CCTGCATTAACTTTACTATTAGTAAGTAGTATAGTAGAAAACGGAGCTGGAACAGGATGA
ACTGTTTACCCACCTTTATCTTCTAATATCGCTCATGGAGGAGCTTCTGTTGATTTAGCT
ATTTTTTCTTTACACTTAGCAGGAATTTCTTCAATTTTAGGAGCTGTAAATTTTATTACT
ACAGTTATTAATATACGATCAACAGGAATTACATTCGACCGAATACCATTATTTGTTTGA
TCTGTAGTAATTACAGCTTTATTACTTTTATTATCTTTACCAGTATTAGCAGGTGCTATT
ACTATATTATTAACAGATCGAAATCTTAATACATCATTCTTTGACCCAGCAGGAGGAGGA
GACCCAATCTTGTACCAACATTTATTTAATAATTAATAATTCCATATGTAGAAATATAAGAGTATAAATCATATTG

AACCTAAAAAGTAAGAAGAATTATAATTATTTAAAGCCTTATTATAAAAAAATAAAGAAA

TATTAGAAATTAAATAACCTCTAACTCCTCCTACAATACAAACAAATAAAGTTAATAATT

TTAAATAAGAAGGAAGAACTACTACTACTGGTGTAGGAAAAATTAATCAACTTAATATAC

TACCCCCAAAAATTCTCAAAATTAATAGTCCTATTATACTTTTTAATATAACTCAACCTT

CATCATTTAGTATATTTAAAGAAGAAAAATTTGAATCCCCA

GTTGTCTTCGCTCCCGCTAACATCACCACTGAAATCAAG

TCCGTAGAAATGCACCACGAAGCTCTCGTTGAAGCTGTTCCCGGTGACAACGTTGGTTTC

AACGTTAAGAACGTCTCCGTCAAGGAATTGCGTCGTGGTTACGTCGCTGGTGACTCCAAG

GCTAGCCCCCCCAAGGGTGCTGCTGACTTCACCGCTCAAGTCATCGTCTTGAACCACCCT

GGTCAAATCTCCAACGGTTACACTCCCGTTTTGGATTGTCACACCGCTCACATTGCTTGC

AAATTCGCCGAAATCAAGGAGAAGGTCGAT

>ITTVLc2

AGAATTTTAATTCGAGCTGAATTAGGACACC
CTGGTGCATTAATTGGAGATGACCAAATTTATAATGTAATTGTTACAGCTCATGCTTTTA
TTATAATTTTCTTTATAGTAATACCAATTATAATTGGAGGATTTGGAAATTGATTAGTTC
CTTTAATATTAGGAGCCCCAGATATAGCATTCCCTCGAATAAATAATATAAGTTTTTGAC
TTTTACCTCCTGCATTAACTTTATTATTAGTAAGTAGTATAGTAGAAAACGGAGCTGGAA
CAGGATGAACAGTTTACCCTCCTTTATCATCTAATATCGCTCATGGAGGAGCTTCTGTTG
ATTTAGCTATTTTTTCTCTTCATTTAGCAGGAATTTCATCAATTTTAGGAGCTGTAAATT
TTATTACTACAGTTATTAATATACGATCAACAGGAATTACTTTTGACCGAATACCTTTAT
TCGTTTGATCAGTAGTAATTACAGCTTTATTACTTTTATTATCATTACCAGTATTAGCTG
GAGCTATTACTATACTTTTAACTGATCGAAATCTTAATACTTCATTCTTTGATCCAGCAG
GAGGAGGAGACCCAATTTTATACCAACATTTATTTAATAATTAATAATCCCGTAAGTAGAAATATAAGGTATAAACCATAT

TGACCCTAAAAAATAAGAAAAATTATAATTATTTAAAGCCTTATTATAAAAAAATAAAGA

AATATTTGAAATTATATACCCTCTTACTCCTCCTACAATACAAACAAATAATGTTAATAA

CTTCAAATATGAAGGAAGAACTACAACTACTGGAGTTGGAAAAATTAATCATCTTAATAT

TCTTCCTCCAAAAATTCTTAAAATTAATAACCCTATTATACTCTTTAATATTACTCAACC

TTCATCATTTAATATATTTAAAGAAGAAAAATTTGAATCACCAGTTGTCATCGCTCCCGCTAACATCACCACTGAAGTCAAGTCCGTTGAAATGCACCACGAA

GCTCTCGTTGAAGCCGTCCCCGGTGACAACGTTGGTTTCAACGTAAAGAACGTCTCCGTC

AAGGAATTGCGTCGTGGTTACGTCGCTGGTGACTCCAAGGCCAACCCCCCCAAGGGTGCC

GCTGACTTCACCGCTCAAGTCATCGTCTTGAACCATCCCGGTCAAATCTCCAACGGTTAC

ACTCCCGTTTTGGATTGTCACACCGCTCACATTGCTTGCAAATTCGCCGAAATCAAGGAG

AAGGTCGAT

>ITTVCvo1

AGAATTTTAATTCGAGCTGAACTAGGGCA
TCCTGGAGCATTAATTGGAGATGATCAAATTTATAATGTAATTGTTACAGCTCATGCTTT
TATTATAATTTTTTTTATAGTAATACCAATTATAATTGGAGGGTTTGGAAATTGATTAGT
TCCTTTAATATTAGGAGCCCCAGATATAGCATTCCCTCGAATAAATAATATAAGTTTCTG
ACTTTTACCTCCTGCATTAACTTTACTATTAGTAAGTAGTATAGTAGAAAACGGAGCTGG
AACTGGATGAACTGTTTATCCACCTTTATCTTCTAATATTGCACATGGAGGAGCTTCTGT
TGATTTAGCTATTTTTTCTTTACATTTAGCAGGAATTTCTTCAATTTTAGGAGCTGTAAA
TTTTATTACTACAGTTATTAATATACGATCAACAGGTATTACCTTCGACCGAATACCATT
ATTTGTTTGATCAGTAGTAATTACAGCCTTATTACTTTTATTATCTTTACCAGTATTAGC
AGGAGCTATTACTATATTATTAACAGATCGAAATCTTAATACTTCATTCTTTGACCCAGC
AGGAGGAGGAGATCCAATTTTATACCAACACTTATTTAGTAATTAATAATTCCGTATGTAGAAATATAAGAGCATAAACCACATTGA

CCCTAAAAAATAAGAAGAATTATAGTTATTTAAAGCCTTATTATAAAAAAATAAAGAAAT

ATTAGAAATTAAATACCCTCTAATTCCTCCTACAATACAAACAAATAAAGTTAATAACTT

TAAATAAGAAGGTAGAACTACTACTACTGGTGTAGGAAAAATTAATCAACTTAACATTCT

ACCCCCAAAAATTCTTAAAATTAATAACCCTATTATACTCTTTAACATAACTCAACCTTC

ATCATTTAATATATTTAAAGAAGAAAAATTTGAATCCCCAGTTGTCATCGCTCCCGCTAACATCACCACTGAAGTCAAATC

CGTAGAAATGCACCACGAAGCTCTCGTTGAAGCCGTCCCCGGTGACAACGTTGGTTTCAA

CGTTAAGAACGTCTCCGTCAAGGAATTGCGTCGTGGTTACGTCGCTGGTGACTCCAAGGC

TAGCCCCCCCAAGGGTGCTGCTGACTTCACCGCTCAAGTCATCGTCTTGAACCACCCTGG

TCAAATCTCCAACGGTTACACTCCCGTTTTGGATTGTCACACCGCTCACATTGCTTGCAA

ATTCGCCGAAATCAAGGAGAAGGTCGAT

>DataPrte1

AGAATCCTAATTCGAGCT

GAATTAGGGCACCCTGGAGCACTAATTGGAGATGACCAAATTTATAATGTAATTGTAACGGCTCACGCTT

TTATTATAATTTTCTTTATAGTAATACCAATTATAATTGGAGGATTTGGAAATTGACTAGTTCCCCTTAT

ATTAGGGGCTCCTGATATAGCATTCCCTCGAATAAATAATATAAGTTTCTGACTTTTACCTCCTGCATTA

ACTTTATTATTAGTAAGTAGTATAGTAGAAAATGGGGCTGGAACAGGATGAACTGTTTACCCACCTTTAT

CTTCTAATATTGCTCACGGAGGAGCATCTGTTGATTTAGCTATTTTCTCTCTTCACTTGGCCGGAATTTC

TTCAATTTTAGGAGCTGTAAATTTCATTACAACTGTAATTAATATACGATCTACAGGAATTACATTTGAT

CGAATACCTTTATTTGTTTGATCTGTAGTTATTACTGCTCTTTTACTTTTATTATCTTTACCAGTATTAG

CAGGTGCTATTACTATATTATTAACTGACCGAAATTTAAATACTTCATTTTTTGATCCAGCTGGAGGAGG

AGACCCTATTTTATACCAACATTTATTT AATAATT

AATAATTCCATAAGTAGAAATATAAGGTATAAATCACATTGAACCTAAAAAATAAGAAGAATGATAATTA

TTTAAAGCCTTGTTATAAAAAAATAAAGAAACATTAGAAATTATATACCCTACTAACCCTCCAACAATAC

AAACAAATAATGTTAATAACTTTAAATAAAAAGGTAAAACTACTACTATAGGAGTAGGAAAAATTAACCA

TCTTAACATTCTACCTCCAAAAATTCTTAAAATTAATAAACCTATTATACTCTTTAATATTACTCAACCT

TCATCATTTAACATATTTAAAGAAGAAAAATTAGAATCCCCAGTTGTCTTCGCTCCCGCTAACATCACCACTGAAGTCAAGTCCGTTGAAATGCACCACGAAGCTTTGGCTGAAGCCGTGCCCGGTGACAACGTTGGTTTCAACGTAAAGAACGTCTCCGTCAAGGAATTGCGTCGTGGTTACGTCGCTGGTGACTCCAAGGCCAACCCCCCCAAGGGTGCTGCTGACTTCACCGCTCAAGTCATCGTCTTGAACCACCCCGGTCAAATCTCCAACGGTTACACCCCCGTTTTGGATTGTCACACCGCTCACATTGCTTGCAAATTCGCTGAAATCAAGGAGAAGGTCGAC

>DataLse1

AGAATTCTAATTCGAGCT

GAATTAGGACATCCTGGAGCTTTAATTGGAGATGATCAAATTTATAATGTAATTGTTACAGCTCATGCTT

TTATTATAATTTTTTTTATAGTAATGCCAATTATAATTGGAGGATTTGGAAATTGATTAGTTCCATTAAT

ACTAGGAGCTCCAGATATAGCATTCCCTCGAATAAATAATATAAGTTTTTGACTTTTACCTCCTGCATTA

ACTTTATTATTAGTTAGTAGTATAGTAGAAAACGGAGCTGGAACAGGATGAACAGTTTACCCTCCTCTAT

CTTCTAATATTGCTCATGGAGGAGCTTCTGTTGATTTAGCTATTTTCTCTCTTCATTTAGCAGGAATTTC

TTCAATTTTAGGAGCTGTAAATTTTATTACTACAGTTATTAATATACGATCAACAGGAATTACTTTTGAT

CGAATACCTTTATTTGTTTGATCAGTAGTAATTACAGCTTTATTACTTTTATTATCATTACCAGTATTAG

CAGGAGCTATTACAATACTTTTAACAGACCGAAATCTTAATACATCATTCTTTGACCCTGCAGGAGGAGG

AGATCCAATTTTATACCAACATTTATTTAATAATTAATAATTCCATAAGTAGAAATATAAGGTATAAACCACATAGAACCTAAAA

AATAAGAAAAATTATAATTATTTAAAGCCTTATTATAAAAAAATAAAGAAACATGAGAAATTATATAACC

TATAATACCCCCAACAATACAAACAAATAATGTTAATAATTTTAAATAAGAAGGAAGAACAACTACTATA

GGAGTAGGAAAAATCAATCATCTTAACATTCTCCCACCAAAAATTCTTAAAATTAATAAACCTATTATAC

TTTTCAATATTACTCAACCTTCATCATTTAATATATTTAAAGAAGAAAAATTAGAATCACCA GTTGTCTTCGCYCCCGCTAACATCACCACTGAAGTCAAGTCCGTTGAAATGCACCACGAAGCTCTCGTTGAAGCCGTCCCCGGTGACAACGTTGGTTTCAACGTAAAGAACGTCTCCGTCAAGGAATTGCGTCGTGGTTACGTCGCTGGTGACTCCAAGGCYAACCCCCCCAAGGGCGCCGCTGACTTCACCGCTCAAGTCATCGTCTTGAACCACCCTGGTCAAATCTCCAACGGTTACACCCCCGTTTTGGATTGTCACACCGCTCACATTGCTTGCAAATTCGCCGAAATCAAGGAGAAGGTCGAT

>DataLill1

AGAATTTTAATTCGAGCT

GAATTAGGACACCCTGGTGCATTAATTGGAGATGACCAAATTTATAATGTAATTGTTACAGCTCATGCTT

TTATTATAATTTTCTTTATAGTAATGCCAATTATAATTGGAGGATTTGGAAATTGATTAGTTCCTTTAAT

ATTAGGAGCTCCAGATATAGCATTCCCTCGAATAAATAATATAAGATTTTGACTTTTACCTCCTGCATTA

ACTTTACTATTAGTAAGTAGTATAGTAGAAAACGGAGCTGGAACAGGATGAACAGTTTACCCTCCTTTAT

CATCTAATATCGCTCATGGAGGAGCTTCTGTTGATTTAGCTATTTTTTCTCTTCATTTAGCAGGAATTTC

ATCAATTTTAGGAGCTGTAAATTTTATTACTACAGTTATTAATATACGATCAACAGGGATTACTTTTGAT

CGAATGCCTTTATTCGTTTGATCAGTAGTAATTACAGCTTTATTACTTTTATTATCATTACCAGTATTAG

CTGGAGCTATTACTATACTTTTAACTGATCGAAATCTTAATACTTCATTCTTTGACCCAGCAGGAGGAGG

AGACCCAATTTTATACCAACATTTATTTAATAATTAATAATTCCGTAAGTAGAAATATAAGGTATAAACCACATTGACCCTAAAAAATAAGAAAAATTATAATTATTTAAAGCCTTATTATAAAAAAATAAGGAAATATTTGAAATTATATACCCTCTTACTCCTCCTACAATACAAACAAATAATGTTAATAACTTTAAATATGAAGGAAGAACTACAACTACTGGAGTTGGAAAAATTAATCATCTTAATATTCTTCCTCCAAAAATTCTTAAAATTAATAATCCTATTATACTTTTTAATATTACTCAACCTTCATCATTTAATATATTTAAAGAAGAAAAATTTGAATCACCAGTTGTCTTCGCTCCCGCTAACATCACCACTGAAGTCAAGTCCGTTGAAATGCACCACGAAGCTCTCGTTGAAGCCGTCCCCGGTGACAACGTTGGTTTCAACGTAAAGAACGTCTCCGTCAAGGAATTGCGTCGTGGTTACGTCGCTGGTGACTCCAAGGCCAACCCCCCCAAGGGTGCCGCTGACTTCACCGCTCAAGTCATCGTCTTGAACCATCCCGGTCAAATCTCCAACGGTTACACTCCCGTTTTGGATTGTCACACCGCTCACATTGCTTGCAAATTCGCCGAAATCAAGGAGAAGGTCGAT

**COI+ND5+EF1a+PER sequences**

>ITMOII10Ls
AGAATTCTAATTCGAGCTGAATTAGGACATCCTGGAG
CTTTAATTGGAGATGATCAAATTTATAATGTAATTGTTACAGCTCATGCTTTTATTATAA
TTTTTTTTATAGTAATGCCAATTATAATTGGAGGATTTGGAAATTGATTAGTTCCATTAA
TACTAGGAGCTCCAGATATAGCATTCCCTCGAATAAATAATATAAGTTTTTGACTTTTAC
CTCCTGCATTAACTTTATTATTAGTTAGTAGTATAGTAGAAAACGGAGCTGGAACAGGAT
GAACAGTTTACCCTCCTCTATCTTCTAATATTGCTCATGGAGGAGCTTCTGTTGATTTAG
CTATTTTCTCTCTTCATTTAGCAGGAATTTCTTCAATTTTAGGAGCTGTAAATTTTATTA
CTACAGTTATTAATATACGATCAACAGGAATTACTTTTGATCGAATACCTTTATTTGTTT
GATCAGTAGTAATTACAGCTTTATTACTTTTATTATCATTACCAGTATTAGCAGGAGCTA
TTACAATACTTTTAACAGACCGAAATCTTAATACATCATTCTTTGATCCTGCAGGAGGAG
GAGATCCAATTTTATACCAACATTTATTT

GATAATTAATAATTCCATAAGTAGAAATATAAGGTATAAACCACATAGAACCTA
AAAAATAAGAAAAATTATAATTATTTAAAGCCTTATTATAAAAAAATAAAGAAACATGAG
AAATTATATAACCTATAATACCCCCAACAATACAAACAAATAATGTTAATAATTTTAAAT
AAGAAGGAAGAACAACTACTATAGGAGTAGGAAAAATTAATCATCTTAACATTCTCCCAC
CAAAAATTCTTAAAATTAATAAACCTATTATACTTTTCAATATTACTCAACCTTCATCAT
TTAATATATTTAAAGAAGAAAAATTAGAATCACCA

GTTGTCTTC
GCTCCCGCTAACATCACCACTGAAGTCAAGTCCGTTGAAATGCACCACGAAGCTCTCGTT
GAAGCCGTCCCCGGTGACAACGTTGGTTTCAACGTAAAGAACGTCTCCGTCAAGGAATTG
CGTCGTGGTTACGTCGCTGGTGACTCCAAGGCTAACCCCCCCAAGGGCGCCGCTGACTTC
ACCGCTCAAGTCATCGTCTTGAACCACCCTGGTCAAATCTCCAACGGTTACACCCCCGTT
TTGGATTGTCACACCGCTCACATTGCTTGCAAATTCGCCGAAATCAAGGAGAAGGTCGATGGATGCACGCA
GTACCCTAAGTCCGGTTCAGTGCTTTGAGGGCAGCGGTGGAAGTGGTTCATCGGGAAATT
TTACTTCGGGCAGTAACTTAAATATGGGCAGTGTGACTAATACCAGCAACACCGGTACTG
GTACTTCCTCAGGAAGTGTTCCCATGGTCACGTTGACGGAATCTCTACTAAATAAACATA
ACGATGAAATGGAAAAGTTTATGCTTAAGAAGCATAGAGAATCTCGAGGACGAAGTGGCG
AGAAGAACAAAAAGAATTCTGAAAAGATAATGGAGTACACTGGCCCGGGACACGGAATCA
AAAGAGTTGGTTCACA
>ITMOII1Cvi
AGAATTCTAATTCGAGCCGAACTAGGACATCCTGG
AGCATTAATTGGAGATGACCAAATTTATAATGTAATTGTTACAGCTCATGCTTTTATTAT
AATTTTTTTTATAGTAATACCAATTATAATTGGAGGATTTGGTAATTGATTAGTCCCTTT
AATATTAGGAGCTCCAGATATAGCCTTCCCTCGGATAAACAATATAAGTTTCTGACTTTT
ACCTCCTGCATTAACTTTACTATTAGTAAGTAGTATAGTAGAAAACGGAGCTGGAACAGG
ATGAACTGTTTACCCACCTTTATCTTCTAATATTGCTCATGGAGGAGCTTCTGTTGATTT
AGCTATTTTTTCTTTACACTTAGCAGGAATTTCTTCAATTTTAGGAGCTGTAAATTTTAT
TACTACAGTTATTAATATACGATCAACAGGAATTACATTCGACCGAATACCATTATTTGT
TTGATCTGTAGTAATTACAGCTTTATTACTTTTATTATCTTTACCAGTATTAGCAGGTGC
TATTACTATATTATTAACAGATCGAAATCTTAATACATCATTCTTTGACCCAGCAGGAGG
AGGAGACCCAATCTTGTACCAACATTTATTT

AATAATTAATAATTCCATATGTAGAAATATAAGGTATAAATCAT
ATTGAACCTAAAAAGTAAGAAGAATTATAATTATTTAAAGCCTTATTATAAAAAAATAAA
GAAATATTAGAAATTAAATAACCTCTAACTCCTCCTACAATACAAACAAATAAAGTTAAT
AATTTTAAATAAGAAGGAAGAACTACTACTACTGGTGTAGGAAAAATTAATCAACTTAAT
ATACTACCCCCAAAAATTCTCAAAATTAATAGTCCTATTATACTTTTTAATATAACTCAA
CCTTCATCATTTAGTATATTTAAAGAAGAAAAATTTGAATCCCCAGTTGTCTTCGCTCCCGCTAACATCACCACTGAAGTCAAG
TCCGTAGAAATGCACCACGAAGCTCTCGTTGAAGCTGTTCCCGGTGACAACGTTGGTTTC
AACGTTAAGAACGTCTCCGTCAAGGAATTGCGTCGTGGTTACGTCGCTGGTGACTCCAAG
GCTAGCCCCCCCAAGGGTGCTGCTGACTTCACCGCTCAAGTCATCGTCTTGAACCACCCT
GGTCAAATCTCCAACGGTTACACTCCCGTTTTGGATTGTCACACCGCTCACATTGCTTGC
AAATTCGCCGAAATCAAGGAGAAGGTCGATTGAAGCACGCAGTACCCTGAGTCCGGTTCAGTGCTTTGAGGGTAGCGGTGGTAG
TGGATCCTCAGGAAATTTTACTTCGGGTAGCAACTTGAATATGGGCAGTGTTACTAATAC
AAGCAATACCGGTACAGGCACTTCATCGGGAAGTGTACCCATGGTTACGTTGACGGAATC
TCTGCTAAATAAACACAACGATGAAATGGAAAAGTTTATGCTAAAGAAACACAGAGAATC
TCGCGGACGAAGTGGTGAGAAGAATAAAAAGAACTCGGAAAAGATAATGGAGTATACTGG
TCCTGGGCATGGAATAAAGAGAGTTGGCTCACA

>ITMOII2Cvi
AGAATTCTAATTCGAGCCGAACTAGGACATCCTGG
AGCATTAATTGGAGATGACCAAATTTATAATGTAATTGTTACAGCTCATGCTTTTATTAT
AATTTTTTTTATAGTAATACCAATTATAATTGGAGGATTTGGTAATTGATTAGTCCCTTT
AATATTAGGAGCTCCAGATATAGCCTTCCCTCGGATAAACAATATAAGTTTCTGACTTTT
ACCTCCTGCATTAACTTTACTATTAGTAAGTAGTATAGTAGAAAACGGAGCTGGAACAGG
ATGAACTGTTTACCCACCTTTATCTTCTAATATTGCTCATGGAGGAGCTTCTGTTGATTT
AGCTATTTTTTCTTTACACTTAGCAGGAATTTCTTCAATTTTAGGAGCTGTAAATTTTAT
TACTACAGTTATTAATATACGATCAACAGGAATTACATTCGACCGAATACCATTATTTGT
TTGATCTGTAGTAATTACAGCTTTATTACTTTTATTATCTTTACCAGTATTAGCAGGTGC
TATTACTATATTATTAACAGATCGAAATCTTAATACATCATTCTTTGACCCAGCAGGAGG
AGGAGACCCAATCTTGTACCAACATTTATTTAATAATTAATAATTCCATATGTAGAAATATAAGGTATAAATCATAT
TGAACCTAAAAAGTAAGAAGAATTATAATTATTTAAAGCCTTATTATAAAAAAATAAAGA
AATATTAGAAATTAAATAACCTCTAACTCCTCCTACAATACAAACAAATAAAGTTAATAA
TTTTAAATAAGAAGGAAGAACTACTACTACTGGTGTAGGAAAAATTAATCAACTTAATAT
ACTACCCCCAAAAATTCTCAAAATTAATAGTCCTATTATACTTTTTAATATAACTCAACC
TTCATCATTTAGTATATTTAAAGAAGAAAAATTTGAATCCCCAGTTGTCTTCGCTCCCGCTAACATCACCACTGAAGTCAAG
TCCGTAGAAATGCACCACGAAGCTCTCGTTGAAGCTGTTCCCGGTGACAACGTTGGTTTC
AACGTTAAGAACGTCTCCGTCAAGGAATTGCGTCGTGGTTACGTCGCTGGTGACTCCAAG
GCTAGCCCCCCCAAGGGTGCTGCTGACTTCACCGCTCAAGTCATCGTCTTGAACCACCCT
GGTCAAATCTCCAACGGTTACACTCCCGTTTTGGATTGTCACACCGCTCACATTGCTTGC
AAATTCGCCGAAATCAAGGAGAAGGTCGATTGAAGCACGCAGTACCCTGAGTCCGGTTCAGTGCTTTGAGGGTAGCGGTGGTAGTGGAT
CCTCGGGAAATTTTACTTCGGGTAGCAACTTGAATATGGGCAGTGTTACTAATACAAGCA
ATACCGGTACAGGCACTTCATCGGGAAGTGTACCCATGGTTACGTTGACGGAATCTCTAC
TAAATAAACACAACGATGAAATGGAAAAGTTTATGCTAAAGAAACACAGAGAATCTCGCG
GACGAAGTGGTGAGAAGAATAAAAAGAACTCGGAAAAGATAATGGAGTATACTGGTCCTG
GGCATGGAATAAAGAGAGTTGGCTCACA

>ITMOII3Cvi
AGAATTCTAATTCGAGCCGAACTAGGACATCCTGGAGCATTAATTGGAGATGACCAAATTTATAATGTAATTGTTACAGCTCATGCTTTTATTATAATTTTTTTTATAGTAATACCAATTATAATTGGAGGATTTGGTAATTGATTAGTCCCTT

TAATATTAGGAGCTCCAGATATAGCCTTCCCTCGGATAAACAATATAAGTTTCTGACTTT

TACCTCCTGCATTAACTTTACTATTAGTAAGTAGTATAGTAGAAAACGGAGCTGGAACAG

GATGAACTGTTTACCCACCTTTATCTTCTAATATCGCTCATGGAGGAGCTTCTGTTGATT

TAGCTATTTTTTCTTTACACTTAGCAGGAATTTCTTCAATTTTAGGAGCTGTAAATTTTA

TTACTACAGTTATTAATATACGATCAACAGGAATTACATTCGACCGAATACCATTATTTG

TTTGATCTGTAGTAATTACAGCTTTATTACTTTTATTATCTTTACCAGTATTAGCAGGTG

CTATTACTATATTATTAACAGATCGAAATCTTAATACATCATTCTTTGACCCAGCAGGAG

GAGGAGACCCAATCTTGTACCAACATTTATTTAATAATTGATAATTCCATATGTAGAAATATAAGGTATAAATCATATT
GAACCTAAAAAGTAAGAAGAATTATAATTATTTAAAGCCTTATTATAAAAAAATAAAGAA
ATATTAGAAATTAAATAACCTCTAACTCCTCCTACAATACAAACAAATAAAGTTAATAAT
TTTAAATAAGAAGGAAGAACTACTACTACTGGTGTAGGAAAAATTAATCAACTTAATATA
CTACCCCCAAAAATTCTCAAAATTAATAGTCCTATTATACTTTTTAATATAACTCAACCT
TCATCATTTAGTATATTTAAAGAAGAAAAATTTGAATCCCCAGTTGTCTTCGCTCCCGCTAACATCACCACTGAAGT
CAAGTCCGTAGAAATGCACCACGAAGCTCTCGTTGAAGCTGTTCCCGGTGACAACGTTGG
TTTCAACGTTAAGAACGTCTCCGTCAAGGAATTGCGTCGTGGTTACGTCGCTGGTGACTC
CAAGGCTAGCCCCCCCAAGGGTGCTGCTGACTTCACCGCTCAAGTCATCGTCTTGAACCA
CCCTGGTCAAATCTCCAACGGTTACACTCCCGTTTTGGATTGTCACACCGCTCACATTGC
TTGCAAATTCGCCGAAATCAAGGAGAAGGTCGATTGAAGCACGCAGTACCCTGAGTCCGGTTCAGTGCTTTGAGGGTAGCGGTGGTAGT
GGATCCTCAGGAAATTTTACTTCGGGTAGCAACTTGAATATGGGCAGTGTTACTAATACA
AGCAATACCGGTACAGGCACTTCATCGGGAAGTGTACCCATGGTTACGTTGACGGAATCT
CTACTAAATAAACACAACGATGAAATGGAAAAGTTTATGCTAAAGAAACACAGAGAATCT
CGCGGACGAAGTGGTGAGAAGAATAAAAAGAACTCGGAAAAGATAATGGAGTATACTGGT
CCTGGGCATGGAATAAAGAGAGTTGGCTCACA

>ITMOII4Cvi
AGAATTCTAATTCGAGCCGAACTAGGACATCCTGGAG
CATTAATTGGAGATGACCAAATTTATAATGTAATTGTTACAGCTCATGCTTTTATTATAA
TTTTTTTTATAGTAATACCAATTATAATTGGAGGATTTGGTAATTGATTAGTCCCTTTAA
TATTAGGAGCTCCAGATATAGCCTTCCCTCGGATAAACAATATAAGTTTCTGACTTTTAC
CTCCTGCATTAACTTTACTATTAGTAAGTAGTATAGTAGAAAACGGAGCTGGAACAGGAT
GAACTGTTTACCCACCTTTATCTTCTAATATCGCTCATGGAGGAGCTTCTGTTGATTTAG
CTATTTTTTCTTTACACTTAGCAGGAATTTCTTCAATTTTAGGAGCTGTAAATTTTATTA
CTACAGTTATTAATATACGATCAACAGGAATTACATTCGACCGAATACCATTATTTGTTT
GATCTGTAGTAATTACAGCTTTATTACTTTTATTATCTTTACCAGTATTAGCAGGTGCTA
TTACTATATTATTAACAGATCGAAATCTTAATACATCATTCTTTGACCCAGCAGGAGGAG
GAGACCCAATCTTGTACCAACATTTATTTAATAATTAATAATTCCATATGTAGAAATATAAGGTATAAATCATATTGAAC
CTAAAAAGTAAGAAGAATTATAATTATTTAAAGCCTTATTATAAAAAAATAAAGAAATAT
TAGAAATTAAATAACCTCTAACTCCTCCTACAATACAAACAAATAAAGTTAATAATTTTA
AATAAGAAGGAAGAACTACTACTACTGGTGTAGGAAAAATTAATCAACTTAATATACTAC
CCCCAAAAATTCTCAAAATTAATAGTCCTATTATACTTTTTAATATAACTCAACCTTCAT
CATTTAGTATATTTAAAGAAGAAAAATTTGAATCCCCAGTTGTCTTCGCTCCCGCTAACATCACCACTGAAG
TCAAGTCCGTAGAAATGCACCACGAAGCTCTCGTTGAAGCTGTTCCCGGTGACAACGTTG
GTTTCAACGTTAAGAACGTCTCCGTCAAGGAATTGCGTCGTGGTTACGTCGCTGGTGACT
CCAAGGCTAGCCCCCCCAAGGGTGCTGCTGACTTCACCGCTCAAGTCATCGTCTTGAACC
ACCCTGGTCAAATCTCCAACGGTTACACTCCCGTTTTGGATTGTCACACCGCTCACATTG
CTTGCAAATTCGCCGAAATCAAGGAGAAGGTCGATTGAAGCACGCAGTACCCTGAGTCCGGTTCAGTGCTTTGAGGGTAGCGGTGGTAGTG
GATCCTCAGGAAATTTTACTTCGGGTAGCAACTTGAATATGGGCAGTGTTACTAATACAA
GCAATACCGGTACAGGCACTTCATCGGGAAGTGTACCCATGGTTACGTTGACGGAATCTC
TGCTAAATAAACACAACGATGAAATGGAAAAGTTTATGCTAAAGAAACACAGAGAATCTC
GCGGACGAAGTGGTGAGAAGAATAAAAAGAACTCGGAAAAGATAATGGAGTATACTGGTC
CTGGGCATGGAATAAAGAGAGTTGGCTCACA

>ITMOII5Cvi
AGAATTCTAATTCGAGCCGAACTAGGACATC
CTGGAGCATTAATTGGAGATGACCAAATTTATAATGTAATTGTTACAGCTCATGCTTTTA
TTATAATTTTTTTTATAGTAATACCAATTATAATTGGAGGATTTGGTAATTGATTAGTCC
CTTTAATATTAGGAGCTCCAGATATAGCCTTCCCTCGGATAAACAATATAAGTTTCTGAC
TTTTACCTCCTGCATTAACTTTACTATTAGTAAGTAGTATAGTAGAAAACGGAGCTGGAA
CAGGATGAACTGTTTACCCACCTTTATCTTCTAATATTGCTCATGGAGGAGCTTCTGTTG
ATTTAGCTATTTTTTCTTTACACTTAGCAGGAATTTCTTCAATTTTAGGAGCTGTAAATT
TTATTACTACAGTTATTAATATACGATCAACAGGAATTACATTCGACCGAATACCATTAT
TTGTTTGATCTGTAGTAATTACAGCTTTATTACTTTTATTATCTTTACCAGTATTAGCAG
GTGCTATTACTATATTATTAACAGATCGAAATCTTAATACATCATTCTTTGACCCAGCAG
GAGGAGGAGACCCAATCTTGTACCAACATTTATTTAATAATTAATAATTCCATATGTAGAAATATAAGGTATAAATCATATTGAAC
CTAAAAAATAAGAAGAATTATAATTATTTAAAGCCTTATTATAAAAAAATAAAGAAATAT
TAGAAATTAAATAACCTCTAACTCCTCCTACAATACAAACAAATAAAGTTAATAATTTTA
AATAAGAAGGAAGAACTACTACTACTGGTGTAGGAAAAATTAATCAACTTAATATACTAC
CCCCAAAAATTCTCAAAATTAATAGTCCTATTATACTTTTTAATATAACTCAACCTTCAT
CATTTAGTATATTTAAAGAAGAAAAATTTGAATCCCCAGTTGTCTTCGCTCCCGCTAACATCACCACTGAAGT
CAAGTCCGTAGAAATGCACCACGAAGCTCTCGTTGAAGCTGTTCCCGGTGACAACGTTGG
TTTCAACGTTAAGAACGTCTCCGTCAAGGAATTGCGTCGTGGTTACGTCGCTGGTGACTC
CAAGGCTAGCCCCCCCAAGGGTGCTGCTGACTTCACCGCTCAAGTCATCGTCTTGAACCA
CCCTGGTCAAATCTCCAACGGTTACACTCCCGTTTTGGATTGTCACACCGCTCACATTGC
TTGCAAATTCGCCGAAATCAAGGAGAAGGTCGATTGAAGCACGCAGTACCCTGAGTCCGGTTCAGTGCTTTGAGGGTAGCGGTGGTAGTGG
ATCCTCGGGAAATTTTACTTCGGGTAGCAACTTGAATATGGGCAGTGTTACTAATACAAG
CAATACCGGTACAGGCACTTCATCGGGAAGTGTACCCATGGTTACGTTGACGGAATCTCT
ACTAAATAAACACAACGATGAAATGGAAAAGTTTATGCTAAAGAAACACAGAGAATCTCG
CGGACGAAGTGGTGAGAAGAATAAAAAGAACTCGGAAAAGATAATGGAGTATACTGGTCC
TGGGCATGGAATAAAGAGAGTTGGCTCACA

>ITMOII6Ls
AGAATTCTAATTCGAGCTGAATTAGGACATCCT
GGAGCTTTAATTGGAGATGATCAAATTTATAATGTAATTGTTACAGCTCATGCTTTTATT
ATAATTTTTTTTATAGTAATGCCAATTATAATTGGAGGATTTGGAAATTGATTAGTTCCA
TTAATACTAGGAGCTCCAGATATAGCATTCCCTCGAATAAATAATATAAGTTTTTGACTT
TTACCTCCTGCATTAACTTTATTATTAGTTAGTAGTATAGTAGAAAACGGAGCTGGAACA
GGATGAACAGTTTACCCTCCTCTATCTTCTAATATTGCTCATGGAGGAGCTTCTGTTGAT
TTAGCTATTTTCTCTCTTCATTTAGCAGGAATTTCTTCAATTTTAGGAGCTGTAAATTTT
ATTACTACAGTTATTAATATACGATCAACAGGAATTACTTTTGATCGAATACCTTTATTT
GTTTGATCAGTAGTAATTACAGCTTTATTACTTTTATTATCATTACCAGTATTAGCAGGA
GCTATTACAATACTTTTAACAGACCGAAATCTTAATACATCATTCTTTGACCCTGCAGGA
GGAGGAGATCCAATTTTATACCAACATTTATTTAATAATTAATAATTCCATAAGTAGAAATATAAGGTATAAACCAC
ATAGAACCTAAAAAATAAGAAAAATTATAATTATTTAAAGCCTTGTTATAAAAAAATAAA
GAAACATTAGAAATTATATAACCTATAATACCCCCAACAATACAAACAAATAATGTTAAT
AATTTTAAATAAGAAGGAAGAACAATTACTATAGGAGTAGGAAAAATCAATCATCTTAAC
ATTCTCCCACCAAAAATTCTTAAAATTAATAAACCTATTATACTTTTCAATATTACTCAA
CCTTCATCATTTAATATATTTAAAGAAGAAAAATTAGAATCACCAGTTGTCTTCGCTCCCGCTAACATCACCACTGAAGTCAAG
TCCGTTGAAATGCACCACGAAGCTCTCGTTGAAGCCGTCCCCGGTGACAACGTTGGTTTC
AACGTAAAGAACGTCTCCGTCAAGGAATTGCGTCGTGGTTACGTCGCTGGTGACTCCAAG
GCTAACCCCCCCAAGGGCGCCGCTGACTTCACCGCTCAAGTCATCGTCTTGAACCACCCT
GGTCAAATCTCCAACGGTTACACCCCCGTTTTGGATTGTCACACCGCTCACATTGCTTGC
AAATTCGCCGAAATCAAGGAGAAGGTCGATCGATGCACGCAGTACCCTAAGTCCGGTTCAGTGCTTTGAGGGCAGCGGTGGAAGT
GGTTCATCGGGAAATTTTACTTCGGGCAGTAACTTAAATATGGGCAGTGTGACTAATACC
AGCAACACCGGTACTGGTACTTCCTCTGGAAGTGTTCCCATGGTCACGTTGACCGAATCT
CTACTAAATAAACATAACGATGAAATGGAAAAGTTTATGCTTAAGAAGCATAGAGAATCT
CGAGGACGAAGTGGCGAGAAGAACAAAAAGAATTCTGAAAAGATAATGGAGTACACTGGC
CCGGGACACGGAATCAAAAGAGTTGGTTCACA

>ITMOII7Ls
AGAATTCTAATTCGAGCTGAATTAGGA
CATCCTGGAGCTTTAATTGGAGATGATCAAATTTATAATGTAATTGTTACAGCTCATGCT
TTTATTATAATTTTTTTTATAGTAATGCCAATTATAATTGGAGGATTTGGAAATTGATTA
GTTCCATTAATACTAGGAGCTCCAGATATAGCATTCCCTCGAATAAATAATATAAGTTTT
TGACTTTTACCTCCTGCATTAACTTTATTATTAGTTAGTAGTATAGTAGAAAACGGAGCT
GGAACAGGATGAACAGTTTACCCTCCTCTATCTTCTAATATTGCTCATGGAGGAGCTTCT
GTTGATTTAGCTATTTTCTCTCTTCATTTAGCAGGAATTTCTTCAATTTTAGGAGCTGTA
AATTTTATTACTACAGTTATTAATATACGATCAACAGGAATTACTTTTGATCGAATACCT
TTATTTGTTTGATCAGTAGTAATTACAGCTTTATTACTTTTATTATCATTACCAGTATTA
GCAGGAGCTATTACAATACTTTTAACAGACCGAAATCTTAATACATCATTCTTTGACCCT
GCAGGAGGAGGAGATCCAATTTTTATACCAACATTTATTAATAATTAATAATTCCATAAGTAGAAATATAAGGTATAAACCACATAGAACCT
AAAAAATAAGAAAAATTATAATTATTTAAAGCCTTATTATAAAAAAATAAAGAAACATTA
GAAATTATATAACCTATAATACCCCCAACAATACAAACAAATAATGTTAATAATTTTAAA
TAAGAAGGAAGAACAACTACTATAGGAGTAGGAAAAATCAATCATCTTAACATTCTCCCA
CCAAAAATTCTTAAAATTAATAAACCTATTATACTTTTCAATATTACTCAACCTTCATCA
TTTAATATATTTAAAGAAGAAAAATTAGAATCACCAGTTGTCTTCGCTCCCGCTAACATCACCACTGAAGTCAAGT
CCGTTGAAATGCACCACGAAGCTCTCGTTGAAGCCGTCCCCGGTGACAACGTTGGTTTCA
ACGTAAAGAACGTCTCCGTCAAGGAATTGCGTCGTGGTTACGTCGCTGGTGACTCCAAGG
CCAACCCCCCCAAGGGCGCCGCTGACTTCACCGCTCAAGTCATCGTCTTGAACCACCCTG
GTCAAATCTCCAACGGTTACACCCCCGTTTTGGATTGTCACACCGCTCACATTGCTTGCA
AATTCGCCGAAATCAAGGAGAAGGTCGATCGATGCACGCAGTACCCTAAGTCCGGTTCAGTGCTTTGAGGGCAGCGGTGGAAGT
GGTTCATCGGGAAATTTTACTTCGGGCAGTAACTTAAATATGGGCAGTGTGACTAATACC
AGCAACACCGGTACTGGTACTTCCTCAGGAAGTGTTCCCATGGTCACGTTGACCGAATCT
CTACTAAATAAACATAACGATGAAATGGAAAAGTTTATGCTTAAGAAGCATAGAGAATCT
CGAGGACGAAGTGGCGAGAAGAACAAAAAGAATTCTGAAAAGATAATGGAGTACACTGGC
CCGGGACACGGAATCAAAAGAGTTGGTTCACA

>ITMOII8Ls
AGAATTCTAATTCGAGCTGAATTAGGACATCCT
GGAGCTTTAATTGGAGATGATCAAATTTACAATGTAATTGTTACAGCTCATGCTTTTATT
ATAATTTTTTTTATAGTAATGCCAATTATAATTGGAGGATTTGGAAATTGATTAGTTCCA
TTAATACTAGGAGCTCCAGATATAGCATTCCCTCGAATAAATAATATAAGTTTTTGACTT
TTACCTCCTGCATTAACTTTATTATTAGTTAGTAGTATAGTAGAAAACGGAGCTGGAACA
GGATGAACAGTTTACCCTCCTCTATCTTCTAATATTGCTCATGGAGGAGCTTCTGTTGAT
TTAGCTATTTTCTCTCTTCATTTAGCAGGAATTTCTTCAATTTTAGGAGCTGTAAATTTT
ATTACTACAGTTATTAATATACGATCAACAGGAATTACTTTTGATCGAATACCTTTATTT
GTTTGATCAGTAGTAATTACAGCTTTATTACTTTTATTATCATTACCAGTATTAGCGGGA
GCTATTACAATACTTTTAACAGACCGAAATCTTAATACATCATTCTTTGACCCTGCAGGA
GGAGGAGATCCAATTTTATACCAACATTTATTTAATAATTAATAATTCCATAAGTAGAAATATATGGTATAAACCACAT
AGAACCTAAAAAATAAGAAAAATTATAATTATTTAAAGCCTTATTATAAAAAAATAAAGA
AACATTAGAAATTATATAACCTATAATACCCCCAACAATACAAACAAATAATGTTAATAA
TTTTAAATAAGAAGGAAGAACAACTACTATAGGAGTAGGAAAAATCAATCATCTTAACAT
TCTCCCACCAAAAATTCTTAAAATTAATAAACCTATTATACTTTTCAATATTACTCAACC
TTCATCATTTAATATATTTAAAGAAGAAAAATTAGAATCACCAGTTGTCTTCGCTCCCGCTAACATCACCACTGAAGTCAAG
TCCGTTGAAATGCACCACGAAGCTCTCGTTGAAGCCGTCCCCGGTGACAACGTTGGTTTC
AACGTAAAGAACGTCTCCGTCAAGGAATTGCGTCGTGGTTACGTCGCTGGTGACTCCAAG
GCTAACCCCCCCAAGGGCGCCGCTGACTTCACCGCTCAAGTCATCGTCTTGAACCACCCT
GGTCAAATCTCCAACGGTTACACCCCCGTTTTGGATTGTCACACCGCTCACATTGCTTGC
AAATTCGCCGAAATCAAGGAGAAGGTCGATCGATGCACGCAGTACCCTAAGTCCGGTTCAGTGCTTTGAGGGCAGCGGTGGAAGT
GGTTCATCGGGAAATTTTACTTCGGGCAGTAACTTAAATATGGGCAGTGTGACTAATACC
AGCAACACCGGTACTGGTACTTCCTCAGGAAGTGTTCCCATGGTCACGTTGACGGAATCT
CTACTAAATAAACATAACGATGAAATGGAAAAGTTTATGCTTAAGAAGCATAGAGAATCT
CGAGGACGAAGTGGCGAGAAGAACAAAAAGAATTCTGAAAAGATAATGGAGTACACTGGC
CCGGGACACGGAATCAAAAGAGTTGGTTCACA

>ITMOII9Ls
AGAATTCTAATTCGAGCTGAATTAGGACATCCT
GGAGCTTTAATTGGAGATGATCAAATTTATAATGTAATTGTTACAGCTCATGCTTTTATT
ATAATTTTTTTTATAGTAATGCCAATTATAATTGGAGGATTTGGAAATTGATTAGTTCCA
TTAATACTAGGAGCTCCAGATATAGCATTCCCTCGAATAAATAATATAAGTTTTTGACTT
TTACCTCCTGCATTAACTTTATTATTAGTTAGTAGTATAGTAGAAAACGGAGCTGGAACA
GGATGAACAGTTTACCCTCCTCTATCTTCTAATATTGCTCATGGAGGAGCTTCTGTTGAT
TTAGCTATTTTCTCTCTTCATTTAGCAGGAATTTCTTCAATTTTAGGAGCTGTAAATTTT
ATTACTACAGTTATTAATATACGATCAACAGGAATTACTTTTGATCGAATACCTTTATTT
GTTTGATCAGTAGTAATTACAGCTTTATTACTTTTATTATCATTACCAGTATTAGCAGGA
GCTATTACAATACTTTTAACAGACCGAAATCTTAATACATCATTCTTTGACCCTGCAGGA
GGAGGAGATCCAATTTTATACCAACATTTATTT

AATAATTAATAATTCCATAAGTAGAAATATAAGGTATAAACCA
CATAGAACCTAAAAAATAAGAAAAATTATAATTATTTAAAGCCTTATTATAAAAAAATAA
AGAAACATTAGAAATTATATAACCTATAATACCCCCAACAATACAAACAAATAATGTTAA
TAATTTTAAATAAGAAGGAAGAACAACTACTATAGGAGTAGGAAAAATCAATCATCTTAA
CATTCTCCCACCAAAAATTCTTAAAATTAATAAACCTATTATACTTTTCAATATTACTCA
ACCTTCATCATTTAATATATTTAAAGAAGAAAAATTAGAATCACCAGTTGTCTTCGCTCCCGCTAACATCACCACTGAAGTCAAGTCCGTTGAAATGC
ACCACGAAGCTCTCGTTGAAGCCGTCCCCGGTGACAACGTTGGTTTCAACGTAAAGAACG
TCTCCGTCAAGGAATTGCGTCGTGGTTACGTCGCTGGTGACTCCAAGGCCAACCCCCCCA
AGGGCGCCGCTGACTTCACCGCTCAAGTCATCGTCTTGAACCACCCTGGTCAAATCTCCA
ACGGTTATACCCCCGTTTTGGATTGTCACACCGCTCACATTGCTTGCAAATTCGCCGAAA
TCAAGGAGAAGGTCGATCGATGCACGCAGTACCCTAAGTCCGGTTCAGTGCTTTGAGGGCAGCGGTGGAAGTGGTTCGTCGGGAAATTTTACTTCGGGCAGTAACTTAAATATGGGCAGTGTGACTAATAC
CAGCAACACCGGTACTGGTACTTCCTCTGGAAGTGTTCCCATGGTCACGTTGACCGAATC
TCTACTAAATAAACATAACGATGAAATGGAAAAGTTTATGCTTAAGAAGCATAGAGAATC
TCGAGGACGAAGTGGCGAGAAGAACAAAAAGAATTCTGAAAAGATAATGGAGTACACTGG
CCCGGGACACGGAATCAAAAGAGTTGGTTCACA

>ITTVLs1

AGAATTCTAATTCGAGCTGAATTAGGACAT
CCTGGAGCTTTAATTGGAGATGATCAAATTTATAATGTAATTGTTACAGCTCATGCTTTT
ATTATAATTTTTTTTATAGTAATGCCAATTATAATTGGAGGATTTGGAAATTGATTAGTT
CCATTAATACTAGGAGCTCCAGATATAGCATTCCCTCGAATAAATAATATAAGTTTTTGA
CTTTTACCTCCTGCATTAACTTTATTATTAGTTAGTAGTATAGTAGAAAACGGAGCTGGA
ACAGGATGAACAGTTTACCCTCCTCTATCTTCTAATATTGCTCATGGAGGAGCTTCTGTT
GATTTAGCTATTTTCTCTCTTCATTTAGCAGGAATTTCTTCAATTTTAGGAGCTGTAAAT
TTTATTACTACAGTTATTAATATACGATCAACAGGAATTACTTTTGATCGAATACCTTTA
TTTGTTTGATCAGTAGTAATTACAGCTTTATTACTTTTATTATCATTACCAGTATTAGCA
GGAGCTATTACAATACTTTTAACAGACCGAAATCTTAATACATCATTCTTTGACCCTGCA
GGAGGAGGAGATCCAATTTTATACCAACATTTATTTAATAATTAATAATTCCATAAGTAGAAATATAAGAGTATAAACCACATAGA

ACCTAAAAAATAAGAAAAATTATAATTATTTAAAGCCTTATTATAAAAAAATAAAGAAAC

ATTAGAAATTATATAACCTATAATACCCCCAACAATACAAACAAATAATGTTAATAATTT

TAAATAAGAAGGAAGAACAACTACTATAGGAGTAGGAAAAATCAATCATCTTAACATTCT

CCCACCAAAAATTCTTAAAATTAATAAACCTATTATACTTTTCAATATTACTCAACCTTC

ATCATTTAATATATTTAAAGAAGAAAAATTAGAATCACCAGTTGTCTTCGCTCCCGCTAACATCACCACTGAAGTCAAG

TCCGTTGAAATGCACCACGAAGCTCTCGTTGAAGCCGTCCCCGGTGACAACGTTGGTTTC

AACGTAAAGAACGTCTCCGTCAAGGAATTGCGTCGTGGTTACGTCGCTGGTGACTCCAAG

GCTAACCCCCCCAAGGGCGCCGCTGACTTCACCGCTCAAGTCATCGTCTTGAACCACCCT

GGTCAAATCTCCAACGGTTACACCCCCGTTTTGGATTGTCACACCGCTCACATTGCTTGC

AAATTCGCCGAAATCAAGGAGAAGGTCGAT CGATGCACGCAGTACCCTAAGTCCGGTTCAGTGCTTTGAGGGCAGCGGTGGAAGT

GGTTCATCGGGAAATTTTACTTCGGGCAGTAACTTAAATATGGGCAGTGTGACTAATACC

AGCAACACCGGTACTGGTACTTCCTCTGGAAGTGTTCCCATGGTCACGTTGACCGAATCT

CTACTAAATAAACATAACGATGAAATGGAAAAGTTTATGCTTAAGAAGCATAGAGAATCT

CGAGGACGAAGTGGCGAGAAGAACAAAAAGAATTCTGAAAAGATAATGGAGTACACTGGC

CCGGGACACGGAATCAAAAGAGTTGGTTCACA

>UKCvo

AGAATTTTAATTCGAGCTGAACTAGGGCATC
CTGGAGCATTAATTGGAGATGACCAAATTTATAATGTAATTGTTACAGCTCATGCTTTTA
TTATAATTTTTTTTATAGTAATACCAATTATAATTGGAGGGTTTGGAAATTGATTAGTTC
CTTTAATATTAGGAGCCCCAGATATAGCATTCCCTCGAATAAATAATATAAGTTTCTGAC
TTTTACCTCCTGCATTAACTTTATTATTAGTAAGTAGTATAGTAGAAAACGGAGCTGGAA
CTGGATGAACTGTTTATCCACCTTTATCTTCTAATATTGCACATGGAGGAGCTTCTGTTG
ATTTAGCTATTTTTTCTTTACATTTAGCAGGAATTTCTTCAATTTTAGGAGCTGTAAATT
TTATTACTACAGTTATTAATATACGATCAACAGGTATTACCTTCGACCGAATACCATTAT
TTGTTTGATCAGTAGTAATTACAGCCTTATTACTTTTATTATCTTTACCAGTATTAGCAG
GAGCTATTACTATATTATTAACAGATCGAAATCTTAATACTTCATTCTTTGACCCAGCAG
GAGGAGGAGATCCAATTTTATACCAACACTTATTTAATAATTAATAATTCCGTATGTAGAAATATAAGGCATAAACCACAT

TGACCCTAAAAAATAAGAAGAATTATAGTTATTTAAAGCCTTATTATAAAAAAATAAAGA

AATATTAGAAATTAAATACCCTCTAATTCCTCCTACAATACAAACAAATAAAGTTAATAA

CTTTAAATAAGAAGGTAGAACTACTACTACTGGTGTAGGAAAAATTAATCAACTTAACAT

TCTACCCCCAAAAATTCTTAAAATTAATAACCCTATTATACTCTTTAACATAACTCAACC

TTCATCATTTAATATATTTAAAGAAGAAAAATTTGAATCCCCAGTTGTCTTCGCTCCCGCTAACATCACCACTGAAGTCAAATCCGTAGAAATGCACCACGAAGCTCTCGTT

GAAGCCGTCCCCGGTGACAACGTTGGTTTCAACGTTAAGAACGTCTCCGTCAAGGAATTG

CGTCGTGGTTACGTCGCTGGTGACTCCAAGGCTAGCCCCCCCAAGGGTGCTGCTGACTTC

ACCGCTCAAGTCATCGTCTTGAACCACCCTGGTCAAATCTCCAACGGTTACACTCCCGTT

TTGGATTGTCACACCGCTCACATTGCTTGCAAATTCGCCGAAATCAAGGAGAAGGTCGATTGAAGCACGCAGTACCCTGAGTCCGGTTCAGTGCTTTGAGGGTAGCGGTGGTAGTGGATC

CTCAGGAAATTTTACTTCGGGCAGCAACTTGAATATGGGCAGTGTTACTAATACAAGCAA

TACCGGTACAGGCACTTCATCGGGAAGTGTACCCATGGTTACGTTGACGGAATCTCTACT

AAATAAACACAACGATGAAATGGAAAAGTTTATGCTAAAGAAACACAGAGAATCTCGCGG

ACGCAGTGGTGAGAAGAATAAAAAGAACTCGGAAAAGATAATGGAGTATTCTGGTCCTGG

GCATGGAATAAAGAGAGTTGGCTCACA

>ITTVLs2

AGAATTCTAATTCGAGCTGAATTAGGACATC
CTGGAGCTTTAATTGGAGATGATCAAATTTATAATGTAATTGTTACAGCTCATGCTTTTA
TTATAATTTTTTTTATGGTAATGCCAATTATAATTGGAGGATTTGGAAATTGATTAGTTC
CATTAATACTAGGAGCTCCAGATATAGCATTCCCTCGAATAAATAATATAAGTTTTTGAC
TTTTACCTCCTGCATTAACTTTATTATTAGTTAGTAGTATAGTAGAAAACGGAGCTGGAA
CAGGATGAACAGTTTACCCTCCTCTATCTTCTAATATTGCTCATGGAGGAGCTTCTGTTG
ATTTAGCTATTTTCTCTCTTCATTTAGCAGGAATTTCTTCAATTTTAGGAGCTGTAAATT
TTATTACTACAGTTATTAATATACGATCAACAGGAATTACTTTTGATCGAATACCTTTAT
TTGTTTGATCAGTAGTAATTACAGCTTTATTACTTTTATTATCATTACCAGTATTAGCAG
GAGCTATTACAATACTTTTAACAGACCGAAATCTTAATACATCATTCTTTGACCCTGCAG
GAGGAGGAGATCCAATTTTATACCAACATTTATTTAATAATTAATAATTCCATAAGTAGAAATATAAGAGTATAAACCACATAGAACC

TAAAAAATAAGAAAAATTATAATTATTTAAAGCCTTATTATAAAAAAATAAAGAAACATT

AGAAATTATATAACCTATAATACCCCCAACAATACAAACAAATAATGTTAATAATTTTAA

ATAAGAAGGAAGAACAACTACTATAGGAGTAGGAAAAATCAATCATCTTAACATTCTCCC

ACCAAAAATTCTTAAAATTAATAAACCTATTATACTTTTCAATATTACTCAACCTTCATC

ATTTAATATATTTAAAGAAGAAAAATTAGAATCACCAGTTGTCTTCGCTCCCGCTAACATCACCACTGAAGTCAAGTCCGTTGAAATGCACCACGAAGCTCTCGTTGAAGCCGTCCCCGGTGACAACGTTG

GTTTCAACGTAAAGAACGTCTCCGTTAAGGAATTGCGTCGTGGTTACGTCGCTGGTGACT

CCAAGGCCAACCCCCCCAAGGGCGCCGCTGACTTCACCGCTCAAGTCATCGTCTTGAACC

ACCCTGGTCAAATCTCCAACGGTTACACCCCCGTTTTGGATTGTCACACCGCTCACATTG

CTTGCAAATTCGCCGAAATCAAGGAGAAGGTCGATGGATGCACGCAGTACCCTAAGTCCGGTTCAGTGCTTTGAGGGCAGCGGTGGAAGT

GGTTCATCGGGAAATTTTACTTCGGGCAGTAACTTAAATATGGGCAGTGTGACTAATACC

AGCAACACCGGTACTGGTACTTCCTCAGGAAGTGTTCCCATGGTCACGTTGACGGAATCT

CTACTAAATAAACATAACGATGAAATGGAAAAGTTTATGCTTAAGAAGCATAGAGAATCT

CGAGGACGAAGTGGCGAGAAGAACAAAAAGAATTCTGAAAAGATAATGGAGTACACTGGC

CCGGGACACGGAATCAAAAGAGTTGGTTCACA

>ITTVLs3

AGAATTCTAATTCGAGCTGAATTAGGACATC
CTGGAGCTTTAATTGGAGATGATCAAATTTATAATGTAATTGTTACAGCTCATGCTTTTA
TTATAATTTTTTTTATAGTAATGCCAATTATAATTGGAGGATTTGGAAATTGATTAGTTC
CATTAATACTAGGAGCTCCAGATATAGCATTCCCTCGAATAAATAATATAAGTTTTTGAC
TTTTACCTCCTGCATTAACTTTATTATTAGTTAGTAGTATAGTAGAAAACGGAGCTGGAA
CAGGATGAACAGTTTACCCTCCTCTATCTTCTAATATTGCTCATGGAGGAGCTTCTGTTG
ATTTAGCTATTTTCTCTCTTCATTTAGCAGGAATTTCTTCAATTTTAGGAGCTGTAAATT
TTATTACTACAGTTATTAATATACGATCAACAGGAATTACTTTTGATCGAATACCTTTAT
TTGTTTGATCAGTAGTAATTACAGCTTTATTACTTTTATTATCATTACCAGTATTAGCAG
GAGCTATTACAATACTTTTAACAGACCGAAATCTTAATACATCATTCTTTGACCCTGCAG
GAGGAGGAGATCCAATTTTATACCAACATTTATTTAATAATTAATAATTCCATAAGTAGAAATATAAGAGTATAAACCACATAGAACC

TAAAAAATAAGAAAAATTATAATTATTTAAAGCCTTATTATAAAAAAATAAAGAAACATT

AGAAATTATATAACCTATAATACCCCCAACAATACAAACAAATAATGTTAATAATTTTAA

ATAAGAAGGAAGAACAACTACTATAGGAGTAGGAAAAATCAATCATCTTAACATTCTCCC

ACCAAAAATTCTTAAAATTAATAAACCTATTATACTTTTTAATATTACTCAACCTTCATC

ATTTAATATATTTAAAGAAGAAAAATTAGAATCACCAGTTGTCTTCGCTCCCGCTAACATCACCACTGAAGTCAAG

TCCGTTGAAATGCACCACGAAGCTCTCGTTGAAGCCGTCCCCGGTGACAACGTTGGTTTC

AACGTAAAGAACGTCTCCGTCAAGGAATTGCGTCGTGGTTACGTCGCTGGTGACTCCAAG

GCCAACCCCCCCAAGGGCGCCGCTGACTTCACCGCTCAAGTCATCGTCTTGAACCACCCT

GGTCAAATCTCCAACGGTTACACCCCCGTTTTGGATTGTCACACCGCTCACATTGCTTGC

AAATTCGCCGAAATCAAGGAGAAGGTCGATGGATGCACGCAGTACCCTAAGTCCGGTTCAGTGCTTTGAGGGCAGCGGTGGAAGT

GGTTCATCGGGAAATTTTACTTCGGGCAGTAACTTAAATATGGGCAGTGTGACTAATACC

AGCAACACCGGTACTGGTACTTCCTCAGGAAGTGTTCCCATGGTCACGTTGACGGAATCT

CTACTAAATAAACATAACGATGAAATGGAAAAGTTTATGCTTAAGAAGCATAGAGAATCT

CGAGGACGAAGTGGCGAGAAGAACAAAAAGAATTCTGAAAAGATAATGGAGTACACTGGC

CCGGGACACGGAATCAAAAGAGTTGGTTCACA

>ITTVLc1

AGAATTTTAATTCGAGCTGAATTAGGACACCCTGGTG
CATTAATTGGAGATGACCAAATTTATAATGTAATTGTTACAGCTCATGCTTTTATTATAA
TTTTCTTTATAGTAATACCAATTATAATTGGAGGATTTGGAAATTGATTAGTTCCTTTAA
TATTAGGAGCCCCAGATATGGCATTCCCTCGAATAAATAATATAAGATTTTGACTTTTAC
CTCCTGCATTAACTTTATTATTAGTAAGTAGTATAGTAGAAAACGGAGCTGGAACAGGAT
GAACAGTTTACCCTCCTTTATCATCTAATATCGCTCATGGAGGAGCTTCTGTTGATTTAG
CTATTTTTTCTCTTCATTTAGCAGGAATCTCATCAATTTTAGGGGCTGTAAATTTTATTA
CTACAGTTATTAATATACGATCAACAGGAATTACTTTTGACCGAATACCTTTATTCGTTT
GATCAGTAGTAATTACAGCTTTATTACTTTTATTATCATTACCAGTATTAGCTGGAGCTA
TTACTATACTTTTAACTGACCGAAATCTTAATACTTCATTCTTTGACCCAGCAGGAGGAG
GAGACCCAATTTTATACCAACATTTATTTAATAATTAATAATTCCGTAAGTAGAAATATAAGGTATAAACCACATT

GACCCTAAAAAATAAGAAAAATTATAATTATTTAAAGCCTTATTATAAAAAAATAAAGAA

ATATTTGAAATTATATACCCTCTTACTCCTCCTACAATACAAACAAATAATGTTAATAAC

TTCAAATATGAAGGAAGAACTACAACTACTGGAGTTGGAAAAATTAATCATCTTAATATT

CTTCCCCCAAAAATTCTTAAAATTAATAACCCTATTATACTTTTTAATATTACTCAACCT

TCATCATTTAATATATTTAAAGAAGAAAAATTTGAATCACCAGTTGTCATCGCTCCCGCTAACATCACCACTGAAGTCAAGTCCGTTGAAAT

GCACCACGAAGCTCTCGTTGAAGCCGTCCCCGGTGACAACGTTGGTTTCAACGTAAAGAA

CGTCTCCGTCAAGGAATTGCGTCGTGGTTACGTCGCTGGTGACTCCAAGGCCAACCCCCC

CAAGGGTGCCGCTGACTTCACCGCTCAAGTCATCGTCTTGAACCATCCCGGTCAAATCTC

CAACGGTTACACTCCCGTTTTGGATTGTCACACCGCTCACATTGCTTGCAAATTCGCCGA

AATCAAGGAGAAGGTCGATTGATGCACGCAGTGCTTTGAGTCCGGTTCAGTGCTTTGAAGGCAGCGGTGGTAG

TGGTTCATCGGGAAATTTTACTTCGGGCAGTAACTTGAATATGGGCAGTGTGACTAATAC

CAGCAACACTGGTACCGGCACTTCCTCGGGAAGTGTTCCCATGGTTACATTAACGGAATC

TCTGCTAAATAAGCATAACGATGAAATGGAAAAATTTATGCTAAAAAAGCACAGAGAATC

TCGTGGACGAAGTGGCGAGAAGAATAAAAAGACTTCTGAAAAGATAATGGAATATACTGG

ACCGGGGCATGGAATCAAAAGAGTTGGATCACA

>ITTVCvo3

AGAATTTTAATTCGAGCTGAACTAGGGCAT
CCTGGAGCATTAATTGGAGATGATCAAATTTATAATGTAATTGTTACAGCTCATGCTTTT
ATTATAATTTTTTTTATAGTAATACCAATTATAATTGGAGGGTTTGGAAATTGATTAGTT
CCTTTAATATTAGGAGCCCCAGATATAGCATTCCCTCGAATAAATAATATAAGTTTCTGA
CTTTTACCTCCTGCATTAACTTTACTATTAGTAAGTAGTATAGTAGAAAACGGAGCTGGA
ACTGGATGAACTGTTTATCCACCTTTATCTTCTAATATTGCACATGGAGGAGCTTCTGTT
GATTTAGCTATTTTTTCTTTACATTTAGCAGGAATTTCTTCAATTTTAGGAGCTGTAAAT
TTTATTACTACAGTTATTAATATACGATCAACAGGTATTACCTTCGACCGAATACCATTA
TTTGTTTGATCAGTAGTAATTACAGCCTTATTACTTTTATTATCTTTACCAGTATTAGCA
GGAGCTATTACTATATTATTAACAGATCGAAATCTTAATACTTCATTCTTTGACCCAGCA
GGAGGAGGAGATCCAATTTTATACCAACACTTATTTAGTAATTAATAATTCCGTATGTAGAAATATAAGGCATAAACCACATT

GACCCTAAAAAATAAGAAGAATTATAGTTATTTAAAGCCTTATTATAAAAAAATAAAGAA

ATATTAGAAATTAAATACCCTCTAATTCCTCCTACAATACAAACAAATAAAGTTAATAAC

TTTAAATAAGAAGGTAGAACTACTACTACTGGTGTAGGAAAAATTAATCAACTTAACATT

CTACCCCCAAAAATTCTTAAAATTAATAACCCTATTATACTCTTTAACATAACTCAACCT

TCATCATTTAATATATTTAAAGAAGAAAAATTTGAATCCCCA

GTTGTCTTCGCTCCCGCTAACATCACCACTGAAGTCAAATCCGTAG

AAATGCACCACGAAGCTCTCGTTGAAGCCGTCCCCGGTGACAACGTTGGTTTCAACGTTA

AGAACGTCTCCGTCAAGGAATTGCGTCGTGGTTACGTCGCTGGTGACTCCAAGGCTAGCC

CCCCCAAGGGTGCTGCTGACTTCACCGCTCAAGTCATCGTCTTGAACCACCCTGGTCAAA

TCTCCAACGGTTACACTCCCGTTTTGGATTGTCACACCGCTCACATTGCTTGCAAATTCG

CCGAAATCAAGGAGAAGGTCGATTGAA

GCACGCAGTACCCTGAGTCCGGTTCAGTGCTTTGAGGGTAGCGGTGGTAGTGGATCCTCA

GGAAATTTTACTTCGGGCAGCAACTTGAATATGGGCAGTGTTACTAATACAAGCAATACC

GGTACAGGCACTTCATCGGGAAGTGTACCCATGGTTACGTTGACGGAATCTCTACTAAAT

AAACACAACGATGAAATGGAAAAGTTTATGCTAAAGAAACACAGAGAATCTCGCGGACGA

AGTGGTGAGAAGAATAAAAAGAACTCGGAAAAGATAATGGAGTATACTGGTCCTGGGCAT

GGAATAAAGAGAGTTGGCTCACA

>ITTVCvi3

AGAATTCTAATTCGAGCCGAACTAGGAC
ATCCTGGAGCATTAATTGGAGATGACCAAATTTATAATGTAATTGTTACAGCTCATGCTT
TTATTATAATTTTTTTTATAGTAATACCAATTATAATTGGAGGATTTGGTAATTGATTAG
TCCCTTTAATATTAGGAGCTCCAGATATAGCCTTCCCTCGGATAAACAATATAAGTTTCT
GACTTTTACCTCCTGCATTAACTTTACTATTAGTAAGTAGTATAGTAGAAAACGGAGCTG
GAACAGGATGAACTGTTTACCCACCTTTATCTTCTAATATCGCTCATGGAGGAGCTTCTG
TTGATTTAGCTATTTTTTCTTTACACTTAGCAGGAATTTCTTCAATTTTAGGGGCTGTAA
ATTTTATTACTACAGTTATTAATATACGATCAACAGGAATTACATTCGACCGAATACCAT
TATTTGTTTGATCTGTAGTAATTACAGCTTTATTACTTTTATTATCTTTACCAGTATTAG
CAGGTGCTATTACTATATTATTAACAGATCGAAATCTTAATACATCATTCTTTGACCCAG
CAGGAGGAGGAGACCCAATCTTGTACCAACATTTATTTAATAATTAATAATTCCATATGTAGAAATATAAGGTATAAATCATATTGAA

CCTAAAAAGTAAGAAGAATTATAATTATTTAAAGCCTTATTATAAAAAAATAAAGAAATA

TTAGAAATTAAATAACCTCTAACTCCTCCTACAATACAAACAAATAAAGTTAATAATTTT

AAATAAGAAGGAAGAACTACTACTACTGGTGTAGGAAAAATTAATCAACTTAATATACTA

CCCCCAAAAATTCTCAAAATTAATAGTCCTATTATACTTTTTAATATAACTCAACCTTCA

TCATTTAGTATATTTAAAGAAGAAAAATTTGAATCCCCAGTTGTCTTCGCTCCCGCTAACATCACCACTGAAGTCAAGT

CCGTAGAAATGCACCACGAAGCTCTCGTTGAAGCTGTTCCCGGTGACAACGTTGGTTTCA

ACGTTAAGAACGTCTCCGTCAAGGAATTGCGTCGTGGTTACGTCGCTGGTGACTCCAAGG

CTAGCCCCCCCAAGGGTGCTGCTGACTTCACCGCTCAAGTCATCGTCTTGAACCACCCTG

GTCAAATCTCCAACGGTTACACTCCCGTTTTGGATTGTCACACCGCTCACATTGCTTGCA

AATTCGCCGAAATCAAGGAGAAGGTCGATTGAAGCACGCAGTACCCTGAGTCCGGTTCAGTGCTTTGAGGGTAGCGGTGGTAGT

GGATCCTCAGGAAATTTTACTTCGGGTAGCAACTTGAATATGGGCAGTGTTACTAATACA

AGCAATACCGGTACAGGCACTTCATCGGGAAGTGTACCCATGGTTACGTTGACGGAATCT

CTACTAAATAAACACAACGATGAAATGGAAAAGTTTATGCTAAAGAAACACAGAGAATCT

CGCGGACGAAGTGGTGAGAAGAATAAAAAGAACTCGGAAAAGATAATGGAGTATACTGGT

CCTGGGCATGGAATAAAGAGAGTTGGCTCACA

>ITMOCvi4

AGAATTCTAATTCGAGCCGAACTAGGACA
TCCTGGAGCATTAATTGGAGATGACCAAATTTATAATGTAATTGTTACAGCTCATGCTTT
TATTATAATTTTTTTTATAGTAATACCAATTATAATTGGAGGATTTGGTAATTGATTAGT
CCCTTTAATATTAGGAGCTCCAGATATAGCCTTCCCTCGGATAAACAATATAAGTTTCTG
ACTTTTACCTCCTGCATTAACTTTACTATTAGTAAGTAGTATAGTAGAAAACGGAGCTGG
AACAGGATGAACTGTTTACCCACCTTTATCTTCTAATATCGCTCATGGAGGAGCTTCTGT
TGATTTAGCTATTTTTTCTTTACACTTAGCAGGAATTTCTTCAATTTTAGGAGCTGTAAA
TTTTATTACTACAGTTATTAATATACGATCAACAGGAATTACATTCGACCGAATACCATT
ATTTGTTTGATCTGTAGTAATTACAGCTTTATTACTTTTATTATCTTTACCAGTATTAGC
AGGTGCTATTACTATATTATTAACAGATCGAAATCTTAATACATCATTCTTTGACCCAGC
AGGAGGAGGAGACCCAATCTTGTACCAACATTTATTTAATAATTAATAATTCCATATGTAGAAATATAAGGTATAAATCATATT

GAACCTAAAAAGTAAGAAGAATTATAATTATTTAAAGCCTTATTATAAAAAAATAAAGAA

ATATTAGAAATTAAATAACCTCTAACTCCTCCTACAATACAAACAAATAAAGTTAATAAT

TTTAAATAAGAAGGAAGAACTACTACTACTGGTGTAGGAAAAATTAATCAACTTAATATA

CTACCCCCAAAAATTCTCAAAATTAATAGTCCTATTATACTTTTTAATATAACTCAACCT

TCATCATTTAGTATATTTAAAGAAGAAAAATTTGAATCCCCAGTTGTCTTCGCTCCCGCTAACATCACCACTGAAGTCAAGTC

CGTAGAAATGCACCACGAAGCTCTCGTTGAAGCTGTTCCCGGTGACAACGTTGGTTTCAA

CGTTAAGAACGTCTCCGTCAAGGAATTGCGTCGTGGTTACGTCGCTGGTGACTCCAAGGC

TAGCCCCCCCAAGGGTGCTGCTGACTTCACCGCTCAAGTCATCGTCTTGAACCACCCTGG

TCAAATCTCCAACGGTTACACTCCCGTTTTGGATTGTCACACCGCTCACATTGCTTGCAA

ATTCGCCGAAATCAAGGAGAAGGTCGATTGAAGCACGCAGTACCCTGAGTCCGGTTCAGTGCTTTGAGGGTAGCGGTGGT

AGTGGATCCTCGGGAAATTTTACTTCGGGTAGCAACTTGAATATGGGCAGTGTTACTAAT

ACAAGCAATACCGGTACAGGCACTTCATCGGGAAGTGTACCCATGGTTACGTTGACGGAA

TCTCTACTAAATAAACACAACGATGAAATGGAAAAGTTTATGCTAAAGAAACACAGAGAA

TCTCGCGGACGAAGTGGTGAGAAGAATAAAAAGAACTCGGAAAAGATAATGGAGTATACT

GGTCCTGGGCATGGAATAAAGAGAGTTGGCTCACA

>ITTVSA

AGAATTCTTATTCGAGCAGAATTAGGTCAC
CCTGGTGCATTAATTGGTGATGATCAAATTTATAATGTAATTGTTACAGCCCATGCTTTC
ATTATAATTTTTTTTATAGTAATACCAATTATAATTGGAGGATTTGGAAATTGATTAGTG
CCAATTATACTAGGAGCTCCAGATATAGCCTTCCCTCGGATAAACAATATAAGTTTTTGA
CTTTTACCTCCTGCATTAACATTGCTTCTAGTAAGTAGTATAGTAGAAAATGGAGCTGGA
ACAGGTTGAACTGTATACCCTCCTTTATCTTCTAATATTGCTCATGGAGGAGCTTCTGTT
GATTTAGCTATTTTTTCTCTCCATTTAGCTGGAATTTCTTCAATTCTAGGAGCAGTAAAT
TTTATTACTACAGTTATTAATATACGATCAACAGGAATCACTTTTGATCGAATACCTTTA
TTTGTATGATCTGTAGTAATCACAGCCCTACTTTTATTACTTTCTTTACCTGTACTTGCC
GGAGCTATTACTATATTATTAACTGATCGAAATATTAATACTTCATTTTTTGACCCGGCA
GGAGGAGGAGATCCTATTCTATATCAACATTTATTTAATAATTTATAATCCCATAAGTAGAAATATAAGGCATAAATCAT

ATTGAACCTAAAAAATAAGAACTATTATATATATTTAAAGCCTTATTAGAAAAAAATAAA

GAAATATTAGAAATTAAATAACCTATTAATCCTCCTACAATACACACAAATAAAGTTAAT

AACTTTAAATAATAAGGTAAAACAATTACCATAGGACTAGGAAAAATTAATCAACTTAAT

ATTCTACCCCCAAAAATTCTTAAAATTAATAATCCTATTATACTTTTTAATATAACTCAA

CCCTCATCATTTAATATATTTAAAGAAGAAAAATTCGAATCTCCA

GTTGTCTTCGCCCCCGCTAACATCACCACTGAAGT

CAAGTCCGTTGAAATGCACCACGAAGCTCTTGTTGAAGCCGTTCCCGGTGACAACGTTGG

TTTCAACGTTAAGAACGTCTCCGTCAAGGAATTGCGTCGTGGTTACGTCGCTGGTGACTC

CAAGGCTTCTCCCCCCAAGGGTGCTGCTGACTTCACCGCTCAAGTTATTGTGTTGAACCA

TCCTGGTCAAATCTCCAACGGTTATACCCCCGTCTTGGATTGTCACACCGCTCACATTGC

TTGCAAATTCGCTGAAATCAAGGAGAAGGTCGATTGATGCACGAAGTACATTAAGCCCGGTTCAGTGCTTTGAGGGTAGTGGTGGCAG

TGGTTCGTCAGGAAATTTTACATCTGGCAGTAATCTTAATATGGGCAGTGTCACTAACAC

AAGCAATACCGGCACGGGTACTTCCTCGGGTAGTGTTCCCATGGTAACTTTAACAGAATC

TCTGTTAAATAAACACAACGATGAAATGGAAAAATTTATGCTGAAAAAACATCGCGAATC

TCGTGGACGAAGCGGTGAAAAAAATAAAAAGAATACCGAAAAAATAATGGAATACAGTGG

TCCAGGGCATGGAATTAAACGGGTAGGATCCCA

>ITMOCvi3

AGAATTCTAATTCGAGCCGAACTAGGACATCCTGGA
GCATTAATTGGAGATGACCAAATTTATAATGTAATTGTTACAGCTCATGCTTTTATTATA
ATTTTTTTTATAGTAATACCAATTATAATTGGAGGATTTGGTAATTGATTAGTCCCTTTA
ATATTAGGAGCTCCAGATATAGCCTTCCCTCGGATAAACAATATAAGTTTCTGACTTTTA
CCTCCTGCATTAACTTTACTATTAGTAAGTAGTATAGTAGAAAACGGAGCTGGAACAGGA
TGAACTGTTTACCCACCTTTATCTTCTAATATCGCTCATGGAGGAGCTTCTGTTGATTTA
GCTATTTTTTCTTTACACTTAGCAGGAATTTCTTCAATTTTAGGAGCTGTAAATTTTATT
ACTACAGTTATTAATATACGATCAACAGGAATTACATTCGACCGAATACCATTATTTGTT
TGATCTGTAGTAATTACAGCTTTATTACTTTTATTATCTTTACCAGTATTAGCAGGTGCT
ATTACTATATTATTAACAGATCGAAATCTTAATACATCATTCTTTGACCCAGCAGGAGGA
GGAGACCCAATCTTGTACCAACATTTATTTAATAATTAATAATTCCATATGTAGAAATATAAGGTATAAATCATATTGA

ACCTAAAAAGTAAGAAGAATTATAATTATTTAAAGCCTTATTATAAAAAAATAAAGAAAT

ATTAGAAATTAAATAACCTCTAATTCCTCCTACAATACAAACAAATAAAGTTAATAATTT

TAAATAAAAAGGAAGAACTACTACTACTGGTGTAGGAAAAATTAATCAACTTAATATACT

ACCCCCAAAAATTCTCAAAATTAATAGTCCTATTATACTTTTTAATATAACTCAACCTTC

ATCATTTAGTATATTTAAAGAAGAAAAATTTGAATCTCCAGTTGTCTTCGCTCCCGCTAACATCACCACTGAAGTCAAGTCCGTAGAAATGCACCACGAA

GCTCTCGTTGAAGCTGTTCCCGGTGACAACGTTGGTTTCAACGTTAAGAACGTCTCCGTC

AAGGAATTGCGTCGTGGTTACGTCGCTGGTGACTCCAAGGCTAGCCCCCCCAAGGGTGCT

GCTGACTTCACCGCTCAAGTCATCGTCTTGAACCACCCTGGTCAAATCTCCAACGGTTAC

ACTCCCGTTTTGGATTGTCACACCGCTCACATTGCTTGCAAATTCGCCGAAATCAAGGAG

AAGGTCGATTGAAGCACGCAGTACCCTGAGTCCGGTTCAGTGCTTTGAGGGTAGCGGTGGTAGT

GGATCCTCAGGAAATTTTACTTCGGGTAGCAACTTGAATATGGGCAGTGTTACTAATACA

AGCAATACCGGTACAGGCACTTCATCGGGAAGTGTACCCATGGTTACGTTGACGGAATCT

CTACTAAATAAACACAACGATGAAATGGAAAAGTTTATGCTAAAGAAACACAGAGAATCT

CGCGGACGAAGTGGTGAGAAGAATAAAAAGAACTCGGAAAAGATAATGGAGTATACTGGT

CCTGGGCATGGAATAAAGAGAGTTGGCTCACA

>ITVVLs

AGAATTCTAATTCGAGCTGAATTAGGACATCCT
GGAGCTTTAATTGGAGATGATCAAATTTATAATGTAATTGTTACAGCTCATGCTTTTATT
ATAATTTTTTTTATAGTAATGCCAATTATAATTGGAGGATTTGGAAATTGATTAGTTCCA
TTAATACTAGGAGCTCCAGATATAGCATTCCCTCGAATAAATAATATAAGTTTTTGACTT
TTACCTCCTGCATTAACTTTATTATTAGTTAGTAGTATAGTAGAAAACGGAGCTGGAACA
GGATGAACAGTTTACCCTCCTCTATCTTCTAATATTGCTCATGGAGGAGCTTCTGTTGAT
TTAGCTATTTTCTCTCTTCATTTAGCAGGAATTTCTTCAATTTTAGGAGCTGTAAATTTT
ATTACTACAGTTATTAATATACGATCAACAGGAATTACTTTTGATCGAATACCTTTATTT
GTTTGATCAGTAGTAATTACAGCTTTATTACTTTTATTATCATTACCAGTATTAGCAGGA
GCTATTACAATACTTTTAACAGACCGAAATCTTAATACATCATTCTTTGACCCTGCAGGA
GGAGGAGATCCAATTTTATACCAACATTTATTTATAATTTAATAATTCCATAAGTAGAAATATAAGAGGTATAAACCACATAGAA

CCTAAAAAATAAGAAAAATTATAATTATTTAAAGCCTTATTATAAAAAAATAAAGAAACA

TTAGAAATTATATAACCTATAATACCCCCAACAATACAAACAAATAATGTTAATAATTTT

AAATAAGAAGGAAGAACAACTACTATAGGAGTAGGAAAAATCAATCATCTTAACATTCTC

CCACCAAAAATTCTTAAAATTAATAAACCTATTATACTTTTCAATATTACTCAACCTTCA

TCATTTAATATATTTAAAGAAGAAAAATTAGAATCACCAGTTGTCTTCGCTCCCGCTAACATCACCACTGAAGTCA

AGTCCGTTGAAATGCACCACGAAGCTCTCGTTGAAGCCGTCCCCGGTGACAACGTTGGTT

TCAACGTAAAGAACGTCTCCGTCAAGGAATTGCGTCGTGGTTACGTCGCTGGTGACTCCA

AGGCCAACCCCCCCAAGGGCGCCGCTGACTTCACCGCTCAAGTCATCGTCTTGAACCACC

CTGGTCAAATCTCCAACGGTTACACCCCCGTTTTGGATTGTCACACCGCTCACATTGCTT

GCAAATTCGCCGAAATCAAGGAGAAGGTCGATGGATGCACGCAGTACCCTAAGTCCGGTTCAGTGCTTTGAGGGCAGCGGTGGAAG

TGGTTCATCGGGAAATTTTACTTCGGGCAGTAACTTAAATATGGGCAGTGTGACTAATAC

CAGCAACACCGGTACTGGTACTTCCTCAGGAAGTGTTCCCATGGTCACGTTGACCGAATC

TCTACTAAATAAACATAACGATGAAATGGAAAAGTTTATGCTTAAGAAGCATAGAGAATC

TCGAGGACGAAGTGGCGAGAAGAACAAAAAGAATTCTGAAAAGATAATGGAGTACACTGG

CCCGGGACACGGAATCAAAAGAGTTGGTTCACA

>1ITLse

AGAATTCTAATTCGAGCTGAATTAGGACATC
CTGGAGCTTTAATTGGAGACGATCAAATTTATAATGTAATTGTTACAGCTCATGCTTTTA
TTATAATTTTTTTTATAGTAATGCCAATTATAATTGGAGGATTTGGAAATTGATTAGTTC
CATTAATACTAGGAGCTCCAGATATAGCATTCCCTCGAATAAATAATATAAGTTTTTGAC
TTTTACCTCCTGCATTAACTTTATTATTAGTTAGTAGTATAGTAGAAAACGGAGCTGGAA
CAGGATGAACAGTTTACCCTCCTCTATCTTCTAATATTGCTCATGGAGGAGCTTCTGTTG
ATTTAGCTATTTTCTCTCTTCATTTAGCAGGAATTTCTTCAATTTTAGGAGCTGTAAATT
TTATTACTACAGTTATTAATATACGATCAACAGGAATTACTTTTGATCGAATACCTTTAT
TTGTTTGATCAGTAGTAATTACAGCTTTATTACTTTTATTATCATTACCAGTATTAGCAG
GAGCTATTACAATACTTTTAACAGACCGAAATCTTAATACATCATTCTTTGACCCTGCAG
GAGGAGGAGATCCAATTTTATACCAACATTTATTTAATAATTAATAATTCCATAAGTAGAAATATAAGAGTATAAACCACATAGAAC

CTAAAAAATAAGAAAAATTATAATTATTTAAAGCCTTATTATAAAAAAATAAAGAAACAT

TAGAAATTATATAACCTATAATACCCCCAACAATACAAACAAATAATGTTAATAATTTTA

AATAAGAAGGAAGAACAACTACTATAGGAGTAGGAAAAATCAATCATCTTAACATTCTCC

CACCAAAAATTCTTAAAATTAATAAACCTATTATACTTTTCAATATTACTCAACCTTCAT

CATTTAATATATTTAAAGAAGAAAAATTAGAATCACCA

GTTGTCTTCGCTCCCGCTAACATCACCACTGAAGTCAAGT

CCGTTGAAATGCACCACGAAGCTCTCGTTGAAGCCGTCCCCGGTGACAACGTTGGTTTCA

ACGTAAAGAACGTCTCCGTTAAGGAATTGCGTCGTGGTTACGTCGCTGGTGACTCCAAGG

CCAACCCCCCCAAGGGCGCCGCTGACTTCACCGCTCAAGTCATCGTCTTGAACCACCCTG

GTCAAATCTCCAACGGTTACACCCCCGTTTTGGATTGTCACACCGCTCACATTGCTTGCA

AATTCGCCGAAATCAAGGAGAAGGTCGATGGATGCACGCAGTACCCTAAGTCCGGTTCAGTGCTTTGAGGGCAGCGGTGGAAG

TGGTTCATCGGGAAATTTTACTTCGGGCAGTAACTTAAATATGGGCAGTGTGACTAATAC

CAGCAACACCGGTACTGGTACTTCCTCAGGAAGTGTTCCCATGGTCACGTTGACGGAATC

TCTACTAAATAAACATAACGATGAAATGGAAAAGTTTATGCTTAAGAAGCATAGAGAATC

TCGAGGACGAAGTGGCGAGAAGAACAAAAAGAATTCTGAAAAGATAATGGAGTACACTGG

CCCGGGACACGGAATCAAAAGAGTTGGTTCACA

>2ITLse

AGAATTCTAATTCGAGCTGAATTAGGACATCCT
GGAGCTTTAATTGGAGATGATCAAATTTATAATGTAATTGTTACAGCTCATGCTTTTATT
ATAATTTTTTTTATAGTAATGCCAATTATAATTGGAGGATTTGGAAATTGATTAGTTCCA
TTAATACTAGGAGCTCCAGATATAGCATTCCCTCGAATAAATAATATAAGTTTTTGACTT
TTACCTCCTGCATTAACTTTATTATTAGTTAGTAGTATAGTAGAAAACGGAGCTGGAACA
GGATGAACAGTTTACCCTCCTCTATCTTCTAATATTGCTCATGGAGGAGCTTCTGTTGAT
TTAGCTATTTTCTCTCTTCATTTAGCAGGAATTTCTTCAATTTTAGGAGCTGTAAATTTT
ATTACTACAGTTATTAATATACGATCAACAGGAATTACTTTTGATCGAATACCTTTATTT
GTTTGATCAGTAGTAATTACAGCTTTATTACTTTTATTATCATTACCAGTATTAGCAGGA
GCTATTACAATACTTTTAACAGACCGAAATCTTAATACATCATTCTTTGACCCTGCAGGA
GGAGGAGATCCAATTTTATACCAACATTTATTTAATAATTAATAATTCCATAAGTAGAAATATAAGGTATAAACCAC

ATAGAACCTAAAAAATAAGAAAAATTATAATTATTTAAAGCCTTATTATAAAAAAATAAA

GAAACATTAGAAATTATATAACCTATAATACCCCCAACAATACAAACAAATAATGTTAAT

AATTTTAAATAAGAAGGAAGAACAACTACTATAGGAGTAGGAAAAATCAATCATCTTAAC

ATTCTCCCACCAAAAATTCTTAAAATTAATAAACCTATTATACTTTTCAATATTACTCAA

CCTTCATCATTTAATATATTTAAAGAAGAAAAATTAGAATCACCA

GTTGTCTTCGCTCCCGCTAACATCACCAC

TGAAGTCAAGTCCGTTGAAATGCACCACGAAGCTCTCGTTGAAGCCGTCCCCGGTGACAA

CGTTGGTTTCAACGTAAAGAACGTCTCCGTCAAGGAATTGCGTCGTGGTTACGTCGCTGG

TGACTCCAAGGCCAACCCCCCCAAGGGCGCCGCTGACTTCACCGCTCAAGTCATCGTCTT

GAACCACCCTGGTCAAATCTCCAACGGTTACACCCCCGTTTTGGATTGTCACACCGCTCA

CATTGCTTGCAAATTCGCCGAAATCAAGGAGAAGGTCGATGGATGCACGCAGTACCCTAAGTCCGGTTCAGTGCTTTGAGGGCAGCGGTGG

AAGTGGTTCATCGGGAAATTTTACTTCGGGCAGTAACTTAAATATGGGCAGTGTGACTAA

TACCAGCAACACCGGTACTGGTACTTCCTCAGGAAGTGTTCCCATGGTCACGTTGACGGA

ATCTCTACTAAATAAACATAACGATGAAATGGAAAAGTTTATGCTTAAGAAGCATAGAGA

ATCTCGAGGACGAAGTGGCGAGAAGAACAAAAAGAATTCTGAAAAGATAATGGAGTACAC

TGGCCCGGGACACGGAATCAAAAGAGTTGGTTCACA

>3ITLse

AGAATTCTAATTCGAGCTGAATTAGGACATCCTGG
AGCTTTAATTGGAGATGATCAAATTTATAATGTAATTGTTACAGCTCATGCTTTTATTAT
AATTTTTTTTATAGTAATGCCAATTATAATTGGAGGATTTGGAAATTGATTAGTTCCATT
AATACTAGGAGCTCCAGATATAGCATTCCCTCGAATAAATAATATAAGTTTTTGACTTTT
ACCTCCTGCATTAACTTTATTATTAGTTAGTAGTATAGTAGAAAACGGAGCTGGAACAGG
ATGAACAGTTTACCCTCCTCTATCTTCTAATATTGCTCATGGAGGAGCTTCTGTTGATTT
AGCTATTTTCTCTCTTCATTTAGCAGGAATTTCTTCAATTTTAGGAGCTGTAAATTTTAT
TACTACAGTTATTAATATACGATCAACAGGAATTACTTTTGATCGAATACCTTTATTTGT
TTGATCAGTAGTAATTACAGCTTTATTACTTTTATTATCATTACCAGTATTAGCAGGAGC
TATTACAATACTTTTAACAGACCGAAATCTTAATACATCATTCTTTGACCCTGCAGGAGG
AGGAGATCCAATTTTATACCAACATTTATTTAATAATTAATAATTCCATAAGTAGAAATATAAGGTATAAACCACATAGAAC

CTAAAAAATAAGAAAAATTATAATTATTTAAAGCCTTATTATAAAAAAATAAAGAAACAT

TAGAAATTATATAACCTATAATACCCCCAACAATACAAACAAATAATGTTAATAATTTTA

AATAAGAAGGAAGAACAACTACTATAGGAGTAGGAAAAATCAATCATCTTAACATTCTCC

CACCAAAAATTCTTAAAATTAATAAACCTATTATACTTTTCAATATTACTCAACCTTCAT

CATTTAATATATTTAAAGAAGAAAAATTAGAATCACCAGTTGTCTTCGCTCCCGCTAACATCACCACTGAAG

TCAAGTCCGTTGAAATGCACCACGAAGCTCTCGTTGAAGCCGTCCCCGGTGACAACGTTG

GTTTCAACGTAAAGAACGTCTCCGTCAAGGAATTGCGTCGTGGTTACGTCGCTGGTGACT

CCAAGGCCAACCCCCCCAAGGGCGCCGCTGACTTCACCGCTCAAGTCATCGTCTTGAACC

ACCCTGGTCAAATCTCCAACGGTTACACCCCCGTTTTGGATTGTCACACCGCTCACATTG

CTTGCAAATTCGCCGAAATCAAGGAGAAGGTCGATGGATGCACGCAGTACCCTAAGTCCGGTTCAGTGCTTTGAGGGCAGCGGTGGAAG

TGGTTCATCGGGAAATTTTACTTCGGGCAGTAACTTAAATATGGGCAGTGTGACTAATAC

CAGCAACACCGGTACTGGTACTTCCTCAGGAAGTGTTCCCATGGTCACGTTGACCGAATC

TCTACTAAATAAACATAACGATGAAATGGAAAAGTTTATGCTTAAGAAGCATAGAGAATC

TCGAGGACGAAGTGGCGAGAAGAACAAAAAGAATTCTGAAAAGATAATGGAATACACTGG

CCCGGGACACGGAATCAAAAGAGTTGGTTCACA

>4ITLse

AGAATTCTAATTCGAGCTGAATTAGGACATCC
TGGAGCTTTAATTGGAGATGATCAAATTTATAATGTAATTGTTACAGCTCATGCTTTTAT
TATAATTTTTTTTATAGTAATGCCAATTATAATTGGAGGATTTGGAAATTGATTAGTTCC
ATTAATACTAGGAGCTCCAGATATAGCATTCCCTCGAATAAATAATATAAGTTTTTGACT
TTTACCTCCTGCATTAACTTTATTATTAGTTAGTAGTATAGTAGAAAACGGAGCTGGAAC
AGGATGAACAGTTTACCCTCCTCTATCTTCTAATATTGCTCATGGAGGAGCTTCTGTTGA
TTTAGCTATTTTCTCTCTTCATTTAGCAGGAATTTCTTCAATTTTAGGAGCTGTAAATTT
TATTACTACAGTTATTAATATACGATCAACAGGAATTACTTTTGATCGAATACCTTTATT
TGTTTGATCAGTAGTAATTACAGCTTTATTACTTTTATTATCATTACCAGTATTAGCAGG
AGCTATTACAATACTTTTAACAGACCGAAATCTTAATACATCATTCTTTGACCCTGCAGG
AGGAGGAGATCCAATTTTATACCAACATTTATTTAATAATTAATAATTCCATAAGTAGAAATATAAGAGTATAAACCA

CATAGAACCTAAAAAATAAGAAAAATTATAATTATTTAAAGCCTTATTATAAAAAAATAA

AGAAACATTAGAAATTATATAACCTATAATACCCCCAACAATACAAACAAATAATGTTAA

TAATTTTAAATAAGAAGGAAGAACAACTACTATAGGGGTAGGAAAAATCAATCATCTTAA

CATTCTCCCACCAAAAATTCTTAAAATTAATAAACCTATTATACTTTTCAATATTACTCA

ACCTTCATCATTTAATATATTTAAAGAAGAAAAATTAGAATCACCA

GTTGTTTTCGCTCCCGCTAACATCACCACT

GAAGTCAAGTCCGTTGAAATGCACCACGAAGCTCTCGTTGAAGCCGTCCCCGGTGACAAC

GTTGGTTTCAACGTAAAGAACGTCTCCGTCAAGGAATTGCGTCGTGGTTACGTCGCTGGT

GACTCCAAGGCTAACCCCCCCAAGGGCGCCGCTGACTTCACCGCTCAAGTCATCGTCTTG

AACCACCCTGGTCAAATCTCCAACGGTTACACCCCCGTTTTGGATTGTCACACCGCTCAC

ATTGCTTGCAAATTCGCCGAAATCAAGGAGAAGGTCGATCGATGCACGCAGTACCCTAAGTCCGGTTCAGTGCTTTGAGGGCAGCGGTGGAAG

TGGTTCATCGGGAAATTTTACTTCGGGCAGTAACTTAAATATGGGCAGTGTGACTAATAC

CAGCAACACCGGTACTGGTACTTCCTCAGGAAGTGTTCCCATGGTCACGTTGACCGAATC

TCTACTAAATAAACATAACGATGAAATGGAAAAGTTTATGCTTAAGAAGCATAGAGAATC

TCGAGGACGAAGTGGCGAGAAGAACAAAAAGAATTCTGAAAAGATAATGGAGTACACTGG

CCCGGGACACGGAATCAAAAGAGTTGGTTCACA

>5ITLse

AGAATTCTAATTCGAGCTGAATTAGGACATCCTGGAGC
TTTAATTGGAGATGATCAAATTTATAATGTAATTGTTACAGCTCATGCTTTTATTATAAT
TTTTTTTATAGTAATGCCAATTATAATTGGAGGATTTGGAAATTGATTAGTTCCATTAAT
ACTAGGAGCTCCAGATATAGCATTCCCTCGAATAAATAATATAAGTTTTTGACTTTTACC
TCCTGCATTAACTTTATTATTAGTTAGTAGTATAGTAGAAAACGGAGCTGGAACAGGATG
AACAGTTTACCCTCCTCTATCTTCTAATATTGCTCATGGAGGAGCTTCTGTTGATTTAGC
TATTTTCTCTCTTCATTTAGCAGGAATTTCTTCAATTTTAGGAGCTGTAAATTTTATTAC
TACAGTTATTAATATACGATCAACAGGAATTACTTTTGATCGAATACCTTTATTTGTTTG
ATCAGTAGTAATTACAGCTTTATTACTTTTATTATCATTACCAGTATTAGCAGGAGCTAT
TACAATACTTTTAACAGACCGAAATCTTAATACATCATTCTTTGACCCTGCAGGAGGAGG
AGATCCAATTTTATACCAACATTTATTTAATAATTAATAATTCCATAAGTAGAAATATAAGGTATAAACCACATAGAACC

TAAAAAATAAGAAAAATTATAATTATTTAAAGCCTTATTATAAAAAAATAAAGAAACATT

AGAAATTATATAACCTATAATACCCCCAACAATACAAACAAATAATGTTAATAATTTTAA

ATAAGAAGGAAGAACAACTACTATAGGAGTAGGAAAAATCAATCATCTTAACATTCTCCC

ACCAAAAATTCTTAAAATTAATAAACCTATTATACTTTTCAATATTACTCAACCTTCATC

ATTTAATATATTTAAAGAAGAAAAATTAGAATCACCAGTTGTCTTCGCTCCCGCTAACATCACCACTGAAGTCAAGTCCGTTGA

AATGCACCACGAAGCTCTCGTTGAAGCCGTCCCCGGTGACAACGTTGGTTTCAACGTAAA

GAACGTCTCCGTCAAGGAATTGCGTCGTGGTTACGTCGCTGGTGACTCCAAGGCTAACCC

CCCCAAGGGCGCCGCTGACTTCACCGCTCAAGTCATCGTCTTGAACCACCCTGGTCAAAT

CTCCAACGGTTACACCCCCGTTTTGGATTGTCACACCGCTCACATTGCTTGCAAATTCGC

CGAAATCAAGGAGAAGGTCGAT GGATGCACGCAGTACCCTAAGTCCGGTTCAGTGCTTTGAGGGCAGCGGTGGAAG

TGGTTCATCGGGAAATTTTACTTCGGGCAGTAACTTAAATATGGGCAGTGTGACTAATAC

CAGCAACACCGGTACTGGTACTTCCTCAGGAAGTGTTCCCATGGTCACGTTGACGGAATC

TCTACTAAATAAACATAACGATGAAATGGAAAAGTTTATGCTTAAGAAGCATAGAGAATC

TCGAGGACGAAGTGGCGAGAAGAACAAAAAGAATTCTGAAAAGATAATGGAGTACACTGG

CCCGGGACACGGAATCAAAAGAGTTGGTTCACA

>6ITLse

AGAATTCTAATTCGAGCTGAATTAGGACATCCTGGAGC
TTTAATTGGAGATGATCAAATTTATAATGTAATTGTTACAGCTCATGCTTTTATTATAAT
TTTTTTTATAGTAATGCCAATTATAATTGGAGGATTTGGAAATTGATTAGTTCCATTAAT
ACTAGGAGCTCCAGATATAGCATTCCCTCGAATAAATAATATAAGTTTTTGACTTTTACC
TCCTGCATTAACTTTATTATTAGTTAGTAGTATAGTAGAAAACGGAGCTGGAACAGGATG
AACAGTTTACCCTCCTCTATCTTCTAATATTGCTCATGGAGGAGCTTCTGTTGATTTAGC
TATTTTCTCTCTTCATTTAGCAGGAATTTCTTCAATTTTAGGAGCTGTAAATTTTATTAC
TACAGTTATTAATATACGATCAACAGGAATTACTTTTGATCGAATACCTTTATTTGTTTG
ATCAGTAGTAATTACAGCTTTATTACTTTTATTATCATTACCAGTATTAGCAGGAGCTAT
TACAATACTTTTAACAGACCGAAATCTTAATACATCATTCTTTGACCCTGCAGGAGGAGG
AGATCCAATTTTATACCAACATTTATTTAATAATTAATAATTCCATAAGTAGAAATATAAGGTATAAACCACATAGAA

CCTAAAAAATAAGAAAAATTATAATTATTTAAAGCCTTATTATAAAAAAATAAAGAAACA

TTAGAAATTATATAACCTATAATACCCCCAACAATACAAACAAATAATGTTAATAATTTT

AAATAAGAAGGAAGAACAACTACTATAGGAGTAGGAAAAATCAATCATCTTAACATTCTC

CCACCAAAAATTCTTAAAATTAATAAACCTATTATACTTTTCAATATTACTCAACCTTCA

TCATTTAATATATTTAAAGAAGAAAAATTAGAATCACCAGTTGTCTTCGCTCCCGCTAACATCACCACTGAAGTCAAGT

CCGTTGAAATGCACCACGAAGCTCTCGTTGAAGCCGTCCCCGGTGACAACGTTGGTTTCA

ACGTAAAGAACGTCTCCGTTAAGGAATTGCGTCGTGGTTACGTCGCTGGTGACTCCAAGG

CCAACCCCCCCAAGGGCGCCGCTGACTTCACCGCTCAAGTCATCGTCTTGAACCACCCTG

GTCAAATCTCCAACGGTTACACCCCCGTTTTGGATTGTCACACCGCTCACATTGCTTGCA

AATTCGCCGAAATCAAGGAGAAGGTCGATCGATGCACGCAGTACCCTAAGTCCGGTTCAGTGCTTTGAGGGCAGCGGTGGAAG

TGGTTCGTCGGGAAATTTTACTTCGGGCAGTAACTTAAATATGGGCAGTGTGACTAATAC

CAGCAACACCGGTACTGGTACTTCCTCTGGAAGTGTTCCCATGGTCACGTTGACCGAATC

TCTACTAAATAAACATAACGATGAAATGGAAAAGTTTATGCTTAAGAAGCATAGAGAATC

TCGAGGACGAAGTGGCGAGAAGAACAAAAAGAATTCTGAAAAGATAATGGAGTACACTGG

CCCGGGACACGGAATCAAAAGAGTTGGTTCACA

>7ITLse

AGAATTCTAATTCGAGCTGAATTAGGACATC
CTGGAGCTTTAATTGGAGATGATCAAATTTATAATGTAATTGTTACAGCTCATGCTTTTA
TTATAATTTTTTTTATAGTAATGCCAATTATAATTGGAGGATTTGGAAATTGATTAGTTC
CATTAATACTAGGAGCTCCAGATATAGCATTCCCTCGAATAAATAATATAAGTTTTTGAC
TTTTACCTCCTGCATTAACTTTATTATTAGTTAGTAGTATAGTAGAAAACGGAGCTGGAA
CAGGATGAACAGTTTACCCTCCTCTATCTTCTAATATTGCTCATGGAGGAGCTTCTGTTG
ATTTAGCTATTTTCTCTCTTCATTTAGCAGGAATTTCTTCAATTTTAGGAGCTGTAAATT
TTATTACTACAGTTATTAATATACGATCAACAGGAATTACTTTTGATCGAATACCTTTAT
TTGTTTGATCAGTAGTAATTACAGCTTTATTACTTTTATTATCATTACCAGTATTAGCAG
GAGCTATTACAATACTTTTAACAGACCGAAATCTTAATACATCATTCTTTGACCCTGCAG
GAGGAGGAGATCCAATTTTATACCAACATTTATTTAATAATTAATAATTCCATAAGTAGAAATATAAGGTATAAACCACATAG

AACCTAAAAAATAAGAAAAATTATAATTATTTAAAGCCTTATTATAAAAAAATAAAGAAA

CATTAGAAATTATATAACCTATAATACCCCCAACAATACAAACAAATAATGTTAATAATT

TTAAATAAGAAGGAAGAACAACTACTATAGGAGTAGGAAAAATCAATCATCTTAACATTC

TCCCACCAAAAATTCTTAAAATTAATAAACCTATTATACTTTTCAATATTACTCAACCTT

CATCATTTAATATATTTAAAGAAGAAAAATTAGAATCACCGTTGTCTTCGCTCCCGCTAACATCACCACTGAAGTCAAGTCCGTTG

AAATGCACCACGAAGCTCTCGTTGAAGCCGTCCCCGGTGACAACGTTGGTTTCAACGTAA

AGAACGTCTCCGTCAAGGAATTGCGTCGTGGTTACGTCGCTGGTGACTCCAAGGCCAACC

CCCCCAAGGGCGCCGCTGACTTCACCGCTCAAGTCATCGTCTTGAACCACCCTGGTCAAA

TCTCCAACGGTTACACCCCCGTTTTGGATTGTCACACCGCTCACATTGCTTGCAAATTCG

CCGAAATCAAGGAGAAGGTCGATGGATGCACGCAGTACCCTAAGTCCGGTTCAGTGCTTTGAGGGCAGCGGTGGAAGTGGT

TCATCGGGAAATTTTACTTCGGGCAGTAACTTAAATATGGGCAGTGTGACTAATACCAGC

AACACCGGTACTGGTACTTCCTCAGGAAGTGTTCCCATGGTCACGTTGACCGAATCTCTA

CTAAATAAACATAACGATGAAATGGAAAAGTTTATGCTTAAGAAGCATAGAGAATCTCGA

GGACGAAGTGGCGAGAAGAACAAAAAGAATTCTGAAAAGATAATGGAATACACTGGCCCG

GGACACGGAATCAAAAGAGTTGGTTCACA

>8ITLse

AGAATTCTAATTCGAGCTGAATTAGGACATCCTGGAG
CTTTAATTGGAGACGATCAAATTTATAATGTAATTGTTACAGCTCATGCTTTTATTATAA
TTTTTTTTATAGTAATGCCAATTATAATTGGAGGATTTGGAAATTGATTAGTTCCATTAA
TACTAGGAGCTCCAGATATAGCATTCCCTCGAATAAATAATATAAGTTTTTGACTTTTAC
CTCCTGCATTAACTTTATTATTAGTTAGTAGTATAGTAGAAAACGGAGCTGGAACAGGAT
GAACAGTTTACCCTCCTCTATCTTCTAATATTGCTCATGGAGGAGCTTCTGTTGATTTAG
CTATTTTCTCTCTTCATTTAGCAGGAATTTCTTCAATTTTAGGAGCTGTAAATTTTATTA
CTACAGTTATTAATATACGATCAACAGGAATTACTTTTGATCGAATACCTTTATTTGTTT
GATCAGTAGTAATTACAGCTTTATTACTTTTATTATCATTACCAGTATTAGCAGGAGCTA
TTACAATACTTTTAACAGACCGAAATCTTAATACATCATTCTTTGACCCTGCAGGAGGAG
GAGATCCAATTTTATACCAACATTTATTTAATAATTAATAATTCCATAAGTAGAAATATAAGAGT

ATAAACCACATAGAACCTAAAAAATAAGAAAAATTATAATTATTTAAAGCCTTATTATAA

AAAAATAAAGAAACATTAGAAATTATATAACCTATAATACCCCCAACAATACAAACAAAT

AATGTTAATAATTTTAAATAAGAAGGAAGAACAACTACTATAGGAGTAGGAAAAATCAAT

CATCTTAACATTCTCCCACCAAAAATTCTTAAAATTAATAAACCTATTATACTTTTCAAT

ATTACTCAACCTTCATCATTTAATATATTTAAAGAAGAAAAATTAGAATCACCAGTTGTCTTCGCCCCCGCTAACATCACCACTGAAGTCAAGTC

CGTTGAAATGCACCACGAAGCTCTCGTTGAAGCCGTCCCCGGTGACAACGTTGGTTTCAA

CGTAAAGAACGTCTCCGTCAAGGAATTGCGTCGTGGTTACGTCGCTGGTGACTCCAAGGC

CAACCCCCCCAAGGGCGCCGCTGACTTCACCGCTCAAGTCATCGTCTTGAACCACCCTGG

TCAAATCTCCAACGGTTACACCCCCGTTTTGGATTGTCACACCGCTCACATTGCTTGCAA

ATTCGCCGAAATCAAGGAGAAGGTCGATGGATGCACGCAGTACCCTAAGTCCGGTTCAGTGCTTTGAGGGCAGCGGTGGAAG

TGGTTCATCGGGAAATTTTACTTCGGGCAGTAACTTAAATATGGGCAGTGTGACTAATAC

CAGCAACACCGGTACTGGTACTTCCTCTGGAAGTGTTCCCATGGTCACGTTGACCGAATC

TCTACTAAATAAACATAACGATGAAATGGAAAAGTTTATGCTTAAGAAGCATAGAGAATC

TCGAGGACGAAGTGGCGAGAAGAACAAAAAGAATTCTGAAAAGATAATGGAGTACACTGG

CCCGGGACACGGAATCAAAAGAGTTGGTTCACA

>9ITLse

AGAATTCTAATTCGAGCTGAATTAGGACATCCT
GGAGCTTTAATTGGAGATGATCAAATTTATAATGTAATTGTTACAGCTCATGCTTTTATT
ATAATTTTTTTTATAGTAATGCCAATTATAATTGGAGGATTTGGAAATTGATTAGTTCCA
TTAATACTAGGAGCTCCAGATATAGCATTCCCTCGAATAAATAATATAAGTTTTTGACTT
TTACCTCCTGCATTAACTTTATTATTAGTTAGTAGTATAGTAGAAAACGGAGCTGGAACA
GGATGAACAGTTTACCCTCCTCTATCTTCTAATATTGCTCATGGAGGAGCTTCTGTTGAT
TTAGCTATTTTCTCTCTTCATTTAGCAGGAATTTCTTCAATTTTAGGAGCTGTAAATTTT
ATTACTACAGTTATTAATATACGATCAACAGGAATTACTTTTGATCGAATACCTTTATTT
GTTTGATCAGTAGTAATTACAGCTTTATTACTTTTATTATCATTACCAGTATTAGCAGGA
GCTATTACAATACTTTTAACAGACCGAAATCTTAATACATCATTCTTTGACCCTGCAGGA
GGAGGAGATCCAATTTTATACCAACATTTATTTAATAATTAATAATTCCATAAGTAGAAATATAAGGTATAAACCAC

ATAGAACCTAAAAAATAAGAAAAATTATAATTATTTAAAGCCTTATTATAAAAAAATAAA

GAAACATTAGAAATTATATAACCTATAATACCCCCAACAATACAAACAAATAATGTTAAT

AATTTTAAATAAGAAGGAAGAACAACTACTATAGGAGTAGGAAAAATCAATCATCTTAAC

ATTCTCCCACCAAAAATTCTTAAAATTAATAAACCTATTATACTTTTCAATATTACTCAA

CCTTCATCATTTAATATATTTAAAGAAGAAAAATTAGAATCACCA

GTTGTCTTCGCTCCCGCTAACATCACCACTGAAGTCAAGT

CCGTTGAAATGCACCACGAAGCTCTCGTTGAAGCCGTCCCCGGTGACAACGTTGGTTTCA

ACGTAAAGAACGTCTCCGTCAAGGAATTGCGTCGTGGTTACGTCGCTGGTGACTCCAAGG

CCAACCCCCCCAAGGGCGCCGCTGACTTCACCGCTCAAGTCATCGTCTTGAACCACCCTG

GTCAAATCTCCAACGGTTACACCCCCGTTTTGGATTGTCACACCGCTCACATTGCTTGCA

AATTCGCCGAAATCAAGGAGAAGGTCGAT GGATGCACGCAGTACCCTAAGTCCGGTTCAGTGCTTTGAGGGCAGCGGTGGAAG

TGGTTCATCGGGAAATTTTACTTCGGGCAGTAACTTAAATATGGGCAGTGTGACTAATAC

CAGCAACACCGGTACTGGTACTTCCTCAGGAAGTGTTCCCATGGTCACGTTGACGGAATC

TCTACTAAATAAACATAACGATGAAATGGAAAAGTTTATGCTTAAGAAGCATAGAGAATC

TCGAGGACGAAGTGGCGAGAAGAACAAAAAGAATTCTGAAAAGATAATGGAGTACACTGG

CCCGGGACACGGAATCAAAAGAGTTGGTTCACA

>1BGLse

AGAATTCTAATTCGAGCTGAATTAGGACATCCTGG
AGCTTTAATTGGAGATGATCAAATTTATAATGTAATTGTTACAGCTCATGCTTTTATTAT
AATTTTTTTTATAGTAATGCCAATTATAATTGGAGGATTTGGAAATTGATTAGTTCCATT
AATACTAGGAGCTCCAGATATAGCATTCCCTCGAATAAATAATATAAGTTTTTGACTTTT
ACCTCCTGCATTAACTTTATTATTAGTTAGTAGTATAGTAGAAAACGGAGCTGGAACAGG
ATGAACAGTTTACCCTCCTCTATCTTCTAATATTGCTCATGGAGGAGCTTCTGTTGATTT
AGCTATTTTCTCTCTTCATTTAGCAGGAATTTCTTCAATTTTAGGAGCTGTAAATTTTAT
TACTACAGTTATTAATATACGATCAACAGGAATTACTTTTGATCGAATACCTTTATTTGT
TTGATCAGTAGTAATTACAGCTTTATTACTTTTATTATCATTACCAGTATTAGCAGGAGC
TATTACAATACTTTTAACAGACCGAAATCTTAATACATCATTCTTTGACCCTGCAGGAGG
AGGAGATCCAATTTTATACCAACATTTATTT
AATAATTAATAATTCCATAAGTAGAAATATAAGGTATAAACCACATAGA

ACCTAAAAAATAAGAAAAATTATAATTATTTAAAGCCTTATTATAAAAAAATAAAGAAAC

ATTAGAAATTATATAACCTATAATACCCCCAACAATACAAACAAATAATGTTAATAATTT

TAAATAAGAAGGAAGAACAACTACTATAGGAGTAGGAAAAATCAATCATCTTAACATTCT

CCCACCAAAAATTCTTAAAATTAATAAACCTATTATACTTTTCAATATTACTCAACCTTC

ATCATTTAATATATTTAAAGAAGAAAAATTAGAATCACCA

GTTGTCTTCGCTCCCG

CTAACATCACCACTGAAGTCAAGTCCGTTGAAATGCACCACGAAGCTCTCGTTGAAGCCG

TCCCCGGTGACAACGTTGGTTTCAACGTAAAGAACGTCTCCGTCAAGGAATTGCGTCGTG

GTTACGTCGCTGGTGACTCCAAGGCCAACCCCCCCAAGGGCGCCGCTGACTTCACCGCTC

AAGTCATCGTCTTGAACCACCCTGGTCAAATCTCCAACGGTTACACCCCCGTTTTGGATT

GTCACACCGCTCACATTGCTTGCAAATTCGCCGAAATCAAGGAGAAGGTCGAT CGATGCACGC

AGTACCCTAAGTCCGGTTCAGTGCTTTGAGGGCAGCGGTGGAAGTGGTTCATCGGGAAAT

TTTACTTCGGGCAGTAACTTAAATATGGGCAGTGTGACTAATACCAGCAACACCGGTACT

GGTACTTCCTCAGGAAGTGTTCCCATGGTCACGTTGACCGAATCTCTACTAAATAAACAT

AACGATGAAATGGAAAAGTTTATGCTTAAGAAGCATAGAGAATCTCGAGGACGAAGTGGC

GAGAAGAACAAAAAGAATTCTGAAAAGATAATGGAGTACACTGGCCCGGGACACGGAATC

AAAAGAGTTGGTTCACA

>2BGCvi

AGAATTCTAATTCGAGCCGAACTAGGACA
TCCTGGAGCATTAATTGGAGATGACCAAATTTATAATGTAATTGTTACAGCTCATGCTTT
TATTATAATTTTTTTTATAGTAATACCAATTATAATTGGAGGATTTGGTAATTGATTAGT
CCCTTTAATATTAGGAGCTCCAGATATAGCCTTCCCTCGGATAAACAATATAAGTTTCTG
ACTTTTACCTCCTGCATTAACTTTACTATTAGTAAGTAGTATAGTAGAAAACGGAGCTGG
AACAGGATGAACTGTTTACCCACCTTTATCTTCTAATATCGCTCATGGAGGAGCTTCTGT
TGATTTAGCTATTTTTTCTTTACACTTAGCAGGAATTTCTTCAATTTTAGGAGCTGTAAA
TTTTATTACTACAGTTATTAATATACGATCAACAGGAATTACATTCGACCGAATACCATT
ATTTGTTTGATCTGTAGTAATTACAGCTTTATTACTTTTATTATCTTTACCAGTATTAGC
AGGTGCTATTACTATATTATTAACAGATCGAAATCTTAATACATCATTCTTTGATCCAGC
AGGAGGAGGAGACCCAATCTTGTACCAACATTTATTT
AATAATTAATAATTCCATATGTAGAAATATAAGGTATAAATCATATT

GAACCTAAAAAGTAAGAAGAATTATAATTATTTAAAGCCTTATTATAAAAAAATAAAGAA

ATATTAGAAATTAAATAACCTCTAACTCCTCCTACAATACAAACAAATAAAGTTAATAAT

TTTAAATAAGAAGGAAGAACTACTACTACTGGTGTAGGAAAAATTAATCAACTTAATATA

CTACCCCCAAAAATTCTCAAAATTAATAGTCCTATTATACTTTTTAATATAACTCAACCT

TCATCATTTAGTATATTTAAAGAAGAAAAATTTGAATCCCCA

GTTGTCTTCGCTCCCGCTAACATCACCACTGAAGTCAAGTCCG

TAGAAATGCACCACGAAGCTCTCGTTGAAGCTGTTCCCGGTGACAACGTTGGTTTCAACG

TTAAGAACGTCTCCGTCAAGGAATTGCGTCGTGGTTACGTCGCTGGTGACTCCAAGGCTA

GCCCCCCCAAGGGTGCTGCTGACTTCACCGCTCAAGTCATCGTCTTGAACCACCCTGGTC

AAATCTCCAACGGTTACACTCCCGTTTTGGATTGTCACACCGCTCACATTGCTTGCAAAT

TCGCCGAAATCAAGGAGAAGGTCGATTGAAGCACGCAGTACCCTGAGTCCGGTTCAGTGCTTTGAGGGTAGCGGTGGTAG

TGGATCCTCAGGAAATTTTACTTCGGGTAGCAACTTGAATATGGGCAGTGTTACTAATAC

AAGCAATACCGGTACAGGCACTTCATCGGGAAGTGTACCCATGGTTACGTTGACGGAATC

TCTACTAAATAAACACAACGATGAAATGGAAAAGTTTATGCTAAAGAAACACAGAGAATC

TCGCGGACGAAGTGGTGAGAAGAATAAAAAGAACTCGGAAAAGATAATGGAGTATACTGG

TCCTGGGCATGGAATAAAGAGAGTTGGCTCACA

>3BGCym

AGAATTTTAATTCGAGCCGAACTAGGACACCCTGGAGCATTAAT
TGGTGATGACCAAATCTATAATGTAATTGTTACAGCTCATGCTTTTATTATAATTTTTTT
TATAGTAATACCAATTATAATTGGAGGGTTTGGTAATTGATTAGTCCCTTTAATGTTAGG
AGCCCCAGATATAGCTTTCCCTCGGATAAACAATATAAGTTTCTGACTTTTACCTCCTGC
ATTAACTTTACTATTAGTAAGTAGTATAGTGGAAAACGGAGCTGGAACAGGATGAACTGT
TTATCCACCTTTATCATCTAATATTGCTCATGGAGGAGCTTCTGTTGATTTAGCTATTTT
TTCTTTACATTTAGCGGGAATTTCTTCAATTTTAGGAGCTGTAAATTTTATTACAACAGT
TATTAATATACGATCAACAGGAATTACTTTTGACCGAATACCCCTATTCGTTTGATCAGT
AGTAATTACAGCTTTATTACTTTTACTATCTTTACCTGTTCTAGCTGGGGCTATTACTAT
ATTATTAACAGACCGAAACCTTAATACTTCATTCTTCGACCCAGCAGGAGGAGGAGACCC
AATTCTATACCAACACTTATTTAATAATTAATAATTCCGTATGTAGAAATATAAGGTATAAATCACATT

GACCCTAAAAAGTAAGAAGAGTTATAATTATTTAAAGCCTTATTGTAAAAAAATAAAGAA

ATGTTAGAAATTAAATAACCTCTAACTCCCCCTACAATACAAACAAATAAAGTTAATAAT

TTTAAATAATAAGGAAGAACCACTACTACAGGGGTAGGAAAAATCAATCAACTTAATATT

CTACCTCCAAAGATTCTCAAAATTAATAACCCTATTATACTTTTTAATATAACTCAACCT

TCATCATTTAATATATTTAAAGAAGAAAAATTAGAATCCCCAGTTGTCTTCGCTCCCGCTAACATCACCACTGAAGTCAAGTCCGTAGAAATGCACCACGAAGCTCTCGTTGAAGCCGTTCCCGGTGACAA

CGTTGGTTTCAACGTTAAGAACGTCTCCGTCAAGGAATTGCGTCGTGGTTACGTCGCTGG

TGACTCCAAGGCTAGCCCCCCCAAGGGTGCTGCTGACTTCACCGCTCAAGTCATCGTCTT

GAACCACCCTGGTCAAATCTCCAACGGTTACACTCCCGTTTTGGATTGTCACACCGCTCA

CATTGCTTGCAAATTCGCCGAAATCAAGGAGAAGGTCGATTGAAGCACGCAGTACCCTGAGTCCGGTTCAGTGCTTTGAGGGTAGCGGTGGTAGTGGAT

CCTCAGGAAATTTTACTTCGGGCAGCAACTTGAATATGGGCAGTGTTACTAATACAAGCA

ATACCGGCACAGGCACTTCATCGGGAAGTGTACCCATGGTTACGTTGACGGAATCTCTAC

TAAATAAACATAACGATGAAATGGAAAAGTTTATGCTAAAGAAACACAGAGAATCTCGCG

GACGAAGTGGTGAGAAGAATAAAAAGAACTCGGAAAAGATAATGGAGTATACTGGTCCTG

GACATGGAGTAAAGAGAGTTGGCTCACA

>CvoUK

AGAATTTTAATTCGAGCTGAACTAGGGCAT
CCTGGAGCATTAATTGGAGATGATCAAATTTATAATGTAATTGTTACAGCTCATGCTTTT
ATTATAATTTTTTTTATAGTAATACCAATTATAATTGGAGGGTTTGGAAATTGATTAGTT
CCTTTAATATTAGGAGCCCCAGATATAGCATTCCCTCGAATAAATAATATAAGTTTCTGA
CTTTTACCTCCTGCATTAACTTTACTATTAGTAAGTAGTATAGTAGAAAACGGAGCTGGA
ACTGGATGAACTGTTTATCCACCTTTATCTTCTAATATTGCACATGGAGGAGCTTCTGTT
GATTTAGCTATTTTTTCTTTACATTTAGCAGGAATTTCTTCAATTTTAGGAGCTGTAAAT
TTTATTACTACAGTTATTAATATACGATCAACAGGTATTACCTTCGACCGAATACCATTA
TTTGTTTGATCAGTAGTAATTACAGCCTTATTACTTTTATTATCTTTACCAGTATTAGCA
GGAGCTATTACTATATTATTAACAGATCGAAATCTTAATACTTCATTCTTTGACCCAGCA
GGAGGAGGAGATCCAATTTTATACCAACACTTATTTAGTAATTAATAATTCCGTATGTAGAAATATAAGGCATAAACCACATTGAC

CCTAAAAAATAAGAAGAATTATAGTTATTTAAAGCCTTATTATAAAAAAATAAAGAAATA

TTAGAAATTAAATACCCTCTAATTCCTCCTACAATACAAACAAATAAAGTTAATAACTTT

AAATAAGAAGGTAGAACTACTACTACTGGTGTAGGAAAAATTAATCAACTTAACATTCTA

CCCCCAAAAATTCTTAAAATTAATAACCCTATTATACTCTTTAACATAACTCAACCTTCA

TCATTTAATATATTTAAAGAAGAAAAATTTGAATCCCCAGTTGTCTTCGCTCCCGCTAACATCACCACTGAAGTCAAAT

CCGTAGAAATGCACCACGAAGCTCTCGTTGAAGCCGTCCCCGGTGACAACGTTGGTTTCA

ACGTTAAGAACGTCTCCGTCAAGGAATTGCGTCGTGGTTACGTCGCTGGTGACTCCAAGG

CTAGCCCCCCCAAGGGTGCTGCTGACTTCACCGCTCAAGTCATCGTCTTGAACCACCCTG

GTCAAATCTCCAACGGTTACACTCCCGTTTTGGATTGTCACACCGCTCACATTGCTTGCA

AATTCGCCGAAATCAAGGAGAAGGTCGATTGAAGCACGCAGTACCCTGAGTCCGGTTCAGTGCTTTGAGGGTAGCGGTGGTAGTG

GATCCTCAGGAAATTTTACTTCGGGTAGCAACTTGAATATGGGCAGTGTTACTAATACAA

GCAATACCGGTACAGGCACTTCATCGGGAAGTGTACCCATGGTTACGTTGACGGAATCTC

TACTAAATAAACACAACGATGAAATGGAAAAGTTTATGCTAAAGAAACACAGAGAATCTC

GTGGACGAAGTGGTGAGAAGAATAAAAAGAACTCGGAAAAGATAATGGAGTATACTGGTC

CTGGGCATGGAATAAAGAGAGTTGGCTCACA

>ITMO3Cvi

AGAATTCTAATTCGAGCCGAACTAGGACATCCTG
GAGCATTAATTGGAGATGACCAAATTTATAATGTAATTGTTACAGCTCATGCTTTTATTA
TAATTTTTTTTATAGTAATACCAATTATAATTGGAGGATTTGGTAATTGATTAGTTCCTT
TAATATTAGGAGCTCCAGATATAGCATTCCCTCGGATAAACAATATAAGTTTCTGACTTT
TACCTCCTGCATTAACTTTACTATTAGTAAGTAGTATAGTAGAAAACGGAGCTGGAACAG
GATGAACTGTTTACCCACCTTTATCTTCTAATATCGCTCATGGAGGAGCTTCTGTTGATT
TAGCTATTTTTTCTTTACACTTGGCAGGAATTTCTTCAATTTTAGGAGCTGTAAATTTTA
TTACTACAGTTATTAATATACGATCAACAGGAATTACATTCGACCGAATACCATTATTTG
TTTGATCTGTAGTAATTACAGCTTTATTACTTTTATTATCTTTACCAGTATTAGCAGGTG
CTATTACTATATTATTAACAGATCGAAATCTTAATACATCATTCTTTGACCCAGCAGGAG
GAGGAGACCCAATCTTGTACCAACATTTATTTAATAATTAATAATTCCATATGTAGAAATATAAGGTATAAATCATATTG

AACCTAAAAAGTAAGAAGAATTATAATTATTTAAAGCCTTATTATAAAAAAATAAAGAAA

TATTAGAAATTAAATAACCTCTAACTCCTCCTACAATACAAACAAATAAAGTTAATAATT

TTAAATAAGAAGGAAGAACTACTACTACTGGTGTAGGAAAAATTAATCAACTTAATATAC

TACCCCCAAAAATTCTCAAAATTAATAGTCCTATTATACTTTTTAATATAACTCAACCTT

CATCATTTAGTATATTTAAAGAAGAAAAATTTGAATCCCCA

GTTGTCTTCGCTCCCGCTAACATCACCACTGAAGTCAAGT

CCGTAGAAATGCACCACGAAGCTCTCGTTGAAGCTGTTCCCGGTGACAACGTTGGTTTCA

ACGTTAAGAACGTCTCCGTCAAGGAATTGCGTCGTGGTTACGTCGCTGGTGACTCCAAGG

CTAGCCCCCCCAAGGGTGCTGCTGACTTCACCGCTCAAGTCATCGTCTTGAACCACCCTG

GTCAAATCTCCAACGGTTACACTCCCGTTTTGGATTGTCACACCGCTCACATTGCTTGCA

AATTCGCCGAAATCAAGGAGAAGGTCGATTGAAGCACG

CAGTACCCTGAGTCCGGTTCAGTGCTTTGAGGGTAGCGGTGGTAGTGGATCCTCGGGAAA

TTTTACTTCGGGTAGCAACTTGAATATGGGCAGTGTTACTAATACAAGCAATACCGGTAC

AGGCACTTCATCGGGAAGTGTACCCATGGTTACGTTGACGGAATCTCTACTAAATAAACA

CAACGATGAAATGGAAAAGTTTATGCTAAAGAAACACAGAGAATCTCGCGGACGAAGTGG

TGAGAAGAATAAAAAGAACTCGGAAAAGATAATGGAGTATACTGGTCCTGGGCATGGAAT

AAAGAGAGTTGGCTCACA

>ITMO2Li

AGAATTCTAATTCGAGCTGAATTAGGACACC
CTGGTGCACTAATTGGAGATGACCAAATTTATAATGTAATTGTTACAGCTCATGCTTTTA
TTATAATTTTCTTTATAGTAATACCAATTATAATTGGAGGATTTGGTAATTGATTAGTTC
CTTTAATGTTAGGAGCCCCAGATATAGCCTTCCCTCGATTAAATAATATAAGATTTTGAC
TTTTACCTCCTGCACTAACTTTATTATTAGTAAGTAGTATAGTAGAAAACGGAGCTGGAA
CAGGATGAACAGTTTACCCCCCTTTATCATCTAATATCGCTCATGGAGGAGCTTCTGTTG
ATTTAGCTATTTTTTCTCTTCATTTAGCAGGAATTTCTTCAATTTTAGGGGCTGTAAATT
TTATTACTACAGTCATTAATATACGATCAACAGGAATTACTTTTGACCGAATACCTTTAT
TTGTTTGATCAGTTGTAATTACAGCTCTATTACTTTTATTATCATTACCAGTATTAGCAG
GAGCTATTACAATACTTTTAACTGACCGAAACCTTAATACTTCATTTTTTGACCCTGCAG
GAGGAGGAGACCCAATTCTATACCAACATCTATTTAATAATTAATAATTCCGTAAGTAGAAATATAAGGTATAAATCATA

TTGATCCTAAAAAATAAGAAAAATTATAATTATTTAAAGCCTTATTATAAAAAAATAAAG

AAATATTTGAAATTATATAACCTCTTACTCCTCCTACAATACAAACAAATAATGTTAACA

ACTTTAAATATGAAGGTAATACTACTACTACTGGGGTAGGAAAAATTAATCATCTTAACA

TTCTCCCCCCAAAAATTCTTAAAATTAATAAACCTATTATACTTTTTAATATAACTCAAC

CTTCATCATTTAATATATTTAAAGAAGAAAAATTTGAATCACCA

GTTGTCTTCGCTCCCGCTAA

CATCACCACTGAAGTCAAGTCCGTTGAAATGCACCACGAAGCTCTCGTTGAAGCCGTTCC

CGGTGACAACGTTGGTTTCAACGTAAAGAACGTCTCCGTCAAGGAATTGCGTCGTGGTTA

CGTCGCTGGTGACTCCAAGGCCAGCCCCCCCAAGGGTGCCGCTGACTTCACCGCTCAAGT

CATCGTCTTGAACCATCCCGGTCAAATCTCCAACGGTTACACTCCCGTTTTGGATTGTCA

CACCGCTCACATTGCTTGCAAATTCGCCGAAATCAAGGAGAAGGTCGAT CGATGCACGCAGTACTTTGAGTCCGGTTCAGTGCTTTGAAGGCAGCGGTGGTAG

TGGTTCATCGGGAAATTTTACTTCGGGCAGTAATTTGAATATGGGTAGTGTGACAAATAC

CAGCAACACCGGTACCGGCACTTCATCGGGAAGTGTTCCCATGGTTACGTTAACGGAATC

TCTACTAAATAAACATAACGATGAAATGGAAAAGTTTATGCTAAAGAAACATAGAGAATC

TCGTGGACGAAGTGGTGAGAAGAATAAAAAGAATTCTGAAAAGATAATGGAGTATACTGG

ACCGGGGCATGGAATCAAAAGAGTTGGATCTCA

>4ITCvi

AGAATTCTAATTCGAGCCGAACTAGGAC
ATCCTGGAGCATTAATTGGAGATGACCAAATTTATAATGTAATTGTTACAGCTCATGCTT
TTATTATAATTTTTTTTATAGTAATACCAATTATAATTGGAGGATTTGGTAATTGATTAG
TCCCTTTAATATTAGGAGCTCCAGATATAGCCTTCCCTCGGATAAACAATATAAGTTTCT
GACTTTTACCTCCTGCATTAACTTTACTATTAGTAAGTAGTATAGTAGAAAACGGAGCTG
GAACAGGATGAACTGTTTACCCACCTTTATCTTCTAATATCGCTCATGGAGGAGCTTCTG
TTGATTTAGCTATTTTTTCTTTACACTTAGCAGGAATTTCTTCAATTTTAGGAGCTGTAA
ATTTTATTACTACAGTTATTAATATACGATCAACAGGAATTACATTCGACCGAATACCAT
TATTTGTTTGATCTGTAGTAATTACAGCTTTATTACTTTTATTATCTTTACCAGTATTAG
CAGGTGCTATTACTATATTATTAACAGATCGAAATCTTAATACATCATTCTTTGACCCAG
CAGGAGGAGGAGACCCAATCTTGTACCAACATTTATTT

AATAATTAATAATTCCATATGTAGAAATATAAGGTATAAATCATATTGA

ACCTAAAAAGTAAGAAGAATTATAATTATTTAAAGCCTTATTATAAAAAAATAAAGAAAT

ATTAGAAATTAAATAACCTCTAACTCCTCCTACAATACAAACAAATAAAGTTAATAATTT

TAAATAAGAAGGAAGAACTACTACTACTGGTGTAGGAAAAATTAATCAACTTAATATACT

ACCCCCAAAAATTCTCAAAATTAATAGTCCTATTATACTTTTTAATATAACTCAACCTTC

ATCATTTAGTATATTTAAAGAAGAAAAATTTGAATCCCCA

GTTGTCTTCGCTCCCGCTAACATCACCACTG

AAGTCAAGTCCGTAGAAATGCACCACGAAGCTCTCGTTGAAGCTGTTCCCGGTGACAACG

TTGGTTTCAACGTTAAGAACGTCTCCGTCAAGGAATTGCGTCGTGGTTACGTCGCTGGTG

ACTCCAAGGCTAGCCCCCCCAAGGGTGCTGCTGACTTCACCGCTCAAGTCATCGTCTTGA

ACCACCCTGGTCAAATCTCCAACGGTTACACTCCCGTTTTGGATTGTCACACCGCTCACA

TTGCTTGCAAATTCGCCGAAATCAAGGAAAAGGTCGAT TGAAGC

ACGCAGTACCCTGAGTCCGGTTCAGTGCTTTGAGGGTAGCGGTGGTAGTGGATCCTCAGG

AAATTTTACTTCGGGTAGCAACTTGAATATGGGCAGTGTTACTAATACAAGCAATACCGG

TACAGGCACTTCATCGGGAAGTGTACCCATGGTTACGTTGACGGAATCTCTACTAAATAA

ACACAACGATGAAATGGAAAAGTTTATGCTAAAGAAACACAGAGAATCTCGCGGACGAAG

TGGTGAGAAGAATAAAAAGAACTCGGAAAAGATAATGGAGTATACTGGTCCTGGGCATGG

AATAAAGAGAGTTGGCTCACA

>ITMO1Cvo

AGAATTTTAATTCGAGCTGAACTAGGGCATC
CTGGAGCATTAATTGGAGATGATCAAATTTATAATGTAATTGTTACAGCTCATGCTTTTA
TTATAATTTTTTTTATAGTAATACCAATTATAATTGGAGGGTTTGGAAATTGATTAGTTC
CTTTAATATTAGGAGCCCCAGATATAGCATTCCCTCGAATAAATAATATAAGTTTCTGAC
TTTTACCTCCTGCATTAACTTTACTATTAGTAAGTAGTATAGTAGAAAACGGAGCTGGAA
CTGGATGAACTGTTTATCCACCTTTATCTTCTAATATTGCACATGGAGGAGCTTCTGTTG
ATTTAGCTATTTTTTCTTTACATTTAGCAGGAATTTCTTCAATTTTAGGAGCTGTAAATT
TTATTACTACAGTTATTAATATACGATCAACAGGTATTACCTTCGACCGAATACCATTAT
TTGTTTGATCAGTAGTAATTACAGCCTTATTACTTTTATTATCTTTACCAGTATTAGCAG
GAGCTATTACTATATTATTAACAGATCGAAATCTTAATACTTCATTCTTTGACCCAGCAG
GAGGAGGAGATCCAATTTTATACCAACACTTATTT

AGTAATTAATAATTCCGTATGTAGAAATATAAGGCATAAACCACA

TTGACCCTAAAAAATAAGAAGAATTATAGTTATTTAAAGCCTTATTATAAAAAAATAAAG

AAATATTAGAAATTAAATACCCTCTAATTCCTCCTACAATACAAACAAATAAAGTTAATA

ACTTTAAATAAGAAGGTAGAACTACTACTACTGGTGTAGGAAAAATTAATCAACTTAACA

TTCTACCCCCAAAAATTCTTAAAATTAATAACCCTATTATACTCTTTAACATAACTCAAC

CTTCATCATTTAATATATTTAAAGAAGAAAAATTTGAATCCCCAGTTGTCTTCGCTCCCGCTAACATCACCACTGAAGT

CAAATCCGTAGAAATGCACCACGAAGCTCTCGTTGAAGCCGTCCCCGGTGACAACGTTGG

TTTCAACGTTAAGAACGTCTCCGTCAAGGAATTGCGTCGTGGTTACGTCGCTGGTGACTC

CAAGGCTAGCCCCCCCAAGGGTGCTGCTGACTTCACCGCTCAAGTCATCGTCTTGAACCA

CCCTGGTCAAATCTCCAACGGTTACACTCCCGTTTTGGATTGTCACACCGCTCACATTGC

TTGCAAATTCGCCGAAATCAAGGAGAAGGTCGAT TGA

AGCACGCAGTACCCTGAGTCCGGTTCAGTGCTTTGAGGGTAGCGGTGGTAGTGGATCCTC

AGGAAATTTTACTTCGGGCAGCAACTTGAATATGGGCAGTGTTACTAATACAAGCAATAC

CGGTACAGGCACTTCATCGGGAAGTGTACCCATGGTTACGTTGACGGAATCTCTACTAAA

TAAACACAACGATGAAATGGAAAAGTTTATGCTAAAGAAACACAGAGAATCTCGCGGACG

AAGTGGTGAGAAGAATAAAAAGAACTCGGAAAAGATAATGGAGTATACTGGTCCTGGGCA

TGGAATAAAGAGAGTTGGCTCACA

>5ITCvi

AGAATTCTAATTCGAGCCGAACTAGGACA
TCCTGGAGCATTAATTGGAGATGACCAAATTTATAATGTAATTGTTACAGCTCATGCTTT
TATTATAATTTTTTTTATAGTAATACCAATTATAATTGGAGGATTTGGTAATTGATTAGT
CCCTTTAATATTAGGAGCTCCAGATATAGCCTTCCCTCGGATAAACAATATAAGTTTCTG
ACTTTTACCTCCTGCATTAACTTTACTATTAGTAAGTAGTATAGTAGAAAACGGAGCTGG
AACAGGATGAACTGTTTACCCACCTTTATCTTCTAATATCGCTCATGGAGGAGCTTCTGT
TGATTTAGCTATTTTTTCTTTACACTTAGCAGGAATTTCTTCAATTTTAGGAGCTGTAAA
TTTTATTACTACAGTTATTAATATACGATCAACAGGAATTACATTCGACCGAATACCATT
ATTTGTTTGATCTGTAGTAATTACAGCTTTATTACTTTTATTATCTTTACCAGTATTAGC
AGGTGCTATTACTATATTATTAACAGATCGAAATCTTAATACATCATTCTTTGACCCAGC
AGGAGGAGGAGACCCAATCTTGTACCAACATTTATTTAATAATTAATAATTCCATATGTAGAAATATAAGGTATAAATCATATT

GAACCTAAAAAGTAAGAAGAATTATAATTATTTAAAGCCTTATTATAAAAAAATAAAGAA

ATATTAGAAATTAAATAACCTCTAACTCCTCCTACAATACAAACAAATAAAGTTAATAAT

TTTAAATAAGAAGGAAGAACTACTACTACTGGTGTAGGAAAAATTAATCAACTTAATATA

CTACCCCCAAAAATTCTCAAAATTAATAGTCCTATTATACTTTTTAATATAACTCAACCT

TCATCATTTAGTATATTTAAAGAAGAAAAATTTGAATCCCCAGTTGTCTTCGCTCCCGCTAACATCACCACTGAAGTCAAGTCCGTAGAAATGCACCACGAAGCTCTCGTTGAAGCTGTTCCCGGTGACA

ACGTTGGTTTCAACGTTAAGAACGTCTCCGTCAAGGAATTGCGTCGTGGTTACGTCGCTG

GTGACTCCAAGGCTAGCCCCCCCAAGGGTGCTGCTGACTTCACCGCTCAAGTCATCGTCT

TGAACCACCCTGGTCAAATCTCCAACGGTTACACTCCCGTTTTGGATTGTCACACCGCTC

ACATTGCTTGCAAATTCGCCGAAATCAAGGAGAAGGTCGATTGAAGCACGCAGTACCCTGAGTCCGGTTCAGTGCTTTGAGGGTAGCGGT

GGTAGTGGATCCTCGGGAAATTTTACTTCGGGTAGCAACTTGAATATGGGCAGTGTTACT

AATACAAGCAATACCGGTACAGGCACTTCATCGGGAAGTGTACCCATGGTTACGTTGACG

GAATCTCTACTAAATAAACACAACGATGAAATGGAAAAGTTTATGCTAAAGAAACACAGA

GAATCTCGCGGACGAAGTGGTGAGAAGAATAAAAAGAACTCGGAAAAGATAATGGAGTAT

ACTGGTCCTGGGCATGGAATAAAGAGAGTTGGCTCACA

>6ITCvi

AGAATTCTAATTCGAGCCGAACTAGGACAT
CCTGGAGCATTAATTGGAGATGACCAAATTTATAATGTAATTGTTACAGCTCATGCTTTT
ATTATAATTTTTTTTATAGTAATACCAATTATAATTGGAGGATTTGGTAATTGATTAGTC
CCTTTAATATTAGGAGCTCCAGATATAGCCTTCCCTCGGATAAACAATATAAGTTTCTGA
CTTTTACCTCCTGCATTAACTTTACTATTAGTAAGTAGTATAGTAGAAAACGGAGCTGGA
ACAGGATGAACTGTTTACCCACCTTTATCTTCTAATATCGCTCATGGAGGAGCTTCTGTT
GATTTAGCTATTTTTTCTTTACACTTAGCAGGAATTTCTTCAATTTTAGGAGCTGTAAAT
TTTATTACTACAGTTATTAATATACGATCAACAGGAATTACATTCGACCGAATACCATTA
TTTGTTTGATCTGTAGTAATTACAGCTTTATTACTTTTATTATCTTTACCAGTATTAGCA
GGTGCTATTACTATATTATTAACAGATCGAAATCTTAATACATCATTCTTTGACCCAGCA
GGAGGAGGAGACCCAATCTTGTACCAACATTTATTT

AATAATTAATAATTCCATATGTAGAAATATAAGGTATAAATCATATTGA

ACCTAAAAAGTAAGAAGAATTATAATTATTTAAAGCCTTATTATAAAAAAATAAAGAAAT

ATTAGAAATTAAATAACCTCTAACTCCTCCTACAATACAAACAAATAAAGTTAATAATTT

TAAATAAGAAGGAAGAACTACTACTACTGGTGTAGGAAAAATTAATCAACTTAATATACT

ACCCCCAAAAATTCTCAAAATTAATAGTCCTATTATACTTTTTAATATAACTCAACCTTC

ATCATTTAGTATATTTAAAGAAGAAAAATTTGAATCCCCAGTTGTCTTCGCTCCCGCTAACATCACCACTGAAG

TCAAGTCCGTAGAAATGCACCACGAAGCTCTCGTTGAAGCTGTTCCCGGTGACAACGTTG

GTTTCAACGTTAAGAACGTCTCCGTCAAGGAATTGCGTCGTGGTTACGTCGCTGGTGACT

CCAAGGCTAGCCCCCCCAAGGGTGCTGCTGACTTCACCGCTCAAGTCATCGTCTTGAACC

ACCCTGGTCAAATCTCCAACGGTTACACTCCCGTTTTGGATTGTCACACCGCTCACATTG

CTTGCAAATTCGCCGAAATCAAGGAGAAGGTCGATTGA

AGCACGCAGTACCCTGAGTCCGGTTCAGTGCTTTGAGGGTAGCGGTGGTAGTGGATCCTC

AGGAAATTTTACTTCGGGTAGCAACTTGAATATGGGCAGTGTTACTAATACAAGCAATAC

CGGTACAGGCACTTCATCGGGAAGTGTACCCATGGTTACGTTGACGGAATCTCTACTAAA

TAAACACAACGATGAAATGGAAAAGTTTATGCTAAAGAAACACAGAGAATCTCGCGGACG

AAGTGGTGAGAAGAATAAAAAGAACTCGGAAAAGATAATGGAGTATACTGGTCCTGGGCA

TGGAATAAAGAGAGTTGGCTCACA

>9ITCvi

AGAATTCTAATTCGAGCCGAACTAGGACATCCTGGA
GCATTAATTGGAGATGACCAAATTTATAATGTAATTGTTACAGCTCATGCTTTTATTATA
ATTTTTTTTATAGTAATACCAATTATAATTGGAGGATTTGGTAATTGATTAGTCCCTTTA
ATATTAGGAGCTCCAGATATAGCCTTCCCTCGGATAAACAATATAAGTTTCTGACTTTTA
CCTCCTGCATTAACTTTACTATTAGTAAGTAGTATAGTAGAAAACGGAGCTGGAACAGGA
TGAACTGTTTACCCACCTTTATCTTCTAATATCGCTCATGGAGGAGCTTCTGTTGATTTA
GCTATTTTTTCTTTACACTTAGCAGGAATTTCTTCAATTTTAGGAGCTGTAAATTTTATT
ACTACAGTTATTAATATACGATCAACAGGAATTACATTCGACCGAATACCATTATTTGTT
TGATCTGTAGTAATTACAGCTTTATTACTTTTATTATCTTTACCAGTATTAGCAGGTGCT
ATTACTATATTATTAACAGATCGAAATCTTAATACATCATTCTTTGACCCAGCAGGAGGA
GGAGACCCAATCTTGTACCAACATTTATTTAATAATTAATAATTCCATATGTAGAAATATAAGGTATAAATCATA

TTGAACCTAAAAAGTAAGAAGAATTATAATTATTTAAAGCCTTATTATAAAAAAATAAAG

AAATATTAGAAATTAAATAACCTCTAACTCCTCCTACAATACAAACAAATAAAGTTAATA

ATTTTAAATAAGAAGGAAGAACTACTACTACTGGTGTAGGAAAAATTAATCAACTTAATA

TACTACCCCCAAAAATTCTCAAAATTAATAGTCCTATTATACTTTTTAATATAACTCAAC

CTTCATCATTTAGTATATTTAAAGAAGAAAAATTTGAATCCCCAGTTGTCTTCGCTCCCGCTAACATCACCACTGAAGTC

AAGTCCGTAGAAATGCACCACGAAGCTCTCGTTGAAGCTGTTCCCGGTGACAACGTTGGT

TTCAACGTTAAGAACGTCTCCGTCAAGGAATTGCGTCGTGGTTACGTCGCTGGTGACTCC

AAGGCTAGCCCCCCCAAGGGTGCTGCTGACTTCACCGCTCAAGTCATCGTCTTGAACCAC

CCTGGTCAAATCTCCAACGGTTACACTCCCGTTTTGGATTGTCACACCGCTCACATTGCT

TGCAAATTCGCCGAAATCAAGGAGAAGGTCGATTGAAGCACGCAGTACCCTGAGTCCGGTTCAGTGCTTTGAGGGTAGCGGTGGTAGTG

GATCCTCGGGAAATTTTACTTCGGGTAGCAACTTGAATATGGGCAGTGTTACTAATACAA

GCAATACCGGTACAGGCACTTCATCGGGAAGTGTACCCATGGTTACGTTGACGGAATCTC

TACTAAATAAACACAACGATGAAATGGAAAAGTTTATGCTAAAGAAACACAGAGAATCTC

GCGGACGAAGTGGTGAGAAGAATAAAAAGAACTCGGAAAAGATAATGGAGTATACTGGTC

CTGGGCATGGAATAAAGAGAGTTGGCTCACA

>ITTVLc2

AGAATTTTAATTCGAGCTGAATTAGGACACC
CTGGTGCATTAATTGGAGATGACCAAATTTATAATGTAATTGTTACAGCTCATGCTTTTA
TTATAATTTTCTTTATAGTAATACCAATTATAATTGGAGGATTTGGAAATTGATTAGTTC
CTTTAATATTAGGAGCCCCAGATATAGCATTCCCTCGAATAAATAATATAAGTTTTTGAC
TTTTACCTCCTGCATTAACTTTATTATTAGTAAGTAGTATAGTAGAAAACGGAGCTGGAA
CAGGATGAACAGTTTACCCTCCTTTATCATCTAATATCGCTCATGGAGGAGCTTCTGTTG
ATTTAGCTATTTTTTCTCTTCATTTAGCAGGAATTTCATCAATTTTAGGAGCTGTAAATT
TTATTACTACAGTTATTAATATACGATCAACAGGAATTACTTTTGACCGAATACCTTTAT
TCGTTTGATCAGTAGTAATTACAGCTTTATTACTTTTATTATCATTACCAGTATTAGCTG
GAGCTATTACTATACTTTTAACTGATCGAAATCTTAATACTTCATTCTTTGATCCAGCAG
GAGGAGGAGACCCAATTTTATACCAACATTTATTTAATAATTAATAATCCCGTAAGTAGAAATATAAGGTATAAACCATAT

TGACCCTAAAAAATAAGAAAAATTATAATTATTTAAAGCCTTATTATAAAAAAATAAAGA

AATATTTGAAATTATATACCCTCTTACTCCTCCTACAATACAAACAAATAATGTTAATAA

CTTCAAATATGAAGGAAGAACTACAACTACTGGAGTTGGAAAAATTAATCATCTTAATAT

TCTTCCTCCAAAAATTCTTAAAATTAATAACCCTATTATACTCTTTAATATTACTCAACC

TTCATCATTTAATATATTTAAAGAAGAAAAATTTGAATCACCAGTTGTCATCGCTCCCGCTAACATCACCACTGAAGTCAAGTCCGTTGAAATGCACCACGAA

GCTCTCGTTGAAGCCGTCCCCGGTGACAACGTTGGTTTCAACGTAAAGAACGTCTCCGTC

AAGGAATTGCGTCGTGGTTACGTCGCTGGTGACTCCAAGGCCAACCCCCCCAAGGGTGCC

GCTGACTTCACCGCTCAAGTCATCGTCTTGAACCATCCCGGTCAAATCTCCAACGGTTAC

ACTCCCGTTTTGGATTGTCACACCGCTCACATTGCTTGCAAATTCGCCGAAATCAAGGAG

AAGGTCGATTGATGCACGCAGTGCTTTGAGTCCGGTTCAGTGCTTTGAAGGCAGCGGTGGTAG

TGGTTCATCGGGAAATTTTACTTCGGGCAGTAACTTGAATATGGGCAGTGTGACTAATAC

CAGCAACACTGGTACCGGCACTTCCTCGGGAAGTGTTCCCATGGTTACATTAACGGAATC

TCTGCTAAATAAGCATAACGATGAAATGGAAAAATTTATGCTAAAAAAGCACAGAGAATC

TCGTGGACGAAGTGGCGAGAAGAATAAAAAGACTTCTGAAAAGATAATGGAATATACTGG

ACCGGGGCATGGAATCAAAAGAGTTGGATCACA

>ITTVCvo1

AGAATTTTAATTCGAGCTGAACTAGGGCA
TCCTGGAGCATTAATTGGAGATGATCAAATTTATAATGTAATTGTTACAGCTCATGCTTT
TATTATAATTTTTTTTATAGTAATACCAATTATAATTGGAGGGTTTGGAAATTGATTAGT
TCCTTTAATATTAGGAGCCCCAGATATAGCATTCCCTCGAATAAATAATATAAGTTTCTG
ACTTTTACCTCCTGCATTAACTTTACTATTAGTAAGTAGTATAGTAGAAAACGGAGCTGG
AACTGGATGAACTGTTTATCCACCTTTATCTTCTAATATTGCACATGGAGGAGCTTCTGT
TGATTTAGCTATTTTTTCTTTACATTTAGCAGGAATTTCTTCAATTTTAGGAGCTGTAAA
TTTTATTACTACAGTTATTAATATACGATCAACAGGTATTACCTTCGACCGAATACCATT
ATTTGTTTGATCAGTAGTAATTACAGCCTTATTACTTTTATTATCTTTACCAGTATTAGC
AGGAGCTATTACTATATTATTAACAGATCGAAATCTTAATACTTCATTCTTTGACCCAGC
AGGAGGAGGAGATCCAATTTTATACCAACACTTATTTAGTAATTAATAATTCCGTATGTAGAAATATAAGAGCATAAACCACATTGA

CCCTAAAAAATAAGAAGAATTATAGTTATTTAAAGCCTTATTATAAAAAAATAAAGAAAT

ATTAGAAATTAAATACCCTCTAATTCCTCCTACAATACAAACAAATAAAGTTAATAACTT

TAAATAAGAAGGTAGAACTACTACTACTGGTGTAGGAAAAATTAATCAACTTAACATTCT

ACCCCCAAAAATTCTTAAAATTAATAACCCTATTATACTCTTTAACATAACTCAACCTTC

ATCATTTAATATATTTAAAGAAGAAAAATTTGAATCCCCAGTTGTCATCGCTCCCGCTAACATCACCACTGAAGTCAAATC

CGTAGAAATGCACCACGAAGCTCTCGTTGAAGCCGTCCCCGGTGACAACGTTGGTTTCAA

CGTTAAGAACGTCTCCGTCAAGGAATTGCGTCGTGGTTACGTCGCTGGTGACTCCAAGGC

TAGCCCCCCCAAGGGTGCTGCTGACTTCACCGCTCAAGTCATCGTCTTGAACCACCCTGG

TCAAATCTCCAACGGTTACACTCCCGTTTTGGATTGTCACACCGCTCACATTGCTTGCAA

ATTCGCCGAAATCAAGGAGAAGGTCGATTGAAGCACGCAGTACCCTGAGTCCGGTTCAGTGCTTTGAGGGTAGCGGTGGTAGT

GGATCCTCAGGAAATTTTACTTCGGGCAGCAACTTGAATATGGGCAGTGTTACTAATACA

AGCAATACCGGTACAGGCACTTCATCGGGAAGTGTACCCATGGTTACGTTGACGGAATCT

CTACTAAATAAACACAACGATGAAATGGAAAAGTTTATGCTAAAGAAACACAGAGAATCT

CGCGGACGCAGTGGTGAGAAGAATAAAAAGAACTCGGAAAAGATAATGGAGTATACTGGT

CCTGGGCATGGAATAAAGAGAGTTGGCTCACA

>DataLse1

AGAATTCTAATTCGAGCT

GAATTAGGACATCCTGGAGCTTTAATTGGAGATGATCAAATTTATAATGTAATTGTTACAGCTCATGCTT

TTATTATAATTTTTTTTATAGTAATGCCAATTATAATTGGAGGATTTGGAAATTGATTAGTTCCATTAAT

ACTAGGAGCTCCAGATATAGCATTCCCTCGAATAAATAATATAAGTTTTTGACTTTTACCTCCTGCATTA

ACTTTATTATTAGTTAGTAGTATAGTAGAAAACGGAGCTGGAACAGGATGAACAGTTTACCCTCCTCTAT

CTTCTAATATTGCTCATGGAGGAGCTTCTGTTGATTTAGCTATTTTCTCTCTTCATTTAGCAGGAATTTC

TTCAATTTTAGGAGCTGTAAATTTTATTACTACAGTTATTAATATACGATCAACAGGAATTACTTTTGAT

CGAATACCTTTATTTGTTTGATCAGTAGTAATTACAGCTTTATTACTTTTATTATCATTACCAGTATTAG

CAGGAGCTATTACAATACTTTTAACAGACCGAAATCTTAATACATCATTCTTTGACCCTGCAGGAGGAGG

AGATCCAATTTTATACCAACATTTATTTAATAATTAATAATTCCATAAGTAGAAATATAAGGTATAAACCACATAGAACCTAAAA

AATAAGAAAAATTATAATTATTTAAAGCCTTATTATAAAAAAATAAAGAAACATGAGAAATTATATAACC

TATAATACCCCCAACAATACAAACAAATAATGTTAATAATTTTAAATAAGAAGGAAGAACAACTACTATA

GGAGTAGGAAAAATCAATCATCTTAACATTCTCCCACCAAAAATTCTTAAAATTAATAAACCTATTATAC

TTTTCAATATTACTCAACCTTCATCATTTAATATATTTAAAGAAGAAAAATTAGAATCACCAGTTGTCTTCGCYCCCGCTAACATCACCA

CTGAAGTCAAGTCCGTTGAAATGCACCACGAAGCTCTCGTTGAAGCCGTCCCCGGTGACAACGTTGGTTTCAACGTAAAGAACGTCTCCGTCAAGGAATTGCGTCGTGGTTACGTCGCTGGTGACTCCAAGGCYAACCCCCCCAAGGGCGCCGCTGACTTCACCGCTCAAGTCATCGTCTTGAACCACCCTGGTCAAATCTCCAACGGTTACACCCCCGTTTTGGATTGTCACACCGCTCACATTGCTTGCAAATTCGCCGAAATCAAGGAGAAGGTCGATGGATGCACGCAGTACCCTAAGTCCGGTTCAGTGCTTTGAGGGCAGCGGTGGAAGTGGTTCATCGGGAAATTTTACTTCGGGC

AGTAACTTAAATATGGGCAGTGTGACTAATACCAGCAACACCGGTACTGGTACTTCCTCAGGAAGKGTTC

CCATGGTCACGTTGACSGAATCTCTACTAAATAAACATAACGATGAAATGGAAAAGTTTATGCTTAAGAA

GCATAGAGAATCTCGAGGACGAAGTGGCGAGAAGAACAAAAAGAATTCTGAAAAGATAATGGAGTACACT

GGCCCGGGACACGGAATCAAAAGAGTTGGTTCACA

>DataLill1

AGAATTTTAATTCGAGCTGAATTAGGACACCCTGGTGCATTAATTGGAGATGACCAAATTTATAATGTAATTGTTACAGCTCATGCTTTTATTATAATTTTCTTTATAGTAATGCCAATTATAATTGGAGGATTTGGAAATTGATTAGTTCCTTTAATATTAGGAGCTCCAGATATAGCATTCCCTCGAATAAATAATATAAGATTTTGACTTTTACCTCCTGCATTAACTTTACTATTAGTAAGTAGTATAGTAGAAAACGGAGCTGGAACAGGATGAACAGTTTACCCTCCTTTATCATCTAATATCGCTCATGGAGGAGCTTCTGTTGATTTAGCTATTTTTTCTCTTCATTTAGCAGGAATTTCATCAATTTTAGGAGCTGTAAATTTTATTACTACAGTTATTAATATACGATCAACAGGGATTACTTTTGAT

CGAATGCCTTTATTCGTTTGATCAGTAGTAATTACAGCTTTATTACTTTTATTATCATTACCAGTATTAG

CTGGAGCTATTACTATACTTTTAACTGATCGAAATCTTAATACTTCATTCTTTGACCCAGCAGGAGGAGG

AGACCCAATTTTATACCAACATTTATTTAATAATTAATAATTCCGTAAGTAGAAATATAAGGTATAAACCACATTGACCCTAAAAAATAAGAAAAATTATAATTATTTAAAGCCTTATTATAAAAAAATAAGGAAATATTTGAAATTATATACCCTCTTACTCCTCCTACAATACAAACAAATAATGTTAATAACTTTAAATATGAAGGAAGAACTACAACTACTGGAGTTGGAAAAATTAATCATCTTAATATTCTTCCTCCAAAAATTCTTAAAATTAATAATCCTATTATACTTTTTAATATTACTCAACCTTCATCATTTAATATATTTAAAGAAGAAAAATTTGAATCACCAGTTGTCTTCGCTCCCGCTAACATCACCACTGAAGTCAAGTCCGTTGAAATGCACCACGAAGCTCTCGTTGAAGCCGTCCCCGGTGACAACGTTGGTTTCAACGTAAAGAACGTCTCCGTCAAGGAATTGCGTCGTGGTTACGTCGCTGGTGACTCCAAGGCCAACCCCCCCAAGGGTGCCGCTGACTTCACCGCTCAAGTCATCGTCTTGAACCATCCCGGTCAAATCTCCAACGGTTACACTCCCGTTTTGGATTGTCACACCGCTCACATTGCTTGCAAATTCGCCGAAATCAAGGAGAAGGTCGATGGATGCACGCAGTACTTTGAGTCCGGTTCAGTGTTTTGAAGGCAGCGGTGGTAGTGGCTCATCGGGAAATTTTACTTCGGGCAGTAACTTGAATATGGGCAGTGTGACTAATACCAGCAACACTGGTACCGGCACTTCCTCGGGAAGTGTTCCCATGGTTACATTAACGGAATCTCTGCTAAATAAACATAACGATGAAATGGAGAAATTTATGCTAAAGAAGCACAGAGAATCTCGTGGACGAAGTGGCGAGAAGAATAAAAAGACATCTGAAAAGATAATGGAATATACTGGACCGGGGCATGGAATCAAAAGAGTTGGTTCACA
